# Metabolic Profiling of a Mapping Population Exposes New Insights in the Regulation of Seed Metabolism and Seed, Fruit, and Plant Relations

**DOI:** 10.1371/journal.pgen.1002612

**Published:** 2012-03-29

**Authors:** David Toubiana, Yaniv Semel, Takayuki Tohge, Romina Beleggia, Luigi Cattivelli, Leah Rosental, Zoran Nikoloski, Dani Zamir, Alisdair R. Fernie, Aaron Fait

**Affiliations:** 1 Max Planck Institute of Molecular Plant Physiology, Potsdam-Golm, Germany; 2 French Associates Institute for Agriculture and Biotechnology of Drylands (FAAB), The Jacob Blaustein Institutes for Desert Research, Ben-Gurion University of the Negev, Sede Boqer, Israel; 3 The Robert H. Smith Institute of Plant Sciences and Genetics in Agriculture, The Hebrew University of Jerusalem, Faculty of Agriculture, Rehovot, Israel; 4 CRA Cereal Research Center, Foggia, Italy; The University of North Carolina at Chapel Hill, United States of America

## Abstract

To investigate the regulation of seed metabolism and to estimate the degree of metabolic natural variability, metabolite profiling and network analysis were applied to a collection of 76 different homozygous tomato introgression lines (ILs) grown in the field in two consecutive harvest seasons. Factorial ANOVA confirmed the presence of 30 metabolite quantitative trait loci (mQTL). Amino acid contents displayed a high degree of variability across the population, with similar patterns across the two seasons, while sugars exhibited significant seasonal fluctuations. Upon integration of data for tomato pericarp metabolite profiling, factorial ANOVA identified the main factor for metabolic polymorphism to be the genotypic background rather than the environment or the tissue. Analysis of the coefficient of variance indicated greater phenotypic plasticity in the ILs than in the M82 tomato cultivar. Broad-sense estimate of heritability suggested that the mode of inheritance of metabolite traits in the seed differed from that in the fruit. Correlation-based metabolic network analysis comparing metabolite data for the seed with that for the pericarp showed that the seed network displayed tighter interdependence of metabolic processes than the fruit. Amino acids in the seed metabolic network were shown to play a central hub-like role in the topology of the network, maintaining high interactions with other metabolite categories, i.e., sugars and organic acids. Network analysis identified six exceptionally highly co-regulated amino acids, Gly, Ser, Thr, Ile, Val, and Pro. The strong interdependence of this group was confirmed by the mQTL mapping. Taken together these results (i) reflect the extensive redundancy of the regulation underlying seed metabolism, (ii) demonstrate the tight co-ordination of seed metabolism with respect to fruit metabolism, and (iii) emphasize the centrality of the amino acid module in the seed metabolic network. Finally, the study highlights the added value of integrating metabolic network analysis with mQTL mapping.

## Author Summary

Seeds represent 70% of the food source for man and livestock. However, as a result of millennia of domestication, crop plants have undergone major genetic deterioration, leading to a loss of important quality traits. Thus, the reintroduction of these quality traits is the key to the improvement of crops in modern agriculture. Seed quality traits include nutritional components, such as proteins and amino acids, and seed germination and storability, which are, in turn, inherently related to metabolism. To understand the genetic basis of seed metabolism—a strategic need in the improvement of seed crops—we studied a collection of offspring plants stemming from the cross between a domesticated tomato cultivar *Solanum lycopersicum* cv M82 and its distant wild relative *S. pennellii*. We monitored the changes in metabolism and studied the mode of regulation of the concentration of metabolites in the seeds as a result of genetic introgression, by taking advantage of state-of-the-art technologies and methods of data elaboration such as network-based analysis. We identified a number of candidate genes that may be useful in manipulations to enhance nutritional values in seeds. Finally, in an effort to study the relation among the seed, the fruit, and the mother plant, we determined potential yield-associated metabolic markers.

## Introduction

Tomato is one of the most important crops worldwide (FAO Statistical Database; last updated 2011), being used mainly for human consumption. The tomato fruit contributes essential supplements to the human diet, such as flavonoids, carotenoids [1], fibers [2], vitamins and essential amino acids [3]–[5]. The additional importance of the tomato as a crop plant is that it constitutes the best-studied fruit-bearing model organism [6]–[10], being closely related to potato, eggplant, and pepper—all members of the Solanaceae family [11], [12]. The domestication of tomato, as of many other crop plants, is coupled to the erosion of its genetic variability [13]–[17], leading to the loss of valuable traits. Strategies based on the exploitation of natural variation are being extensively employed [16], [18]–[21] in an effort to reintroduce the lost genetic variation into cultivated species, including tomato [7], rapeseed [22], wheat [23], rice [24], [25], barley [26], soybean [27], maize [28], the common bean [29], and pepper [30]. This approach has led to the generation of mapping populations, facilitating the identification of a vast array of quantitative trait loci (QTL), including loci for yield-related traits, flowering time, fruit quality (in terms of BRIX) and plant-specific organ relations [11], [31]–[35]. Despite the accessibility to these mapping populations, to date, *Arabidopsis* has been almost exclusively used to study the genetic basis of seed traits. Numerous QTL associated with seed size, dormancy [36]–[38] seedling vigor [39], [40], and tolerance to salt [41] has been identified and mapped.

The utility of advanced genetic populations to the study of metabolic traits in plants has been demonstrated by the generation and analysis of a set of 76 introgression lines (ILs) in tomato. This population was developed by crossing the cultivated strain *Solanum lycopersicum* with its distant wild relative *S. pennellii*. The establishment of these tomato ILs has led to the discovery of more than 2,500 QTL associated with plant morphology and fruit metabolism [2], [7], [35], [42]–[44]. Existing studies imply that metabolite levels are generally controlled by multiple genes and are therefore considered as quantitative traits (QT) regulated by metabolite QTL (mQTL).

Seed quality traits, such as protein, starch and oil contents, as well as seed dormancy and vigor, frequently defined as complex traits, are functionally related to C-N balance, central metabolism and sink-source interaction during development on the mother plant [45]–[50]. Although recent studies on seed development [51]–[56] have been invaluable in revealing aspects of the regulation of metabolism, questions concerning the genetic basis of seed trait variability remain open due to the lack of integrative studies on a population scale.

Despite the increased attention currently being paid to enhancing the nutritional values of seed crops [16], [57], [58] and to the economic value of seed quality, the genetic basis regulating the metabolic processes and C-N balance leading to quantitative changes in seed traits has yet to be explored. Moreover, the association between mother plant traits and seed metabolism and vigor has not yet been subjected to genome-scale comparison in crops in general and in tomato in particular.

Here, we analyzed the metabolite contents of dry tomato seeds from two consecutive harvests of a collection of ILs previously used to investigate the fruit pericarp metabolome [2], [35]. MQTL mapping and correlation-based metabolic network analysis facilitated the integration of the generated profiles with metabolic and morphological data collected in earlier studies ([2], [35]; www.phenome-networks.com). The findings are discussed with respect to the recent advances in the regulation of plant metabolism.

## Results

### Metabolic profiling of seeds in a tomato IL population identifies 46 mQTLs and suggests strong post-transcriptional regulation

**Figure 1:**
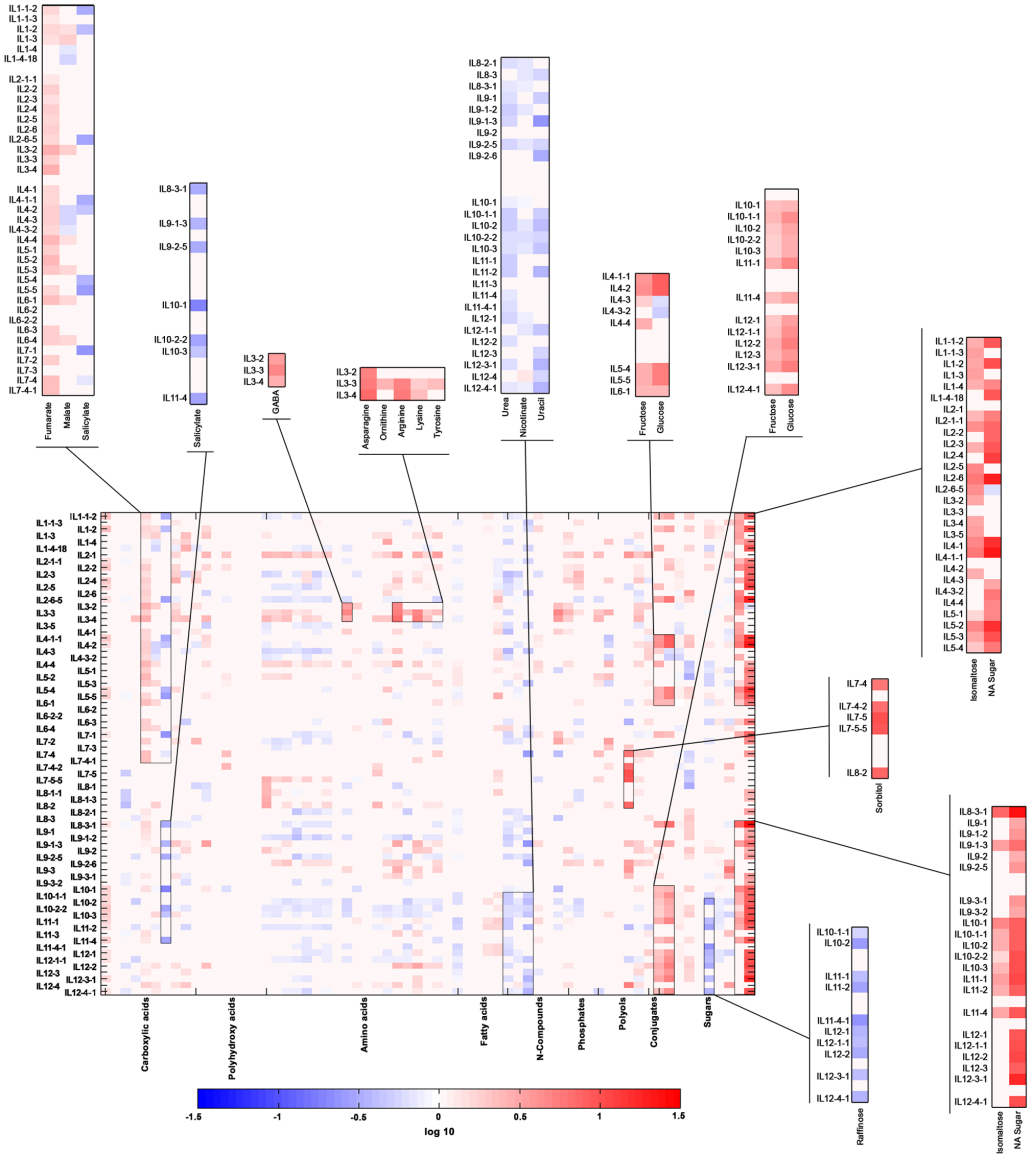
Heat map of metabolites measured across the IL collection during season I. Heat map representation of changes in metabolite levels measured on dry IL seeds of harvest season I in Akko, Israel. Significance of fold change with respect to cultivar M82 (control) was evaluated by Dunnett's test. Blue rectangles indicate a significant decrease in metabolite content, and red rectangles, a significant increase in metabolite content. Pink areas indicate a non-significant change of metabolic concentrations. Metabolites were categorized according to their compound class. A mirror heat map of significance values is given in Figure S1. Groups of metabolites for which changes were highly significant (p<0.001) are delimited within black rectangles and magnified outside the heat map.

**Figure 2:**
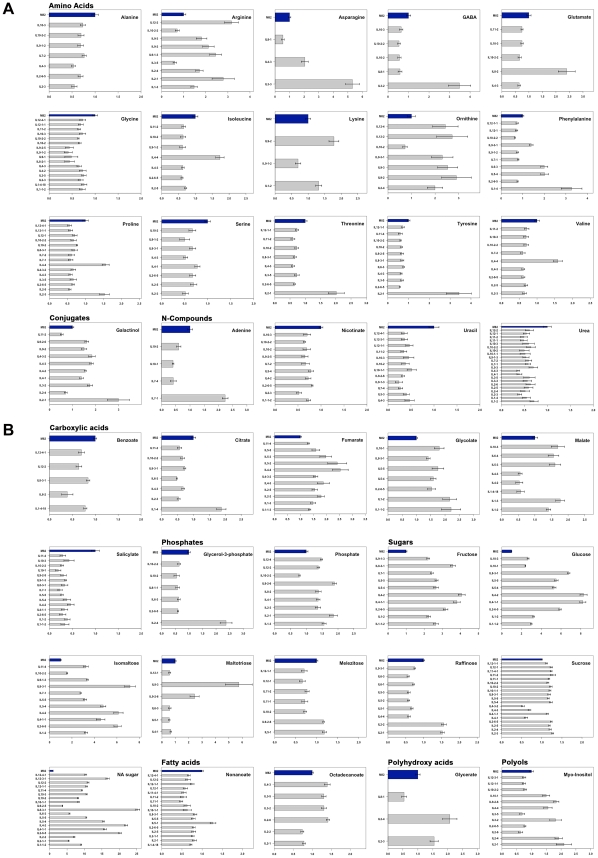
Significant metabolites identified for different ILs in season I. Bar graph representation of significant metabolites identified by Dunnett's-test (p-value<0.05 – after Bonferroni correction) as applied to dry IL seeds of harvest season I in Akko, Israel, in comparison with the control M82. Each bar graph depicts a single metabolite and fold change as compared to M82. Control (M82) levels are shown in dark blue. Metabolites are categorized according to their compound class.

**Figure 3:**
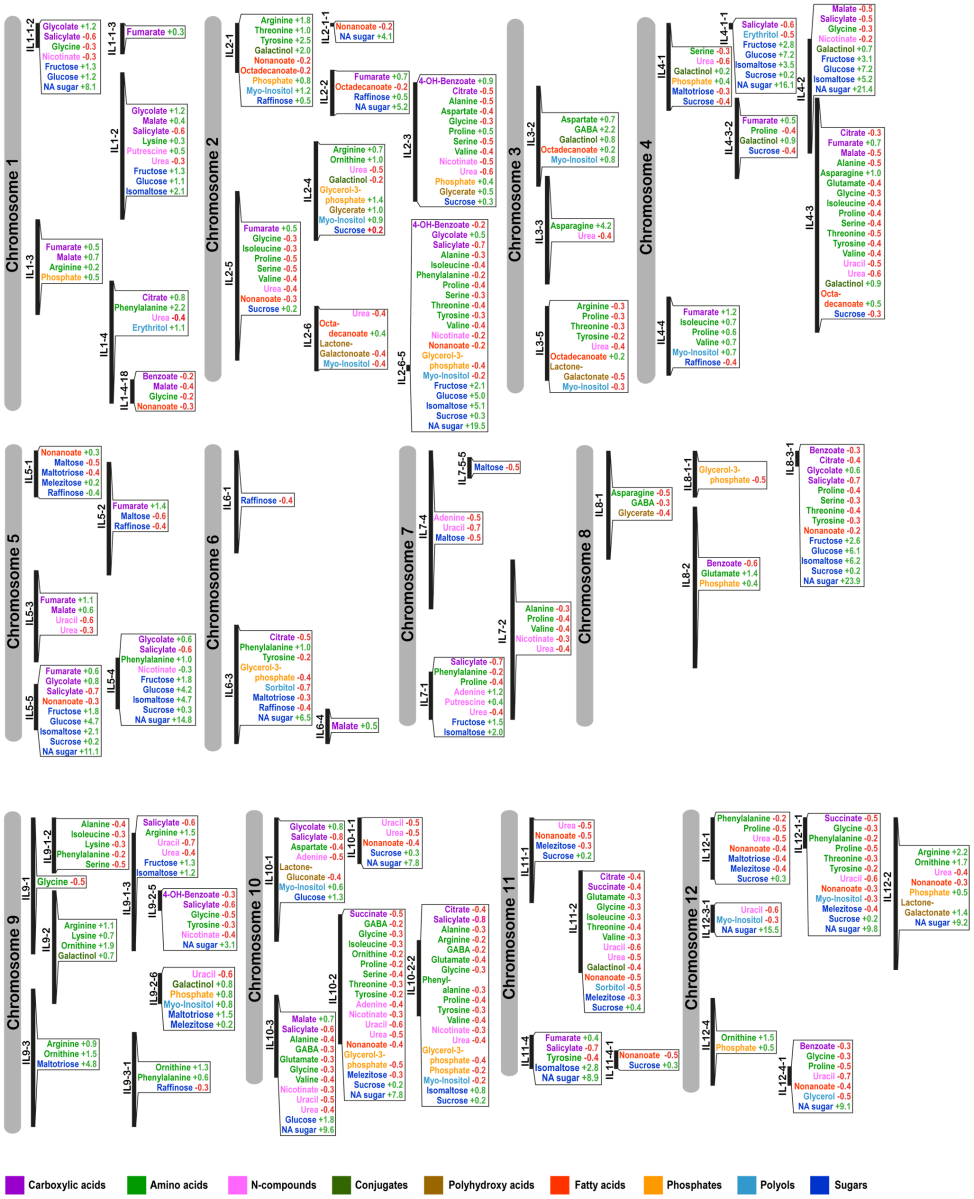
Mapping of significant changes in metabolite content onto introgressed segments. Visualization of significant changes as compared to M82 as identified by Dunnett's test applied to IL season I harvest in Akko, Israel. The chromosomes and the site of introgression are schematically presented with the associated metabolites. Only metabolites exhibiting a significant difference from the control value (p-value<0.05, after the Bonferroni correction) are shown. Values next to metabolites indicate a positive (green) or negative (red), respectively, change in comparison with the value for the control M82. Each value is based on a fold change, e.g., glycolate on IL1-1-2 indicates a +1.2 significant increase = (control) 1+1.2 = 2.2 higher content of glycolate in IL 1-1-2 than in the control M82. Metabolites were categorized according to their compound class (see color key on the Figure).

To identify the potential QTL involved in the regulation of the level of metabolites in the tomato seed, we used a set of 76 ILs resulting from crosses between the domesticated *Solanum lycopersicum* (cv M82) and its distant relative *S. pennellii*, with each IL carrying a small chromosomal portion (5 cM to 75 cM) of the distant relative within the chromosomal background of the domesticated tomato [59]. Seeds harvested from two consecutive seasons [2] of all 76 ILs and of M82, as control, were subjected to GC-MS analysis for metabolic profiling [60], [61]. In total, we unequivocally quantified 64 annotated metabolites across the population in both seasons. We performed one-way ANOVA for each metabolite across the entire population to identify significant changes (p≤<0.05). Almost all metabolites displayed significant changes across the population. Dunnett's test was used to compare the mean of every metabolite of each IL vis-à-vis the control, M82. For the purpose of visualizing cohesiveness of the ILs and grouping of the metabolites, we decided to include changes at the significance level of p≤0.01. In Figure 1 red and blue rectangles depict significant increases and decreases, respectively, of metabolite quantity vis-à-vis the control. Regions with highly significant changes (p<0.001) are delimited by black rectangles and magnified (Figure 1). There were significant changes across the population in four carboxylic acids, fumarate, malate, citrate, and glycolate, in eleven of the proteogenic amino acids, and in fructose, glucose, and one non-annotated sugar (Figure 1). Other metabolites displayed significant changes in few specific lines, such as glycerol in IL 12-4-1, erythritol in ILs 1-4 and 4-1-1, and Lys in ILs 1-2, 9-1-2, and 9-2 (Figure 1). A more detailed analysis of each metabolite displaying significant changes is presented in the bar graphs in Figure 2a, 2b and Figure 3, showing the relative change in metabolic content with reference to M82 (here significant changes were estimated following Bonferroni's correction). For the purpose of visualization, the fold-change of metabolic levels with respect to M82 was used. ILs were characterized by marked alterations in metabolite level; for example: as compared with M82, benzoate was reduced by more than half in IL8-2; citrate was increased on average by 1.8-fold in IL 1-4; fumarate was increased by more than 2.5-fold in IL 4-4 and IL 5-2; malate was significantly increased in ILs 1-2, 1-3, 5-3, 6-4, and 10-3; and glycolate content was more than twofold in IL 1-1-2 and IL 1-2. The vast majority of the amino acids (15 of the initial 19 amino acids) exhibited significant changes throughout the population. In addition, the level of nonanoic acid varied across the population, with one IL (IL 5-1) exhibiting a 1.3-fold increase with respect to M82, whereas all the other ILs showed a decrease in the abundance of this metabolite (from 0.25 to 0.5). For octadecanoate, levels were increased by up to 1.4-fold in IL 2-6, IL 3-2, IL 3-5, and IL 4-3.

In season I, altered sugar content (mostly accumulation) appeared to be a common trait in the population: Glucose displayed an eightfold increase in ILs 4-1-1 and 4-2; isomaltose increased by 7.5-fold in IL 8-3-1, and maltotriose increased by 5.7-fold in IL 9-3. Significant changes were identified for melezitose in seven different lines, for raffinose in nine lines, and for sucrose in 19 different lines. However, for all the loci associated with significant sugar change, only one was reproducible in season II, namely, IL 8-3-1 for glucose.

Despite the large-scale changes described above, a recurring pattern was detected for a set of amino acids in four different introgressions. By constructing a putative mQTL map (Figure 3), we mapped 354 significant changes in metabolite content, represented on the IL map by a color code referring to the different compound classes. However, it should be noted that the total number of significant changes, and hence potential QTL, is an approximation rather than an exact figure. Some of the ILs share overlapping regions, so that the significant changes detected on two overlapping ILs for the same metabolite indicate that the actual QTL is probably be located in the overlap. Thus, each overlapping IL does not represent one separate QTL.

**Figure 4:**
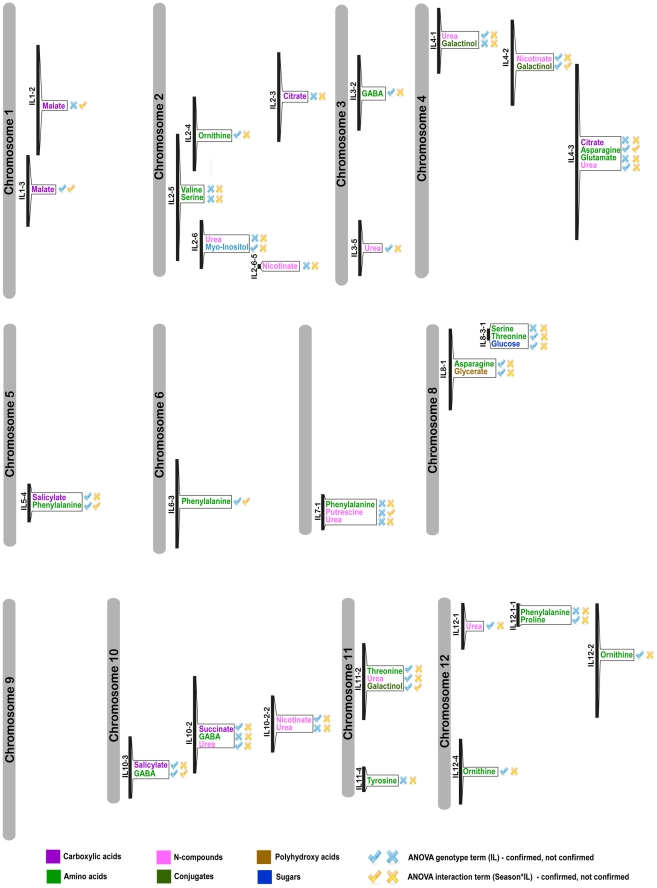
mQTL map of shared significant changes over seasons I and II. GC-MS measurements were performed for consecutive seasons I and II in Akko, Israel; metabolic concentrations were measured in dry seeds. The putative mQTL and their associated metabolites are depicted in the Figure. Symbols next to putative mQTL indicate results of pairwise 2-way ANOVA for each metabolite across the seasons of matching ILs. Blue symbols refer to the single term – genotype; yellow symbols refer to the interaction term – season * genotype. Blue and yellow ticks indicate confirmed by single or interaction term, respectively; blue and yellow crosses indicate not confirmed by single or interaction term, respectively.

The IL map suggests that a distinct group of amino acids (Gly, Ile, Pro, Ser, Val, Thr) are co-localized onto particular introgressions corresponding to ILs 2-3, 2-5, 2-6-5, 4-3 and 11-2. In addition, in IL 10-2 all of the above-named amino acids other than Val were found. Val could, however, be detected in IL 10-3, sharing a large overlap with IL 10-2. Increased levels (>threefold) in central amino acids and the related non-proteinogenic amino acid GABA [62] were shown to occur each in one line only, i.e. IL 3-2 and IL 8-2 (Figure 2a and 2b) respectively, suggesting a specific regulation harbored by each segment, which will probably be worthy of future study. Furthermore, the TCA cycle intermediates fumarate and malate were shown to be localized in close proximity to each other or to co-localize to the same introgressed segment, as, for example, ILs 1-3, 1-1-3, 1-2, 4-2, 4-3, 4-3-2, 5-3 and 5-5, suggesting a shared regulatory mechanism for these two TCA cycle intermediates. More than 30% (123) of all identified significant changes in season I were associated with sugars, of which, fructose, glucose, and sucrose co-localized to many introgressed segments (ILs 2-6-5, 4-1-1, 5-4, 5-5, 8-3-1). In season II, only 13% (n = 46) of the initially identified quantitative metabolite changes (354) were confirmed. A fully annotated map of the conserved metabolic changes (46 putative mQTL) across seasons and their associated loci is presented in Figure 4. Most variability in sugar levels was observed to be subject to seasonal influence. In season I, a consistent 50% reduction in the content of sucrose in ILs 4-1, 4-3, and 4-3-2 was found. In lines 4-1-1 and 4-2, eightfold and fourfold increases relative to M82 were found in the contents of glucose and fructose, respectively. ILs 4-2, 4-3, and 4-3-2 share a large portion of an overlapping chromosomal segment. Despite seasonal fluctuations, the co-localized changes in hexose metabolism indicate tight regulation of sucrose catabolism on chromosome 4. While the confirmed sugar mQTL were constrained to a single locus (IL 8-3-1), amino acids showed the highest degree of shared changes (20 out of 46), particularly Ser, Thr, Phe, GABA, and ornithine. On IL 9-2, an accumulation of the amino acids Lys and ornithine to levels two- and threefold higher, respectively, than those in M82 were detected. There was a fivefold increase for Asn, as compared with the control, on chromosome 3 IL 3-3. In addition, five shared mQTL for the TCA cycle intermediates citrate, succinate, and malate and two more for salicylic acid were identified (Figure 4).

Next, by mapping the changes in seed metabolites onto a chromosome map of the introgressed segments (Figure 3) and comparing it with proposed conserved mQTL in the fruit [2], we identified a few putative shared metabolic traits of fruit and seeds, namely fumarate/malate in the seed and fructose/glucose in the fruit on ILs 1-1-3 and 4-4 and the overlap of ILs 5-4 and 5-5. In addition, the seed mQTL map shows a significant number of overlaps between these organic acids and sugars within the seed as well. To evaluate the genoytpic component and to confirm the conserved putative mQTLs, we performed a pairwise two-way ANOVA for each metabolite in corresponding ILs of the seed datasets for the two seasons; specifically, at an adjusted significance level of 0.05, the genotype term (IL) and the interaction term (season * IL) were investigated. The results are presented in Figure 4, where blue and yellow symbols next to the putative QTL indicate confirmation of the ANOVA at the single and interaction term levels, respectively. In total, 30 out of the 46 previously suggested mQTL were confirmed by the single term (IL), the interaction term (season * IL), or both (IL+season * IL). Interestingly, the proposed QTL for malate in IL1-2 was confirmed solely by the interaction term, while the putative mQTL for malate in IL1-3 was confirmed by both the single and the interaction terms (estimated p-values for the single term are presented in Table S1a, and the interaction term, in Table S1b).

### Abundance of metabolites is affected by genetics, environment, and organ development

For the purpose of testing genetic, environmental, or developmental effects on the changes in metabolite contents across the population, all measurements of the seed metabolites were assembled together with the measurements of the pericarp metabolites (data from [2], [35]). A three-factor ANOVA treatment of the dataset included 3 main factors, 3 two-way interactions, and 1 three-way interaction. The three factors tested with potential impacts on the variance of the different datasets were: Factor A – IL (representing the genetic background), Factor B – organ (seed and fruit), and Factor C – season. Initially, a full-factorial model with all possible interactions and all single and combined effects was designed. The IL factor (genetic background) exhibited the most extensive impact on the variance of the dataset, virtually affecting the content of every metabolite (averaged estimated p value 10^−7^). Yet, all factors contributed significantly to the differences measured. Similar conclusions were drawn for pericarp analyses, as previously described by [35]. In the full factorial model, five metabolites, β-alanine, serine, threonine, succinate, and sucrose, were not affected by factor B (p>0.05). These findings were confirmed by the single factor model, in which GABA and proline were also included. Although the analysis indicated the genotype as the major factor controlling metabolite content in our study, we should mention that this analysis may underestimate the number of conserved mQTLs. Indeed the genetic component is conditional upon the environment as well as upon parameters such as tissue and plant age; therefore, the occurrence of non-conserved QTL is not per se indicative of non-genetic regulation [63].

**Figure 5:**
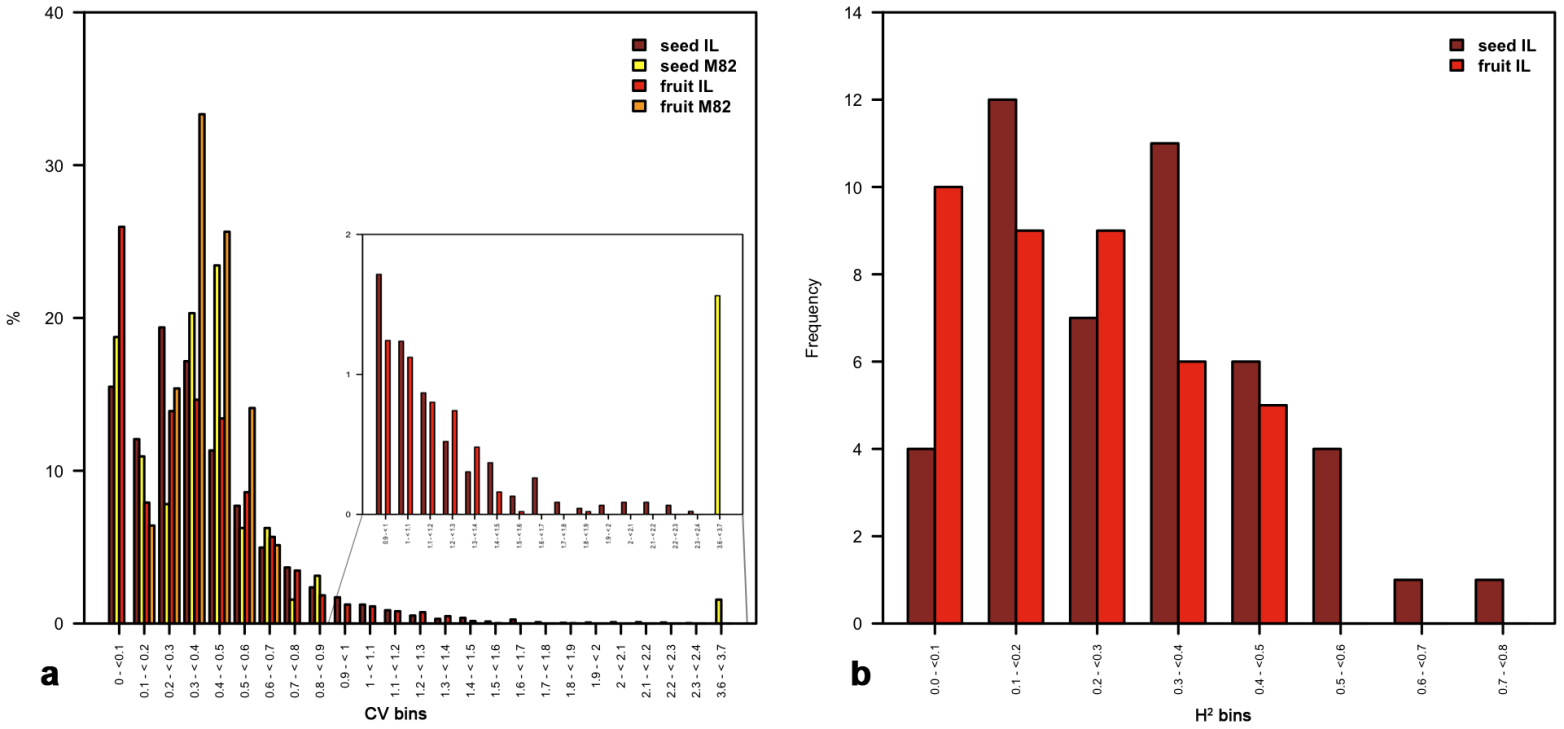
Tests of heritability as estimated on a tomato seed and fruit IL population. Coefficient of variation (CV) values were calculated by taking the ratio of the standard deviation over the mean for every metabolite individually for each IL and M82. Thereafter, the resulting CV values were divided into 40 bins of incrementing intervals of 0.1, for which the relative frequencies in the wild type and the ILs were estimated in both the fruit and in the seed (panel a). Broad-sense heritability (H^2^) values were calculated for all metabolites on the background of the seed and fruit IL population across two and three seasons, respectively. Values of H^2^ were divided into bins of 0.1 intervals. Bar graphs represent the total number for each respective bin on the background of the seed and fruit population (panel b). For exact estimation of H^2^ values see Methods and Materials.

In an effort to understand how phenotypic plasticity is affected by chromosomal substitutions (ILs) in comparison to M82, we calculated the coefficient of variation (CV) on the background of the seed and of the fruit (Figure 5a). The CV allows insights into the effects of genotypic and environmental variability on phenotypic plasticity [64]–[66]. In the present analysis, the CV of each (metabolic) trait was calculated for each individual IL; thus, the higher the CV, the greater the phenotypic plasticity of a particular genotype for a particular trait in response to the environment, i.e. the block design of the experimental setup [2]. For both the seed and the fruit, the CV explains 81% of phenotypic plasticity within the first six bins in the background of the wild-type M82, but only 75% in the background of the ILs. For the M82 seed data the first ten bins include 98% of the CV, while the same percentage of CV is covered for the ILs seed data in 13 bins. The skewing of the frequency distribution of the CV of individual ILs toward higher values suggests greater phenotypic plasticity in the introgressed background than in that of M82. Furthermore, the occurrence of a small portion (∼2%) of CV values greater than 1.6 in the seed background indicates tissue-specific phenotypic plasticity of certain traits. Moreover, the maximum CV value in the background of the fruit was 1.86, while in the seed the maximum CV was 3.67 (almost twofold higher). Interestingly, CV values of single ILs were higher than the arbitrary 0.9 in the background of the fruit, e.g., IL9-2 exceeded the CV value of 0.9 28 times and IL12-1-1 exceeded this value 20 times (results not shown). Highly scoring individual ILs in either the fruit or the seed background suggests a phenotypic plasticity induced by specific chromosomal substitutions.

To investigate the basis of heritability of metabolite abundance in the fruit and the seed separately, we estimated the broad-sense heritability (H^2^) for each trait (metabolite) across ILs and M82 in all three fruit datasets [2], [35] and in the two seed datasets. H^2^ estimates are divided into 10 bins of 0.1 intervals for which the frequencies of metabolites for the fruit and seed dataset are given (Figure 5b). H^2^ values approaching 1 suggest an increasingly unperturbed genotype-to-phenotype link. The H^2^ bar plot reveals a positive skewness in the distribution pattern of the fruit values, while seed values display a near normal distribution. Intriguingly, none of the H^2^ values in the background of the fruit population exceeded 0.5, whereas six occurrences (traits) exceeding 0.5 were scored in the background of the seed population, namely, hexadecenoate, maltotriose, phenylalanine, sorbitol, tyramine, and tyrosine. Taken together, the patterns observed suggest greater broad-sense heritability of metabolic abundance in the seed than in the fruit. This conclusion does not contradict the greater phenotypic plasticity shown earlier in specific ILs, as higher CVs characterized both seed and fruit individual ILs, which suggests enhanced phenotypic plasticity of metabolite traits induced by a small number of exotic introgressed segments.

While seed and fruit samples were harvested in parallel, the possibility that the developmental processes of the two organs can affect metabolite abundance differently cannot be excluded. Moreover, phenotypic polymorphisms in the two tissues might arise from genotype×developmental interactions, such as tissue-specific promoters that cause a gene to be expressed in only one or the other tissue.

### Candidate gene identification in mQTL of interest

In an attempt to identify candidate genes potentially associated with identified mQTL of the seed (Figure 3), all marker genes associated with each introgressed segment were identified via the SOL Genome Network of the tomato genome (http://solgenomics.net/ - for approach see section of Materials and Methods). Next, the functionality of the identified genes was inferred using information on the respective orthologs in the *A. thaliana* genome. Finally, in an effort to find co-responding metabolic genes that could further explain metabolite patterns of change, the SeedNet database (http://vseednet.nottingham.ac.uk; [67]) and the Seed Co-Prediciton Network SCoPNET database (http://vseednet.nottingham.ac.uk; [68]) were queried.

By applying the described approach, a number of potential candidate genes were identified, putatively regulating altered metabolic processes in the seed, as follows:

Amino acid metabolism: Co-localized QTL characterized by quantitative changes in the Arg/ornithine content for season I were shown to be shared for ornithine in season II on IL 2-4. In this segment, gene *At4g21120* was identified; this gene encodes for a member of the cationic amino acid transporter subfamily and is involved in Arg import. Associated with Arg/ornithine metabolism, the identified marker gene itself is most probably responsible for the altered levels detected for Arg and ornithine.

Glycolysis: Although significant changes in sugars in season I did not reoccur in season II, ILs 4-1, 4-3, and 4-3-2 still displayed intriguing features. Characterized by highly overlapping segments, these three ILs had increased levels of fructose and glucose together with a decreased content of sucrose, suggesting a QTL embedding genes with a significant impact on sucrose metabolism. For the introgressed segments associated with sucrose, namely, 4-1 and 4-3, no gene directly involved in sucrose metabolism was found. However, on IL 4-3 ortholog *At5g10920* was detected; this gene is involved in the Arg biosynthetic process [TAIR database] and is correlated by SeedNet to gene *At5g48300*, which encodes for a glucose-1-phosphate adenylyltransferase (Table S2). In addition, on IL 4-3-2 in chromosome 4, we identified ortholog *At5g63840*, which is involved in cellulose biosynthetic processes with a glucosidase and a hydrolase. Here, too, in agreement with our findings, SCoPNET co-predicted a strong association with aforementioned gene *At5g48300* (Table S3).

By following the same approach, we also identified candidate genes putatively associated with the pattern of change of co-localized fumarate/malate and glucose/fructose (Figure S2) in ILs 1-1-3, 4-4, and 5-4 (for IL 5-4 see Text S1). In these cases, we identified *Arabidopsis* orthologs exhibiting significant correlations to genes found in the introgressed segment and associated with the co-localized mQTLs. That said, on segment IL 1-1-3 we identified an aspartate semialdeyhde dehydrogenase, *At1g14810* [TAIR database], shown to be correlated to a fructose biphosphate aldolase (*r* = 0.81) involved in the catalysis of fructose-6-phosphate, and concomitantly correlated to malate dehydrogenase (*r* = 0.84), a key enzyme in the TCA cycle, as mentioned earlier (Table S4). On IL 4-4, ortholog *At1g20575* involved in D-ribose catabolism [TAIR database] was found to co-predict on SCoPNET to pyruvate kinase, an important enzyme in glycolysis (Table S5) and concomitantly to succinate dehydrogenase of the TCA cycle and mitochondrial electron transport chain on SeedNet (Table S6).

It should be noted that all genes as mentioned here have been identified by marker gene investigation of the tomato genome. Their functionality in association to the mQTL is to some extent speculative and remains to be tested. Moreover, a metabolic gene may not necessarily represent the regulating gene of metabolite content, and a significant amount of non-linearity between the level of expression of a “metabolic” gene and the content of the respective metabolite has been shown in tomato and Arabidopsis [69], [70].

Potential single-nucleotide polymorphisms (SNPs) or frame shifts within the sequences of the open-reading frame (ORF) and promoter regions of several candidate genes of the alleles were analyzed (Text S2). DNA sequences of *S. lycopersicum* alleles were obtained by BLAST searches of predicted cDNA sequences in the NCBI (http://www.ncbi.nlm.nih.gov/) and SOL Genomics Network (http://solgenomics.net/) Website, while those of *S. pennellii* were obtained by sequencing PCR products by using primers whose design was based on the sequence of *S. lycopersicum* genes (Text S2). The allelic sequences were then aligned and compared. The alleles of *SGN-U261955*, an Arg/ornithine-metabolism-related gene and ortholog of *AAT1* (*At4g21120*; [71]), revealed a 178-bp difference in the background of total lengths: *S. lycopersicum* = 4697 bp and *S. pennellii* = 4691 bp (Text S2). Moreover, a comparison of genetic polymorphism on the promoter region revealed a 178-bp of gap in the *S. lycopersicum* promoter sequence. Two genes that were predicted to be associated with glycolysis, *SGN-U242840* (putative argininosuccinate-lyase, orthologous gene of *At5g10920*) and *SGN-U217186* (putative glucosidase, orthologous gene of *At5g63840*), revealed several polymorphisms in their coding regions. The *S. lycopersicum* allele of *SGN-U242840* was characterized by an 84-bp gene deletion in its ORF (see Text S2). In contrast, no allelic differences were seen in the ORF sequence of *SGN-U217816*. However, many allelic differences were found in the promoter region, where even more gaps (107/1000 bp) were observed. Thus, the large differences in the promoter region, but high similarity of the coding region, hints at different modes of regulation of gene expression, causing the changes in metabolic levels, rather than a diverse functionality of the gene product. However, further experimentation will be needed to confirm this hypothesis, including testing the possibility of tissue-specific expression of candidate genes, which might lead to the differences shown in the seed and fruit metabolism of the same IL.

### Correlation analysis reveals a highly concerted interplay of amino acids in the seed metabolic network, as affected by genetic introgression

In an attempt to evaluate co-regulation of groups of metabolites affected by genetic alteration, we conducted a series of correlation analyses on the metabolite profiles of the IL population for seasons I and II. A pair-wise correlation among all metabolites, applying the Pearson's product-moment correlation is visualized as a heat map in Figures S3 and S4. In this visualization, the triangular constellation of amino acids in the center of the figure indicates a high correlation between the pattern of change in the contents of most amino acids across the population. An averaged absolute *r*-value of 0.53 was calculated for all pair-wise correlations between amino acids, ranging between 0.98 for Val and Ile and −0.03 for β-Ala and Met. Amino acids showed mainly positive correlations, with a ratio of 189 positive correlations to one negative correlation. Six amino acids, Val, Ile, Gly, Pro, Ser, and Thr, exhibited notably high correlations, with an average *r*-value of 0.87. The high degree of correlation of these six amino acids can be explained by their co-localization onto the same QTL (Figure 3). Other significantly high correlations among the amino acids were found between GABA and β-Ala, and between Asn, ornithine, Lys and Tyr. In contrast, Asp, Glu and Met exhibited a noticeably low degree of co-ordination among the amino acids. Correlation analysis revealed that amino acids were also strongly positively correlated to carboxylic acids, polyhydroxy acids, N compounds, phosphates, and polyols, including compounds such as urea, nicotinate, phosphoric acid, glycerol-3-phosphate, glycerol, erythritol, sorbitol, and inositol. The same amino acids also showed strong correlations with pyruvate, a key intersect of various metabolic pathways, with an average *r*-value of 0.48, and a maximum *r*-value 0.63 between Gly and pyruvate. Furthermore, significant correlations were detected between the following amino acids, Asn, Asp, Arg, Lys, Tyr, β-Ala, GABA, ornithine and 4-OH-benzoate of the shikimate pathway and citrate.

Generally, there were only a few negative correlations, and these involved mainly sugars: 52.9% of the total 620 correlations associated with sugars were negative. The strongest negative correlation of −0.55 for the complete dataset was recorded for the fatty acid nonanoate and for sucrose. The TCA cycle intermediates, fumarate and malate, and salicylate also correlated negatively to the monosaccharides fructose and glucose and to the disaccharide sucrose.

Using the same approach, we also computed all pairwise correlations based on both seasons, which will be discussed in the context of network analysis.

### Correlation analysis between morphological traits and seed metabolites displays negative inter-organ correlation and reveals contrasting trends

**Figure 6:**
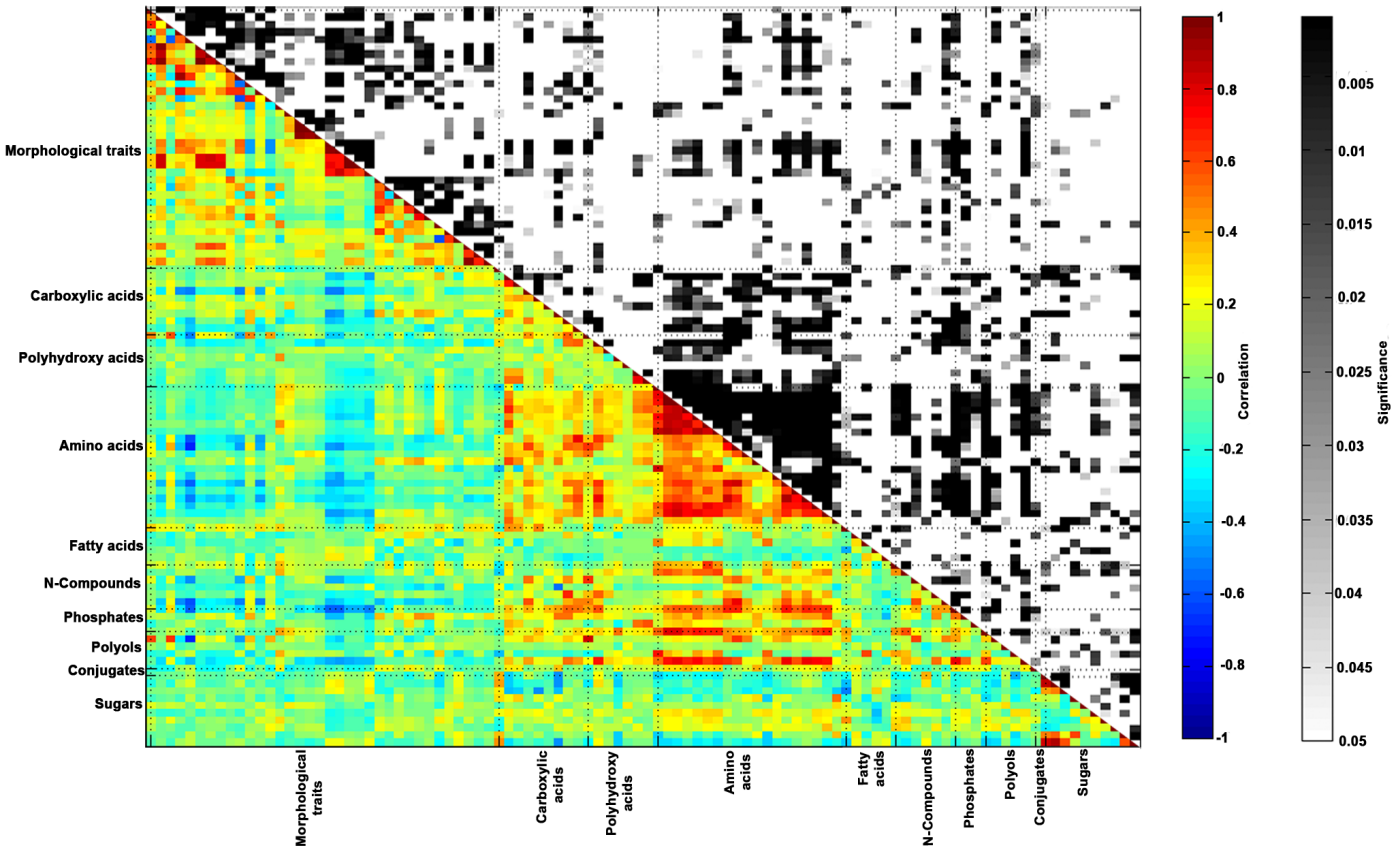
Morphological traits—metabolite correlation/significance. Correlation between metabolic data as analyzed on dry IL seeds of harvest season I in Akko, Israel and the ILs' morphological traits. The Pearson product-moment correlation was used to calculate all pairwise correlations between morphological traits and metabolites heading the rows and morphological traits and metabolites heading the columns. In the colored area, rectangles represent *r* values resulting from Pearson correlation coefficient computation (see correlation color key). In the black and white area, rectangles represent p-values respective to Pearson correlation coefficient (see Significance color key). *Z*-score transformation was employed to enable correlation computation. X and Y-axes are categorized into morphological traits and metabolites, grouped by compound classes.

Next, we analyzed our metabolite dataset against traits of the mother plant, the fruit and the seed for associations [2], [35]. In total, 35 morphological traits were compared to the 64 seed metabolite compositions (the entire dataset is available on http://phnserver.phenome-networks.com). Following z-score transformation, we performed correlation analysis employing the Pearson's product-moment correlation. The results of this analysis are presented in Figure 6 and in greater detail in Figure S4.

The analysis produced a relatively high number of negative correlations: in total, 61.2% (1392 out of 2275 correlations) of all associations were negative. The correlation between GABA and the harvest index (HI) displayed the highest negative value of −0.71 in the association matrix (p<0.005), indicating that the content of GABA in the seed is highly associated with the growth of the mother plant. Furthermore, significant – and mainly negative – associations were found primarily for morphological traits of the seed and of the fruit (Figure S4). Specific metabolites were shown to correlate with morphological traits—among the organic acids, glycerate, 4-OH-benzoate, benzoate, pyruvate, citrate, salicylate, fumarate, malate, and particularly succinate; among the amino acids, GABA, Asp, Met, Gln, Phe, Asn, ornithine, Arg, Lys, and Thr; and among N compounds, uracil, putrescine, tyramine, and adenine. The amino acids Lys, Arg, Asn, Phe, and ornithine concertedly correlated (negatively) to HI and to fruit weight, and Tyr, Lys, Arg, Asn, Phe, Glu, Met, Asp, ornithine, and GABA correlated (negatively) to the ratios – seed weight to fruit and seed number to fruit (Figure S4). A strong positive correlation was found between glycerate and several morphological traits (plant weight, total yield, and BRIX, Figure S4). The traits plant weight and BRIX also correlated positively (∼0.7) with the content of polyol erythritol in the seed.

### Network analysis highlights the higher intragenotypic correlation of seed metabolism than fruit metabolism

To further investigate the degree of correlation between metabolites in the seed, we applied network analysis and visualized the metabolite-to-metabolite association via a graph of nodes and edges. In addition, the metabolites measured in the pericarp by Schauer et al. [2] were integrated into a comparative analysis in an attempt to unfold inter-organ correlation to introgression and common mechanisms of metabolic regulation. In the generated networks, each node represents a metabolite, and an edge joining two nodes represents the association between two metabolites, in our case a correlation (either positive or negative) across the population of introgression lines. Results for all networks as described here are presented in Table S7. The resulting fully annotated networks are presented in Figures S5 and S6, where metabolites are grouped and color-coded according to their compound classes. The graphic outcome and network parameters revealed that the interconnectivity of the seed metabolite network is significantly greater than that of the fruit metabolite network: All measured network parameters, particularly network connectivity and density, reflect a tighter interconnectivity of the seed metabolite network over the fruit metabolite network, i.e. a twofold higher connectivity characterized the seed network (Table S7). The fruit network included 83 nodes, interlinked by 383 edges. At the *r* threshold of 0.3, the fruit network showed a lower degree of connectivity (9.23) than the seed network (Table S7). The resulting ratio of edges to nodes for the fruit network was 4.61, as opposed to 10.95 for the seed network. It should be noted that the sustainability of the fruit metabolite network could be achieved only at an *r*-value threshold of 0.3, which implies weaker associations between metabolites. As opposed to the significant divergence in the other network parameters, the cluster coefficient of the seed network was similar to that of the fruit network. The network analysis suggests a generally higher independence of groups of metabolites in the fruit network as compared to the dependence of all metabolites on the amino acid module in the seed network. To test whether the differences in network properties are due to inherent differences in the variance of the two datasets, we conducted the following test. The average variance of the entire seed dataset for season I was calculated and used as reference value to rank the computed variance of each IL of the fruit dataset for season I (Table 1). Next, subsets of the identified fruit ILs were used to construct subset networks. Applying F-value statistics, we showed that none of the chosen fruit ILs was significantly different from the average seed variance (Table S8). The initial subset was composed of 15 ILs incremented iteratively to a total of 25 ILs, listed in Table 1. Each subset was subjected to network analysis computing the following network measures: density, degree, clustering coefficient, and diameter. To determine the statistical difference to the respective network measures in the seed metabolite network, we permuted the subset of ILs 1,000 times, calculating all the above-mentioned measures at each permutation. The results of the permutation tests were used to estimate p-values of the observed differences between the subsets and initial seed network measures (Table 1). As shown in Table 1, all fruit IL subset networks exhibited lower values for the network measures density, degree, and clustering coefficient than the seed network. It should be noted that the findings of the network analysis show trends of the calculated network properties. For example, while degree and density show increasing trends with incremented number of ILs (from 0.032 to 0.15 for 15 to 25, 50, and 76 lines), the clustering coefficient shows a decreasing trend (from 0.45 to 0.38 for 15 to 76 lines). However, all properties display mild fluctuations, as exemplified for density and degree properties between subsets with 17 and 19 ILs. This observation can be attributed to the addition of a single IL and its impact on the topology of the network, as each IL modifies the outcome of the correlation coefficients and their respective p-values. Diameter, on the other hand, exhibits strong fluctuations, with a stagnating pattern towards an increasing number of ILs. This finding was not surprising considering that graph (network) theory postulates on diameter of a network: giving the length of the longest from all pairwise shortest paths, the diameter of a network is an ordered property (max = longest) expected on average to be strongly affected by addition of few, strategically positioned, edges. In contrast, the average degree and the density – depending only on the number of edges, but not their position in the network with respect to the other edges – are expected to exhibit much smaller fluctuations, which is in keeping with the results from our empirical study.

**Table 1:** Network measures estimated on fruit and seed datasets.

Network	Density		Degree		Clustering coefficient		Diameter	
	value	p-value^1^	value	p-value^1^	value	p-value^1^	value	p-value^1^
**76 Seed IL network**	**0.35**	**-**	**21.87**	**-**	**0.61**	**-**	**6**	**-**
15 Fruit IL network	0.032	0.001	2.49	0.001	0.45	0.001	9	1.000
16 Fruit IL network	0.036	0.001	2.77	0.001	0.44	0.001	12	1.000
17 Fruit IL network	0.040	0.001	3.05	0.001	0.43	0.001	12	1.000
18 Fruit IL network	0.043	0.001	3.28	0.001	0.47	0.001	13	1.000
19 Fruit IL network	0.041	0.001	3.18	0.001	0.45	0.001	11	1.000
20 Fruit IL network	0.043	0.001	3.31	0.001	0.43	0.001	10	1.000
21 Fruit IL network	0.047	0.001	3.59	0.001	0.49	0.001	11	1.000
22 Fruit IL network	0.047	0.001	3.62	0.001	0.47	0.001	9	1.000
23 Fruit IL network	0.044	0.001	3.72	0.001	0.43	0.001	11	1.000
24 Fruit IL network	0.044	0.001	3.77	0.001	0.43	0.001	10	1.000
25 Fruit IL network	0.047	0.001	3.77	0.001	0.42	0.001	12	1.000
50 Fruit IL network	0.078	0.001	5.97	0.001	0.47	0.001	11	1.000
76 Fruit IL network	0.15	0.001	8.18	0.001	0.38	0.001	7	1.000

1 p value based on 1,000 permutations.

Individual ILs were ranked according to increasing difference in variance of the fruit dataset compared to the average variance in the seed dataset (Table S8). Network properties were calculated from a network reconstructed by using the data from the ordered list of ILs. For instance, for n = 25, the 25 ILs from Table S8 were used in creation of the correlation network associated to the data from only these n = 25 ILs. Subsets comprising the first 15 to 25, 50 and 76 of the fruit ILs ranked in non-decreasing order with respect to their variance were used to construct correlation-based networks (*r*≥0.3, p≤0.01). Four network properties were calculated for each subset-based fruit network: density, degree, clustering coefficient, and diameter (value). Values represent the estimates of the respective network measures for each subset of fruit ILs. By performing the classical permutation test with 1,000 repetitions, the statistical significance of the differences in measures between the subset-based fruit networks and the seed network (first data row in the table) were measured. In each permutation, the order of each metabolite within the subset was randomized, and the newly ordered dataset was subjected to correlation analysis and network measures estimation. The difference between newly generated network property values in the seed and the fruit, upon randomization, were tested to check whether their value is at most that of the difference for the original networks. Subsequently, the total number of occurrences meeting this criterion formed the basis for the empirical p-value estimation. With the exception of the network diameter, density, degree, and clustering coefficient of the fruit IL subset networks are significantly different from the corresponding measures in the seed network.

The same test was performed iteratively on subsets of ranked seed ILs, based on the former calculated average variance of the seed dataset to exclude the possibility that the differences in network measures are a consequence of the reduced number of ILs used in the test. All network measures for the seed IL subsets were significantly different from those for the fruit IL subsets (data not shown). Taken together, these results indicate that the observed differences in network topology are indeed a consequence of differential metabolic regulation in response to genetic alteration rather than the result of differences in variance between the seed and fruit. That said, the discrepancies in the variance of the two datasets – possibly originating in different heritabilities – of the two tissues, their different ploidy levels, the tissue-specific regulation of gene expression, and differences in the accuracy of defining the developmental stage of the two tissues amplify the differences measured between the two networks.

### Amino acids function as a structural hub within the seed metabolic network

Modules are defined as metabolites affiliated with their compound classes and highly interconnected. Within the seed network, the most apparent module is of amino acids (illustrated as green nodes in Figure S5). The amino acids module is characterized by a relatively high interconnectivity: 479 of the total of 689 edges are directly connected to one amino acid or to a number of amino acids. Similarly to the seed network, a tightly intra-linked group of amino acids is evident in the fruit network, where it accounts for 213 of the 383 edges, i.e., 56%, as compared to the 67% in the seed. In contrast to the seed network, the fruit network is characterized by a rigid sugar module. The sugar module incorporates 11 nodes and 86 edges in total (23%), linking it directly to all other compound classes, but particularly to carboxylic acids and polyols. For instance, sugars and carboxylic acids (23 nodes) are interlinked by 11 edges, and sugars and polyols (17 nodes) are connected by 20 edges. Another apparent difference between the two networks is the absence of a fatty acid module in the fruit network.

**Figure 7:**
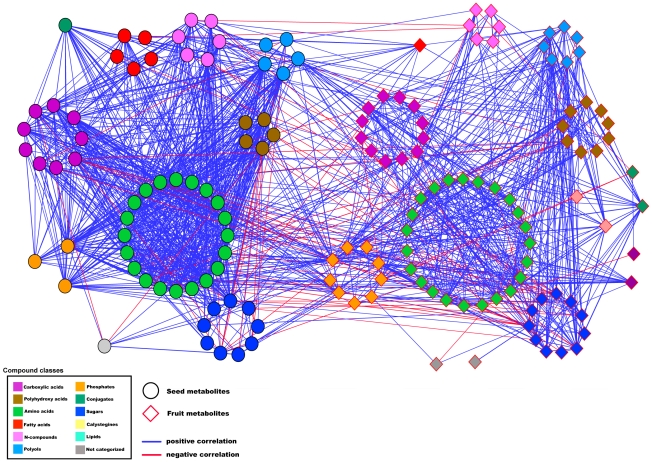
Seed-fruit metabolite network. Network visualization of metabolites as analyzed on dry IL seed and fruit metabolites of harvest season I in Akko, Israel. Metabolites are represented as nodes, and their relations, as edges. The Pearson product-moment correlation was employed to compute all pairwise correlations between metabolites across the entire set of ILs. Only significant correlations are depicted. A significance level of ≤0.01 and an *r*-value of ≥0.3 were considered to be significant. Seed metabolites are depicted as circular black-bordered nodes, fruit metabolites are depicted as diamond-shaped red-bordered nodes. Metabolites are color coded and clustered according to their compound classes. The two tissue sub-networks are separated spatially into the left region (seed network) and right region (fruit network). Positive correlations are denoted as blue edges, and negative correlations are denoted as red edges. Computations of the correlations were conducted under the R environment. Cytoscape was used to generate the graphical output of the networks.

To test the occurrence of associations between the seed and the fruit networks, a combinatorial fruit-seed metabolite network was constructed (Figure 7). The results of network parameter calculations are presented in Table S7. The combinatorial network revealed a large number of negative linkages between the fruit and the seed modules—37.5% of 269 edges connecting the two tissues. It should be noted that fruits and seeds were collected in parallel and thus the associations may indeed reflect true metabolic interactions between the two organs. In particular, sugar metabolites of the fruit network demonstrated negative correlations to seed metabolites, notably to amino acids, whereas fruit phosphate maintained 25 positive correlations to seed metabolites, mostly to seed amino acids. In the seed-fruit correlation analysis, negative correlations between sugars were evident: seed sucrose correlated negatively to almost all fruit sugars; a similar pattern was observed for raffinose; fruit glucose and fruit fructose correlated negatively to most compound classes of the seed, particularly to amino acids and carboxylic acids, whereas seed fructose and seed glucose did not display negative correlations to fruit sugars. Octadecanoate, the only fatty acid apparent in the fruit network, connected with more edges to the seed network, particularly to organic acids, amino acids, and phosphates, than to the fruit network (14∶2 connection). Notably, the module of the fruit amino acids did not show connections to the seed network. We confirmed the general observations of the network topology and modular structure by employing network analysis on season II and by generating integrated networks for both seasons. The results are summarized in Figures S5, S6, and S7 and Text S3.

### The NeMo algorithm identifies structurally functional clusters and further highlights the amino acids in the seed as core to its structure

**Figure 8:**
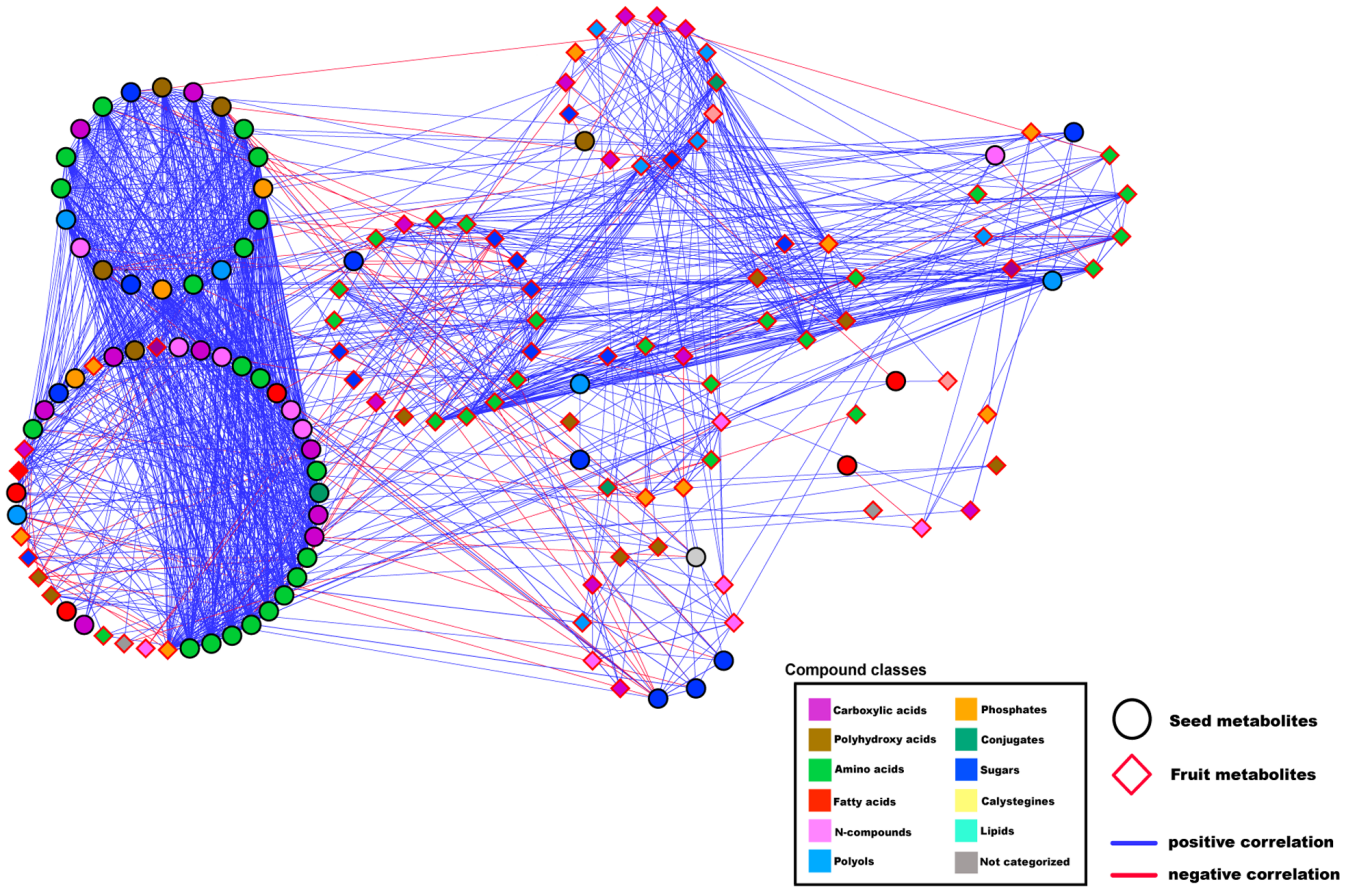
Degree of connectivity cluster network. Network visualization of metabolites as analyzed on dry seed and the fruit of IL season I harvest in Akko, Israel. Metabolites are presented as nodes, and their relations, as edges. The Pearson product-moment correlation was employed to compute all pairwise correlations across the entire set of ILs. Only significant correlations are shown. Clusters of high connectivity of metabolites were generated on the basis of the network presented in Figure 7 by applying the clustering algorithm supplied by the NeMo for Cytoscape plug-in. Metabolites were clustered together on the basis of the degree of connectivity to adjacent metabolites. The two main clusters (seed-fruit) were verified by computing the modularity value Q by using the fast greedy community algorithm under the R environment. Seed metabolites are depicted as circular black-bordered nodes, and fruit metabolites are depicted as diamond-shaped red-bordered nodes. Positive correlations are denoted as blue edges, and negative correlations, as red edges. Computations of the correlations were conducted under the R environment. Cytoscape was used to generate the graphical output of network.

We next used the combined network to generate clusters of metabolites displaying significantly high degrees of connectivity between nodes. When applied to the integrated data matrix, the NeMo algorithm [72] generated two separate main clusters of seed and fruit metabolites (Figure 8). Within the seed metabolite cluster, we detected two highly interconnected sub-clusters, which could, in fact, be merged into a single cluster. We confirmed the validity of the NeMo algorithm (see Materials and Methods) by permuting the metabolite class membership and calculating the resulting modularity. Furthermore, amino acids were embedded and spread equally between the seed sub-clusters and were highly connected to all other seed metabolites. In contrast to fruit amino acids, which were scattered throughout all fruit clusters, the seed amino acids β-Ala, Val, Ser, Arg, Thr, Pro, Ile, and Asp, appeared to be acting as a single group, maintaining a high number of connections to the second seed metabolite cluster, which included Gly, the sixth of the above-mentioned amino acids. The centrality of the amino acid module in the seed was evident across the two seasons (Figure S8). Cluster analysis on the intersect graph showed the grouping of Thr, Ile, Val, Pro, and Ser into one cluster, whereas Gly was embedded in a different cluster. The cluster analysis of the seed season II network showed that Ile, Val, Pro, and Ser were grouped into one cluster, whereas Thr and Gly were incorporated into a different cluster.

**Figure 9:**
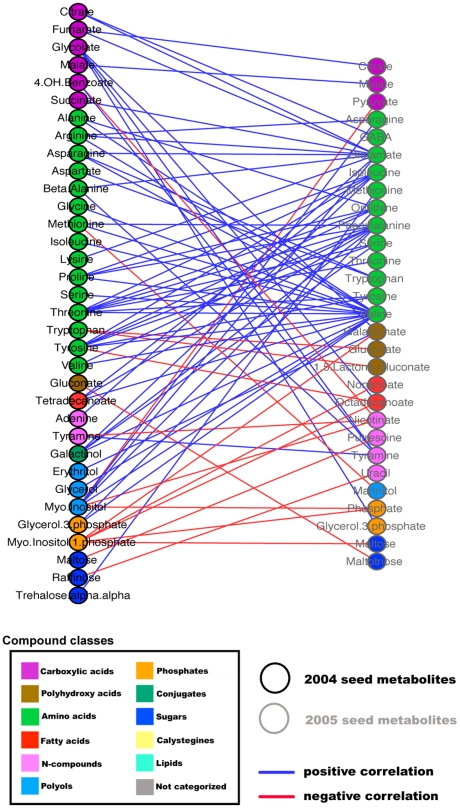
Bipartite cross-seasonal correlation network. Bipartite cross-seasonal network visualization of metabolites as analyzed on dry IL seeds of harvest seasons I and II in Akko, Israel. Metabolites were correlated employing Pearson's correlation coefficient among seasons and ordered according to their compound classes. Nodes represent metabolites of harvest seasons I and II, respectively. Edges represent significant correlations across seasons among metabolites. Blue edges depict positive correlation, and red edges, negative correlations. A significance level of ≤0.01 and an *r*-value of ≥0.3 were considered to be significant. Computations of the correlations were conducted under the R environment. Cytoscape was used to generate the graphical output of the network.

In an effort to assess the observed correlations for seasons I and II and to understand the nature of cross-seasonal changes (environmental or genetic), we correlated metabolites for the seed across the two seasons, applying the same parametric constraints as before. The results are presented in Figure 9 as a bipartite network graph. The amino acid modules for both seasons maintained an extensive number of cross-seasonal links. In total, the two amino acid modules accounted for 27 of the 63 nodes, 67 of the 82 edges, and 35 edges of purely positive correlations to each other. Here, too, we observed the tight connectivity of the above-mentioned amino acids across the seasons, i.e. Ile of season II was linked to Pro, Thr, and Tyr of season I; Thr of season I was connected to Ile, Phe, Ser, Thr, Tyr, Val, and ornithine of season II; and Val of season II was connected to 12 of the 15 amino acids of season I. Another noticeable example of cross-seasonal linkage was malate of season I with malate of season II. During the QTL mapping, we detected two malate QTL in ILs 1-2 and 1-3. The cross-seasonal correlation of malate appears to be of a genetic nature and thus confirms the validity of the detected QTL and further hints at a genetic basis in the co-regulation of the carboxylic acids and sugars.

Lastly, correlation analysis was used to compare changes in metabolite abundance in seeds and fruit. All three available fruit networks (season I, III, and IV) [2] and seed networks (season I and II) were analyzed for conserved correlations, always applying the same constraining parameters as already described (*r*≥0.3, p≤0.01). In addition, all six possible cross–tissue networks were constructed, where each combinatorial network was compared for re-occurring correlations to each network. The results are given in Table S9. Two main observations were made via these analyses: i) the relative number of conserved correlations across two seed seasons always exceeded the number of conserved correlations among all the possible combinations between three fruit seasons, and ii) there were no conserved correlations between the combinatorial fruit-seed networks across all seasons, thus hinting at a conditional environmental factor in fruit-seed interaction.

## Discussion

In this study, the genetic basis of natural variability in seed primary metabolism and its response to perturbation were investigated. To accomplish this aim, we employed metabolite profiling on seeds from a collection of tomato introgression lines (ILs) containing segmental substitutions of the wild species chromosome in the genetic background of the M82 cultivar. Next, we integrated data from previous metabolic profiling studies on fruit pericarp together with yield-related parameters and plant morphological traits [2], [7]. The integrated heterogeneous data matrices were investigated by correlation network analysis, which allowed us to comparatively study the structure and topology of the seeds and of the fruit metabolic network.

A number of putative mQTL were identified, in particular, for amino acids and organic acids, which – in contrast to most sugars – were confirmed in two consecutive seasons by pairwise two-way ANOVA.

An investigation of the genetic basis of metabolite polymorphism, by factorial analysis, indicated that genetic factors significantly affected the level of every metabolite. The CV confirmed these findings, showing a general promoting effect of the genetic introgression on the phenotypic plasticity of the metabolite traits. In addition, distinct chromosomal segments led to higher phenotypic plasticity in the seed in ∼2% of the traits. Nonetheless, in specific comparisons of tissue-dependent broad-sense heritability, the greater occurrences of H^2^ values approaching 1 in the analysis of the seed dataset indicates increased heritability of metabolic traits in the seed compared to the fruit, while the high frequency of relatively low H^2^ values in the background of the fruit suggests metabolic polymorphism more affected by environmental factors.

Correlation-based network analysis indicated that the seed metabolic network is inherently more coordinated than the fruit network, as is reflected in a higher number of correlations (the vast majority of which being positive correlations), leading to higher density and higher cluster coefficients. In other words, a more conserved link between genotype and trait, associated with highly synchronized patterns of change in metabolite abundance, reflects the tight regulation of metabolic processes in the seed, which is probably aimed at maintaining the metabolic balance needed for storage reserve allocation, C-N partitioning, and reorganization of metabolism at the onset of germination. We hypothesize that the metabolism of the fruit pericarp, if not acting concertedly as it should, poses a lesser danger to species survival than irregularities in seed metabolism.

The findings of significantly coordinated regulation of metabolic processes in seeds of different species, e.g., strawberry, *Arabidopsis* and pea [73]–[76], and of defective germination in seeds with unbalanced metabolism [77]–[81] imply that during evolution of seed-plants plants have also been selected on the basis of their efficient regulation of seed metabolism. Seed metabolism acts concertedly both during development and when major perturbations are introduced [73], [74], [76]. Coordinated activity of metabolic processes may be due to the presence of transcription factors or to allosteric and/or epistatic interactions [82], [83]. Via sequence analysis of a subset of candidate genes and the corresponding promoter regions, we concluded that certain metabolic polymorphisms result either from differential functionality of the gene product or from differential gene regulation. Moreover, variations at the metabolite level might originate from a developmental regulation of gene expression. The relevance of developmental specificity, i.e., tissue-specific promoters, and variation in the ploidy levels of the fruit and the seed in relation to metabolic regulation should be further investigated. Last, we cannot exclude different degrees of precision in defining the developmental stage of the two tissues between different plants.

Statistical analysis indicated significant effects of the season and of organ development on metabolite abundance. Thus, it is not inconceivable that the genetic network controlling the tomato seed metabolism is dependent, at least in part, on both the environment and on tissue and plant development [60], [84], [85]. The high fluctuations of the seed sugar levels between seasons I and II may be in response to differences in environmental conditions. According to the Israel Meteorological Service (IMS – ims.gov.il), temperatures recorded for August of season I (2004) were below average. Season II (2005), on the other hand, showed temperatures above average of up to +1°C during the daytime and up to +2.5°C at night in plane plateaus, such as the Western Galilee Experimental Station, Akko, Israel, where the plants were grown. As shown by the 3-factor modeling, the seasonal factor has a significant impact on the variance of the dataset. Schauer et al. [35] have previously shown that inter-seasonal comparisons greatly reduce the number of QTL; they recorded only 43 conserved QTL across three seasons of initially 889 detected significant changes [2].

The high degree of negative correlations between seed metabolites and morphological traits indicates an opposite pattern of change of distinct morphological traits with respect to the specific metabolite quantities across the population. Cartographic network analyses revealed that, in particular, the contents of seed metabolites were negatively correlated with the harvest index (HI). These results are in keeping with earlier findings showing negative associations between fruit metabolites and the HI [2], [7]. While correlation analysis alone cannot reveal the direction of cause and effect, it is likely that the supply of metabolites and/or the tradeoff between organs during plant growth determines the level of metabolites in each respective organ. Negative correlations between seed metabolites and HI were observed mainly for amino acids and N compounds, which also shared a correlation to the seed-weight-to-fruit ratio and seed-number-to-fruit ratio. Interestingly, a similar observation was made for the amino acid composition of the pericarp [2], and subsequent studies on a different introgression line population confirmed that this correlation held under different cultivation practices that modified the whole plant sink-source balance [58]. Taken together, these lines of evidence show a direct link between N partitioning and crop yield. That said, sugar levels in the seed do not seem to be affected by changes in morphological traits as opposed to the sugar levels in the fruit pericarp [2]. These pieces of evidence suggest that the delivery of photoassimilates, in particular C moieties, from the leaves to the seeds does not stand in relation to the delivery to the fruit pericarp, as recorded by Schauer et al. [2]. Generally, since the development of seeds is set as the terminal stage of plant development, it may be concluded that major global changes in metabolite levels in the seed are the result of variation in growth and resource allocation, rather than vice versa, suggesting competition for resources between vegetative growth and reproductive organ development. A marked interplay between fruit and seed was shown by applying network analysis. Short fatty acids, polyamines, putrescine, organic phosphates, and particularly sugars, were involved in correlations between the two organs (Figure 8). It is reasonable to suggest that in the regulation of carbon allocation via processes of sugar sensing and transport [86], a pyramidal interaction regulates pericarp sugar metabolism (on the basis of plant vegetative growth and HI), which, in turn, affects the seed precursors for storage reserves. Beyond metabolic linkages, it is clear that plants function as integrated systems, in which metabolic and developmental pathways draw on common resource pools and respond to changes in environmental energy and resource supplies [87].

With regard to the regulation of seed metabolic processes, network analysis revealed the existence of a tightly inter-regulated amino acid module, acting as the backbone to the network, in contrast to the independent amino acid sub-network of the fruit pericarp. The occurrence of a highly intra-connected amino acid module is also in keeping with results from analyses of an *A. thaliana* seed ethylmethane sulfonate (EMS) mutant population, which showed an unexpected concerted change in the content of 12 biosynthetically unrelated proteogenic free amino acids [88]. The inter-dependence of biosynthetically unrelated amino acids observed in our study concurred with that of biosynthetic related amino acids, such as Gly, Ser, Thr, Ile and Val, of which Thr, Gly, and Ile are directly associated with the Asp family [89], [90], Ser is closely related to Gly, and Val biosynthesis is initiated by Thr (KEGG pathway database - [91]–[93]). Amino acids closely related by a biochemical pathway exhibited even stronger correlations than the average in the amino acid module. The significant positive correlations between amino acids imply that ratios between amino acid levels within a seed “must” be maintained, and they reflect a highly regulated amino acid metabolism that includes both protein and non-protein amino acids (i.e. GABA), both aromatic and aliphatic, likely to occur at the post-transcriptional level in the regulation of N allocation. That said, we cannot rule out the possibility that integration of induced changes at the transcriptional level accounts for the intragenotypic correlation of amino acid metabolism. The vast number of highly significant associations between the amino acids and carbon metabolites in the seed is indicative of considerable crosstalk between C and N networks, as is exemplified by the correlation between pyruvate-nicotinate (niacin, precursor of NAD), on the one hand, and amino acids and glycolytic intermediates, on the other hand. Our results support previous suggestions of an extensively overlapping regulatory basis for central pathways in N and C metabolism [46], [94], [95].

Furthermore, network analysis suggests a possible important functional role for unbound metabolites in the dry seed [47] to ensure a balance of the downstream processes vital for germination, such as protein assembly and hormone biosynthesis; for instance, the alteration in Lys metabolism during seed maturation in *Arabidopsis* caused abnormal protein biochemical characteristics (solubility) and impaired germination [47], [76]. To date, the function of unbound metabolites in mature seeds has been widely disregarded. Dry mature seeds – for instance in *A. thaliana* – store mRNAs of more than half of all genes [96] that regulate the content and proportions of unbound pools of metabolites in the mature seeds. These lines of evidence suggest that the regulation of unbound metabolites during see maturation is vital for post-dispersal storage and germination.

To conclude, our study shows that metabolite profiling in combination with significant genetic variability can reveal important regulatory mechanisms in seed metabolism. Network analysis, coupled with tests of heritability and phenotypic plasticity, highlighted the inherent differences between seeds and fruit in the metabolic network structure and in the modes of inheritance and revealed a hierarchy of regulation between morphological yield-related and metabolite traits. Being applied on data from heterogeneous sources, correlation-based network analysis has proven successful—from the simple test of consistency of the measurements across seasons, through a comprehensive understanding of fruit-seed metabolite response to genetic alteration, to the identification of modules and metabolites with significant structural roles, which are worthy of further research. In particular, the analysis of the seed metabolic response to genetic alteration highlighted the relevance to keeping specific areas of metabolism balanced. As such, metabolic network analysis combined with genetic resources can lead to the development of significant supportive approaches in defining broader strategies for crop quality improvement.

## Materials and Methods

### Growth conditions

The metabolite data set was obtained from seeds of field-grown ILs from two seasons (2004 – season I and 2005 – season II) isolated from exactly the same plants as the fruit pericarp material described in ref. [2], [35]. The field trials were conducted at the Western Galilee Experimental Station in Akko, Israel. Plants were grown in a completely randomized design with one plant per m^2^. Seedlings were grown in greenhouses for 35–40 days and then transferred to the field. Fruit was harvested when 80 to 100% of the tomatoes were red [59]. The field was irrigated with 320 m^3^ of water per 1,000 m^2^ of field area throughout the season.

### Metabolite profiling by gas chromatography/mass spectrometry

Relative metabolite content was determined essentially as described in ref. [60] and [61] with modifications specific to tomato [97] and seed tissue [73].

### Data processing and statistics

Metabolite data generated by GC-MS comprised unique mass intensity values for each annotated compound. The raw data for each metabolite was normalized by dividing each value by the median of all tags in the corresponding data file generated from each chromatogram. In addition, to overcome biases due to separate injection periods, we ran a bulked extraction of M82 as reference throughout the injection sets. The response values were then normalized on the M82 reference sample. Descriptive statistics were calculated with R statistical software, Microsoft Excel 2004 for Mac, and MatLab 2008b version. For subsequent multivariate analyses, the data were log_10_ transformed. Solely for purposes of visualization, the data were also fold-transformed.

### QTL mapping

QTL mapping analyses were performed on the reference-based normalized raw data, followed by log_10_ transformation. To test whether metabolite quantities changed significantly across all ILs and the control M82, one-way ANOVA was performed for each metabolite. A permissive threshold level of ≤0.05 was chosen to test for significant changes. Subsequently, metabolites that were identified to be significantly altered were subjected to Dunnett's test, with M82 as the control. This analysis detected specific ILs with significant quantitative changes in metabolic levels and as such identified putative QTL. After the Bonferroni correction, our initial p-value of 0.05 resulted in a critical value of ≤0.006. Despite this highly restrictive criterion, we chose here to depict putative QTL with a critical value of ≤0.01 in the Dunnett's test heat map. Values illustrated by gradual color codes in the Dunnett's test heat map exclusively delimit significant changes, but additionally depict the average metabolic level for each identified IL after log transformation, as mentioned above. Pinkish areas indicate no significant alterations of metabolic quantities vis-à-vis control M82. Due to the differences in the datasets, the confidence intervals were calculated for the season II dataset. Only ILs generating a significant change for both seasons, following the strict adjusted p-value after the Bonferroni correction, were considered to be shared, leading to the identification of 46 shared QTL. The QTL heat map and hot spots were assembled with MatLab 2008b version and edited with Photoshop version 8.0 for Mac, also serving as the graphics editor for the QTL map. The bar graph figure displays ILs for significantly identified metabolites and the metabolic quantity in relation to control M82. Solely for the purpose of visualization, all values for metabolites were averaged and displayed as a fold changes with respect to M82. The bar graphs were generated in R, utilizing the default settings, and edited in Photoshop.

### Two- and three-way-factor ANOVA modeling

The three main factors with potential impact on the variance of the different datasets were chosen as follows: IL (representing the genetic background), tissue (seed and fruit), and season (I, II, III, IV). All ANOVA models were tested with log-transformed data with fixed factors, creating dummy variables for the season and the tissue factor. A significance threshold of p≤0.05 was chosen. For the 2-way ANOVA, seed seasons I and II were integrated into the analysis. Here, a pairwise ANOVA was run for every metabolite with corresponding ILs and controls for both seasons. For the 3-way ANOVA, a full-factorial model with all possible interactions and with all single and combined effects, was initially designed, including the fruit season I, III, and IV datasets and the seed season I dataset. In an iterative mode, the model was applied individually to all metabolites present in all datasets subjected to the analysis. Based on the outcomes, subsequent models were formulated. Also, models testing only for two-way interactions or two-way and single effects were designed to show the impact of single factors and combined factors on the variance of the datasets.

### Heritability test: Coefficient of variation (CV) and broad-sense heritability (H^2^)

The CV values were calculated by taking the ratio of the standard deviation over the mean for every metabolite individually for each IL and M82. Subsequently, the resulting CV values were divided into 40 bins of incrementing intervals of 0.1 for which the relative frequencies in the wild type and the ILs were estimated (Figure 5a) in both the fruit and the seed. Broad-sense heritability was calculated by estimating mean square values from ANOVA by applying the following linear model: y_ijkl_ = M+S_i_+R(S)_j(i)_+G_k_+GS_ik_+ε_ijkl_, where y corresponds to the log of a single metabolite, M to the grand mean, S to the season, R(S)_j(i)_ to the effect of replicate j in season i, G to the genotype, and ε to the error term. As for the CV analysis, the results were arranged into bins of 0.1 intervals, for which the absolute amount of each bin was determined separately for each tissue. Heritability was calculated per trait (across all ILs and M82), while the CV of each trait was estimated for each individual IL.

### Candidate gene identification

Following QTL mapping and comparative analysis of various tissue and seasonal maps, introgressed segments of interest were identified for further analysis of candidate gene identification. We used the map of the tomato IL population as provided by the Sol Genome network. This map displays individual chromosomes with restriction sites of the different introgressed segments. It also displays all identified marker genes. We developed a script automatically identifying all marker genes in the HTML code, following its link to the TAIR database and downloading the functionality of the gene as provided. After obtaining all information, each identified marker gene in the sites of interest was manually examined for its functionality and proposed as a potential candidate gene, based on its relevance to metabolic activity; each marker of interest was then subjected to database query, as described above. Genes with unknown functionalities were also included into the list of potential candidate genes.

### Gene sequence and alignment analysis

DNA sequences of *SGN-U261955*, *SGN-U217186*, and S*GN-U242840* of M82 were obtained by BLAST search of predicted cDNA sequence in the NCBI (http://www.ncbi.nlm.nih.gov/) and SOL Genomics Network (http://solgenomics.net/) websites, using sequences of cDNA obtained from the SOL Genomics Network website. DNA of *S. pennellii* was extracted from leaf tissues by using standard protocols. The primers for sequencing of *S. pennellii* were designed on the basis of the sequence of M82 genes (Text S2). The PCR products were cloned and sequenced. The resulting sequences were aligned using Clustal X2 (http://www.clustal.org/) and GeneDoc (http://www.nrbsc.org/gfx/genedoc/).

### Correlation analysis—pairwise correlation

Correlation analysis between all metabolite pairs and metabolite plus morphological trait pairs were performed by averaging IL replicates for each metabolite and morphological trait. Correlations across whole populations were calculated by using Pearson's product-moment correlation (Pearson's ρ), as provided by the R statistical software. Prior to correlation calculations for metabolite and morphological trait pairs, data was standardized by Z-score transformation. A Z-score quantifies the original value in terms of the number of standard deviations of that score from the mean of the distribution and thereby facilitates comparisons of observations from different normal distributions. If the variable to be transformed is the sample mean, then the standard deviation is substituted for the standard error. Corresponding p-values were calculated using the cor.test function as provided by R, which analyzes the association between paired samples.

### Network analysis

Networks constructed for seed and fruit metabolites, in separate and holistic modes, were based on correlation analyses. To combine metabolic data from the fruit and the seed, each data set was normalized by its tissue weight. For the seed we utilized the correlation matrix described above, and for the fruit we calculated correlations based on Pearson's product-moment correlation. For the normalized combinatorial data set, the Pearson's product-moment correlation was also applied. Correlations between all metabolites were tested by using IL mean values (n = 76 lines) in season I. Since the metabolites yielded 3655 and 2016 pairs in the fruit and seed matrix, respectively, we chose a critical p-value of ≤0.01 and an *r*–value of ≥0.3 to detect significant correlations and to generate adjacency matrices. The 689 and 383 resulting pairs for the seed and fruit networks, respectively, were depicted as a cartographic network, where a node corresponds to a metabolite and a link between two nodes represents a significant correlation between those two metabolites. All computations and preparation for subsequent network visualizations were generated in R. The graphical presentation of the network was composed with Cytoscape version 2.7.0. Network statistics were computed utilizing the plugin NetworkAnalyzer 2.6.1 [98] developed at the Max Planck Institute for Informatic (MPII) in Saarbruecken, Germany for the following network parameters: degree of connectivity, defined as the average number of edges adjacent to the nodes in a network; the clustering coefficient, describing the local cohesiveness of a network and computing the probability of connectivity of two nodes with a common neighbor; the network density, characterizing the proportion of edges in a network in relation to the total amount of potential edges in a network; and the diameter, which is defined as the longest path among all shortest paths over all pairs of nodes present in the network [99]. Module identifications were performed utilizing the plugin NeMo (Network Module Identification) v1.4 [72], which detects modules based on high connectivity. To verify modules as displayed by the NeMo plugin, we computed the modularity value Q by predefining memberships assigning seed metabolites to one cluster and fruit metabolites to another cluster. We then permuted the membership vector 10^5^ times, recalculating Q and computing p. As a second approach, we applied the fast greedy community algorithm to verify the tightly connected seed metabolism, as observed before. We also calculated Q, recording the maximal value as computed by the algorithm. We randomized the seed-fruit network 10^4^ times and recalculated max Q for each iteration to compute p. All calculations were performed utilizing functions of the igraph package in R.

### Network measure comparison

The average variance of the seed season I dataset was computed, under consideration of outliers, by independently calculating the variance of each metabolite for each IL and estimating the average. Similarly, the variance of metabolites for every IL was calculated in the fruit dataset. Subsequently, the average variance was calculated for each IL. The single fruit IL variance averages were ranked in accordance to the seed season I dataset average variance (closest to furthest). The differences of the single fruit IL variances to the averaged seed variance were determined by applying F-value statistics. Iteratively subsets of the ranked ILs containing the average metabolic values were used to create networks and compute network measures by using functions of the R igraph package. To be consistent with former correlation based network construction, we applied the same thresholds for correlation coefficients (0.3) and p-values (0.01). At each iteration, the number of ranked fruit ILs was incremented, generating subsets of ILs ranging from 15 to 25. Each subset was subjected to permutation tests to evaluate statistical differences between seed metabolite network measures and subset network measures. The proposed null-hypotheses yielded no significant differences between subset network measures and seed network measures.

### Data set availability

The original data set of seed metabolites as generated by GC-MS conductance may be viewed on the Phenome Networks platform (URL listed in the section of URLs). This platform facilitates the manipulation, correlation and analysis of data in a way similar to that described here. Users may view all statistical tests performed here.

### URLs

R statistical software, http://www.R-project.org; Cytoscape: Analyzing and Visualizing Network data, http://www.cytoscape.org; Phenome Networks, http://phnserver.phenome-networks.com/; The Sol Genomics Network, http://solgenomics.net/; SeedNet and SCoPNET, http://vseed.nottingham.ac.uk/; The *Arabidopsis* Information Resource (TAIR), http://www.arabidopsis.org/; The A. thaliana Co-Response Database, http://csbdb.mpimp-golm.mpg.de/csbdb/dbcor/ath/ath_tsgq.html; The KEGG pathway database, http://www.genome.jp/kegg/pathway.html; NCBI, http://www.ncbi.nlm.nih.gov/; ClustalX, http://www.clustal.org/; GeneDoc, http://www.clustal.org/; Israel Meteorological Service, http://ims.gov.il.

## Supporting Information

Figure S1 Heat map of metabolites that changed significantly across the IL collection during season I. Heat map representation of significant metabolites identified by Dunnett's test as applied to IL season I harvest in Akko, Israel, as compared with cultivar M82 (control) analyzed on dry seeds. Each metabolite was individually compared with M82 on each IL. Colored rectangles indicate a significant change as compared with the control. A probability threshold of ≤0.01 is illustrated. Metabolites were categorized according to their compound class. Groups of metabolites yielding highly significant changes (p<0.001) are delimited within black rectangles and magnified outside the heat map.
(PDF)

Figure S2 Co-localization of seed and fruit mQTL. Map of co-localized mQTL of fumarate, malate, fructose, and glucose in the seed and fruit as analyzed on season I and II harvests in Akko, Israel. Co-localizations of named metabolites show a putative tissue independent relationship between the two TCA cycle intermediates and the two monosaccharaides.
(PDF)

Figure S3 Metabolite-metabolite correlation. Visualization of metabolite-metabolite correlation. Heat map of metabolite-metabolite correlations as analyzed on dry IL seeds of IL harvest season I in Akko, Israel. Metabolites were categorized according to their compound class. The Pearson product-moment correlation was employed to compute correlation between metabolites heading the rows and metabolites heading the columns. Each colored rectangle depicts an *r* value resulting from the computation (see color key top right). Regions with accumulated high correlation throughout various metabolites are identified by black rectangles and magnified outside the heat map.
(PDF)

Figure S4 Morphological traits – metabolite correlation. Depiction of correlation between metabolic data as analyzed on dry IL seeds harvest season I in Akko, Israel and morphological traits of the ILs. The Pearson product-moment correlation was employed to compute correlation between morphological traits heading the rows and metabolites heading the columns. Colored rectangles display *r* values resulting from Pearson's correlation coefficient computations (see color key). *Z*-score transformation was used to enable correlation calculations. Areas with high correlation are denoted by black rectangles and magnified outside the graph. Metabolites were categorized according to their compound class.
(PDF)

Figure S5 Seed metabolite network. Network visualization of metabolites as analyzed on dry IL seeds of harvest seasons I and II in Akko, Israel. Metabolites are presented as nodes, and their relations, as edges. Metabolites are color-coded and clustered according to the compound classes. The Pearson product-moment correlation was applied across the entire set of ILs to compute pairwise correlations. Only significant correlations are depicted. A significance level of <0.01 and an *r*-value of >0.3 were considered to be significant. Positive correlations are shown as blue edges, negative correlations, as red edges.
(PDF)

Figure S6 Fruit metabolite network. Network visualization of metabolites as analyzed on IL fruits of harvest season I in Akko, Israel. Metabolites are presented as nodes, and their relations, as edges. Metabolites are color-coded and modulated according to the compound classes The Pearson product-moment correlation was applied across the entire set of ILs to compute pairwise correlations. Only significant correlations are depicted. A significance level of ≤0.01 and an *r*-value of ≥0.3 were considered to be significant.
(PDF)

Figure S7 Seed metabolite network union of seasons I and II. Network visualization of metabolites as analyzed on dry IL seeds of harvest seasons I and II in Akko, Israel. The networks of the two seasons were converged into a single network. Metabolites are presented as nodes, and their relations, as edges, where red edges represent conserved correlations, green edges represent correlations occurring solely in season I, and blue edges represent correlations occurring solely in season II. Metabolites are color-coded and clustered according to the compound classes. The Pearson product-moment correlation was applied across the entire set of ILs to compute pairwise correlations. Only significant correlations are depicted. A significance level of <0.01 and an *r*-value of >0.3 were considered to be significant. Computations of the correlations were conducted under the R environment. Cytoscape was used to generate the graphical output of the network.
(PDF)

Figure S8 Degree of connectivity cluster network of seasons I and II. Network visualization of metabolites as analyzed on dry IL seeds of harvest seasons I and II in Akko, Israel. Metabolites are presented as nodes, and their relations, as edges. The Pearson product-moment correlation was employed to compute all pairwise correlations across the entire set of ILs. Only significant correlations are depicted. Clusters of high connectivity of metabolites were generated on the basis of the network presented in Figure S5 by applying the clustering algorithm supplied by the NeMo for Cytoscape plug-in. Metabolites were clustered together on the basis of the degree of connectivity to adjacent metabolites. Computations of the correlations were conducted under the R environment. Cytoscape was used to generate graphical output of network.
(PDF)

Figure S9 Seed metabolite network intersect of seasons I and II – compound class and degree of connectivity cluster view. Network visualization of metabolites as analyzed on dry IL seeds of harvest seasons I and II in Akko, Israel. The networks of the two seasons were converged into one network by selecting only the conserved correlations throughout both seasons. Metabolites are presented as nodes, and their relations, as edges, where red edges represent conserved correlations. The Pearson product-moment correlation was employed to compute all pairwise correlations across the entire set of ILs. Only significant correlations are depicted. A significance level of ≤0.01 and an *r*-value of ≥0.3 were considered to be significant. Left region: Metabolites are color coded and modulated according to the compound classes. Right region: Clusters of high connectivity of metabolites were generated on the basis of the network presented in in Figure S5 by applying the clustering algorithm supplied by the NeMo for Cytoscape plug-in. Metabolites are clustered together on the basis of the degree of connectivity to adjacent metabolites.
(PDF)

Table S1 IL pairwise ANOVA for all metabolites across seed seasons I and II. 2-way ANOVA was performed in a pairwise manner for each metabolite in corresponding ILs of the two seed seasons (I and II). The Table shows the estimated p-values for each IL and metabolite for the single term ‘genotype’ (Table S1a) and the interaction term ‘season * genotype’ (Table S1b).
(XLS)

Table S2 Candidate gene *At5g10920* and its correlated genes associated with glycolysis on IL 4-3. Candidate gene *At5g10920* as identified on IL 4-3 putatively associated with glycolysis and correlated genes as generated by SeedNet available on http://vseednet.nottingham.ac.uk. The candidate gene is involved in Arg biosynthesis. The correlated genes are supplied with the Pearson correlation coefficient values. Correlated genes of relevance to glycolysis are highlighted in grey. Localization of gene candidates was achieved by utilizing data as analyzed on dry IL seeds of harvest seasons I and II in Akko, Israel.
(PDF)

Table S3 Candidate gene *At5g63840* and its correlated genes associated with glycolysis on IL 4-3-2. Candidate gene *At5g63840* as identified on IL 4-3-2 putatively associated with glycolysis and co-predicted genes as generated by SCoPNET available on http://vseednet.nottingham.ac.uk. The candidate gene is involved in cellulose biosynthetic processes with glucosidase and hydrolase activities. The co-predicted genes are supplied with the co-prediction PMI values. Co-predicted genes of relevance to glycolysis are highlighted in grey. Localization of gene candidates was achieved by utilizing data as analyzed on dry IL seeds of harvest seasons I and II in Akko, Israel.
(PDF)

Table S4 Candidate gene *At1g14810* and its correlated genes associated with organic acid and hexose sugars on IL 1-1-3. Candidate gene *At1g14810* as identified on IL 1-1-3 putatively associated with organic acid and hexose sugars and correlated genes as generated by SeedNet available on http://vseednet.nottingham.ac.uk. The candidate gene codes for an aspartate semialdeyhde dehydrogenase. The co-predicted genes are supplied with Pearson's coefficient values. Correlated genes of relevance to glycolysis are highlighted in grey. Localization of gene candidates was achieved by utilizing data as analyzed on dry IL seeds of harvest seasons I and II in Akko, Israel.
(PDF)

Table S5 Candidate gene *At1g20575* and its correlated genes associated with organic acid and hexose sugars on IL 4-4. Candidate gene *At1g20575* as identified on IL 4-4 putatively associated with organic acids and hexose sugars and co-predicted genes as generated by SCoPNET available on http://vseednet.nottingham.ac.uk. The candidate gene is involved in D-ribose catabolism. The co-predicted genes are supplied with the co-prediction PMI values. Co-predicted genes of relevance to organic acids or sugars are highlighted in grey. Localization of gene candidates was achieved by utilizing data as analyzed on dry IL seeds of harvest seasons I and II in Akko, Israel.
(PDF)

Table S6 Candidate gene *At1g20575* involved in the D-ribose catabolism and its correlated genes associated with organic acid and hexose sugars on IL 4-4. Candidate gene *At1g20575* as identified on IL 4-4 putatively associated with organic acid and hexose sugars and correlated genes as generated by SeedNet available on http://vseednet.nottingham.ac.uk. The candidate gene is involved in D-ribose catabolism. The co-predicted genes are supplied with Pearson's coefficient values. Correlated genes of relevance to organic acids or sugars are highlighted in grey. Localization of gene candidates was achieved by utilizing data as analyzed on dry IL seeds of harvest seasons I and II in Akko, Israel.
(PDF)

Table S7 Parameters as calculated on seed and fruit networks. Based on the networks presented in Figure 7, Figure 8, and Figures S5, S6, S7, S8, S9, the following typical network typical parameters at different *r* thresholds were computed: number of nodes, number of edges, degree of connectivity, clustering coefficients, network density, and network diameter. In addition, the percentage of the significant correlations at a given *r* threshold in accordance to the total number of correlations were calculated.
(PDF)

Table S8 Fruit ILs ranked based on individual variances compared with overall average variance of seed IL population. Shown are the 25 fruit ILs chosen for the subset network parameters comparison with reference to the network parameters of the seed IL network. ILs were ranked based on their variance as compared with the average variances of the seed IL dataset. To show differences of variances F-statistics were applied to estimate p-values. None of the chosen ILs show significant differences between individual IL variance and overall average seed IL dataset variance.
(PDF)

Table S9 Conserved correlations across seasons and tissues. Conserved correlations across seasons within the same tissue. Conserved correlations of combinatorial fruit-seed networks were also compared for different seasons. To compare networks, datasets must be synchronized for metabolites, which leads to loss of correlations. Roman Capitals refer to harvest seasons.
(PDF)

Text S1 Candidate gene identification via the *A. thaliana* co-response database.
(PDF)

Text S2 Candidate gene BLAST results against *Solanum pennellii*.
(PDF)

Text S3 Confirmation of season I seed network via repetition of analysis on season II seed network.
(PDF)
