# Supplementary figures and images for: Metabolic Profiling of a Mapping Population Exposes New Insights in the Regulation of Seed Metabolism and Seed, Fruit, and Plant Relations
Source: PLoS Genet. 2012 Mar 29;8(3):e1002612. doi: 10.1371/journal.pgen.1002612 (PMC3315483; doi:10.1371/journal.pgen.1002612)

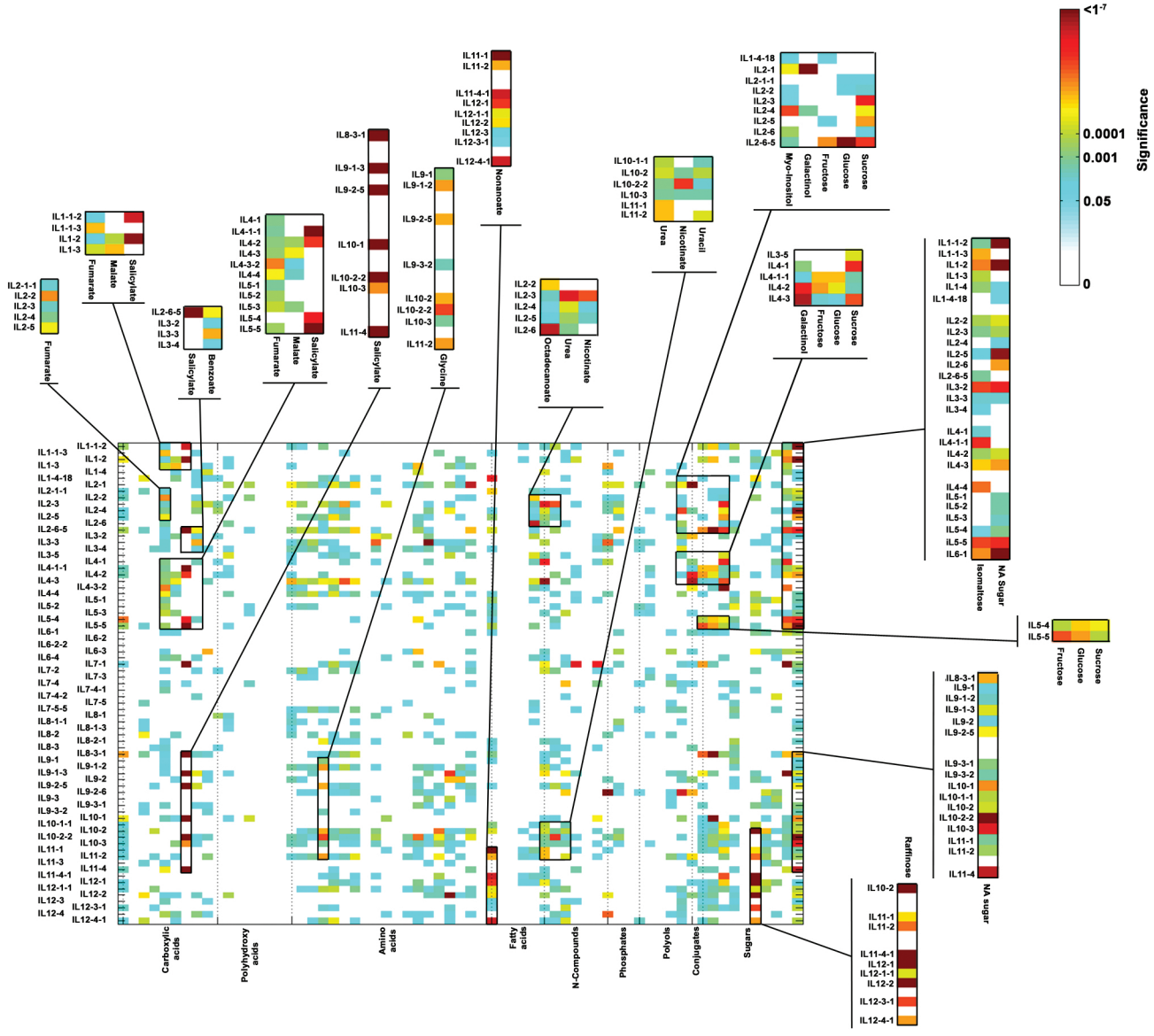

Supplement: Figure S1 — Heat map of metabolites that changed significantly across the IL collection during season I. Heat map representation of significant metabolites identified by Dunnett's test as applied to IL season I harvest in Akko, Israel, as compared with cultivar M82 (control) analyzed on dry seeds. Each metabolite was individually compared with M82 on each IL. Colored rectangles indicate a significant change as compared with the control. A probability threshold of ≤0.01 is illustrated. Metabolites were categorized according to their compound class. Groups of metabolites yielding highly significant changes (p<0.001) are delimited within black rectangles and magnified outside the heat map. (PDF) [file pgen.1002612.s001.pdf]

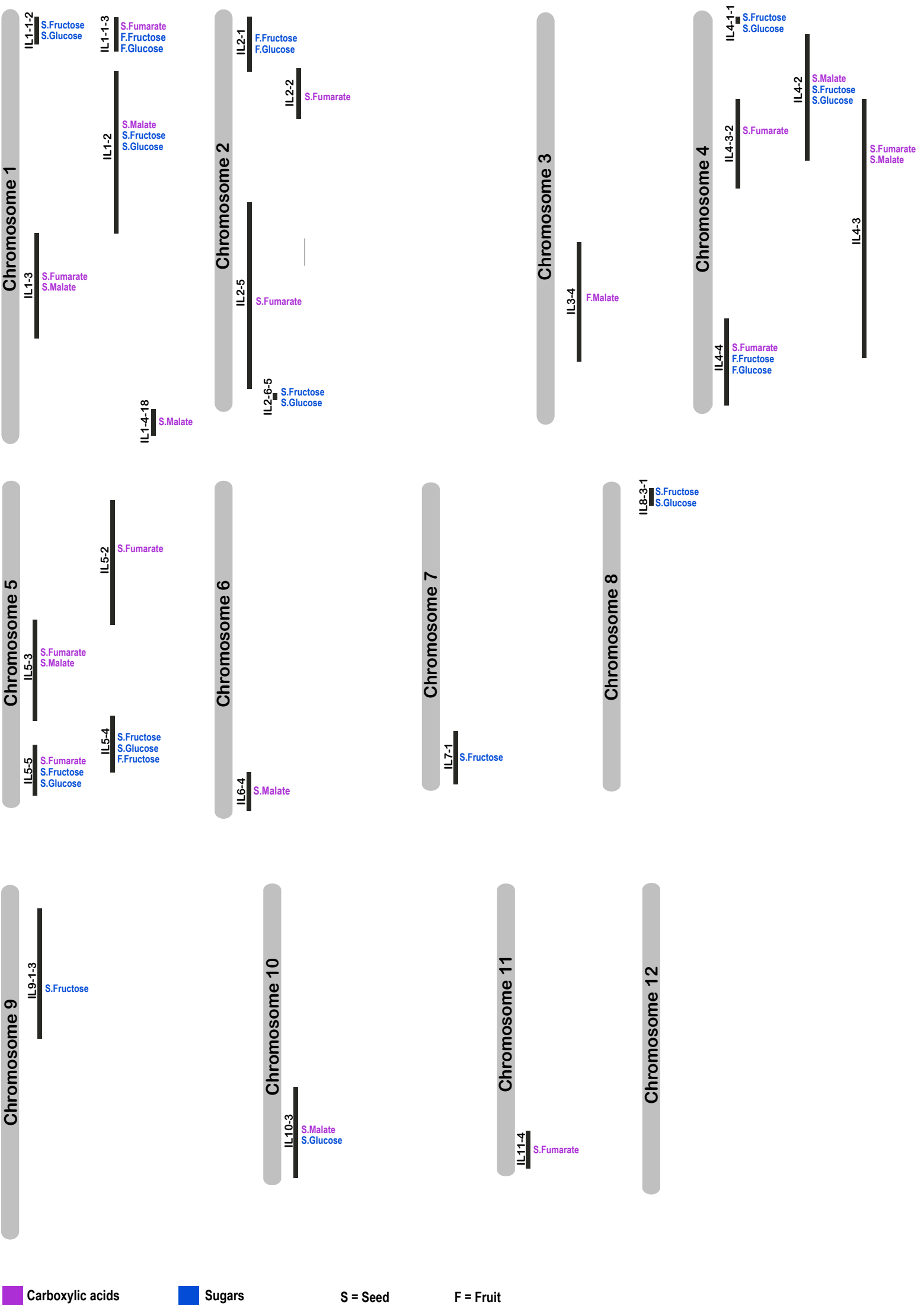

Supplement: Figure S2 — Co-localization of seed and fruit mQTL. Map of co-localized mQTL of fumarate, malate, fructose, and glucose in the seed and fruit as analyzed on season I and II harvests in Akko, Israel. Co-localizations of named metabolites show a putative tissue independent relationship between the two TCA cycle intermediates and the two monosaccharaides. (PDF) [file pgen.1002612.s002.pdf]

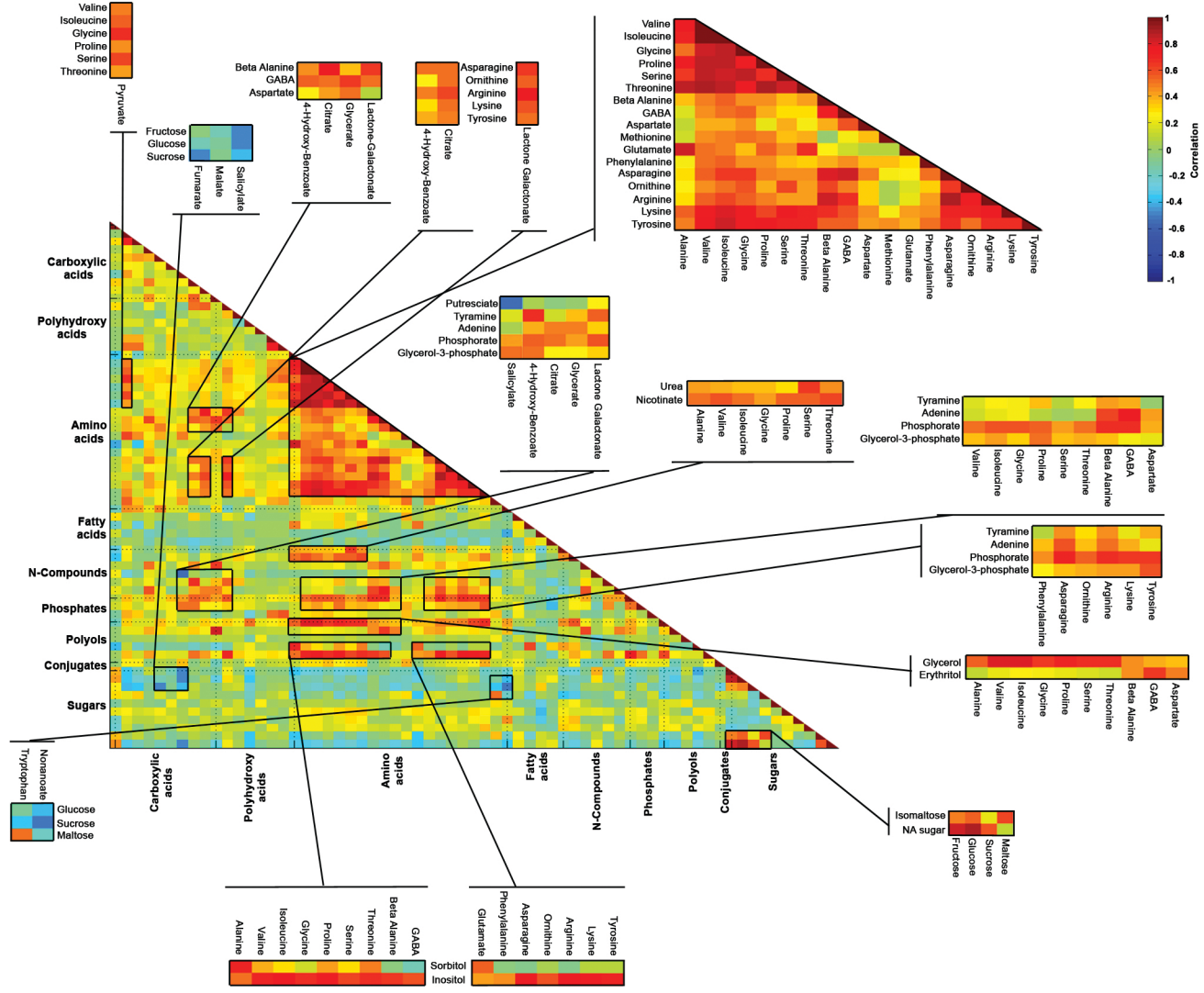

Supplement: Figure S3 — Metabolite-metabolite correlation. Visualization of metabolite-metabolite correlation. Heat map of metabolite-metabolite correlations as analyzed on dry IL seeds of IL harvest season I in Akko, Israel. Metabolites were categorized according to their compound class. The Pearson product-moment correlation was employed to compute correlation between metabolites heading the rows and metabolites heading the columns. Each colored rectangle depicts an r value resulting from the computation (see color key top right). Regions with accumulated high correlation throughout various metabolites are identified by black rectangles and magnified outside the heat map. (PDF) [file pgen.1002612.s003.pdf]

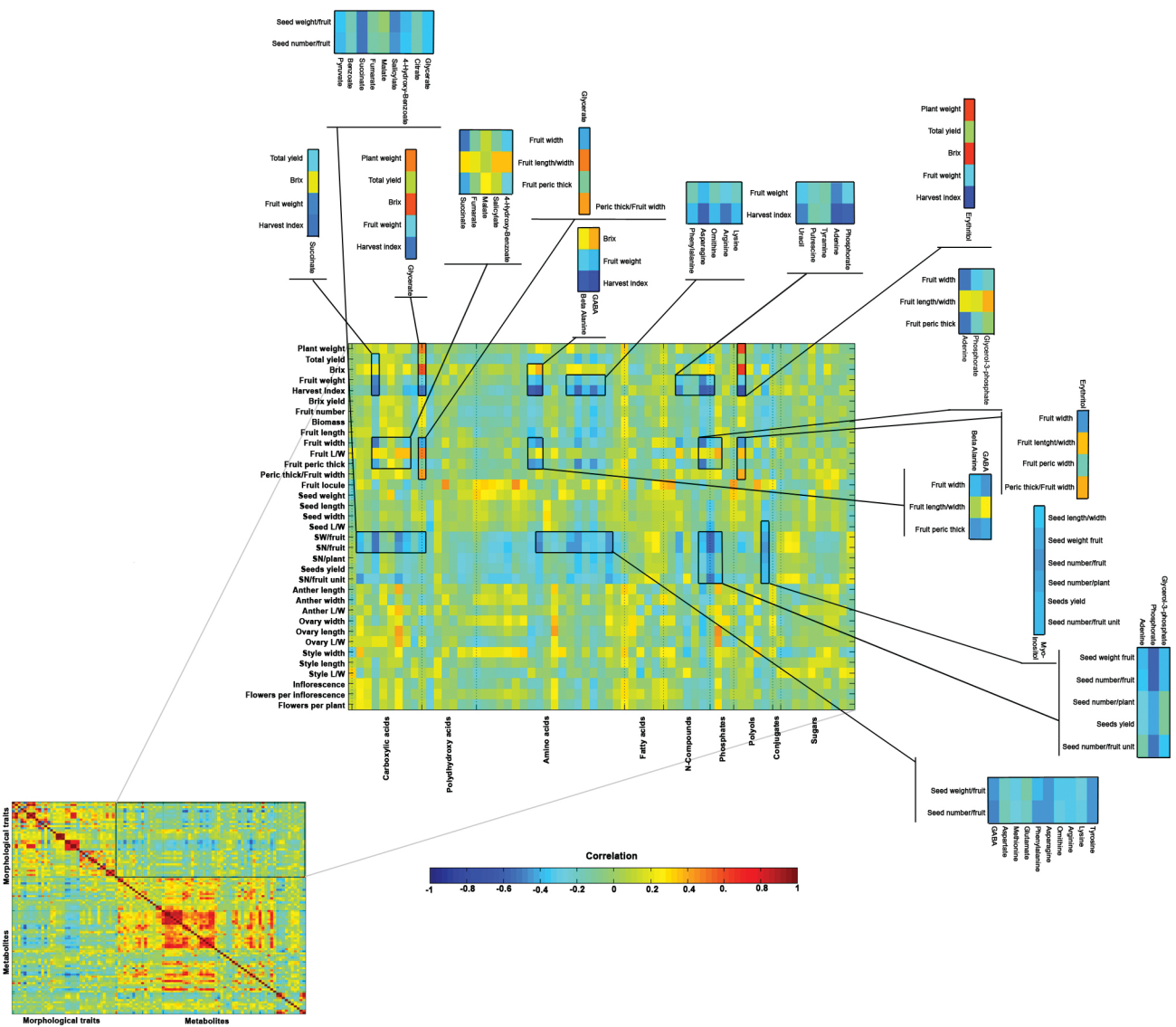

Supplement: Figure S4 — Morphological traits – metabolite correlation. Depiction of correlation between metabolic data as analyzed on dry IL seeds harvest season I in Akko, Israel and morphological traits of the ILs. The Pearson product-moment correlation was employed to compute correlation between morphological traits heading the rows and metabolites heading the columns. Colored rectangles display r values resulting from Pearson's correlation coefficient computations (see color key). Z-score transformation was used to enable correlation calculations. Areas with high correlation are denoted by black rectangles and magnified outside the graph. Metabolites were categorized according to their compound class. (PDF) [file pgen.1002612.s004.pdf]

Fruit metabolite network - p <0.01, r >0.3

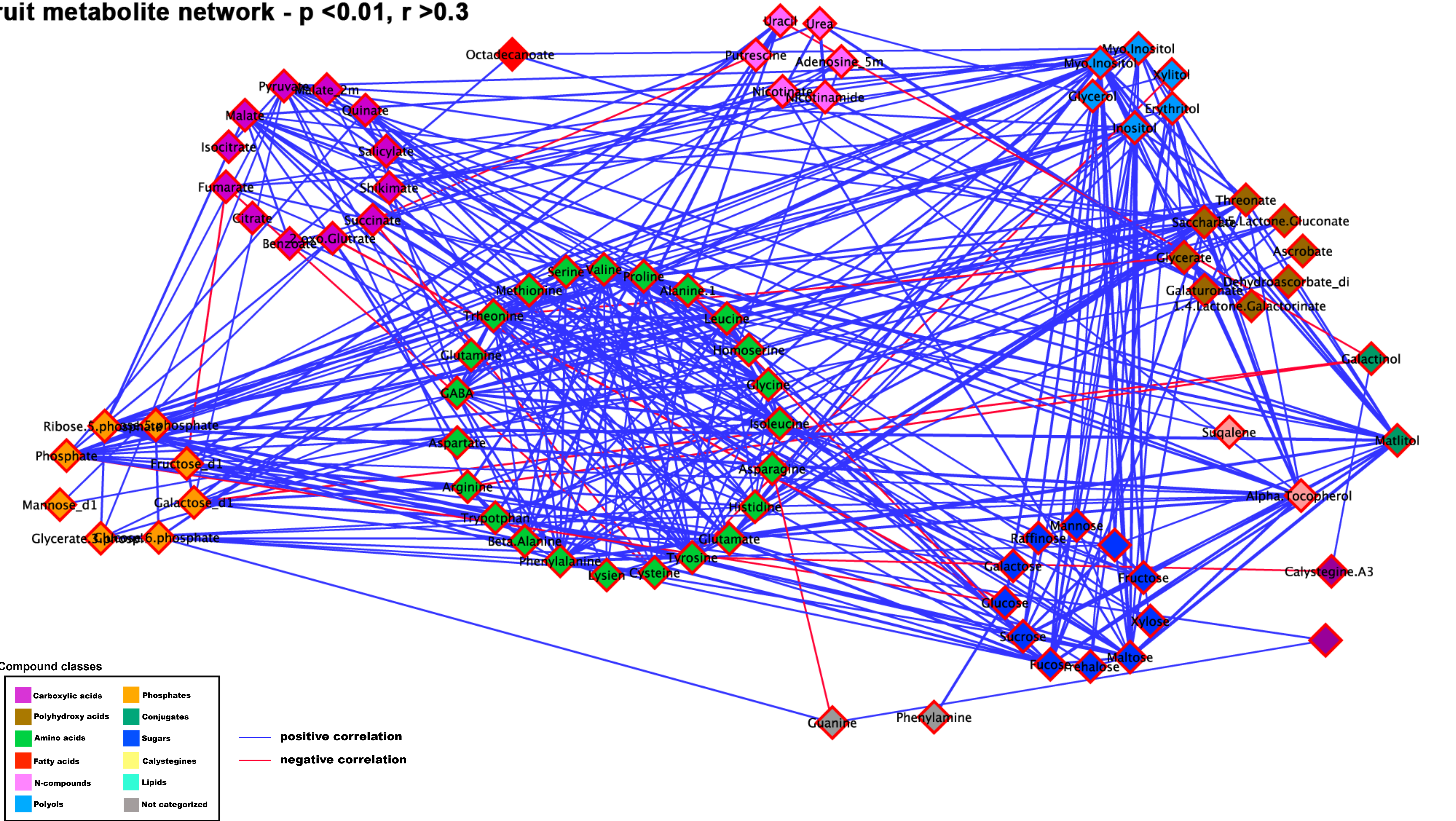

Supplement: Figure S6 — Fruit metabolite network. Network visualization of metabolites as analyzed on IL fruits of harvest season I in Akko, Israel. Metabolites are presented as nodes, and their relations, as edges. Metabolites are color-coded and modulated according to the compound classes The Pearson product-moment correlation was applied across the entire set of ILs to compute pairwise correlations. Only significant correlations are depicted. A significance level of ≤0.01 and an r-value of ≥0.3 were considered to be significant. (PDF) [file pgen.1002612.s006.pdf]

**$r \geq 0.3, p \leq 0.01$**

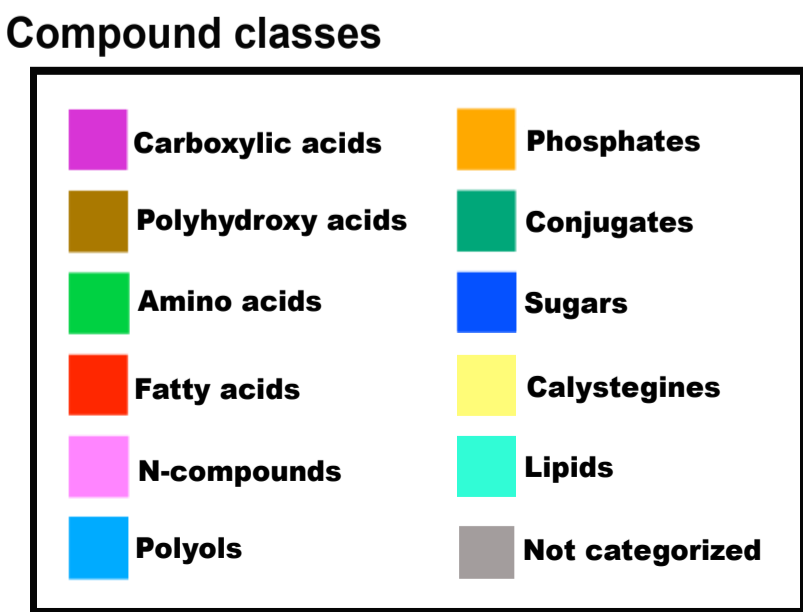

**$r \geq 0.3, p \leq 0.01$**

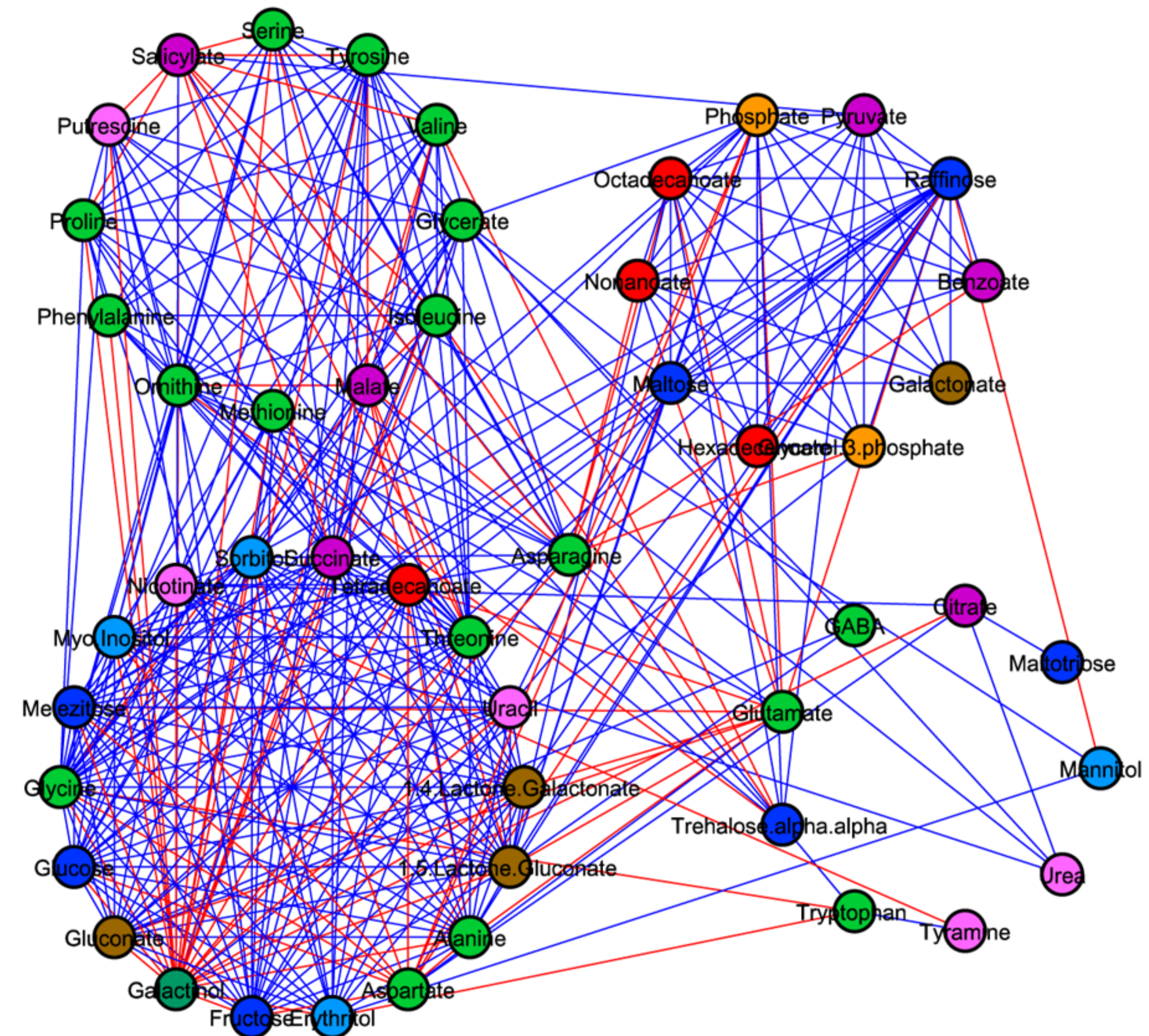

Supplement: Figure S8 — Degree of connectivity cluster network of seasons I and II. Network visualization of metabolites as analyzed on dry IL seeds of harvest seasons I and II in Akko, Israel. Metabolites are presented as nodes, and their relations, as edges. The Pearson product-moment correlation was employed to compute all pairwise correlations across the entire set of ILs. Only significant correlations are depicted. Clusters of high connectivity of metabolites were generated on the basis of the network presented in Figure S5 by applying the clustering algorithm supplied by the NeMo for Cytoscape plug-in. Metabolites were clustered together on the basis of the degree of connectivity to adjacent metabolites. Computations of the correlations were conducted under the R environment. Cytoscape was used to generate graphical output of network. (PDF) [file pgen.1002612.s008.pdf]
