# Supplementary material for: Metabolic Profiling of a Mapping Population Exposes New Insights in the Regulation of Seed Metabolism and Seed, Fruit, and Plant Relations
Source: PLoS Genet. 2012 Mar 29;8(3):e1002612. doi: 10.1371/journal.pgen.1002612 (PMC3315483; doi:10.1371/journal.pgen.1002612)
Supplement: Figure S5 — Seed metabolite network. Network visualization of metabolites as analyzed on dry IL seeds of harvest seasons I and II in Akko, Israel. Metabolites are presented as nodes, and their relations, as edges. Metabolites are color-coded and clustered according to the compound classes. The Pearson product-moment correlation was applied across the entire set of ILs to compute pairwise correlations. Only significant correlations are depicted. A significance level of <0.01 and an r-value of >0.3 were considered to be significant. Positive correlations are shown as blue edges, negative correlations, as red edges. (PDF) [file pgen.1002612.s005.pdf]

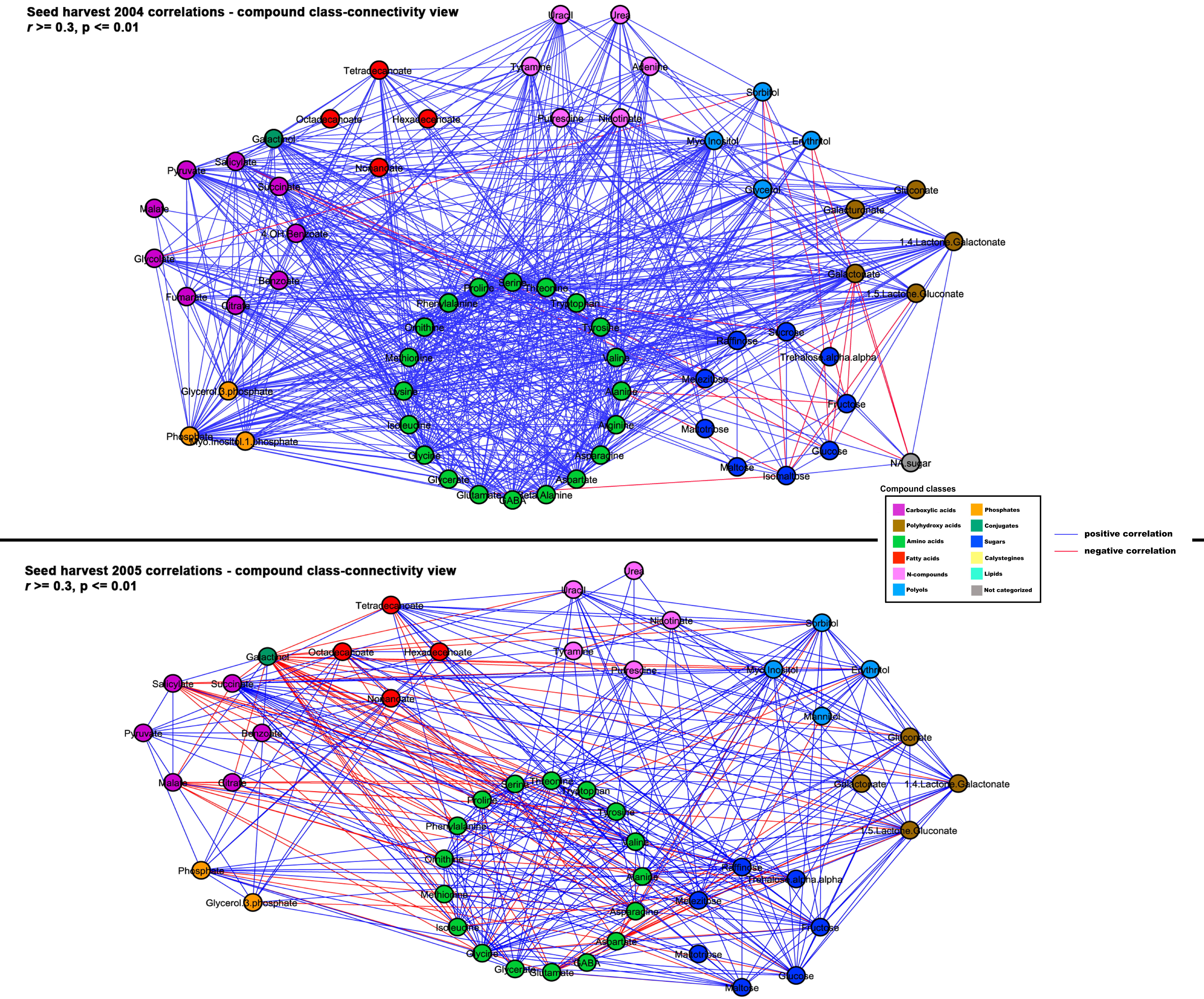

Seed harvest 2004 correlations - compound class-connectivity view
r >= 0.3, p <= 0.01
Uracil
Urea
Tetradecanoate
Tyramine
Adenine
Sorbitol
Octadecanoate
Hexadecenoate
Putrescine
Nicotinate
Galactinol
Myo.Inositol
Erythritol
Salicylate
Nonanoate
Pyruvate
Succinate
Glycerol
Gluconate
Malate
Galacturonate
4.OH.Benzoate
1.4.Lactone.Galactonate
Glycolate
Benzoate
Galactonate
Proline
Serine
Threonine
Fumarate
Citrate
Phenylalanine
Tryptophan
1.5.Lactone.Gluconate
Ornithine
Tyrosine
Sucrose
Raffinose
Methionine
Valine
Trehalose.alpha.alpha
Melezitose
Glycerol.3.phosphate
Lysine
Alanine
Fructose
Isoleucine
Arginine
Maltotriose
Phosphate
Myo.Inositol.1.phosphate
Glycine
Asparagine
Glucose
NA.sugar
Maltose
Glycerate
Aspartate
Isomaltose
Glutamate
GABA
Beta.Alanine
Compound classes
Carboxylic acids
Phosphates
Polyhydroxy acids
Conjugates
Amino acids
Sugars
Fatty acids
Calystegines
N-compounds
Lipids
Polyols
Not categorized
positive correlation
negative correlation
Seed harvest 2005 correlations - compound class-connectivity view
r >= 0.3, p <= 0.01
Urea
Uracil
Tetradecanoate
Nicotinate
Sorbitol
Galactinol
Octadecanoate
Hexadecenoate
Tyramine
Putrescine
Myo.Inositol
Erythritol
Salicylate
Succinate
Nonanoate
Mannitol
Pyruvate
Benzoate
Gluconate
Malate
Citrate
Serine
Threonine
Tryptophan
Galactonate
1.4.Lactone.Galactonate
Proline
Tyrosine
Phenylalanine
1.5.Lactone.Gluconate
Valine
Ornithine
Raffinose
Phosphate
Alanine
Trehalose.alpha.alpha
Methionine
Melezitose
Glycerol.3.phosphate
Asparagine
Isoleucine
Fructose
Aspartate
Glycine
Maltotriose
GABA
Glycerate
Glutamate
Glucose
Maltose
