# Supplementary material for: Metabolic Profiling of a Mapping Population Exposes New Insights in the Regulation of Seed Metabolism and Seed, Fruit, and Plant Relations
Source: PLoS Genet. 2012 Mar 29;8(3):e1002612. doi: 10.1371/journal.pgen.1002612 (PMC3315483; doi:10.1371/journal.pgen.1002612)

Seed harvests 2004 & 2005 correlations - compound class-connectivity view
***r* >= 0.3, p <= 0.01**

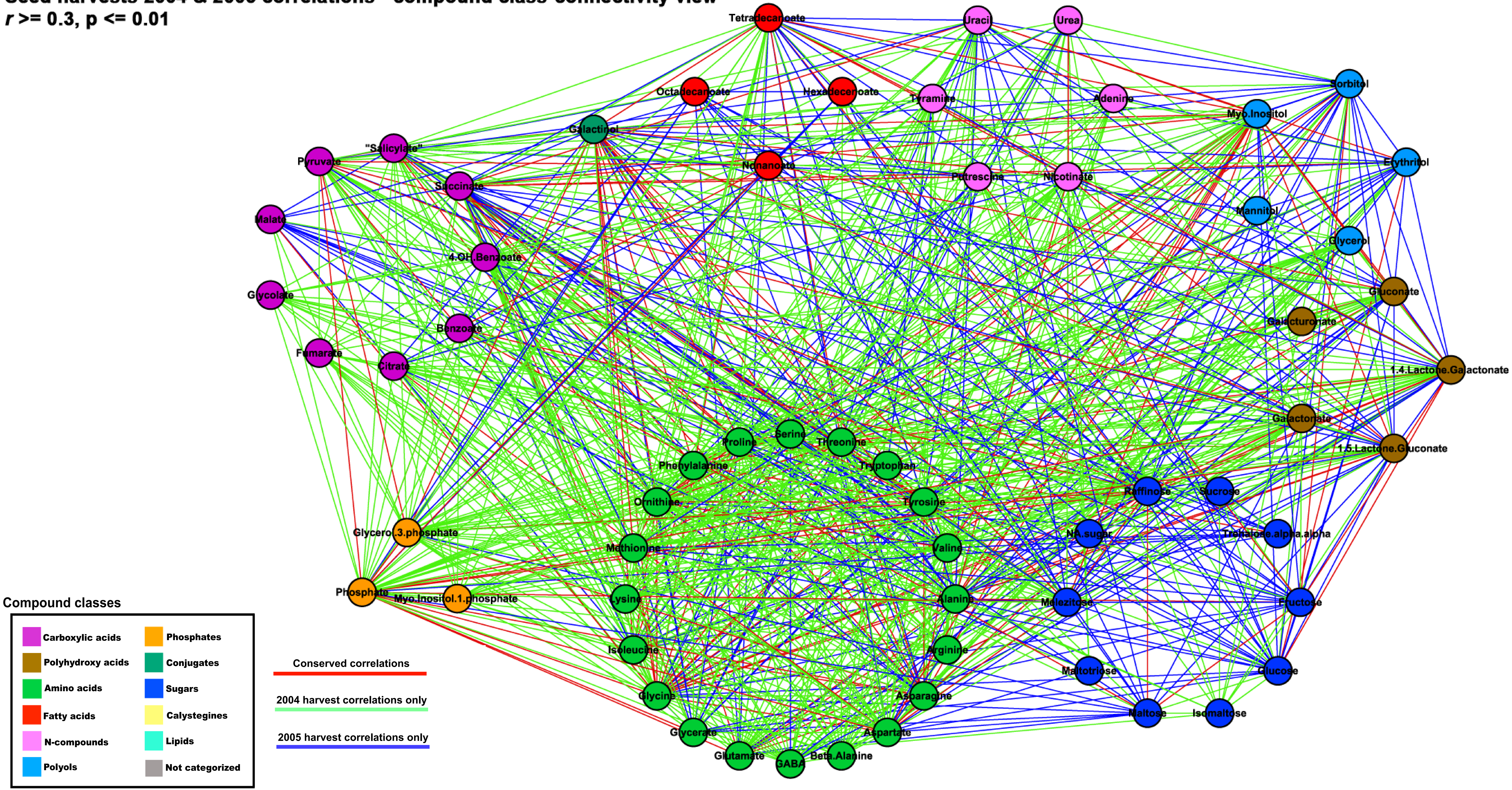

Supplement: Figure S7 — Seed metabolite network union of seasons I and II. Network visualization of metabolites as analyzed on dry IL seeds of harvest seasons I and II in Akko, Israel. The networks of the two seasons were converged into a single network. Metabolites are presented as nodes, and their relations, as edges, where red edges represent conserved correlations, green edges represent correlations occurring solely in season I, and blue edges represent correlations occurring solely in season II. Metabolites are color-coded and clustered according to the compound classes. The Pearson product-moment correlation was applied across the entire set of ILs to compute pairwise correlations. Only significant correlations are depicted. A significance level of <0.01 and an r-value of >0.3 were considered to be significant. Computations of the correlations were conducted under the R environment. Cytoscape was used to generate the graphical output of the network. (PDF) [file pgen.1002612.s007.pdf]
