# Supplementary material for: Metabolic Profiling of a Mapping Population Exposes New Insights in the Regulation of Seed Metabolism and Seed, Fruit, and Plant Relations
Source: PLoS Genet. 2012 Mar 29;8(3):e1002612. doi: 10.1371/journal.pgen.1002612 (PMC3315483; doi:10.1371/journal.pgen.1002612)

Seed harvests 2004-2005 conserved correlations - compound class-connectivity view
***r* >= 0.3, p <= 0.01**

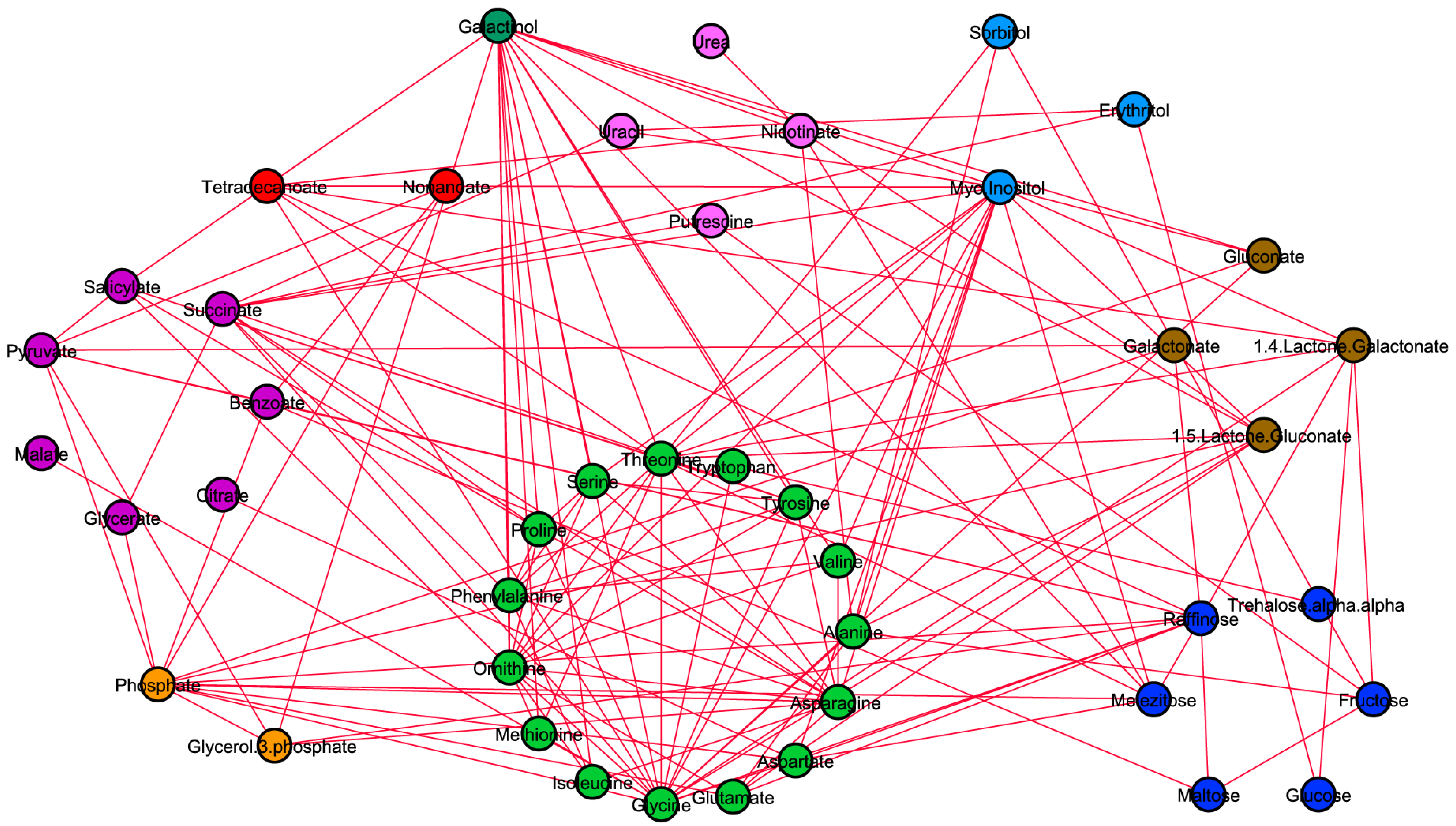

Seed harvests 2004-2005 conserved correlations - cluster view ***r* >= 0.3, p <= 0.01**

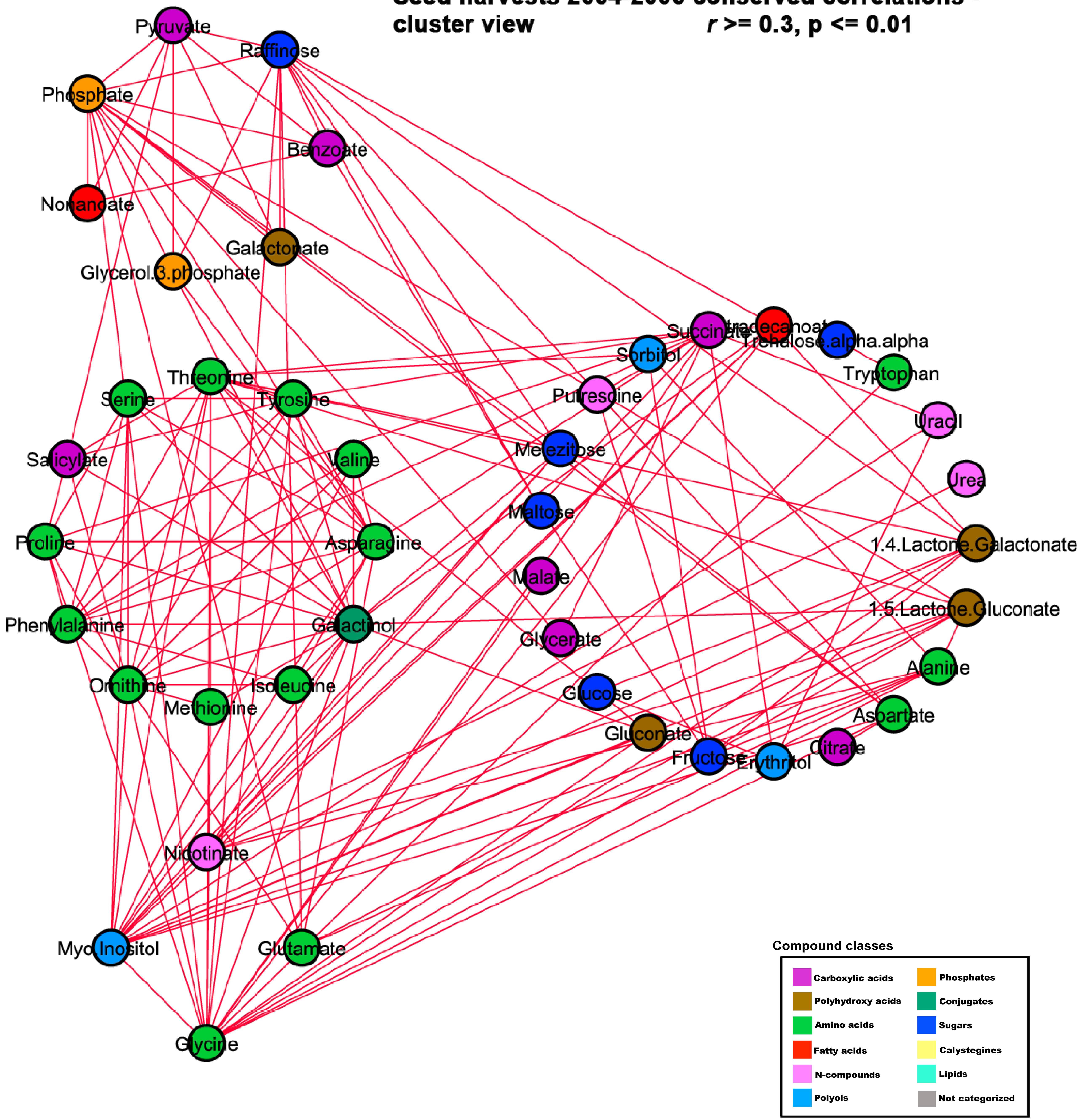

Supplement: Figure S9 — Seed metabolite network intersect of seasons I and II – compound class and degree of connectivity cluster view. Network visualization of metabolites as analyzed on dry IL seeds of harvest seasons I and II in Akko, Israel. The networks of the two seasons were converged into one network by selecting only the conserved correlations throughout both seasons. Metabolites are presented as nodes, and their relations, as edges, where red edges represent conserved correlations. The Pearson product-moment correlation was employed to compute all pairwise correlations across the entire set of ILs. Only significant correlations are depicted. A significance level of ≤0.01 and an r-value of ≥0.3 were considered to be significant. Left region: Metabolites are color coded and modulated according to the compound classes. Right region: Clusters of high connectivity of metabolites were generated on the basis of the network presented in in Figure S5 by applying the clustering algorithm supplied by the NeMo for Cytoscape plug-in. Metabolites are clustered together on the basis of the degree of connectivity to adjacent metabolites. (PDF) [file pgen.1002612.s009.pdf]
