# Supplementary material for: Metabolic Profiling of a Mapping Population Exposes New Insights in the Regulation of Seed Metabolism and Seed, Fruit, and Plant Relations
Source: PLoS Genet. 2012 Mar 29;8(3):e1002612. doi: 10.1371/journal.pgen.1002612 (PMC3315483; doi:10.1371/journal.pgen.1002612)
Supplement: Table S2 — Candidate gene At5g10920 and its correlated genes associated with glycolysis on IL 4-3. Candidate gene At5g10920 as identified on IL 4-3 putatively associated with glycolysis and correlated genes as generated by SeedNet available on http://vseednet.nottingham.ac.uk. The candidate gene is involved in Arg biosynthesis. The correlated genes are supplied with the Pearson correlation coefficient values. Correlated genes of relevance to glycolysis are highlighted in grey. Localization of gene candidates was achieved by utilizing data as analyzed on dry IL seeds of harvest seasons I and II in Akko, Israel. (PDF) [file pgen.1002612.s011.pdf]

SeedNet Correlation Table S2. Candidate gene *At5g10920* and its correlated genes associated with Glycolysis on IL 4-3.

AGI	Links	Annotation	SAM NG/G	ABA Regulation	GA Regulation	Correlation
At1g56050	eFP TAIR Neighbours	GTP-binding protein-related	SAM G			0.87
At1g17200	eFP TAIR Neighbours	integral membrane family protein	SAM G			0.87
At5g24300	eFP TAIR Neighbours	ATSS1/SSI (STARCH SYNTHASE I); transferase, transferring glycosyl groups	SAM G			0.87
At4g33680	eFP TAIR Neighbours	AGD2 (ABERRANT GROWTH AND DEATH 2); transaminase	SAM G			0.87
At5g26710	eFP TAIR Neighbours	glutamate-tRNA ligase, putative / glutamyl-tRNA synthetase, putatuve / GluRS, putative	SAM G			0.87
At3g57610	eFP TAIR Neighbours	ATPURA; adenylosuccinate synthase	SAM G			0.87
At5g20950	eFP TAIR Neighbours	glycosyl hydrolase family 3 protein		ABA Up		0.87
At4g38210	eFP TAIR Neighbours	ATEXPA20 (ARABIDOPSIS THALIANA EXPANSIN A20)	SAM G			0.86
At3g25860	eFP TAIR Neighbours	LTA2 (PLASTID E2 SUBUNIT OF PYRUVATE DECARBOXYLASE); dihydrolipoyllysine-residue acetyltransferase	SAM G			0.86
At5g63810	eFP TAIR Neighbours	BGAL10 (beta-galactosidase 10); beta-galactosidase	SAM G			0.86
At3g16480	eFP TAIR Neighbours	MPPALPHA (mitochondrial processing peptidase alpha subunit); metalloendopeptidase	SAM G			0.86
At5g67510	eFP TAIR Neighbours	60S ribosomal protein L26 (RPL26B)				0.86
At3g25040	eFP TAIR Neighbours	ER lumen protein retaining receptor, putative / HDEL receptor, putative	SAM G			0.86
At3g14310	eFP TAIR Neighbours	ATPME3 (Arabidopsis thaliana pectin methylesterase 3)	SAM G			0.85
At1g31860	eFP TAIR Neighbours	AT-IE (Arabidopsis thaliana bifunctional HisI-HisE protein)				0.85
At1g68560	eFP TAIR Neighbours	ATXYL1/XYL1 (ALPHA-XYLOSIDASE 1); alpha-N-arabinofuranosidase/ hydrolase, hydrolyzing O-glycosyl compounds / xylan 1,4-beta-xylosidase		ABA Up		0.85
At2g47930	eFP TAIR Neighbours	AGP26/ATAGP26 (ARABINOGALACTAN PROTEINS 26)	SAM G			0.85
At4g30800	eFP TAIR Neighbours	40S ribosomal protein S11 (RPS11B)	SAM G			0.85
At1g60160	eFP TAIR Neighbours	potassium transporter family protein	SAM G			0.85
At1g71100	eFP TAIR Neighbours	RSW10 (RADIAL SWELLING 10); ribose-5-phosphate isomerase				0.85
At1g13270	eFP TAIR Neighbours	MAP1C (METHIONINE AMINOPEPTIDASE 1B); metalloexopeptidase	SAM G	ABA Down		0.85
At2g44040 At3g59890	eFP TAIR Neighbours	[AT2G44040, dihydrodipicolinate reductase family protein];[AT3G59890, dihydrodipicolinate reductase family protein]				0.85
At5g48300	eFP TAIR Neighbours	ADG1 (ADP GLUCOSE PYROPHOSPHORYLASE SMALL SUBUNIT 1); glucose-1-phosphate adenylyltransferase	SAM G			0.85
At2g37640	eFP TAIR Neighbours	ATEXPA3 (ARABIDOPSIS THALIANA EXPANSIN A3)			GA Up	0.85
At3g18130	eFP TAIR Neighbours	guanine nucleotide-binding family protein / activated protein kinase C receptor (RACK1)				0.85
At2g20420	eFP TAIR Neighbours	succinyl-CoA ligase (GDP-forming) beta-chain, mitochondrial, putative / succinyl-CoA synthetase, beta chain, putative / SCS-beta, putative				0.85
At3g49680	eFP TAIR Neighbours	ATBCAT-3/BCAT3 (BRANCHED-CHAIN AMINOTRANSFERASE 3); branched-chain-amino-acid transaminase/ catalytic	SAM G	ABA Down		0.85
At1g14810	eFP TAIR Neighbours	semialdehyde dehydrogenase family protein	SAM G			0.85
At5g50370	eFP TAIR Neighbours	adenylate kinase, putative				0.85
At1g27450	eFP TAIR Neighbours	APT1; adenine phosphoribosyltransferase				0.85
At1g17650	eFP TAIR Neighbours	phosphogluconate dehydrogenase (decarboxylating)	SAM G			0.84
At1g68590	eFP TAIR Neighbours	plastid-specific 30S ribosomal protein 3, putative / PSRP-3, putative				0.84
At2g43330	eFP TAIR Neighbours	ATINT1 (INOSITOL TRANSPORTER 1); carbohydrate transmembrane transporter/ sugar:hydrogen ion symporter	SAM G			0.84
At1g63660	eFP TAIR Neighbours	GMP synthase (glutamine-hydrolyzing), putative / glutamine amidotransferase, putative				0.84
At1g32990	eFP TAIR Neighbours	PRPL11 (PLASTID RIBOSOMAL PROTEIN L11); structural constituent of ribosome	SAM G			0.84
At1g48630	eFP TAIR Neighbours	guanine nucleotide-binding family protein / activated protein kinase C receptor, putative / RACK, putative				0.84
At4g35850	eFP TAIR Neighbours	pentatricopeptide (PPR) repeat-containing protein	SAM G			0.84

At1g27970	eFP TAIR Neighbours	NTF2B (NUCLEAR TRANSPORT FACTOR 2B); Ran GTPase binding / protein transporter	SAM G			0.84
At5g49810	eFP TAIR Neighbours	MMT (methionine S-methyltransferase); S-adenosylmethionine-dependent methyltransferase	SAM G	ABA Up		0.84
At4g39880	eFP TAIR Neighbours	ribosomal protein L23 family protein	SAM G			0.84
At5g63980	eFP TAIR Neighbours	SAL1 (FIERY1); 3'(2'),5'-bisphosphate nucleotidase/ inositol or phosphatidylinositol phosphatase	SAM G			0.84
At3g14220	eFP TAIR Neighbours	GDSL-motif lipase/hydrolase family protein				0.84
At3g44590	eFP TAIR Neighbours	60S acidic ribosomal protein P2 (RPP2D)		ABA Down		0.84
At2g40820	eFP TAIR Neighbours	3'-5'-exoribonuclease/ RNA binding	SAM G			0.83
At1g13730	eFP TAIR Neighbours	nuclear transport factor 2 (NTF2) family protein / RNA recognition motif (RRM)-containing protein	SAM G			0.83
At3g46940	eFP TAIR Neighbours	deoxyuridine 5'-triphosphate nucleotidohydrolase family				0.83
At5g11880 At3g14390	eFP TAIR Neighbours	[AT5G11880, diaminopimelate decarboxylase, putative / DAP carboxylase, putative];[AT3G14390, diaminopimelate decarboxylase, putative / DAP carboxylase, putative]				0.83
At5g14040	eFP TAIR Neighbours	mitochondrial phosphate transporter	SAM G			0.83
At4g28360 At1g52370	eFP TAIR Neighbours	[AT4G28360, ribosomal protein L22 family protein];[AT1G52370, ribosomal protein L22 family protein]				0.83
At2g47590	eFP TAIR Neighbours	PHR2 (PHOTOLYASE/BLUE-LIGHT RECEPTOR 2)	SAM G			0.82
At1g12800	eFP TAIR Neighbours	S1 RNA-binding domain-containing protein	SAM G	ABA Down		0.82
At1g69530	eFP TAIR Neighbours	ATEXPA1 (ARABIDOPSIS THALIANA EXPANSIN A1)		ABA Up	GA Up	0.82
At1g44900	eFP TAIR Neighbours	ATP binding / DNA binding / DNA-dependent ATPase	SAM G		GA Up	0.82
At1g34430	eFP TAIR Neighbours	EMB3003 (EMBRYO DEFECTIVE 3003); dihydrolipoyllysine-residue acetyltransferase				0.82
At2g29980	eFP TAIR Neighbours	FAD3 (FATTY ACID DESATURASE 3); omega-3 fatty acid desaturase				0.82
At4g14960	eFP TAIR Neighbours	TUA6 (tubulin alpha-6 chiain)		ABA Down		0.82
At3g56490	eFP TAIR Neighbours	zinc-binding protein, putative / protein kinase C inhibitor, putative				0.82
At3g16780	eFP TAIR Neighbours	60S ribosomal protein L19 (RPL19B)				0.82
At5g22440	eFP TAIR Neighbours	60S ribosomal protein L10A (RPL10aC)				0.82
At2g20450	eFP TAIR Neighbours	60S ribosomal protein L14 (RPL14A)				0.82
At5g48360	eFP TAIR Neighbours	formin homology 2 domain-containing protein / FH2 domain-containing protein	SAM G			0.81
At1g32200	eFP TAIR Neighbours	ATS1 (ACYLTRANSFERASE 1)	SAM G	ABA Down		0.81
At5g60890	eFP TAIR Neighbours	ATMYB34/ATR1/MYB34 (ALTERED TRYPTOPHAN REGULATION, MYB DOMAIN PROTEIN 34); DNA binding / kinase/ transcription activator/ transcription factor	SAM G		GA Up	0.81
At1g31180 At5g14200	eFP TAIR Neighbours	[AT1G31180, 3-isopropylmalate dehydrogenase, chloroplast, putative];[AT5G14200, 3-isopropylmalate dehydrogenase, chloroplast, putative]				0.81
At3g15680	eFP TAIR Neighbours	zinc finger (Ran-binding) family protein	SAM G			0.81
At4g30620	eFP TAIR Neighbours	similar to unknown protein [Arabidopsis thaliana] (TAIR:AT2G24020.1); similar to unknown [Picea sitchensis] (GB:ABK26000.1); similar to Os02g0180200 [Oryza sativa (japonica cultivar-group)] (GB:NP_001046090.1); contains InterPro domain Conserved hypothetical protein CHP00103 (InterPro:IPR004401)	SAM G			0.81
At1g15810	eFP TAIR Neighbours	ribosomal protein S15 family protein	SAM G	ABA Up		0.81
At4g26760	eFP TAIR Neighbours	microtubule associated protein (MAP65/ASE1) family protein	SAM G			0.81
At1g76790	eFP TAIR Neighbours	O-methyltransferase family 2 protein	SAM G			0.81
At2g46170	eFP TAIR Neighbours	reticulon family protein (RTNLB5)	SAM G	ABA Down		0.81
At2g33255	eFP TAIR Neighbours	hydrolase	SAM G			0.81
At2g16430	eFP TAIR Neighbours	ATPAP10/PAP10; protein serine/threonine phosphatase				0.81
At1g01300	eFP TAIR Neighbours	aspartyl protease family protein	SAM G	ABA Up		0.81
At1g80530	eFP TAIR Neighbours	nodulin family protein	SAM G			0.81
At5g51550	eFP TAIR Neighbours	phosphate-responsive 1 family protein	SAM G			0.81
At3g10610	eFP TAIR Neighbours	40S ribosomal protein S17 (RPS17C)				0.81

At3g49080	eFP TAIR Neighbours	ribosomal protein S9 family protein	SAM G			0.81
At3g27740	eFP TAIR Neighbours	CARA (CARBAMOYL PHOSPHATE SYNTHETASE A); carbamoyl-phosphate synthase (glutamine-hydrolyzing)	SAM G			0.81
At5g08260	eFP TAIR Neighbours	SCPL35 (serine carboxypeptidase-like 35); serine carboxypeptidase				0.81
At1g26910	eFP TAIR Neighbours	60S ribosomal protein L10 (RPL10B)	SAM G			0.81
At5g08160	eFP TAIR Neighbours	ATPK3 (Arabidopsis thaliana serine/threonine protein kinase 3); kinase	SAM G			0.81
At4g30810	eFP TAIR Neighbours	SCPL29 (serine carboxypeptidase-like 29); serine carboxypeptidase				0.81
At5g57490	eFP TAIR Neighbours	porin, putative	SAM G			0.81
At5g58250	eFP TAIR Neighbours	similar to hypothetical protein [Vitis vinifera] (GB:CAN75840.1); contains domain PD020337 (PD020337)	SAM G			0.81
At1g76160	eFP TAIR Neighbours	SKS5 (SKU5 Similar 5); copper ion binding / oxidoreductase		ABA Down		0.8
At2g20140	eFP TAIR Neighbours	26S protease regulatory complex subunit 4, putative				0.8
At3g05910	eFP TAIR Neighbours	pectinacetylesterase, putative	SAM G			0.8
At3g22780	eFP TAIR Neighbours	TSO1 (CHINESE FOR 'UGLY'); transcription factor	SAM G	ABA Up		0.8
At5g14750	eFP TAIR Neighbours	ATMYB66/WER/WER1 (WEREWOLF 1); DNA binding / protein binding / transcription factor/ transcription regulator			GA Up	0.8
At1g78060	eFP TAIR Neighbours	glycosyl hydrolase family 3 protein				0.8
At1g21880	eFP TAIR Neighbours	LYM1 (LYSM DOMAIN GPI-ANCHORED PROTEIN 1 PRECURSOR)	SAM G			0.8
At5g57440	eFP TAIR Neighbours	GS1 (GLYCEROL-3-PHOSPHATASE 2); hydrolase		ABA Down		0.8
At4g32400	eFP TAIR Neighbours	SHS1 (SODIUM HYPERSENSITIVE 1); binding / transporter	SAM G			0.8
At2g33330	eFP TAIR Neighbours	33 kDa secretory protein-related	SAM G			0.8
At1g33240	eFP TAIR Neighbours	AT-GTL1 (Arabidopsis thaliana GT2-like 1); transcription factor	SAM G	ABA Up		0.8
At1g74510	eFP TAIR Neighbours	kelch repeat-containing F-box family protein	SAM G			0.8
At2g25840	eFP TAIR Neighbours	OVA4 (OVULE ABORTION 4); ATP binding / aminoacyl-tRNA ligase	SAM G	ABA Down		0.8
At5g62680	eFP TAIR Neighbours	proton-dependent oligopeptide transport (POT) family protein	SAM G			0.8
At1g15000	eFP TAIR Neighbours	SCPL50 (serine carboxypeptidase-like 50); serine carboxypeptidase	SAM G			0.8
At1g29070	eFP TAIR Neighbours	ribosomal protein L34 family protein	SAM G	ABA Down		0.8
At1g60770	eFP TAIR Neighbours	pentatricopeptide (PPR) repeat-containing protein				0.8
At4g12650	eFP TAIR Neighbours	endomembrane protein 70, putative	SAM G			0.8
At4g21150	eFP TAIR Neighbours	ribophorin II (RPN2) family protein	SAM G			0.8
At4g16390	eFP TAIR Neighbours	chloroplastic RNA-binding protein P67, putative		ABA Down		0.8
At1g28395	eFP TAIR Neighbours	similar to unknown protein [Arabidopsis thaliana] (TAIR:AT2G33847.2); similar to unnamed protein product [Vitis vinifera] (GB:CAO39903.1)				0.8
At1g26770	eFP TAIR Neighbours	ATEXPA10 (ARABIDOPSIS THALIANA EXPANSIN A10)		ABA Up	GA Up	0.8
At3g48730	eFP TAIR Neighbours	GSA2 (GLUTAMATE-1-SEMIALDEHYDE 2,1-AMINOMUTASE 2); glutamate-1-semialdehyde 2,1-aminomutase	SAM G	ABA Down		0.8
At2g27840	eFP TAIR Neighbours	HDT4 (histone deacetylase 13)	SAM G	ABA Down		0.8
At2g37790	eFP TAIR Neighbours	aldo/keto reductase family protein				0.79
At3g53800	eFP TAIR Neighbours	armadillo/beta-catenin repeat family protein	SAM G			0.79
At2g14890	eFP TAIR Neighbours	AGP9 (ARABINOGALACTAN PROTEIN 9)				0.79
At1g44835	eFP TAIR Neighbours	YbaK/prolyl-tRNA synthetase family protein				0.79
At2g46420	eFP TAIR Neighbours	similar to unknown protein [Arabidopsis thaliana] (TAIR:AT3G61700.1); similar to unnamed protein product [Vitis vinifera] (GB:CAO39750.1); contains InterPro domain Conserved hypothetical protein CHP01589, plant (InterPro:IPR006476)	SAM G			0.79
At3g61770	eFP TAIR Neighbours	similar to unknown protein [Arabidopsis thaliana] (TAIR:AT1G67600.1); similar to unnamed protein product [Vitis vinifera] (GB:CAO14376.1); contains InterPro domain Acid phosphatase/vanadium-dependent haloperoxidase related (InterPro:IPR003832)	SAM G			0.79

At5g67070	eFP TAIR Neighbours	RALFL34 (RALF-LIKE 34)				0.79
At3g14790	eFP TAIR Neighbours	RHM3 (RHAMNOSE BIOSYNTHESIS 3); catalytic	SAM G			0.79
At5g25490	eFP TAIR Neighbours	zinc finger (Ran-binding) family protein	SAM G			0.79
At3g14240	eFP TAIR Neighbours	subtilase family protein	SAM G	ABA Up		0.79
At3g57560	eFP TAIR Neighbours	aspartate/glutamate/uridylate kinase family protein	SAM G			0.79
At5g19750	eFP TAIR Neighbours	peroxisomal membrane 22 kDa family protein	SAM G	ABA Down		0.79
At5g16870	eFP TAIR Neighbours	similar to unknown protein [Arabidopsis thaliana] (TAIR:AT3G03010.1); similar to unknown protein [Arabidopsis thaliana] (TAIR:AT3G03010.2); similar to hypothetical protein [Vitis vinifera] (GB:CAN83813.1); contains InterPro domain Peptidyl-tRNA hydrolase, PTH2 (InterPro:IPR002833)				0.79
At1g47670	eFP TAIR Neighbours	amino acid transporter family protein	SAM G	ABA Up		0.79
At5g27820	eFP TAIR Neighbours	ribosomal protein L18 family protein		ABA Down		0.79
At2g19670	eFP TAIR Neighbours	protein arginine N-methyltransferase, putative				0.79
At5g14660	eFP TAIR Neighbours	PDF1B (PEPTIDE DEFORMYLASE 1B); peptide deformylase				0.79
At5g57180	eFP TAIR Neighbours	CIA2 (CHLOROPLAST IMPORT APPARATUS 2)	SAM G	ABA Down		0.79
At1g70280	eFP TAIR Neighbours	NHL repeat-containing protein				0.79
At4g22250	eFP TAIR Neighbours	zinc finger (C3HC4-type RING finger) family protein				0.79
At3g27240	eFP TAIR Neighbours	cytochrome c1, putative	SAM G			0.79
At3g57490	eFP TAIR Neighbours	40S ribosomal protein S2 (RPS2D)				0.79
At5g63400	eFP TAIR Neighbours	ADK1 (ADENYLATE KINASE 1); adenylate kinase				0.79
At4g13170	eFP TAIR Neighbours	60S ribosomal protein L13A (RPL13aC)		ABA Down		0.79
At4g39280	eFP TAIR Neighbours	phenylalanyl-tRNA synthetase, putative / phenylalanine--tRNA ligase, putative	SAM G			0.79
At3g55400	eFP TAIR Neighbours	OVA1 (OVULE ABORTION 1); ATP binding / aminoacyl-tRNA ligase				0.79
At1g51730	eFP TAIR Neighbours	RWD domain-containing protein				0.79
At5g67220	eFP TAIR Neighbours	nitrogen regulation family protein				0.79
At4g24830	eFP TAIR Neighbours	arginosuccinate synthase family				0.79
At1g10522	eFP TAIR Neighbours	similar to unnamed protein product [Vitis vinifera] (GB:CAO40945.1)	SAM G			0.79
At4g34120	eFP TAIR Neighbours	LEJ1 (LOSS OF THE TIMING OF ET AND JA BIOSYNTHESIS 1)	SAM G	ABA Down		0.79
At3g62120	eFP TAIR Neighbours	tRNA synthetase class II (G, H, P and S) family protein	SAM G			0.79
At1g16870	eFP TAIR Neighbours	mitochondrial 28S ribosomal protein S29-related				0.79
At1g20950	eFP TAIR Neighbours	pyrophosphate--fructose-6-phosphate 1-phosphotransferase-related / pyrophosphate-dependent 6-phosphofructose-1-kinase-related				0.78
At1g10030	eFP TAIR Neighbours	ERG28 (ARABIDOPSIS HOMOLOG OF YEAST ERGOSTEROL28)	SAM G			0.78
At5g64290	eFP TAIR Neighbours	DCT/DIT2.1 (DICARBOXYLATE TRANSPORT); oxoglutarate:malate antiporter	SAM G			0.78
At2g01290	eFP TAIR Neighbours	ribose-5-phosphate isomerase	SAM G			0.78
At5g50740	eFP TAIR Neighbours	metal ion binding	SAM G			0.78
At1g55360	eFP TAIR Neighbours	similar to unknown protein [Arabidopsis thaliana] (TAIR:AT5G56530.1); similar to unknown protein [Arabidopsis thaliana] (TAIR:AT3G13510.1); similar to unnamed protein product [Vitis vinifera] (GB:CAO22094.1); similar to unknown [Populus trichocarpa] (GB:ABK93966.1); similar to hypothetical protein [Vitis vinifera] (GB:CAN72570.1); contains InterPro domain Protein of unknown function DUF239, plant (InterPro:IPR004314)	SAM G			0.78
At2g42770	eFP TAIR Neighbours	peroxisomal membrane 22 kDa family protein	SAM G	ABA Down		0.78
At5g13870	eFP TAIR Neighbours	EXGT-A4 (ENDOXYLOGLUCAN TRANSFERASE A4); hydrolase, acting on glycosyl bonds			GA Up	0.78
At5g65010	eFP TAIR Neighbours	ASN2 (ASPARAGINE SYNTHETASE 2); asparagine synthase (glutamine-hydrolyzing)				0.78

At1g55200	eFP TAIR Neighbours	protein kinase family protein				0.78
At1g18320 At3g10110	eFP TAIR Neighbours	[AT1G18320, mitochondrial import inner membrane translocase subunit Tim17/Tim22/Tim23 family protein];[AT3G10110, MEE67 (maternal effect embryo arrest 67); P-P-bond-hydrolysis-driven protein transmembrane transporter]				0.78
At2g21260 At2g21250	eFP TAIR Neighbours	[AT2G21260, mannose 6-phosphate reductase (NADPH-dependent), putative];[AT2G21250, mannose 6-phosphate reductase (NADPH-dependent), putative]				0.78
At1g78370	eFP TAIR Neighbours	ATGSTU20 (Arabidopsis thaliana Glutathione S-transferase (class tau) 20); glutathione transferase				0.78
At2g17280	eFP TAIR Neighbours	phosphoglycerate/bisphosphoglycerate mutase family protein	SAM G			0.78
At2g22420	eFP TAIR Neighbours	peroxidase 17 (PER17) (P17)	SAM G		GA Up	0.78
At3g61820	eFP TAIR Neighbours	aspartyl protease family protein	SAM G			0.78
At2g47390	eFP TAIR Neighbours	serine-type endopeptidase/ serine-type peptidase	SAM G			0.78
At5g21060	eFP TAIR Neighbours	homoserine dehydrogenase family protein	SAM G			0.78
At3g48700	eFP TAIR Neighbours	ATCXE13; hydrolase	SAM G			0.78
At3g18800	eFP TAIR Neighbours	similar to unnamed protein product [Vitis vinifera] (GB:CAO46487.1)				0.78
At4g14440	eFP TAIR Neighbours	enoyl-CoA hydratase/isomerase family protein		ABA Down		0.78
At3g07430	eFP TAIR Neighbours	EMB1990 (EMBRYO DEFECTIVE 1990)	SAM G			0.78
At4g24330	eFP TAIR Neighbours	similar to unknown protein [Arabidopsis thaliana] (TAIR:AT5G49945.1); similar to hypothetical protein [Vitis vinifera] (GB:CAN63262.1); contains InterPro domain Protein of unknown function DUF1682 (InterPro:IPR012879)	SAM G			0.78
At1g76400	eFP TAIR Neighbours	ribophorin I family protein		ABA Up		0.78
At3g11630	eFP TAIR Neighbours	2-cys peroxiredoxin, chloroplast (BAS1)				0.78
At3g47520	eFP TAIR Neighbours	MDH (MALATE DEHYDROGENASE); malate dehydrogenase	SAM G			0.78
At2g36170	eFP TAIR Neighbours	ubiquitin extension protein 2 (UBQ2) / 60S ribosomal protein L40 (RPL40A)				0.78
At5g53620	eFP TAIR Neighbours	similar to unnamed protein product [Vitis vinifera] (GB:CAO48590.1)	SAM G	ABA Up		0.78
At1g65290	eFP TAIR Neighbours	MTACP2 (MITOCHONDRIAL ACYL CARRIER PROTEIN 2); acyl carrier				0.78
At1g11680	eFP TAIR Neighbours	CYP51G1 (CYTOCHROME P450 51); oxygen binding				0.78
At5g28060	eFP TAIR Neighbours	40S ribosomal protein S24 (RPS24B)		ABA Down		0.78
At5g15650 At3g02230	eFP TAIR Neighbours	[AT5G15650, RGP2 (REVERSIBLY GLYCOSYLATED POLYPEPTIDE 2); alpha-1,4-glucan-protein synthase (UDP-forming)];[AT3G02230, RGP1 (REVERSIBLY GLYCOSYLATED POLYPEPTIDE 1)]				0.78
At4g10450	eFP TAIR Neighbours	60S ribosomal protein L9 (RPL90D)	SAM G			0.78
At5g25100	eFP TAIR Neighbours	endomembrane protein 70, putative	SAM G			0.78
At4g37910	eFP TAIR Neighbours	MTHSC70-1 (mitochondrial heat shock protein 70-1); ATP binding / unfolded protein binding	SAM G	ABA Up		0.78
At5g08300	eFP TAIR Neighbours	succinyl-CoA ligase (GDP-forming) alpha-chain, mitochondrial, putative / succinyl-CoA synthetase, alpha chain, putative / SCS-alpha, putative	SAM G			0.78
At4g26210	eFP TAIR Neighbours	mitochondrial ATP synthase g subunit family protein		ABA Down		0.78
At3g59650	eFP TAIR Neighbours	mitochondrial ribosomal protein L51/S25/CI-B8 family protein				0.78
At1g64860	eFP TAIR Neighbours	SIGA (SIGMA FACTOR A); DNA binding / DNA-directed RNA polymerase/ transcription factor	SAM G			0.77
At3g13560	eFP TAIR Neighbours	glycosyl hydrolase family 17 protein				0.77
At3g12930	eFP TAIR Neighbours	similar to unknown [Populus trichocarpa] (GB:ABK94112.1); contains InterPro domain Iojap-related protein (InterPro:IPR004394)	SAM G	ABA Down		0.77
At4g00820	eFP TAIR Neighbours	IQD17 (IQ-domain 17); calmodulin binding	SAM G			0.77
At4g08520 At3g09800	eFP TAIR Neighbours	[AT4G08520, clathrin adaptor complex small chain family protein];[AT3G09800, clathrin adaptor complex small chain family protein]				0.77
At2g24280	eFP TAIR Neighbours	serine carboxypeptidase S28 family protein				0.77

At1g64650	eFP TAIR Neighbours	similar to unknown protein [Arabidopsis thaliana] (TAIR:AT4G27720.1); similar to unknown protein [Arabidopsis thaliana] (TAIR:AT3G49310.1); similar to unknown [Populus trichocarpa] (GB:ABK93582.1); similar to unnamed protein product [Vitis vinifera] (GB:CAO48607.1); similar to unnamed protein product [Vitis vinifera] (GB:CAO62932.1); contains InterPro domain Protein of unknown function DUF791 (InterPro:IPR008509); contains InterPro domain MFS general substrate transporter (InterPro:IPR016196)	SAM G			0.77
At5g67280	eFP TAIR Neighbours	RLK (RECEPTOR-LIKE KINASE); ATP binding / kinase/ protein serine/threonine kinase	SAM G	ABA Up		0.77
At3g17790	eFP TAIR Neighbours	ATACP5 (acid phosphatase 5); acid phosphatase/ protein serine/threonine phosphatase	SAM G		GA Up	0.77
At4g31500	eFP TAIR Neighbours	CYP83B1 (CYTOCHROME P450 MONOOXYGENASE 83B1); oxygen binding	SAM G		GA Up	0.77
At1g53580	eFP TAIR Neighbours	ETHE1/GLX2-3/GLY3 (GLYOXALASE 2-3); hydroxyacylglutathione hydrolase	SAM G			0.77
At3g23700	eFP TAIR Neighbours	S1 RNA-binding domain-containing protein	SAM G			0.77
At2g32720	eFP TAIR Neighbours	B5 #4 (cytochrome b5 family protein #4); heme binding / transition metal ion binding				0.77
At3g61100	eFP TAIR Neighbours	similar to unknown protein [Arabidopsis thaliana] (TAIR:AT3G61090.1); similar to unnamed protein product [Vitis vinifera] (GB:CAO40196.1); contains InterPro domain Protein of unknown function DUF537 (InterPro:IPR007491)	SAM G			0.77
At2g28080	eFP TAIR Neighbours	glycosyltransferase family protein	SAM G	ABA Down		0.77
At5g51750	eFP TAIR Neighbours	ATSBT1.3; subtilase	SAM G		GA Up	0.77
At2g33180	eFP TAIR Neighbours	similar to unnamed protein product [Vitis vinifera] (GB:CAO41107.1)		ABA Down		0.77
At3g55420	eFP TAIR Neighbours	similar to hypothetical protein [Vitis vinifera] (GB:CAN83699.1)	SAM G			0.77
At3g04650	eFP TAIR Neighbours	oxidoreductase	SAM G	ABA Down		0.77
At2g36880	eFP TAIR Neighbours	MAT3 (METHIONINE ADENOSYLTRANSFERASE 3); methionine adenosyltransferase	SAM G			0.77
At5g38110	eFP TAIR Neighbours	ASF1B/SGA01/SGA1 (ANTI- SILENCING FUNCTION 1B)				0.77
At2g39870	eFP TAIR Neighbours	similar to unknown protein [Arabidopsis thaliana] (TAIR:AT3G55690.1); similar to unnamed protein product [Vitis vinifera] (GB:CAO69095.1)	SAM G	ABA Up		0.77
At4g34265	eFP TAIR Neighbours	similar to unknown protein [Arabidopsis thaliana] (TAIR:AT2G15000.3); similar to unknown protein [Arabidopsis thaliana] (TAIR:AT2G15000.2); similar to unnamed protein product [Vitis vinifera] (GB:CAO70855.1)				0.77
At4g17520	eFP TAIR Neighbours	nuclear RNA-binding protein, putative				0.77
At1g31970	eFP TAIR Neighbours	STRS1 (STRESS RESPONSE SUPPRESSOR 1); ATP-dependent helicase	SAM G			0.77
At5g13510	eFP TAIR Neighbours	ribosomal protein L10 family protein	SAM G	ABA Down		0.77
At5g56260	eFP TAIR Neighbours	dimethylmenaquinone methyltransferase family protein	SAM G	ABA Down		0.77
At5g45920	eFP TAIR Neighbours	carboxylesterase	SAM G	ABA Up		0.77
At1g34030	eFP TAIR Neighbours	40S ribosomal protein S18 (RPS18B)		ABA Down		0.77
At1g64090	eFP TAIR Neighbours	reticulon family protein (RTNLB3)	SAM G			0.77
At5g24610	eFP TAIR Neighbours	similar to unknown protein [Arabidopsis thaliana] (TAIR:AT3G49550.1); similar to hypothetical protein [Vitis vinifera] (GB:CAN64634.1)	SAM G			0.77
At3g11710	eFP TAIR Neighbours	lysyl-tRNA synthetase, putative / lysine--tRNA ligase, putative				0.77
At3g22300	eFP TAIR Neighbours	RPS10 (RIBOSOMAL PROTEIN S10); structural constituent of ribosome				0.77
At3g45310	eFP TAIR Neighbours	cysteine proteinase, putative				0.77
At1g53240	eFP TAIR Neighbours	malate dehydrogenase (NAD), mitochondrial				0.77
At5g60670	eFP TAIR Neighbours	60S ribosomal protein L12 (RPL12C)				0.77
At5g11340	eFP TAIR Neighbours	GCN5-related N-acetyltransferase (GNAT) family protein				0.77
At1g79560	eFP TAIR Neighbours	EMB1047/FTSH12 (EMBRYO DEFECTIVE 1047); ATP-dependent peptidase/ ATPase/ metallopeptidase	SAM G			0.77
At5g55280	eFP TAIR Neighbours	FTSZ1-1 (FtsZ1-1); structural molecule				0.77

At5g17920 At3g03780	eFP TAIR Neighbours	[AT5G17920, ATCIMS (COBALAMIN-INDEPENDENT METHIONINE SYNTHASE); 5-methyltetrahydropteroyltriglutamate-homocysteine S-methyltransferase/ methionine synthase];[AT3G03780, AtMS2 (Arabidopsis thaliana methionine synthase 2); 5-methyltetrahydropteroyltriglutamate-homocysteine S-methyltransferase]				0.77
At5g08280	eFP TAIR Neighbours	HEMC (HYDROXYMETHYLBILANE SYNTHASE); hydroxymethylbilane synthase	SAM G	ABA Down		0.77
At1g29980	eFP TAIR Neighbours	similar to unknown protein [Arabidopsis thaliana] (TAIR:AT2G34510.1); similar to unknown [Populus trichocarpa] (GB:ABK95079.1); contains InterPro domain Protein of unknown function DUF642 (InterPro:IPR006946); contains InterPro domain Galactose-binding like (InterPro:IPR008979)				0.77
At1g14670	eFP TAIR Neighbours	endomembrane protein 70, putative	SAM G			0.77
At2g33210	eFP TAIR Neighbours	chaperonin, putative	SAM G			0.77
At2g44650	eFP TAIR Neighbours	CHL-CPN10 (chloroplast chaperonin 10)	SAM G	ABA Down		0.77
At1g50900	eFP TAIR Neighbours	similar to hypothetical protein [Vitis vinifera] (GB:CAN65357.1); similar to unnamed protein product [Vitis vinifera] (GB:CAO38995.1); contains InterPro domain Ankyrin (InterPro:IPR002110)		ABA Down		0.76
At4g39690	eFP TAIR Neighbours	similar to unknown [Populus trichocarpa] (GB:ABK94999.1); contains domain PTHR15415 (PTHR15415)	SAM G			0.76
At2g24150	eFP TAIR Neighbours	HHP3 (heptahelical protein 3); receptor	SAM G			0.76
At1g30820	eFP TAIR Neighbours	CTP synthase, putative / UTP--ammonia ligase, putative	SAM G	ABA Up		0.76
At1g71500	eFP TAIR Neighbours	Rieske (2Fe-2S) domain-containing protein	SAM G			0.76
At1g28510	eFP TAIR Neighbours	similar to unknown protein [Arabidopsis thaliana] (TAIR:AT3G58150.1); similar to unnamed protein product [Vitis vinifera] (GB:CAO66379.1); contains InterPro domain Optic atrophy 3-like (InterPro:IPR010754)	SAM G			0.76
At2g37620 At3g53750	eFP TAIR Neighbours	[AT2G37620, ACT1 (ACTIN 1); structural constituent of cytoskeleton];[AT3G53750, ACT3 (ACTIN 3); structural constituent of cytoskeleton]				0.76
At4g04640	eFP TAIR Neighbours	ATPC1 (ATP synthase gamma chain 1)		ABA Down		0.76
At2g05790	eFP TAIR Neighbours	glycosyl hydrolase family 17 protein	SAM G			0.76
At5g57170	eFP TAIR Neighbours	macrophage migration inhibitory factor family protein / MIF family protein	SAM G			0.76
At4g38660	eFP TAIR Neighbours	thaumatin, putative				0.76
At5g44650	eFP TAIR Neighbours	similar to unnamed protein product [Vitis vinifera] (GB:CAO44475.1)				0.76
At5g09650	eFP TAIR Neighbours	ATPPA6 (ARABIDOPSIS THALIANA PYROPHOSPHORYLASE 6); inorganic diphosphatase/ pyrophosphatase				0.76
At5g18460	eFP TAIR Neighbours	similar to unknown protein [Arabidopsis thaliana] (TAIR:AT1G23340.1); similar to unknown protein [Arabidopsis thaliana] (TAIR:AT1G23340.2); similar to unnamed protein product [Vitis vinifera] (GB:CAO38766.1); contains InterPro domain Protein of unknown function DUF239, plant (InterPro:IPR004314)	SAM G			0.76
At3g26060	eFP TAIR Neighbours	ATPRX Q; antioxidant/ peroxiredoxin	SAM G	ABA Down		0.76
At5g40160	eFP TAIR Neighbours	EMB506 (EMBRYO DEFECTIVE 506); protein binding				0.76
At4g32915	eFP TAIR Neighbours	glutamyl-tRNA(Gln) amidotransferase	SAM G	ABA Down		0.76
At2g39890	eFP TAIR Neighbours	ProT1 (PROLINE TRANSPORTER 1); amino acid transmembrane transporter				0.76
At1g33040	eFP TAIR Neighbours	NACA5 (NASCENT POLYPEPTIDE-ASSOCIATED COMPLEX SUBUNIT ALPHA-LIKE PROTEIN 5)				0.76
At2g26900	eFP TAIR Neighbours	bile acid:sodium symporter family protein	SAM G			0.76
At4g37510	eFP TAIR Neighbours	ribonuclease III family protein				0.76
At2g38120	eFP TAIR Neighbours	AUX1 (AUXIN RESISTANT 1); amino acid transmembrane transporter/ transporter	SAM G	ABA Down	GA Up	0.76
At2g40010	eFP TAIR Neighbours	60S acidic ribosomal protein P0 (RPP0A)				0.76
At3g04760	eFP TAIR Neighbours	pentatricopeptide (PPR) repeat-containing protein	SAM G	ABA Down		0.76
At2g25830	eFP TAIR Neighbours	YebC-related				0.76
At5g65310	eFP TAIR Neighbours	ATHB5 (ARABIDOPSIS THALIANA HOMEOBOX PROTEIN 5); transcription factor	SAM G			0.76

At1g20050	eFP TAIR Neighbours	HYD1 (Hydra 1)	SAM G			0.76
At2g04030	eFP TAIR Neighbours	CR88 (EMBRYO DEFECTIVE 1956); ATP binding	SAM G			0.76
At5g17630	eFP TAIR Neighbours	glucose-6-phosphate/phosphate translocator, putative	SAM G			0.76
At3g55410	eFP TAIR Neighbours	2-oxoglutarate dehydrogenase E1 component, putative / oxoglutarate decarboxylase, putative / alpha-ketoglutaric dehydrogenase, putative	SAM G			0.76
At3g63410	eFP TAIR Neighbours	APG1 (ALBINO OR PALE GREEN MUTANT 1); methyltransferase				0.76
At5g17560	eFP TAIR Neighbours	BolA-like family protein	SAM G			0.76
At1g13320	eFP TAIR Neighbours	PP2AA3/PR65 (PROTEIN PHOSPHATASE 2A SUBUNIT A3); protein phosphatase type 2A regulator	SAM G	ABA Up		0.76
At4g30930	eFP TAIR Neighbours	NFD1 (NUCLEAR FUSION DEFECTIVE 1); structural constituent of ribosome				0.76
At3g20330	eFP TAIR Neighbours	aspartate carabmoyltransferase, chloroplast / aspartate transcarbamylase / ATCase (PYRB)				0.76
At4g35000	eFP TAIR Neighbours	APX3 (ASCORBATE PEROXIDASE 3); L-ascorbate peroxidase		ABA Down		0.76
At5g64030	eFP TAIR Neighbours	dehydration-responsive protein-related	SAM G			0.76
At3g20390	eFP TAIR Neighbours	endoribonuclease L-PSP family protein	SAM G			0.76
At3g04770	eFP TAIR Neighbours	RPSAB (40S RIBOSOMAL PROTEIN SA B); structural constituent of ribosome	SAM G			0.76
At5g14450	eFP TAIR Neighbours	GDSL-motif lipase/hydrolase family protein				0.76
At5g08530	eFP TAIR Neighbours	CI51 (51 KDA SUBUNIT OF COMPLEX I); NADH dehydrogenase (ubiquinone)				0.76
At4g25890	eFP TAIR Neighbours	60S acidic ribosomal protein P3 (RPP3A)				0.76
At3g01540	eFP TAIR Neighbours	DRH1 (DEAD box RNA helicase 1)	SAM G			0.76
At4g12600	eFP TAIR Neighbours	ribosomal protein L7Ae/L30e/S12e/Gadd45 family protein				0.76
At4g34290	eFP TAIR Neighbours	SWIB complex BAF60b domain-containing protein				0.76
At3g23990	eFP TAIR Neighbours	HSP60 (Heat shock protein 60); ATP binding / protein binding / unfolded protein binding				0.76
At2g44050	eFP TAIR Neighbours	COS1 (COI1 SUPPRESSOR1); 6,7-dimethyl-8-ribityllumazine synthase	SAM G			0.76
At5g57290	eFP TAIR Neighbours	60S acidic ribosomal protein P3 (RPP3B)				0.76
At5g63510	eFP TAIR Neighbours	GAMMA CAL1 (GAMMA CARBONIC ANHYDRASE LIKE 1); carbonate dehydratase				0.76
At5g61170	eFP TAIR Neighbours	40S ribosomal protein S19 (RPS19C)				0.76
At3g54090	eFP TAIR Neighbours	pfkB-type carbohydrate kinase family protein				0.76
At1g60090	eFP TAIR Neighbours	glycosyl hydrolase family 1 protein	SAM G			0.75
At4g03210	eFP TAIR Neighbours	XTH9 (XYLOGLUCAN ENDOTRANSGLUCOSYLASE/HYDROLASE 9); hydrolase, acting on glycosyl bonds		ABA Down	GA Up	0.75
At3g16950	eFP TAIR Neighbours	LPD1 (LIPOAMIDE DEHYDROGENASE 1)				0.75
At1g10200	eFP TAIR Neighbours	WLIM1; transcription factor		ABA Down		0.75
At1g66150	eFP TAIR Neighbours	TMK1 (TRANSMEMBRANE KINASE 1)				0.75
At1g47960	eFP TAIR Neighbours	C/VIF1 (CELL WALL / VACUOLAR INHIBITOR OF FRUCTOSIDASE 1); pectinesterase inhibitor		ABA Down		0.75
At4g34980	eFP TAIR Neighbours	SLP2 (subtilisin-like serine protease 2); subtilase	SAM G	ABA Up	GA Up	0.75
At5g10240	eFP TAIR Neighbours	ASN3 (ASPARAGINE SYNTHETASE 3); asparagine synthase (glutamine-hydrolyzing)	SAM G	ABA Up		0.75
At1g23080	eFP TAIR Neighbours	PIN7 (PIN-FORMED 7); auxin:hydrogen symporter/ transporter	SAM G		GA Up	0.75
At4g34760	eFP TAIR Neighbours	auxin-responsive family protein	SAM G	ABA Up		0.75
At1g21740	eFP TAIR Neighbours	similar to unknown protein [Arabidopsis thaliana] (TAIR:AT1G77500.1); similar to hypothetical protein [Vitis vinifera] (GB:CAN81539.1); contains InterPro domain Protein of unknown function DUF632 (InterPro:IPR006867); contains InterPro domain Protein of unknown function DUF630 (InterPro:IPR006868)	SAM G	ABA Up		0.75
At2g20515	eFP TAIR Neighbours	similar to unnamed protein product [Vitis vinifera] (GB:CAO40634.1)				0.75
At4g30330	eFP TAIR Neighbours	small nuclear ribonucleoprotein E, putative / snRNP-E, putative / Sm protein E, putative		ABA Down		0.75
At5g65700	eFP TAIR Neighbours	BAM1 (big apical meristem 1); ATP binding / kinase/ protein serine/threonine kinase				0.75

At5g19520	eFP TAIR Neighbours	mechanosensitive ion channel domain-containing protein / MS ion channel domain-containing protein	SAM G			0.75
At2g20610	eFP TAIR Neighbours	SUR1 (SUPERROOT 1); transaminase	SAM G	ABA Up	GA Up	0.75
At2g37660	eFP TAIR Neighbours	binding / catalytic/ coenzyme binding		ABA Down		0.75
At1g10760	eFP TAIR Neighbours	SEX1 (STARCH EXCESS 1)				0.75
At1g50010	eFP TAIR Neighbours	TUA2 (tubulin alpha-2 chain)				0.75
At5g65650	eFP TAIR Neighbours	similar to unknown protein [Arabidopsis thaliana] (TAIR:AT4G36660.1); similar to unknown [Populus trichocarpa] (GB:ABK93535.1); contains InterPro domain Protein of unknown function DUF1195 (InterPro:IPR010608)	SAM G	ABA Down		0.75
At2g38810	eFP TAIR Neighbours	HTA8; DNA binding				0.75
At1g26740	eFP TAIR Neighbours	structural constituent of ribosome				0.75
At4g36180	eFP TAIR Neighbours	leucine-rich repeat family protein	SAM G	ABA Up		0.75
At5g25020 At5g24990	eFP TAIR Neighbours	[AT5G25020, similar to unknown protein [Arabidopsis thaliana] (TAIR:AT5G24990.1); similar to hypothetical protein [Vitis vinifera] (GB:CAN83078.1); contains InterPro domain Protein of unknown function DUF1336 (InterPro:IPR009769)];[AT5G24990, similar to unknown protein [Arabidopsis thaliana] (TAIR:AT5G25010.1); similar to hypothetical protein [Vitis vinifera] (GB:CAN83078.1); contains InterPro domain Protein of unknown function DUF1336 (InterPro:IPR009769)]				0.75
At3g63200	eFP TAIR Neighbours	PLA IIIB/PLP9 (Patatin-like protein 9); nutrient reservoir				0.75
At3g27230	eFP TAIR Neighbours	similar to (ARABIDOPSIS THALIANA RAS ASSOCIATED WITH DIABETES PROTEIN 3), methyltransferase [Arabidopsis thaliana] (TAIR:AT5G40830.1); similar to (ARABIDOPSIS THALIANA RAS ASSOCIATED WITH DIABETES PROTEIN 3), methyltransferase [Arabidopsis thaliana] (TAIR:AT5G40830.2); similar to hypothetical protein [Prunus persica] (GB:AAO14627.1); contains domain SSF53335 (SSF53335)	SAM G	ABA Down		0.75
At1g09390	eFP TAIR Neighbours	GDSL-motif lipase/hydrolase family protein				0.75
At5g15520	eFP TAIR Neighbours	40S ribosomal protein S19 (RPS19B)		ABA Down		0.75
At2g23350	eFP TAIR Neighbours	PAB4 (POLY(A) BINDING PROTEIN 4); RNA binding / translation initiation factor				0.75
At4g32390	eFP TAIR Neighbours	phosphate translocator-related	SAM G			0.75
At5g27470	eFP TAIR Neighbours	seryl-tRNA synthetase / serine--tRNA ligase				0.75
At3g04120	eFP TAIR Neighbours	GAPC (GLYCERALDEHYDE-3-PHOSPHATE DEHYDROGENASE C SUBUNIT); glyceraldehyde-3-phosphate dehydrogenase				0.75
At3g52930	eFP TAIR Neighbours	fructose-bisphosphate aldolase, putative				0.75
At1g47260	eFP TAIR Neighbours	APFI; carbonate dehydratase		ABA Down		0.75
At2g32920	eFP TAIR Neighbours	ATPDIL2-3 (PDI-LIKE 2-3); protein disulfide isomerase		ABA Down		0.75
At3g20820	eFP TAIR Neighbours	leucine-rich repeat family protein	SAM G			0.75
At3g03250	eFP TAIR Neighbours	UGP (UDP-glucose pyrophosphorylase); UTP:glucose-1-phosphate uridylyltransferase	SAM G	ABA Down		0.75
At3g13580	eFP TAIR Neighbours	60S ribosomal protein L7 (RPL7D)				0.75
At4g02930	eFP TAIR Neighbours	elongation factor Tu, putative / EF-Tu, putative				0.75
At2g33840 At1g28350	eFP TAIR Neighbours	[AT2G33840, tRNA synthetase class I (W and Y) family protein];[AT1G28350, ATP binding / aminoacyl-tRNA ligase]				0.75
At2g16600	eFP TAIR Neighbours	ROC3 (rotamase CyP 3); peptidyl-prolyl cis-trans isomerase				0.75
At4g28440	eFP TAIR Neighbours	DNA-binding protein-related				0.75
At4g26300	eFP TAIR Neighbours	EMB1027 (EMBRYO DEFECTIVE 1027); ATP binding / arginine-tRNA ligase	SAM G			0.75
At1g05190	eFP TAIR Neighbours	EMB2394 (EMBRYO DEFECTIVE 2394); structural constituent of ribosome		ABA Down		0.75
At4g28210	eFP TAIR Neighbours	EMB1923 (EMBRYO DEFECTIVE 1923)	SAM G			0.75
At1g75330	eFP TAIR Neighbours	OTC (ORNITHINE CARBAMOYLTRANSFERASE); amino acid binding / carboxyl- or carbamoyltransferase				0.75
At2g40660	eFP TAIR Neighbours	tRNA-binding region domain-containing protein				0.75
At4g02380	eFP TAIR Neighbours	SAG21 (SENESCENCE-ASSOCIATED GENE 21)		ABA Down		0.75

At3g55010	eFP TAIR Neighbours	ATPURM/PUR5; phosphoribosylformylglycinamidine cyclo-ligase				0.75
At2g18040	eFP TAIR Neighbours	PIN1AT (parvulin 1At)				0.75
At5g40200	eFP TAIR Neighbours	DEGP9 (DEGP PROTEASE 9); serine-type peptidase/ trypsin	SAM G			0.75
At2g45740	eFP TAIR Neighbours	PEX11D	SAM G			0.75
At5g24650	eFP TAIR Neighbours	mitochondrial import inner membrane translocase subunit Tim17/Tim22/Tim23 family protein	SAM G			0.75
At3g15190	eFP TAIR Neighbours	chloroplast 30S ribosomal protein S20, putative		ABA Down		0.75
At5g19290	eFP TAIR Neighbours	esterase/lipase/thioesterase family protein	SAM G	ABA Down		0.75

Candidate gene *At5g10920* as identified on IL 4-3 putatively associated with glycolysis and correlated genes as generated by SeedNet available on http://vseednet.nottingham.ac.uk. The candidate gene is involved in Arg biosynthesis. The correlated genes are supplied with the Pearson correlation coefficient values. Correlated genes of relevance to glycolysis are highlighted in grey. Localization of gene candidates was achieved by utilizing data as analyzed on dry IL seeds of harvest seasons I and II in Akko, Israel.
