# Supplementary material for: Metabolic Profiling of a Mapping Population Exposes New Insights in the Regulation of Seed Metabolism and Seed, Fruit, and Plant Relations
Source: PLoS Genet. 2012 Mar 29;8(3):e1002612. doi: 10.1371/journal.pgen.1002612 (PMC3315483; doi:10.1371/journal.pgen.1002612)
Supplement: Table S3 — Candidate gene At5g63840 and its correlated genes associated with glycolysis on IL 4-3-2. Candidate gene At5g63840 as identified on IL 4-3-2 putatively associated with glycolysis and co-predicted genes as generated by SCoPNET available on http://vseednet.nottingham.ac.uk. The candidate gene is involved in cellulose biosynthetic processes with glucosidase and hydrolase activities. The co-predicted genes are supplied with the co-prediction PMI values. Co-predicted genes of relevance to glycolysis are highlighted in grey. Localization of gene candidates was achieved by utilizing data as analyzed on dry IL seeds of harvest seasons I and II in Akko, Israel. (PDF) [file pgen.1002612.s012.pdf]

SCoPNET Co-Prediction Table S3. Candidate gene *At5g63840* and its correlated genes associated with Glycolysis on IL 4-3-2.

AGI	Links	Annotation	ABA Regulation	GA Regulation	Dormancy Node Strength	Germination Node Strength	PMI score
At5g67320	eFP TAIR Neighbours	WD-40 repeat family protein			1	3	7.38
At5g17790	eFP TAIR Neighbours	VAR3 (VARIEGATED 3); binding			0	4	7.38
At5g27210	eFP TAIR Neighbours	similar to transmembrane protein, putative [Arabidopsis thaliana] (TAIR:AT3G05010.1); similar to unnamed protein product [Vitis vinifera] (GB:CAO15118.1)	Col ABA UP		3	3	6.98
At2g26930	eFP TAIR Neighbours	ATCDPMEK (PIGMENT DEFECTIVE 277); 4-(cytidine 5'-diphospho)-2-C-methyl-D-erythritol kinase			3	5	6.69
At4g01790	eFP TAIR Neighbours	ribosomal protein L7Ae/L30e/S12e/Gadd45 family protein / ribonuclease P-related			2	6	6.69
At5g01400	eFP TAIR Neighbours	ESP4 (ENHANCED SILENCING PHENOTYPE 4); binding	Col ABA UP		4	4	6.69
At1g69450	eFP TAIR Neighbours	similar to HYP1 (HYPOTHETICAL PROTEIN 1) [Arabidopsis thaliana] (TAIR:AT3G01100.1); similar to unnamed protein product [Vitis vinifera] (GB:CAO64743.1); similar to unnamed protein product [Vitis vinifera] (GB:CAO15025.1); similar to Protein of unknown function DUF221 [Medicago truncatula] (GB:ABN08272.1); contains InterPro domain Protein of unknown function DUF221; (InterPro:IPR003864)			6	3	6.57
At1g57870	eFP TAIR Neighbours	shaggy-related protein kinase kappa, putative / ASK-kappa, putative			7	3	6.47
At3g49320	eFP TAIR Neighbours	similar to unknown protein [Arabidopsis thaliana] (TAIR:AT5G41970.1); similar to unnamed protein product [Vitis vinifera] (GB:CAO62930.1); contains InterPro domain Metal-dependent protein hydrolase (InterPro:IPR003226)			0	10	6.47
At3g52230	eFP TAIR Neighbours	similar to unknown [Populus trichocarpa] (GB:ABK93315.1)		GA Down	4	6	6.47
At2g47240	eFP TAIR Neighbours	long-chain-fatty-acid--CoA ligase family protein / long-chain acyl-CoA synthetase family protein			0	11	6.37
At3g10810	eFP TAIR Neighbours	zinc finger (C3HC4-type RING finger) family protein	Col ABA UP		1	10	6.37
At5g24740	eFP TAIR Neighbours	similar to unknown protein [Arabidopsis thaliana] (TAIR:AT4G17140.1); similar to hypothetical protein OsI_026627 [Oryza sativa (indica cultivar-group)] (GB:EAZ05395.1); similar to C-5 cytosine-specific DNA methylase; Vacuolar protein sorting-associated protein [Medicago truncatula] (GB:ABN08303.1); similar to hypothetical protein OsJ_024797 [Oryza sativa (japonica cultivar-group)] (GB:EAZ41314.1); contains InterPro domain Vacuolar protein sorting-associated protein; (InterPro:IPR009543)	Col ABA UP		9	2	6.37
At4g33040	eFP TAIR Neighbours	glutaredoxin family protein			1	11	6.29
At5g13980	eFP TAIR Neighbours	glycosyl hydrolase family 38 protein			5	7	6.29
At1g49400	eFP TAIR Neighbours	EMB1129 (EMBRYO DEFECTIVE 1129); structural constituent of ribosome			0	13	6.21
At3g03210	eFP TAIR Neighbours	similar to unnamed protein product [Vitis vinifera] (GB:CAO22195.1)			0	14	6.13
At1g19520	eFP TAIR Neighbours	NFD5 (NUCLEAR FUSION DEFECTIVE 5); transcription factor			0	17	5.94
At5g15350	eFP TAIR Neighbours	plastocyanin-like domain-containing protein	Col ABA Down	GA Up	0	22	5.68
At3g18130	eFP TAIR Neighbours	guanine nucleotide-binding family protein / activated protein kinase C receptor (RACK1)			1	27	5.44
At5g38830	eFP TAIR Neighbours	tRNA synthetase class I (C) family protein			0	28	5.44
At3g21700	eFP TAIR Neighbours	GTP binding	Col ABA UP		0	29	5.4
At5g48300	eFP TAIR Neighbours	ADG1 (ADP GLUCOSE PYROPHOSPHORYLASE SMALL SUBUNIT 1); glucose-1-phosphate adenylyltransferase			0	29	5.4
At1g69526	eFP TAIR Neighbours	UbiE/COQ5 methyltransferase family protein	Col ABA Down		22	11	5.27
At1g10170	eFP TAIR Neighbours	ATNFXL1; transcription factor	Col ABA UP		8	33	5.06
At2g07707_ AtMg00480	eFP TAIR Neighbours				1	44	4.96
At5g56860	eFP TAIR Neighbours	GNC (GATA, NITRATE-INDUCIBLE, CARBON METABOLISM-INVOLVED); transcription factor			0	45	4.96
At2g19860	eFP TAIR Neighbours	ATHXK2 (HEXOKINASE 2); ATP binding / hexokinase			0	47	4.92
At5g13220	eFP TAIR Neighbours	JAS1/JAZ10/TIFY9 (JASMONATE-ZIM-DOMAIN PROTEIN 10)			0	48	4.9

At1g21740	eFP TAIR Neighbours	similar to unknown protein [Arabidopsis thaliana] (TAIR:AT1G77500.1); similar to hypothetical protein [Vitis vinifera] (GB:CAN81539.1); contains InterPro domain Protein of unknown function DUF632 (InterPro:IPR006867); contains InterPro domain Protein of unknown function DUF630 (InterPro:IPR006868)	Col ABA UP		0	50	4.86
At5g65310	eFP TAIR Neighbours	ATHB5 (ARABIDOPSIS THALIANA HOMEOBOX PROTEIN 5); transcription factor			0	81	4.38
At3g10530	eFP TAIR Neighbours	transducin family protein / WD-40 repeat family protein			4	88	4.25
At3g50340	eFP TAIR Neighbours	similar to unknown protein [Arabidopsis thaliana] (TAIR:AT5G67020.1); similar to unknown protein [Oryza sativa (japonica cultivar-group)] (GB:BAD09363.1)	Col ABA UP		0	127	3.93
At4g14680	eFP TAIR Neighbours	APS3 (ATP sulfurylase 2); sulfate adenylyltransferase (ATP)			1	170	3.63
At1g21880	eFP TAIR Neighbours	LYM1 (LYSM DOMAIN GPI-ANCHORED PROTEIN 1 PRECURSOR)			0	208	3.43
At4g05440	eFP TAIR Neighbours	EDA35 (embryo sac development arrest 35)			0	370	2.86
At3g54430	eFP TAIR Neighbours	SRS6 (SHI-RELATED SEQUENCE 6)			0	488	2.58

Candidate gene *At5g63840* as identified on IL 4-3-2 putatively associated with glycolysis and copredicted genes as generated by SCoPNET available on http://vseednet.nottingham.ac.uk. The candidate gene is involved in cellulose biosynthetic processes with a glucosidase and hydrolase activity. The copredicted genes are supplied with the coprediction PMI values. Copredicted genes of relevance to glycolysis are highlighted in grey. Localization of gene candidates was achieved by utilizing data as analyzed on dry IL seeds of harvest seasons I and II in Akko, Israel.
