# Supplementary material for: Metabolic Profiling of a Mapping Population Exposes New Insights in the Regulation of Seed Metabolism and Seed, Fruit, and Plant Relations
Source: PLoS Genet. 2012 Mar 29;8(3):e1002612. doi: 10.1371/journal.pgen.1002612 (PMC3315483; doi:10.1371/journal.pgen.1002612)
Supplement: Table S4 — Candidate gene At1g14810 and its correlated genes associated with organic acid and hexose sugars on IL 1-1-3. Candidate gene At1g14810 as identified on IL 1-1-3 putatively associated with organic acid and hexose sugars and correlated genes as generated by SeedNet available on http://vseednet.nottingham.ac.uk. The candidate gene codes for an aspartate semialdeyhde dehydrogenase. The co-predicted genes are supplied with Pearson's coefficient values. Correlated genes of relevance to glycolysis are highlighted in grey. Localization of gene candidates was achieved by utilizing data as analyzed on dry IL seeds of harvest seasons I and II in Akko, Israel. (PDF) [file pgen.1002612.s013.pdf]

SeedNet Correlation Table S4. Candidate gene At1g14810 and its correlated genes associated with organic acid and hexose sugars on IL 1-1-3.

AGI	Links	Annotation	SAM NG/G	ABA Regulation	GA Regulation	Correlation
At3g18130	eFP TAIR Neighbours	guanine nucleotide-binding family protein / activated protein kinase C receptor (RACK1)				0.92
At1g63660	eFP TAIR Neighbours	GMP synthase (glutamine-hydrolyzing), putative / glutamine amidotransferase, putative				0.91
At1g48630	eFP TAIR Neighbours	guanine nucleotide-binding family protein / activated protein kinase C receptor, putative / RACK, putative				0.91
At4g39880	eFP TAIR Neighbours	ribosomal protein L23 family protein	SAM G			0.9
At4g30800	eFP TAIR Neighbours	40S ribosomal protein S11 (RPS11B)	SAM G			0.9
At3g16780	eFP TAIR Neighbours	60S ribosomal protein L19 (RPL19B)				0.9
At1g56050	eFP TAIR Neighbours	GTP-binding protein-related	SAM G			0.9
At4g16390	eFP TAIR Neighbours	chloroplastic RNA-binding protein P67, putative		ABA Down		0.9
At5g26710	eFP TAIR Neighbours	glutamate-tRNA ligase, putative / glutamyl-tRNA synthetase, putatuve / GluRS, putative	SAM G			0.9
At3g57610	eFP TAIR Neighbours	ATPURA; adenylosuccinate synthase	SAM G			0.89
At1g13270	eFP TAIR Neighbours	MAP1C (METHIONINE AMINOPEPTIDASE 1B); metalloexopeptidase	SAM G	ABA Down		0.89
At5g50370	eFP TAIR Neighbours	adenylate kinase, putative				0.89
At4g33680	eFP TAIR Neighbours	AGD2 (ABERRANT GROWTH AND DEATH 2); transaminase	SAM G			0.89
At1g68590	eFP TAIR Neighbours	plastid-specific 30S ribosomal protein 3, putative / PSRP-3, putative				0.88
At1g17200	eFP TAIR Neighbours	integral membrane family protein	SAM G			0.88
At4g30810	eFP TAIR Neighbours	SCPL29 (serine carboxypeptidase-like 29); serine carboxypeptidase				0.88
At1g31860	eFP TAIR Neighbours	AT-IE (Arabidopsis thaliana bifunctional HisI-HisE protein)				0.88
At3g44590	eFP TAIR Neighbours	60S acidic ribosomal protein P2 (RPP2D)		ABA Down		0.87
At2g37640	eFP TAIR Neighbours	ATEXPA3 (ARABIDOPSIS THALIANA EXPANSIN A3)			GA Up	0.87
At4g13170	eFP TAIR Neighbours	60S ribosomal protein L13A (RPL13aC)		ABA Down		0.87
At1g34030	eFP TAIR Neighbours	40S ribosomal protein S18 (RPS18B)		ABA Down		0.87
At5g11880 At3g14390	eFP TAIR Neighbours	[AT5G11880, diaminopimelate decarboxylase, putative / DAP carboxylase, putative];[AT3G14390, diaminopimelate decarboxylase, putative / DAP carboxylase, putative]				0.87
At1g12800	eFP TAIR Neighbours	S1 RNA-binding domain-containing protein	SAM G	ABA Down		0.87
At1g26770	eFP TAIR Neighbours	ATEXPA10 (ARABIDOPSIS THALIANA EXPANSIN A10)		ABA Up	GA Up	0.87
At5g67070	eFP TAIR Neighbours	RALFL34 (RALF-LIKE 34)				0.86
At1g30820	eFP TAIR Neighbours	CTP synthase, putative / UTP--ammonia ligase, putative	SAM G	ABA Up		0.86
At3g61770	eFP TAIR Neighbours	similar to unknown protein [Arabidopsis thaliana] (TAIR:AT1G67600.1); similar to unnamed protein product [Vitis vinifera] (GB:CAO14376.1); contains InterPro domain Acid phosphatase/vanadium-dependent haloperoxidase related (InterPro:IPR003832)	SAM G			0.86
At4g30620	eFP TAIR Neighbours	similar to unknown protein [Arabidopsis thaliana] (TAIR:AT2G24020.1); similar to unknown [Picea sitchensis] (GB:ABK26000.1); similar to Os02g0180200 [Oryza sativa (japonica cultivar-group)] (GB:NP_001046090.1); contains InterPro domain Conserved hypothetical protein CHP00103 (InterPro:IPR004401)	SAM G			0.86
At5g27470	eFP TAIR Neighbours	seryl-tRNA synthetase / serine--tRNA ligase				0.86
At3g11710	eFP TAIR Neighbours	lysyl-tRNA synthetase, putative / lysine--tRNA ligase, putative				0.86
At2g16430	eFP TAIR Neighbours	ATPAP10/PAP10; protein serine/threonine phosphatase				0.85
At5g63810	eFP TAIR Neighbours	BGAL10 (beta-galactosidase 10); beta-galactosidase	SAM G			0.85
At2g33210	eFP TAIR Neighbours	chaperonin, putative	SAM G			0.85
At1g60160	eFP TAIR Neighbours	potassium transporter family protein	SAM G			0.85
At1g28395	eFP TAIR Neighbours	similar to unknown protein [Arabidopsis thaliana] (TAIR:AT2G33847.2); similar to unnamed protein product [Vitis vinifera] (GB:CAO39903.1)				0.85
At5g46580	eFP TAIR Neighbours	pentatricopeptide (PPR) repeat-containing protein	SAM G			0.85
At4g26760	eFP TAIR Neighbours	microtubule associated protein (MAP65/ASE1) family protein	SAM G			0.85

At5g67260	eFP TAIR Neighbours	CYCD3;2 (CYCLIN D3;2); cyclin-dependent protein kinase		ABA Down		0.85
At1g26910	eFP TAIR Neighbours	60S ribosomal protein L10 (RPL10B)	SAM G			0.85
At2g22420	eFP TAIR Neighbours	peroxidase 17 (PER17) (P17)	SAM G		GA Up	0.85
At5g10920	eFP TAIR Neighbours	argininosuccinate lyase, putative / arginosuccinase, putative	SAM G			0.85
At5g67280	eFP TAIR Neighbours	RLK (RECEPTOR-LIKE KINASE); ATP binding / kinase/ protein serine/threonine kinase	SAM G	ABA Up		0.85
At3g49470	eFP TAIR Neighbours	NACA2 (NASCENT POLYPEPTIDE-ASSOCIATED COMPLEX SUBUNIT ALPHA-LIKE PROTEIN 2)				0.85
At2g36170	eFP TAIR Neighbours	ubiquitin extension protein 2 (UBQ2) / 60S ribosomal protein L40 (RPL40A)				0.85
At5g20950	eFP TAIR Neighbours	glycosyl hydrolase family 3 protein		ABA Up		0.85
At5g11340	eFP TAIR Neighbours	GCN5-related N-acetyltransferase (GNAT) family protein				0.85
At2g27840	eFP TAIR Neighbours	HDT4 (histone deacetylase 13)	SAM G	ABA Down		0.85
At5g24650	eFP TAIR Neighbours	mitochondrial import inner membrane translocase subunit Tim17/Tim22/Tim23 family protein	SAM G			0.85
At1g33040	eFP TAIR Neighbours	NACA5 (NASCENT POLYPEPTIDE-ASSOCIATED COMPLEX SUBUNIT ALPHA-LIKE PROTEIN 5)				0.85
At1g55490	eFP TAIR Neighbours	CPN60B (CHAPERONIN 60 BETA); ATP binding / protein binding / unfolded protein binding		ABA Down		0.85
At4g26210	eFP TAIR Neighbours	mitochondrial ATP synthase g subunit family protein		ABA Down		0.84
At2g29980	eFP TAIR Neighbours	FAD3 (FATTY ACID DESATURASE 3); omega-3 fatty acid desaturase				0.84
At1g31180 At5g14200	eFP TAIR Neighbours	[AT1G31180, 3-isopropylmalate dehydrogenase, chloroplast, putative];[AT5G14200, 3-isopropylmalate dehydrogenase, chloroplast, putative]				0.84
At3g49680	eFP TAIR Neighbours	ATBCAT-3/BCAT3 (BRANCHED-CHAIN AMINOTRANSFERASE 3); branched-chain-amino-acid transaminase/ catalytic	SAM G	ABA Down		0.84
At3g47520	eFP TAIR Neighbours	MDH (MALATE DEHYDROGENASE); malate dehydrogenase	SAM G			0.84
At3g16480	eFP TAIR Neighbours	MPPALPHA (mitochondrial processing peptidase alpha subunit); metalloendopeptidase	SAM G			0.84
At1g51730	eFP TAIR Neighbours	RWD domain-containing protein				0.84
At4g35000	eFP TAIR Neighbours	APX3 (ASCORBATE PEROXIDASE 3); L-ascorbate peroxidase		ABA Down		0.84
At4g35850	eFP TAIR Neighbours	pentatricopeptide (PPR) repeat-containing protein	SAM G			0.84
At5g17560	eFP TAIR Neighbours	BolA-like family protein	SAM G			0.84
At2g46170	eFP TAIR Neighbours	reticulon family protein (RTNLB5)	SAM G	ABA Down		0.84
At5g67510	eFP TAIR Neighbours	60S ribosomal protein L26 (RPL26B)				0.84
At5g14450	eFP TAIR Neighbours	GDSL-motif lipase/hydrolase family protein				0.84
At3g56490	eFP TAIR Neighbours	zinc-binding protein, putative / protein kinase C inhibitor, putative				0.84
At1g16870	eFP TAIR Neighbours	mitochondrial 28S ribosomal protein S29-related				0.84
At5g44650	eFP TAIR Neighbours	similar to unnamed protein product [Vitis vinifera] (GB:CAO44475.1)				0.84
At1g27970	eFP TAIR Neighbours	NTF2B (NUCLEAR TRANSPORT FACTOR 2B); Ran GTPase binding / protein transporter	SAM G			0.84
At5g63980	eFP TAIR Neighbours	SAL1 (FIERY1); 3'(2'),5'-bisphosphate nucleotidase/ inositol or phosphatidylinositol phosphatase	SAM G			0.84
At2g39890	eFP TAIR Neighbours	ProT1 (PROLINE TRANSPORTER 1); amino acid transmembrane transporter				0.84
At2g43330	eFP TAIR Neighbours	ATINT1 (INOSITOL TRANSPORTER 1); carbohydrate transmembrane transporter/ sugar:hydrogen ion symporter	SAM G			0.84
At1g11430	eFP TAIR Neighbours	plastid developmental protein DAG, putative	SAM G			0.84
At1g04480	eFP TAIR Neighbours	60S ribosomal protein L23 (RPL23A)				0.84
At1g53240	eFP TAIR Neighbours	malate dehydrogenase (NAD), mitochondrial				0.84
At1g01300	eFP TAIR Neighbours	aspartyl protease family protein	SAM G	ABA Up		0.83
At1g77350	eFP TAIR Neighbours	similar to unnamed protein product [Vitis vinifera] (GB:CAO47891.1)	SAM G			0.83
At3g18800	eFP TAIR Neighbours	similar to unnamed protein product [Vitis vinifera] (GB:CAO46487.1)				0.83
At5g63400	eFP TAIR Neighbours	ADK1 (ADENYLATE KINASE 1); adenylate kinase				0.83
At1g71100	eFP TAIR Neighbours	RSW10 (RADIAL SWELLING 10); ribose-5-phosphate isomerase				0.83
At2g40660	eFP TAIR Neighbours	tRNA-binding region domain-containing protein				0.83
At5g48300	eFP TAIR Neighbours	ADG1 (ADP GLUCOSE PYROPHOSPHORYLASE SMALL SUBUNIT 1); glucose-1-phosphate adenylyltransferase	SAM G			0.83
At5g03300	eFP TAIR Neighbours	ADK2 (ADENOSINE KINASE 2); kinase				0.83

At1g29980	eFP TAIR Neighbours	similar to unknown protein [Arabidopsis thaliana] (TAIR:AT2G34510.1); similar to unknown [Populus trichocarpa] (GB:ABK95079.1); contains InterPro domain Protein of unknown function DUF642 (InterPro:IPR006946); contains InterPro domain Galactose-binding like (InterPro:IPR008979)				0.83
At1g70280	eFP TAIR Neighbours	NHL repeat-containing protein				0.83
At1g15810	eFP TAIR Neighbours	ribosomal protein S15 family protein	SAM G	ABA Up		0.83
At4g25050	eFP TAIR Neighbours	ACP4 (ACYL CARRIER PROTEIN 4)		ABA Down		0.83
At5g23900	eFP TAIR Neighbours	60S ribosomal protein L13 (RPL13D)				0.83
At3g20330	eFP TAIR Neighbours	aspartate carabmoyltransferase, chloroplast / aspartate transcarbamylase / ATCase (PYRB)				0.83
At1g27450	eFP TAIR Neighbours	APT1; adenine phosphoribosyltransferase				0.83
At1g76160	eFP TAIR Neighbours	SKS5 (SKU5 Similar 5); copper ion binding / oxidoreductase		ABA Down		0.83
At1g69530	eFP TAIR Neighbours	ATEXPA1 (ARABIDOPSIS THALIANA EXPANSIN A1)		ABA Up	GA Up	0.83
At1g29880	eFP TAIR Neighbours	glycyl-tRNA synthetase / glycine--tRNA ligase		ABA Up		0.83
At3g04770	eFP TAIR Neighbours	RPSAB (40S RIBOSOMAL PROTEIN SA B); structural constituent of ribosome	SAM G			0.83
At5g16870	eFP TAIR Neighbours	similar to unknown protein [Arabidopsis thaliana] (TAIR:AT3G03010.1); similar to unknown protein [Arabidopsis thaliana] (TAIR:AT3G03010.2); similar to hypothetical protein [Vitis vinifera] (GB:CAN83813.1); contains InterPro domain Peptidyl-tRNA hydrolase, PTH2 (InterPro:IPR002833)				0.83
At1g76790	eFP TAIR Neighbours	O-methyltransferase family 2 protein	SAM G			0.83
At1g64090	eFP TAIR Neighbours	reticulon family protein (RTNLB3)	SAM G			0.83
At5g55280	eFP TAIR Neighbours	FTSZ1-1 (FtsZ1-1); structural molecule				0.83
At2g40010	eFP TAIR Neighbours	60S acidic ribosomal protein P0 (RPP0A)				0.83
At3g61820	eFP TAIR Neighbours	aspartyl protease family protein	SAM G			0.83
At5g14040	eFP TAIR Neighbours	mitochondrial phosphate transporter	SAM G			0.83
At1g11680	eFP TAIR Neighbours	CYP51G1 (CYTOCHROME P450 51); oxygen binding				0.82
At3g14310	eFP TAIR Neighbours	ATPME3 (Arabidopsis thaliana pectin methylesterase 3)	SAM G			0.82
At5g27820	eFP TAIR Neighbours	ribosomal protein L18 family protein		ABA Down		0.82
At4g17600	eFP TAIR Neighbours	LIL3:1; transcription factor		ABA Down		0.82
At5g15520	eFP TAIR Neighbours	40S ribosomal protein S19 (RPS19B)		ABA Down		0.82
At5g13510	eFP TAIR Neighbours	ribosomal protein L10 family protein	SAM G	ABA Down		0.82
At2g30970	eFP TAIR Neighbours	ASP1 (ASPARTATE AMINOTRANSFERASE 1)				0.82
At3g25040	eFP TAIR Neighbours	ER lumen protein retaining receptor, putative / HDEL receptor, putative	SAM G			0.82
At2g47390	eFP TAIR Neighbours	serine-type endopeptidase/ serine-type peptidase	SAM G			0.82
At5g38110	eFP TAIR Neighbours	ASF1B/SGA01/SGA1 (ANTI- SILENCING FUNCTION 1B)				0.82
At1g24360	eFP TAIR Neighbours	3-oxoacyl-(acyl-carrier protein) reductase, chloroplast / 3-ketoacyl-acyl carrier protein reductase				0.82
At4g34120	eFP TAIR Neighbours	LEJ1 (LOSS OF THE TIMING OF ET AND JA BIOSYNTHESIS 1)	SAM G	ABA Down		0.82
At3g62120	eFP TAIR Neighbours	tRNA synthetase class II (G, H, P and S) family protein	SAM G			0.82
At4g18480	eFP TAIR Neighbours	CHLI1 (CHLORINA 42); magnesium chelatase		ABA Down		0.82
At2g20420	eFP TAIR Neighbours	succinyl-CoA ligase (GDP-forming) beta-chain, mitochondrial, putative / succinyl-CoA synthetase, beta chain, putative / SCS-beta, putative				0.82
At4g14440	eFP TAIR Neighbours	enoyl-CoA hydratase/isomerase family protein		ABA Down		0.82
At4g34265	eFP TAIR Neighbours	similar to unknown protein [Arabidopsis thaliana] (TAIR:AT2G15000.3); similar to unknown protein [Arabidopsis thaliana] (TAIR:AT2G15000.2); similar to unnamed protein product [Vitis vinifera] (GB:CAO70855.1)				0.82
At5g28840	eFP TAIR Neighbours	GME (GDP-D-MANNOSE 3',5'-EPIMERASE); GDP-mannose 3,5-epimerase/ NAD binding / catalytic		ABA Down		0.82
At3g15190	eFP TAIR Neighbours	chloroplast 30S ribosomal protein S20, putative		ABA Down		0.82
At1g36310	eFP TAIR Neighbours	methyltransferase				0.82
At2g19670	eFP TAIR Neighbours	protein arginine N-methyltransferase, putative				0.82
At5g08260	eFP TAIR Neighbours	SCPL35 (serine carboxypeptidase-like 35); serine carboxypeptidase				0.82

At3g45310	eFP TAIR Neighbours	cysteine proteinase, putative				0.82
At3g54090	eFP TAIR Neighbours	pfkB-type carbohydrate kinase family protein				0.82
At5g57180	eFP TAIR Neighbours	CIA2 (CHLOROPLAST IMPORT APPARATUS 2)	SAM G	ABA Down		0.82
At2g43800	eFP TAIR Neighbours	formin homology 2 domain-containing protein / FH2 domain-containing protein	SAM G			0.82
At5g25100	eFP TAIR Neighbours	endomembrane protein 70, putative	SAM G			0.82
At3g18420	eFP TAIR Neighbours	tetratricopeptide repeat (TPR)-containing protein				0.82
At5g09650	eFP TAIR Neighbours	ATPPA6 (ARABIDOPSIS THALIANA PYROPHOSPHORYLASE 6); inorganic diphosphatase/ pyrophosphatase				0.82
At5g03940	eFP TAIR Neighbours	FFC (FIFTY-FOUR CHLOROPLAST HOMOLOGUE); 7S RNA binding / GTP binding / mRNA binding	SAM G			0.82
At3g13860	eFP TAIR Neighbours	chaperonin, putative				0.82
At1g21880	eFP TAIR Neighbours	LYM1 (LYSM DOMAIN GPI-ANCHORED PROTEIN 1 PRECURSOR)	SAM G			0.82
At1g55805	eFP TAIR Neighbours	BolA-like family protein				0.82
At3g15680	eFP TAIR Neighbours	zinc finger (Ran-binding) family protein	SAM G			0.82
At5g30510	eFP TAIR Neighbours	RPS1 (ribosomal protein S1); RNA binding		ABA Down		0.82
At4g02930	eFP TAIR Neighbours	elongation factor Tu, putative / EF-Tu, putative				0.82
At4g14960	eFP TAIR Neighbours	TUA6 (tubulin alpha-6 chiain)		ABA Down		0.81
At5g48360	eFP TAIR Neighbours	formin homology 2 domain-containing protein / FH2 domain-containing protein	SAM G			0.81
At1g10522	eFP TAIR Neighbours	similar to unnamed protein product [Vitis vinifera] (GB:CAO40945.1)	SAM G			0.81
At1g75390 At1g75388	eFP TAIR Neighbours	[AT1G75390, ATBZIP44 (ARABIDOPSIS THALIANA BASIC LEUCINE-ZIPPER 44); DNA binding / protein heterodimerization/ transcription factor];[AT1G75388, CPuORF5 (Conserved peptide upstream open reading frame 5)]				0.81
At2g47930	eFP TAIR Neighbours	AGP26/ATAGP26 (ARABINOGALACTAN PROTEINS 26)	SAM G			0.81
At3g25860	eFP TAIR Neighbours	LTA2 (PLASTID E2 SUBUNIT OF PYRUVATE DECARBOXYLASE); dihydrolipoyllysine-residue acetyltransferase	SAM G			0.81
At2g20140	eFP TAIR Neighbours	26S protease regulatory complex subunit 4, putative				0.81
At5g14320	eFP TAIR Neighbours	30S ribosomal protein S13, chloroplast (CS13)				0.81
At3g52930	eFP TAIR Neighbours	fructose-bisphosphate aldolase, putative				0.81
At3g63410	eFP TAIR Neighbours	APG1 (ALBINO OR PALE GREEN MUTANT 1); methyltransferase				0.81
At2g14890	eFP TAIR Neighbours	AGP9 (ARABINOGALACTAN PROTEIN 9)				0.81
At3g17790	eFP TAIR Neighbours	ATACP5 (acid phosphatase 5); acid phosphatase/ protein serine/threonine phosphatase	SAM G		GA Up	0.81
At4g37660	eFP TAIR Neighbours	ribosomal protein L12 family protein	SAM G			0.81
At5g14750	eFP TAIR Neighbours	ATMYB66/WER/WER1 (WEREWOLF 1); DNA binding / protein binding / transcription factor/ transcription regulator			GA Up	0.81
At5g45620	eFP TAIR Neighbours	26S proteasome regulatory subunit, putative (RPN9)	SAM G			0.81
At2g20450	eFP TAIR Neighbours	60S ribosomal protein L14 (RPL14A)				0.81
At1g32990	eFP TAIR Neighbours	PRPL11 (PLASTID RIBOSOMAL PROTEIN L11); structural constituent of ribosome	SAM G			0.81
At4g27380	eFP TAIR Neighbours	similar to hypothetical protein [Vitis vinifera] (GB:CAN79516.1)				0.81
At5g57490	eFP TAIR Neighbours	porin, putative	SAM G			0.81
At1g28510	eFP TAIR Neighbours	similar to unknown protein [Arabidopsis thaliana] (TAIR:AT3G58150.1); similar to unnamed protein product [Vitis vinifera] (GB:CAO66379.1); contains InterPro domain Optic atrophy 3-like (InterPro:IPR010754)	SAM G			0.81
At2g28080	eFP TAIR Neighbours	glycosyltransferase family protein	SAM G	ABA Down		0.81
At4g26300	eFP TAIR Neighbours	EMB1027 (EMBRYO DEFECTIVE 1027); ATP binding / arginine-tRNA ligase	SAM G			0.81
At4g28210	eFP TAIR Neighbours	EMB1923 (EMBRYO DEFECTIVE 1923)	SAM G			0.81
At3g17465	eFP TAIR Neighbours	RPL3P (ribosomal protein large subunit 3P); structural constituent of ribosome				0.81
At5g56260	eFP TAIR Neighbours	dimethylmenaquinone methyltransferase family protein	SAM G	ABA Down		0.81
At4g25370	eFP TAIR Neighbours	Clp amino terminal domain-containing protein		ABA Down		0.81
At5g60670	eFP TAIR Neighbours	60S ribosomal protein L12 (RPL12C)				0.81
At1g78060	eFP TAIR Neighbours	glycosyl hydrolase family 3 protein				0.81

At4g37910	eFP TAIR Neighbours	MTHSC70-1 (mitochondrial heat shock protein 70-1); ATP binding / unfolded protein binding	SAM G	ABA Up		0.81
At3g02560	eFP TAIR Neighbours	40S ribosomal protein S7 (RPS7B)				0.81
At4g12600	eFP TAIR Neighbours	ribosomal protein L7Ae/L30e/S12e/Gadd45 family protein				0.81
At5g57230	eFP TAIR Neighbours	similar to unknown [Populus trichocarpa x Populus deltoides] (GB:ABK96632.1); contains InterPro domain Thioredoxin-like fold (InterPro:IPR012336)				0.8
At5g57280	eFP TAIR Neighbours	methyltransferase				0.8
At1g04520	eFP TAIR Neighbours	33 kDa secretory protein-related				0.8
At1g60770	eFP TAIR Neighbours	pentatricopeptide (PPR) repeat-containing protein				0.8
At1g62750	eFP TAIR Neighbours	ATSCO1/ATSCO1/CPEF-G/SCO1 (SNOWY COTYLEDON1); translation elongation factor/ translation factor, nucleic acid binding	SAM G	ABA Down		0.8
At5g50810	eFP TAIR Neighbours	TIM8 (Arabidopsis thaliana translocase inner membrane subunit 8); P-P-bond-hydrolysis-driven protein transmembrane transporter				0.8
At5g13870	eFP TAIR Neighbours	EXGT-A4 (ENDOXYLOGLUCAN TRANSFERASE A4); hydrolase, acting on glycosyl bonds			GA Up	0.8
At2g33330	eFP TAIR Neighbours	33 kDa secretory protein-related	SAM G			0.8
At2g37250	eFP TAIR Neighbours	ADK/ATPADK1 (ADENOSINE KINASE); adenylate kinase/ nucleotide kinase	SAM G			0.8
At4g21150	eFP TAIR Neighbours	ribophorin II (RPN2) family protein	SAM G			0.8
At1g10030	eFP TAIR Neighbours	ERG28 (ARABIDOPSIS HOMOLOG OF YEAST ERGOSTEROL28)	SAM G			0.8
At5g35620	eFP TAIR Neighbours	LSP1 (LOSS OF SUSCEPTIBILITY TO POTYVIRUSES); RNA binding / translation initiation factor				0.8
At3g27740	eFP TAIR Neighbours	CARA (CARBAMOYL PHOSPHATE SYNTHETASE A); carbamoyl-phosphate synthase (glutamine-hydrolyzing)	SAM G			0.8
At4g05400	eFP TAIR Neighbours	similar to unknown protein [Arabidopsis thaliana] (TAIR:AT4G21140.1); similar to unnamed protein product [Vitis vinifera] (GB:CAO39711.1)				0.8
At4g32400	eFP TAIR Neighbours	SHS1 (SODIUM HYPERSENSITIVE 1); binding / transporter	SAM G			0.8
At1g01910	eFP TAIR Neighbours	anion-transporting ATPase, putative		ABA Down		0.8
At3g62030	eFP TAIR Neighbours	ROC4 (ROTAMASE CYP 4); peptidyl-prolyl cis-trans isomerase		ABA Down		0.8
At2g21260 At2g21250	eFP TAIR Neighbours	[AT2G21260, mannose 6-phosphate reductase (NADPH-dependent), putative];[AT2G21250, mannose 6-phosphate reductase (NADPH-dependent), putative]				0.8
At5g51100	eFP TAIR Neighbours	FSD2 (FE SUPEROXIDE DISMUTASE 2); iron superoxide dismutase				0.8
At3g53800	eFP TAIR Neighbours	armadillo/beta-catenin repeat family protein	SAM G			0.8
At1g20950	eFP TAIR Neighbours	pyrophosphate--fructose-6-phosphate 1-phosphotransferase-related / pyrophosphate-dependent 6-phosphofructose-1-kinase-related				0.8
At5g05690	eFP TAIR Neighbours	CPD (CABBAGE 3); oxygen binding	SAM G	ABA Down		0.8
At1g03360	eFP TAIR Neighbours	ATRRP4; exonuclease				0.8
At3g62460 At3g62530	eFP TAIR Neighbours	[AT3G62460, similar to PBS lyase HEAT-like repeat-containing protein [Arabidopsis thaliana] (TAIR:AT3G62530.1); similar to unnamed protein product [Vitis vinifera] (GB:CAO40196.1); contains InterPro domain Protein of unknown function DUF537 (InterPro:IPR007491)];[AT3G62530, PBS lyase HEAT-like repeat-containing protein]				0.8
At1g26740	eFP TAIR Neighbours	structural constituent of ribosome				0.8
At2g46420	eFP TAIR Neighbours	similar to unknown protein [Arabidopsis thaliana] (TAIR:AT3G61700.1); similar to unnamed protein product [Vitis vinifera] (GB:CAO39750.1); contains InterPro domain Conserved hypothetical protein CHP01589, plant (InterPro:IPR006476)	SAM G			0.8
At4g03210	eFP TAIR Neighbours	XTH9 (XYLOGLUCAN ENDOTRANSGLUCOSYLASE/HYDROLASE 9); hydrolase, acting on glycosyl bonds		ABA Down	GA Up	0.8
At4g22250	eFP TAIR Neighbours	zinc finger (C3HC4-type RING finger) family protein				0.8
At1g60090	eFP TAIR Neighbours	glycosyl hydrolase family 1 protein	SAM G			0.8
At2g43130	eFP TAIR Neighbours	ARA4 (Arabidopsis Rab GTPase homolog A5c); GTP binding	SAM G			0.8
At3g27240	eFP TAIR Neighbours	cytochrome c1, putative	SAM G			0.8
At3g10610	eFP TAIR Neighbours	40S ribosomal protein S17 (RPS17C)				0.8
At2g04030	eFP TAIR Neighbours	CR88 (EMBRYO DEFECTIVE 1956); ATP binding	SAM G			0.8
At3g07430	eFP TAIR Neighbours	EMB1990 (EMBRYO DEFECTIVE 1990)	SAM G			0.8
At2g37660	eFP TAIR Neighbours	binding / catalytic/ coenzyme binding		ABA Down		0.8

At5g41970	eFP TAIR Neighbours	similar to unknown protein [Arabidopsis thaliana] (TAIR:AT3G49320.1); similar to unnamed protein product [Vitis vinifera] (GB:CAO62930.1); contains InterPro domain Metal-dependent protein hydrolase (InterPro:IPR003226)				0.8
At1g65290	eFP TAIR Neighbours	MTACP2 (MITOCHONDRIAL ACYL CARRIER PROTEIN 2); acyl carrier				0.8
At5g58250	eFP TAIR Neighbours	similar to hypothetical protein [Vitis vinifera] (GB:CAN75840.1); contains domain PD020337 (PD020337)	SAM G			0.8
At5g45920	eFP TAIR Neighbours	carboxylesterase	SAM G	ABA Up		0.8
At3g05910	eFP TAIR Neighbours	pectinacetylesterase, putative	SAM G			0.8
At3g23700	eFP TAIR Neighbours	S1 RNA-binding domain-containing protein	SAM G			0.8
At1g08580	eFP TAIR Neighbours	similar to Pm52 [Prunus mume] (GB:BAE48663.1)				0.8
At3g06040	eFP TAIR Neighbours	ribosomal protein L12 family protein				0.8
At3g02650	eFP TAIR Neighbours	pentatricopeptide (PPR) repeat-containing protein				0.8
At1g22450	eFP TAIR Neighbours	COX6B (CYTOCHROME C OXIDASE 6B); cytochrome-c oxidase				0.8
At5g22440	eFP TAIR Neighbours	60S ribosomal protein L10A (RPL10aC)				0.8
At3g49080	eFP TAIR Neighbours	ribosomal protein S9 family protein	SAM G			0.8
At2g47590	eFP TAIR Neighbours	PHR2 (PHOTOLYASE/BLUE-LIGHT RECEPTOR 2)	SAM G			0.8
At3g44890	eFP TAIR Neighbours	RPL9 (ribosomal protein L9); structural constituent of ribosome		ABA Down		0.8
At1g09390	eFP TAIR Neighbours	GDSL-motif lipase/hydrolase family protein				0.8
At1g54780	eFP TAIR Neighbours	thylakoid lumen 18.3 kDa protein		ABA Down		0.8
At2g22670	eFP TAIR Neighbours	IAA8 (indoleacetic acid-induced protein 8); transcription factor				0.8
At5g26830	eFP TAIR Neighbours	threonyl-tRNA synthetase / threonine--tRNA ligase (THRRS)				0.8
At3g55010	eFP TAIR Neighbours	ATPURM/PUR5; phosphoribosylformylglycinamidine cyclo-ligase				0.8
At1g44835	eFP TAIR Neighbours	YbaK/prolyl-tRNA synthetase family protein				0.8
At1g42960	eFP TAIR Neighbours	expressed protein localized to the inner membrane of the chloroplast.	SAM G			0.8
At3g23990	eFP TAIR Neighbours	HSP60 (Heat shock protein 60); ATP binding / protein binding / unfolded protein binding				0.8
At1g26880	eFP TAIR Neighbours	60S ribosomal protein L34 (RPL34A)				0.8
At4g10480	eFP TAIR Neighbours	nascent polypeptide associated complex alpha chain protein, putative / alpha-NAC, putative				0.8
At3g13120	eFP TAIR Neighbours	30S ribosomal protein S10, chloroplast, putative				0.8
At1g02560	eFP TAIR Neighbours	CLPP5 (NUCLEAR ENCODED CLP PROTEASE 1); endopeptidase Clp	SAM G			0.8
At1g07070	eFP TAIR Neighbours	60S ribosomal protein L35a (RPL35aA)				0.79
At2g24490	eFP TAIR Neighbours	ATRPA2/ROR1/RPA2 (REPLICON PROTEIN A); protein binding	SAM G			0.79
At3g14240	eFP TAIR Neighbours	subtilase family protein	SAM G	ABA Up		0.79
At5g24300	eFP TAIR Neighbours	ATSS1/SSI (STARCH SYNTHASE I); transferase, transferring glycosyl groups	SAM G			0.79
At1g29070	eFP TAIR Neighbours	ribosomal protein L34 family protein	SAM G	ABA Down		0.79
At1g33810	eFP TAIR Neighbours	similar to unknown [Populus trichocarpa] (GB:ABK94119.1)	SAM G			0.79
At3g57560	eFP TAIR Neighbours	aspartate/glutamate/uridylate kinase family protein	SAM G			0.79
At3g02870	eFP TAIR Neighbours	VTC4; 3'(2'),5'-bisphosphate nucleotidase/ L-galactose-1-phosphate phosphatase/ inositol or phosphatidylinositol phosphatase	SAM G		GA Up	0.79
At1g50900	eFP TAIR Neighbours	similar to hypothetical protein [Vitis vinifera] (GB:CAN65357.1); similar to unnamed protein product [Vitis vinifera] (GB:CAO38995.1); contains InterPro domain Ankyrin (InterPro:IPR002110)		ABA Down		0.79
At3g48730	eFP TAIR Neighbours	GSA2 (GLUTAMATE-1-SEMIALDEHYDE 2,1-AMINOMUTASE 2); glutamate-1-semialdehyde 2,1-aminomutase	SAM G	ABA Down		0.79
At5g63310	eFP TAIR Neighbours	NDPK2 (NUCLEOSIDE DIPHOSPHATE KINASE 2); ATP binding / nucleoside diphosphate kinase		ABA Down		0.79
At4g10450	eFP TAIR Neighbours	60S ribosomal protein L9 (RPL90D)	SAM G			0.79
At1g44900	eFP TAIR Neighbours	ATP binding / DNA binding / DNA-dependent ATPase	SAM G		GA Up	0.79
At3g14220	eFP TAIR Neighbours	GDSL-motif lipase/hydrolase family protein				0.79
At2g20515	eFP TAIR Neighbours	similar to unnamed protein product [Vitis vinifera] (GB:CAO40634.1)				0.79
At1g15000	eFP TAIR Neighbours	SCPL50 (serine carboxypeptidase-like 50); serine carboxypeptidase	SAM G			0.79

At5g20600	eFP TAIR Neighbours	similar to unnamed protein product [Vitis vinifera] (GB:CAO43199.1); contains InterPro domain Nucleolar, Nop52; (InterPro:IPR010301)				0.79
At1g75580	eFP TAIR Neighbours	auxin-responsive protein, putative				0.79
At4g14890	eFP TAIR Neighbours	ferredoxin family protein				0.79
At4g38210	eFP TAIR Neighbours	ATEXPA20 (ARABIDOPSIS THALIANA EXPANSIN A20)	SAM G			0.79
At2g37790	eFP TAIR Neighbours	aldo/keto reductase family protein				0.79
At1g10760	eFP TAIR Neighbours	SEX1 (STARCH EXCESS 1)				0.79
At1g17650	eFP TAIR Neighbours	phosphogluconate dehydrogenase (decarboxylating)	SAM G			0.79
At3g22230	eFP TAIR Neighbours	60S ribosomal protein L27 (RPL27B)				0.79
At4g25630	eFP TAIR Neighbours	FIB2 (FIBRILLARIN 2)				0.79
At3g12600	eFP TAIR Neighbours	ATNUDT16 (Arabidopsis thaliana Nudix hydrolase homolog 16); hydrolase		ABA Down		0.79
At2g44650	eFP TAIR Neighbours	CHL-CPN10 (chloroplast chaperonin 10)	SAM G	ABA Down		0.79
At2g47940	eFP TAIR Neighbours	DEGP2 (DEGP PROTEASE 2); serine-type peptidase/ trypsin				0.79
At2g18040	eFP TAIR Neighbours	PIN1AT (parvulin 1At)				0.79
At1g79850	eFP TAIR Neighbours	RPS17 (ribosomal protein S17); structural constituent of ribosome				0.79
At2g28600	eFP TAIR Neighbours	ATP binding / ATP-dependent helicase/ nucleic acid binding		ABA Up		0.79
At5g58030	eFP TAIR Neighbours	transport protein particle (TRAPP) component Bet3 family protein		ABA Down		0.79
At4g29410	eFP TAIR Neighbours	60S ribosomal protein L28 (RPL28C)				0.79
At5g57170	eFP TAIR Neighbours	macrophage migration inhibitory factor family protein / MIF family protein	SAM G			0.79
At5g20180	eFP TAIR Neighbours	ribosomal protein L36 family protein				0.79
At3g20820	eFP TAIR Neighbours	leucine-rich repeat family protein	SAM G			0.79
At1g76400	eFP TAIR Neighbours	ribophorin I family protein		ABA Up		0.79
At2g26500	eFP TAIR Neighbours	cytochrome b6f complex subunit (petM), putative		ABA Down		0.79
At3g14790	eFP TAIR Neighbours	RHM3 (RHAMNOSE BIOSYNTHESIS 3); catalytic	SAM G			0.79
At5g28060	eFP TAIR Neighbours	40S ribosomal protein S24 (RPS24B)		ABA Down		0.79
At1g47260	eFP TAIR Neighbours	APFI; carbonate dehydratase		ABA Down		0.79
At5g15650 At3g02230	eFP TAIR Neighbours	[AT5G15650, RGP2 (REVERSIBLY GLYCOSYLATED POLYPEPTIDE 2); alpha-1,4-glucan-protein synthase (UDP-forming)];[AT3G02230, RGP1 (REVERSIBLY GLYCOSYLATED POLYPEPTIDE 1)]				0.79
At3g53890	eFP TAIR Neighbours	40S ribosomal protein S21 (RPS21B)				0.79
At2g16600	eFP TAIR Neighbours	ROC3 (rotamase CyP 3); peptidyl-prolyl cis-trans isomerase				0.79
At4g02580	eFP TAIR Neighbours	NADH-ubiquinone oxidoreductase 24 kDa subunit, putative				0.79
At5g14910	eFP TAIR Neighbours	heavy-metal-associated domain-containing protein		ABA Down		0.79
At3g57290	eFP TAIR Neighbours	EIF3E (eukaryotic translation initiation factor 3E)				0.79
At3g13470	eFP TAIR Neighbours	chaperonin, putative	SAM G			0.79
At3g08740	eFP TAIR Neighbours	elongation factor P (EF-P) family protein		ABA Down		0.79
At1g27400	eFP TAIR Neighbours	60S ribosomal protein L17 (RPL17A)				0.79
At3g56340	eFP TAIR Neighbours	40S ribosomal protein S26 (RPS26C)				0.79
At1g68560	eFP TAIR Neighbours	ATXYL1/XYL1 (ALPHA-XYLOSIDASE 1); alpha-N-arabinofuranosidase/ hydrolase, hydrolyzing O-glycosyl compounds / xylan 1,4-beta-xylosidase		ABA Up		0.78
At4g30930	eFP TAIR Neighbours	NFD1 (NUCLEAR FUSION DEFECTIVE 1); structural constituent of ribosome				0.78
At1g56330	eFP TAIR Neighbours	SAR1 (SECRETION-ASSOCIATED RAS); GTP binding		ABA Down		0.78
At1g18320 At3g10110	eFP TAIR Neighbours	[AT1G18320, mitochondrial import inner membrane translocase subunit Tim17/Tim22/Tim23 family protein];[AT3G10110, MEE67 (maternal effect embryo arrest 67); P-P-bond-hydrolysis-driven protein transmembrane transporter]				0.78
At2g04530	eFP TAIR Neighbours	CPZ				0.78

At1g22270	eFP TAIR Neighbours	Identical to TRM112-like protein At1g22270 [Arabidopsis Thaliana] (GB:Q8LFJ5;GB:Q9LME3); similar to unknown protein [Arabidopsis thaliana] (TAIR:AT1G78190.1); similar to unknown [Picea sitchensis] (GB:ABK23797.1); contains InterPro domain Protein of unknown function DUF343 (InterPro:IPR005651)				0.78
At2g41950	eFP TAIR Neighbours	similar to unnamed protein product [Vitis vinifera] (GB:CAO23423.1); contains domain S-adenosyl-L-methionine-dependent methyltransferases (SSF53335)				0.78
At5g10240	eFP TAIR Neighbours	ASN3 (ASPARAGINE SYNTHETASE 3); asparagine synthase (glutamine-hydrolyzing)	SAM G	ABA Up		0.78
At1g77440	eFP TAIR Neighbours	PBC2 (20S proteasome beta subunit C 2); peptidase				0.78
At1g11580	eFP TAIR Neighbours	ATPMEPCRA; pectinesterase		ABA Down		0.78
At5g23070	eFP TAIR Neighbours	thymidine kinase, putative				0.78
At1g54030	eFP TAIR Neighbours	GDSL-motif lipase, putative				0.78
At1g55200	eFP TAIR Neighbours	protein kinase family protein				0.78
At5g15350	eFP TAIR Neighbours	plastocyanin-like domain-containing protein		ABA Down	GA Up	0.78
At3g20390	eFP TAIR Neighbours	endoribonuclease L-PSP family protein	SAM G			0.78
At5g61770	eFP TAIR Neighbours	PPAN (PETER PAN-LIKE PROTEIN)				0.78
At1g53070	eFP TAIR Neighbours	legume lectin family protein	SAM G			0.78
At3g63200	eFP TAIR Neighbours	PLA IIIB/PLP9 (Patatin-like protein 9); nutrient reservoir				0.78
At1g54000 At1g54010	eFP TAIR Neighbours	[AT1G54000, myrosinase-associated protein, putative];[AT1G54010, myrosinase-associated protein, putative]				0.78
At1g60950	eFP TAIR Neighbours	FED A (FERREDOXIN 2); 2 iron, 2 sulfur cluster binding / electron carrier/ iron-sulfur cluster binding	SAM G	ABA Down		0.78
At3g12930	eFP TAIR Neighbours	similar to unknown [Populus trichocarpa] (GB:ABK94112.1); contains InterPro domain Iojap-related protein (InterPro:IPR004394)	SAM G	ABA Down		0.78
At1g64970	eFP TAIR Neighbours	G-TMT (GAMMA-TOCOPHEROL METHYLTRANSFERASE)			GA Up	0.78
At1g71880	eFP TAIR Neighbours	SUC1 (SUCROSE-PROTON SYMPORTER 1); carbohydrate transmembrane transporter/ sucrose:hydrogen symporter/ sugar:hydrogen ion symporter		ABA Down		0.78
At5g48630	eFP TAIR Neighbours	cyclin family protein	SAM G	ABA Down		0.78
At3g27380	eFP TAIR Neighbours	SDH2-1 (succinate dehydrogenase 2-1)				0.78
At3g48140	eFP TAIR Neighbours	senescence-associated protein, putative		ABA Down		0.78
At5g46280	eFP TAIR Neighbours	DNA replication licensing factor, putative		ABA Down		0.78
At4g34290	eFP TAIR Neighbours	SWIB complex BAF60b domain-containing protein				0.78
At3g09440	eFP TAIR Neighbours	heat shock cognate 70 kDa protein 3 (HSC70-3) (HSP70-3)	SAM G			0.78
At1g07320	eFP TAIR Neighbours	RPL4 (ribosomal protein L4); poly(U) binding / structural constituent of ribosome		ABA Down		0.78
At5g23310	eFP TAIR Neighbours	FSD3 (FE SUPEROXIDE DISMUTASE 3); iron superoxide dismutase				0.78
At2g24280	eFP TAIR Neighbours	serine carboxypeptidase S28 family protein				0.78
At3g27230	eFP TAIR Neighbours	similar to (ARABIDOPSIS THALIANA RAS ASSOCIATED WITH DIABETES PROTEIN 3), methyltransferase [Arabidopsis thaliana] (TAIR:AT5G40830.1); similar to (ARABIDOPSIS THALIANA RAS ASSOCIATED WITH DIABETES PROTEIN 3), methyltransferase [Arabidopsis thaliana] (TAIR:AT5G40830.2); similar to hypothetical protein [Prunus persica] (GB:AAO14627.1); contains domain SSF53335 (SSF53335)	SAM G	ABA Down		0.78
At2g42770	eFP TAIR Neighbours	peroxisomal membrane 22 kDa family protein	SAM G	ABA Down		0.78
At1g80720	eFP TAIR Neighbours	mitochondrial glycoprotein family protein / MAM33 family protein				0.78
At4g00820	eFP TAIR Neighbours	IQD17 (IQ-domain 17); calmodulin binding	SAM G			0.78
At5g19290	eFP TAIR Neighbours	esterase/lipase/thioesterase family protein	SAM G	ABA Down		0.78
At4g37510	eFP TAIR Neighbours	ribonuclease III family protein				0.78
At5g08180	eFP TAIR Neighbours	ribosomal protein L7Ae/L30e/S12e/Gadd45 family protein			GA Up	0.78
At1g61870	eFP TAIR Neighbours	PPR336 (PENTATRICOPEPTIDE REPEAT 336)				0.78
At3g03600	eFP TAIR Neighbours	RPS2 (RIBOSOMAL PROTEIN S2); structural constituent of ribosome				0.78
At4g00810	eFP TAIR Neighbours	60S acidic ribosomal protein P1 (RPP1B)		ABA Down		0.78
At1g75330	eFP TAIR Neighbours	OTC (ORNITHINE CARBAMOYLTRANSFERASE); amino acid binding / carboxyl- or carbamoyltransferase				0.78
At4g34720	eFP TAIR Neighbours	AVA-P1 (vacuolar H -pumping ATPase 16 kDa proteolipid subunit 1); ATPase/ hydrogen ion transporting ATPase, rotational mechanism		ABA Down		0.78

At1g26340	eFP TAIR Neighbours	B5 #6 (cytochrome b5 family protein #6); heme binding / transition metal ion binding				0.78
At5g64290	eFP TAIR Neighbours	DCT/DIT2.1 (DICARBOXYLATE TRANSPORT); oxoglutarate:malate antiporter	SAM G			0.78
At4g39280	eFP TAIR Neighbours	phenylalanyl-tRNA synthetase, putative / phenylalanine--tRNA ligase, putative	SAM G			0.78
At2g19740	eFP TAIR Neighbours	60S ribosomal protein L31 (RPL31A)				0.78
At1g35680	eFP TAIR Neighbours	50S ribosomal protein L21, chloroplast / CL21 (RPL21)				0.78
At5g14050	eFP TAIR Neighbours	transducin family protein / WD-40 repeat family protein				0.78
At5g08160	eFP TAIR Neighbours	ATPK3 (Arabidopsis thaliana serine/threonine protein kinase 3); kinase	SAM G			0.78
At1g80530	eFP TAIR Neighbours	nodulin family protein	SAM G			0.78
At1g66670	eFP TAIR Neighbours	CLPP3 (Clp protease proteolytic subunit 3); endopeptidase Clp	SAM G			0.78
At5g08280	eFP TAIR Neighbours	HEMC (HYDROXYMETHYLBILANE SYNTHASE); hydroxymethylbilane synthase	SAM G	ABA Down		0.78
At4g18040	eFP TAIR Neighbours	EIF4E (EUKARYOTIC TRANSLATION INITATION FACTOR 4E, LOSS-OF-SUSCEPTIBILITY TO POTYVIRUSES); RNA binding / translation initiation factor				0.78
At5g19760	eFP TAIR Neighbours	dicarboxylate/tricarboxylate carrier (DTC)		ABA Down		0.78
At3g19130	eFP TAIR Neighbours	ATRBP47B (RNA-BINDING PROTEIN 47B); RNA binding				0.78
At5g61170	eFP TAIR Neighbours	40S ribosomal protein S19 (RPS19C)				0.78
At1g43670	eFP TAIR Neighbours	fructose-1,6-bisphosphatase, putative / D-fructose-1,6-bisphosphate 1-phosphohydrolase, putative / FBPase, putative	SAM G			0.78
At5g61030	eFP TAIR Neighbours	GR-RBP3 (glycine-rich RNA-binding protein 3); RNA binding				0.77
At2g29530	eFP TAIR Neighbours	TIM10 (Arabidopsis thaliana translocase inner membrane subunit 10); P-P-bond-hydrolysis-driven protein transmembrane transporter				0.77
At2g20585	eFP TAIR Neighbours	NFD6 (NUCLEAR FUSION DEFECTIVE 6)				0.77
At1g77750	eFP TAIR Neighbours	30S ribosomal protein S13, chloroplast, putative				0.77
At3g24660	eFP TAIR Neighbours	TMKL1 (TRANSMEMBRANE KINASE-LIKE 1); ATP binding / kinase/ protein serine/threonine kinase				0.77
At3g54640	eFP TAIR Neighbours	TSA1 (TRYPTOPHAN SYNTHASE ALPHA CHAIN); tryptophan synthase	SAM G			0.77
At3g04260	eFP TAIR Neighbours	PTAC3 (PLASTID TRANSCRIPTIONALLY ACTIVE3); DNA binding				0.77
At5g67220	eFP TAIR Neighbours	nitrogen regulation family protein				0.77
At2g32720	eFP TAIR Neighbours	B5 #4 (cytochrome b5 family protein #4); heme binding / transition metal ion binding				0.77
At3g07170	eFP TAIR Neighbours	sterile alpha motif (SAM) domain-containing protein				0.77
At5g25490	eFP TAIR Neighbours	zinc finger (Ran-binding) family protein	SAM G			0.77
At2g03420	eFP TAIR Neighbours	similar to expressed protein [Oryza sativa (japonica cultivar-group)] (GB:ABF94594.1); similar to hypothetical protein OsI_010237 [Oryza sativa (indica cultivar-group)] (GB:EAY89004.1)		ABA Down		0.77
At3g16560	eFP TAIR Neighbours	protein phosphatase 2C-related / PP2C-related	SAM G			0.77
At4g28440	eFP TAIR Neighbours	DNA-binding protein-related				0.77
At1g20430	eFP TAIR Neighbours	similar to unknown [Populus trichocarpa] (GB:ABK94921.1)				0.77
At2g33255	eFP TAIR Neighbours	hydrolase	SAM G			0.77
At2g34860	eFP TAIR Neighbours	EDA3 (embryo sac development arrest 3); heat shock protein binding / unfolded protein binding				0.77
At5g19520	eFP TAIR Neighbours	mechanosensitive ion channel domain-containing protein / MS ion channel domain-containing protein	SAM G			0.77
At2g44040 At3g59890	eFP TAIR Neighbours	[AT2G44040, dihydrodipicolinate reductase family protein];[AT3G59890, dihydrodipicolinate reductase family protein]				0.77
At2g39870	eFP TAIR Neighbours	similar to unknown protein [Arabidopsis thaliana] (TAIR:AT3G55690.1); similar to unnamed protein product [Vitis vinifera] (GB:CAO69095.1)	SAM G	ABA Up		0.77
At5g10390	eFP TAIR Neighbours	histone H3				0.77
At1g08520	eFP TAIR Neighbours	CHLD/PDE166 (PIGMENT DEFECTIVE 166); magnesium chelatase/ nucleoside-triphosphatase/ nucleotide binding				0.77
At3g58140	eFP TAIR Neighbours	phenylalanyl-tRNA synthetase class IIc family protein				0.77
At3g57650	eFP TAIR Neighbours	LPAT2 (Lysophosphatidyl acyltransferase 2); 1-acylglycerol-3-phosphate O-acyltransferase	SAM G			0.77
At2g38140	eFP TAIR Neighbours	PSRP4 (PLASTID-SPECIFIC RIBOSOMAL PROTEIN 4); structural constituent of ribosome				0.77
At5g49810	eFP TAIR Neighbours	MMT (methionine S-methyltransferase); S-adenosylmethionine-dependent methyltransferase	SAM G	ABA Up		0.77
At5g41600	eFP TAIR Neighbours	BTI3 (VIRB2-INTERACTING PROTEIN 3)				0.77

At2g19680	eFP TAIR Neighbours	mitochondrial ATP synthase g subunit family protein		ABA Down		0.77
At5g14660	eFP TAIR Neighbours	PDF1B (PEPTIDE DEFORMYLASE 1B); peptide deformylase				0.77
At5g11640	eFP TAIR Neighbours	similar to unnamed protein product [Vitis vinifera] (GB:CAO44011.1); contains InterPro domain Thioredoxin-like fold (InterPro:IPR012336); contains InterPro domain Thioredoxin fold (InterPro:IPR012335)	SAM G	ABA Down		0.77
At1g21050	eFP TAIR Neighbours	similar to unknown protein [Arabidopsis thaliana] (TAIR:AT1G76610.1); similar to hypothetical protein [Vitis vinifera] (GB:CAN67637.1); contains InterPro domain Protein of unknown function DUF617, plant (InterPro:IPR006460)				0.77
At5g13490	eFP TAIR Neighbours	AAC2 (ADP/ATP CARRIER 2); ATP:ADP antiporter/ binding				0.77
At1g69440	eFP TAIR Neighbours	AGO7 (ARGONAUTE7); nucleic acid binding				0.77
At2g41680	eFP TAIR Neighbours	thioredoxin reductase, putative / NADPH-dependent thioredoxin reductase, putative		ABA Down		0.77
At4g24830	eFP TAIR Neighbours	arginosuccinate synthase family				0.77
At5g51750	eFP TAIR Neighbours	ATSBT1.3; subtilase	SAM G		GA Up	0.77
At3g46940	eFP TAIR Neighbours	deoxyuridine 5'-triphosphate nucleotidohydrolase family				0.77
At1g62780	eFP TAIR Neighbours	similar to hypothetical protein [Vitis vinifera] (GB:CAN83165.1)		ABA Down		0.77
At3g23800	eFP TAIR Neighbours	selenium-binding family protein		ABA Down		0.77
At1g12000	eFP TAIR Neighbours	pyrophosphate--fructose-6-phosphate 1-phosphotransferase beta subunit, putative / pyrophosphate-dependent 6-phosphofructose-1-kinase, putative				0.77
At3g13580	eFP TAIR Neighbours	60S ribosomal protein L7 (RPL7D)				0.77
At3g55400	eFP TAIR Neighbours	OVA1 (OVULE ABORTION 1); ATP binding / aminoacyl-tRNA ligase				0.77
At3g27570	eFP TAIR Neighbours	similar to unknown protein [Arabidopsis thaliana] (TAIR:AT5G40510.1); similar to unnamed protein product [Vitis vinifera] (GB:CAO14698.1); contains InterPro domain Thioredoxin-like fold (InterPro:IPR012336); contains InterPro domain Thioredoxin fold (InterPro:IPR012335); contains InterPro domain Sucraseferredoxin-like (InterPro:IPR009737)	SAM G			0.77
At5g53620	eFP TAIR Neighbours	similar to unnamed protein product [Vitis vinifera] (GB:CAO48590.1)	SAM G	ABA Up		0.77
At1g21690	eFP TAIR Neighbours	EMB1968 (EMBRYO DEFECTIVE 1968); ATPase				0.77
At4g14420	eFP TAIR Neighbours	lesion inducing protein-related		ABA Down		0.77
At5g59850	eFP TAIR Neighbours	40S ribosomal protein S15A (RPS15aF)				0.77
At5g40770	eFP TAIR Neighbours	ATPHB3 (PROHIBITIN 3)				0.77
At2g44120	eFP TAIR Neighbours	60S ribosomal protein L7 (RPL7C)				0.77
At5g27770	eFP TAIR Neighbours	60S ribosomal protein L22 (RPL22C)				0.77
At4g31700	eFP TAIR Neighbours	RPS6 (RIBOSOMAL PROTEIN S6); structural constituent of ribosome				0.77
At2g32060	eFP TAIR Neighbours	40S ribosomal protein S12 (RPS12C)		ABA Down		0.77
At1g54270	eFP TAIR Neighbours	EIF4A-2 (eukaryotic translation initiation factor 4A-2); ATP-dependent helicase	SAM G			0.77
At3g23620	eFP TAIR Neighbours	brix domain-containing protein				0.77
At2g30060	eFP TAIR Neighbours	Ran-binding protein 1b (RanBP1b)				0.77
At4g25890	eFP TAIR Neighbours	60S acidic ribosomal protein P3 (RPP3A)				0.76
At4g31790	eFP TAIR Neighbours	diphthine synthase, putative (DPH5)		ABA Down		0.76
At1g13730	eFP TAIR Neighbours	nuclear transport factor 2 (NTF2) family protein / RNA recognition motif (RRM)-containing protein	SAM G			0.76
At1g08500	eFP TAIR Neighbours	plastocyanin-like domain-containing protein			GA Up	0.76
At4g12650	eFP TAIR Neighbours	endomembrane protein 70, putative	SAM G			0.76
At4g28360 At1g52370	eFP TAIR Neighbours	[AT4G28360, ribosomal protein L22 family protein];[AT1G52370, ribosomal protein L22 family protein]				0.76
At2g32220	eFP TAIR Neighbours	60S ribosomal protein L27 (RPL27A)				0.76
At4g26230	eFP TAIR Neighbours	60S ribosomal protein L31 (RPL31B)				0.76
At3g06730	eFP TAIR Neighbours	thioredoxin family protein	SAM G			0.76
At5g65700	eFP TAIR Neighbours	BAM1 (big apical meristem 1); ATP binding / kinase/ protein serine/threonine kinase				0.76
At4g32690	eFP TAIR Neighbours	GLB3 (2-on-2 hemoglobin like gene 3)	SAM G	ABA Down		0.76
At4g23620	eFP TAIR Neighbours	50S ribosomal protein-related				0.76

At2g23150	eFP TAIR Neighbours	NRAMP3 (NRAMP metal ion transporter 3); manganese ion transmembrane transporter/ metal ion transmembrane transporter	SAM G	ABA Down		0.76
At3g20740	eFP TAIR Neighbours	FIE (FERTILIZATION-INDEPENDENT ENDOSPERM 1); nucleotide binding / transcription factor				0.76
At3g43810	eFP TAIR Neighbours	CAM7 (CALMODULIN 7); calcium ion binding		ABA Down		0.76
At5g57970	eFP TAIR Neighbours	methyladenine glycosylase family protein				0.76
At2g05790	eFP TAIR Neighbours	glycosyl hydrolase family 17 protein	SAM G			0.76
At3g27280	eFP TAIR Neighbours	ATPHB4 (PROHIBITIN 4)				0.76
At4g16410	eFP TAIR Neighbours	similar to hypothetical protein [Vitis vinifera] (GB:CAN73304.1); similar to unnamed protein product [Vitis vinifera] (GB:CAO39941.1); contains InterPro domain Protein of unknown function DUF751 (InterPro:IPR008470)		ABA Down		0.76
At2g35450	eFP TAIR Neighbours	hydrolase	SAM G	ABA Down		0.76
At2g36620	eFP TAIR Neighbours	RPL24A (RIBOSOMAL PROTEIN L24); structural constituent of ribosome				0.76
At3g04760	eFP TAIR Neighbours	pentatricopeptide (PPR) repeat-containing protein	SAM G	ABA Down		0.76
At1g54500	eFP TAIR Neighbours	rubredoxin family protein	SAM G			0.76
At3g02050	eFP TAIR Neighbours	KUP3 (K uptake permease 3); potassium ion transmembrane transporter	SAM G			0.76
At5g60890	eFP TAIR Neighbours	ATMYB34/ATR1/MYB34 (ALTERED TRYPTOPHAN REGULATION, MYB DOMAIN PROTEIN 34); DNA binding / kinase/ transcription activator/ transcription factor	SAM G		GA Up	0.76
At2g20360	eFP TAIR Neighbours	binding / catalytic/ coenzyme binding				0.76
At4g30200	eFP TAIR Neighbours	similar to VIN3 (VERNALIZATION INSENSITIVE 3), protein binding / zinc ion binding [Arabidopsis thaliana] (TAIR:AT5G57380.1); similar to unnamed protein product [Vitis vinifera] (GB:CAO43227.1); contains InterPro domain Fibronectin, type III-like fold (InterPro:IPR008957); contains InterPro domain Fibronectin, type III (InterPro:IPR003961)	SAM G			0.76
At1g57660 At1g57860	eFP TAIR Neighbours	[AT1G57660, 60S ribosomal protein L21 (RPL21E)];[AT1G57860, 60S ribosomal protein L21]				0.76
At3g25530	eFP TAIR Neighbours	ATGHBDH/GHBDH; 3-hydroxybutyrate dehydrogenase/ phosphogluconate dehydrogenase (decarboxylating)		ABA Down		0.76
At2g30980	eFP TAIR Neighbours	shaggy-related protein kinase delta / ASK-delta / ASK-dzeta (ASK4)				0.76
At5g67150	eFP TAIR Neighbours	transferase family protein				0.76
At4g17520	eFP TAIR Neighbours	nuclear RNA-binding protein, putative				0.76
At2g33180	eFP TAIR Neighbours	similar to unnamed protein product [Vitis vinifera] (GB:CAO41107.1)		ABA Down		0.76
At5g25020 At5g24990	eFP TAIR Neighbours	[AT5G25020, similar to unknown protein [Arabidopsis thaliana] (TAIR:AT5G24990.1); similar to hypothetical protein [Vitis vinifera] (GB:CAN83078.1); contains InterPro domain Protein of unknown function DUF1336 (InterPro:IPR009769)];[AT5G24990, similar to unknown protein [Arabidopsis thaliana] (TAIR:AT5G25010.1); similar to hypothetical protein [Vitis vinifera] (GB:CAN83078.1); contains InterPro domain Protein of unknown function DUF1336 (InterPro:IPR009769)]				0.76
At1g78370	eFP TAIR Neighbours	ATGSTU20 (Arabidopsis thaliana Glutathione S-transferase (class tau) 20); glutathione transferase				0.76
At4g36880	eFP TAIR Neighbours	CP1 (CYSTEINE PROTEINASE1); cysteine-type peptidase			GA Up	0.76
At5g19750	eFP TAIR Neighbours	peroxisomal membrane 22 kDa family protein	SAM G	ABA Down		0.76
At3g54210	eFP TAIR Neighbours	ribosomal protein L17 family protein		ABA Down		0.76
At2g47510 At5g50950	eFP TAIR Neighbours	[AT2G47510, FUM1 (FUMARASE 1); fumarate hydratase];[AT5G50950, fumarate hydratase, putative / fumarase, putative]				0.76
At2g38810	eFP TAIR Neighbours	HTA8; DNA binding				0.76
At3g26060	eFP TAIR Neighbours	ATPRX Q; antioxidant/ peroxiredoxin	SAM G	ABA Down		0.76
At3g59980	eFP TAIR Neighbours	tRNA-binding region domain-containing protein		ABA Down		0.76
At2g06520	eFP TAIR Neighbours	PSBX (photosystem II subunit X)	SAM G	ABA Down		0.76
At5g54900	eFP TAIR Neighbours	ATRBP45A (RNA-BINDING PROTEIN 45A); RNA binding				0.76
At2g46820	eFP TAIR Neighbours	PSAP/PSI-P/PTAC8/TMP14 (THYLAKOID MEMBRANE PHOSPHOPROTEIN OF 14 KDA); DNA binding	SAM G	ABA Down		0.76
At3g02110	eFP TAIR Neighbours	SCPL25 (serine carboxypeptidase-like 25); serine carboxypeptidase			GA Up	0.76
At5g10560	eFP TAIR Neighbours	glycosyl hydrolase family 3 protein	SAM G			0.76
At5g45650	eFP TAIR Neighbours	subtilase family protein				0.76
At3g15720	eFP TAIR Neighbours	glycoside hydrolase family 28 protein / polygalacturonase (pectinase) family protein			GA Up	0.76

At1g16000	eFP TAIR Neighbours	similar to unknown protein [Arabidopsis thaliana] (TAIR:AT1G80890.1); similar to Os12g0556400 [Oryza sativa (japonica cultivar-group)] (GB:NP_001067003.1); similar to hypothetical protein OsI_037443 [Oryza sativa (indica cultivar-group)] (GB:EAY83484.1); similar to hypothetical protein LOC_Os12g36930 [Oryza sativa (japonica cultivar-group)] (GB:ABA99557.1)				0.76
At3g61440	eFP TAIR Neighbours	ATCYSC1 (BETA-SUBSTITUTED ALA SYNTHASE 3;1); L-3-cyanoalanine synthase/ cysteine synthase		ABA Down		0.76
At5g57290	eFP TAIR Neighbours	60S acidic ribosomal protein P3 (RPP3B)				0.76
At5g17710	eFP TAIR Neighbours	EMB1241 (EMBRYO DEFECTIVE 1241); adenyl-nucleotide exchange factor/ chaperone binding / protein binding / protein homodimerization				0.76
At1g63940	eFP TAIR Neighbours	monodehydroascorbate reductase, putative		ABA Down		0.76
At5g11480	eFP TAIR Neighbours	GTP binding				0.76
At5g20500	eFP TAIR Neighbours	glutaredoxin, putative		ABA Down		0.76
At1g34430	eFP TAIR Neighbours	EMB3003 (EMBRYO DEFECTIVE 3003); dihydrolipoyllysine-residue acetyltransferase				0.76
At3g25920	eFP TAIR Neighbours	RPL15 (ribosomal protein L15)				0.76
At3g57490	eFP TAIR Neighbours	40S ribosomal protein S2 (RPS2D)				0.76
At5g57330	eFP TAIR Neighbours	aldose 1-epimerase family protein				0.76
At5g08300	eFP TAIR Neighbours	succinyl-CoA ligase (GDP-forming) alpha-chain, mitochondrial, putative / succinyl-CoA synthetase, alpha chain, putative / SCS-alpha, putative	SAM G			0.76
At1g67660	eFP TAIR Neighbours	DNA binding / magnesium ion binding / nuclease	SAM G			0.76
At5g20080	eFP TAIR Neighbours	NADH-cytochrome b5 reductase, putative				0.76
At1g07370	eFP TAIR Neighbours	PCNA1 (PROLIFERATING CELLULAR NUCLEAR ANTIGEN); DNA binding / DNA polymerase processivity factor		ABA Down	GA Up	0.76
At1g48350	eFP TAIR Neighbours	ribosomal protein L18 family protein		ABA Down		0.76
At5g54970	eFP TAIR Neighbours	similar to unknown protein [Arabidopsis thaliana] (TAIR:AT4G26960.1)	SAM G	ABA Down		0.76
At3g27820	eFP TAIR Neighbours	ATMDAR4/MDAR4 (MONODEHYDROASCORBATE REDUCTASE 4); monodehydroascorbate reductase (NADH)	SAM G			0.76
At3g04840	eFP TAIR Neighbours	40S ribosomal protein S3A (RPS3aA)				0.76
At5g13450	eFP TAIR Neighbours	ATP synthase delta chain, mitochondrial, putative / H( )-transporting two-sector ATPase, delta (OSCP) subunit, putative				0.76
At2g43030	eFP TAIR Neighbours	ribosomal protein L3 family protein				0.76
At4g11010	eFP TAIR Neighbours	NDPK3 (NUCLEOSIDE DIPHOSPHATE KINASE 3); ATP binding / nucleoside diphosphate kinase				0.76
At1g10960	eFP TAIR Neighbours	ATFD1 (FERREDOXIN 1); 2 iron, 2 sulfur cluster binding / electron carrier/ iron-sulfur cluster binding		ABA Down		0.75
At4g34730	eFP TAIR Neighbours	ribosome-binding factor A family protein	SAM G	ABA Down		0.75
At2g18230	eFP TAIR Neighbours	ATPPA2 (ARABIDOPSIS THALIANA PYROPHOSPHORYLASE 2); inorganic diphosphatase/ pyrophosphatase	SAM G			0.75
At1g14410	eFP TAIR Neighbours	ATWHY1/PTAC1 (A. THALIANA WHIRLY 1); DNA binding / telomeric DNA binding	SAM G			0.75
At5g11560	eFP TAIR Neighbours	catalytic				0.75
At3g56910	eFP TAIR Neighbours	PSRP5 (PLASTID-SPECIFIC 50S RIBOSOMAL PROTEIN 5)		ABA Down		0.75
At1g20050	eFP TAIR Neighbours	HYD1 (Hydra 1)	SAM G			0.75
At1g51980	eFP TAIR Neighbours	mitochondrial processing peptidase alpha subunit, putative	SAM G	ABA Down		0.75
At3g59650	eFP TAIR Neighbours	mitochondrial ribosomal protein L51/S25/CI-B8 family protein				0.75
At4g24330	eFP TAIR Neighbours	similar to unknown protein [Arabidopsis thaliana] (TAIR:AT5G49945.1); similar to hypothetical protein [Vitis vinifera] (GB:CAN63262.1); contains InterPro domain Protein of unknown function DUF1682 (InterPro:IPR012879)	SAM G			0.75
At3g27060	eFP TAIR Neighbours	TSO2 (TSO MEANING 'UGLY' IN CHINESE); ribonucleoside-diphosphate reductase		ABA Down		0.75
At5g23250	eFP TAIR Neighbours	succinyl-CoA ligase (GDP-forming) alpha-chain, mitochondrial, putative / succinyl-CoA synthetase, alpha chain, putative / SCS-alpha, putative				0.75
At4g08520 At3g09800	eFP TAIR Neighbours	[AT4G08520, clathrin adaptor complex small chain family protein];[AT3G09800, clathrin adaptor complex small chain family protein]				0.75
At2g40490	eFP TAIR Neighbours	HEME2; uroporphyrinogen decarboxylase	SAM G	ABA Down		0.75
At4g29510	eFP TAIR Neighbours	ATPRMT11/PRMT11 (ARABIDOPSIS ARGININE METHYLTRANSFERASE 11); protein-arginine N-methyltransferase				0.75
At1g66150	eFP TAIR Neighbours	TMK1 (TRANSMEMBRANE KINASE 1)				0.75
At1g55900	eFP TAIR Neighbours	TIM50 (EMBRYO DEFECTIVE 1860)	SAM G			0.75

At3g16080	eFP TAIR Neighbours	60S ribosomal protein L37 (RPL37C)				0.75
At2g03090	eFP TAIR Neighbours	ATEXPA15 (ARABIDOPSIS THALIANA EXPANSIN A15)	SAM G	ABA Up		0.75
At1g43560	eFP TAIR Neighbours	ATY2 (Arabidopsis thioredoxin y2); thiol-disulfide exchange intermediate	SAM G		GA Up	0.75
At4g38660	eFP TAIR Neighbours	thaumatin, putative				0.75
At5g19690	eFP TAIR Neighbours	STT3A (STAUROSPORIN AND TEMPERATURE SENSITIVE 3-LIKE A); oligosaccharyl transferase				0.75
At3g15710	eFP TAIR Neighbours	signal peptidase, putative				0.75
At5g46290	eFP TAIR Neighbours	KAS I (3-KETOACYL-ACYL CARRIER PROTEIN SYNTHASE I); fatty-acid synthase		ABA Down		0.75
At3g07510	eFP TAIR Neighbours	similar to unknown protein [Arabidopsis thaliana] (TAIR:AT2G01580.1); similar to hypothetical protein [Vitis vinifera] (GB:CAN81121.1); similar to unnamed protein product [Vitis vinifera] (GB:CAO16627.1)				0.75
At3g58180	eFP TAIR Neighbours	PBS lyase HEAT-like repeat-containing protein				0.75
At2g38120	eFP TAIR Neighbours	AUX1 (AUXIN RESISTANT 1); amino acid transmembrane transporter/ transporter	SAM G	ABA Down	GA Up	0.75
At1g74670	eFP TAIR Neighbours	gibberellin-responsive protein, putative			GA Up	0.75
At3g52940	eFP TAIR Neighbours	FK (FACKEL); delta14-sterol reductase	SAM G	ABA Down		0.75
At1g54580 At1g54630	eFP TAIR Neighbours	[AT1G54580, ACP2 (ACYL CARRIER PROTEIN 2)];[AT1G54630, ACP3 (ACYL CARRIER PROTEIN 3)]				0.75
At3g26710	eFP TAIR Neighbours	similar to unnamed protein product [Vitis vinifera] (GB:CAO49107.1)	SAM G	ABA Down		0.75
At4g20270	eFP TAIR Neighbours	BAM3 (big apical meristem 3); ATP binding / protein serine/threonine kinase		ABA Up		0.75
At1g01090	eFP TAIR Neighbours	PDH-E1 ALPHA (PYRUVATE DEHYDROGENASE E1 ALPHA); pyruvate dehydrogenase (acetyl-transferring)				0.75
At3g50340	eFP TAIR Neighbours	similar to unknown protein [Arabidopsis thaliana] (TAIR:AT5G67020.1); similar to unknown protein [Oryza sativa (japonica cultivar-group)] (GB:BAD09363.1)	SAM G	ABA Up		0.75
At5g18650	eFP TAIR Neighbours	zinc finger (C3HC4-type RING finger) family protein	SAM G		GA Up	0.75
At5g44710	eFP TAIR Neighbours	similar to unnamed protein product [Vitis vinifera] (GB:CAO41922.1); contains InterPro domain Ribosomal protein S27, mitochondrial (InterPro:IPR013219)				0.75
At5g53160	eFP TAIR Neighbours	similar to unknown protein [Arabidopsis thaliana] (TAIR:AT4G27920.1); similar to unnamed protein product [Vitis vinifera] (GB:CAO69376.1); similar to hypothetical protein [Vitis vinifera] (GB:CAN64668.1); contains InterPro domain Bet v I allergen; (InterPro:IPR000916)		ABA Down		0.75
At4g00860	eFP TAIR Neighbours	ATOZI1 (ARABIDOPSIS THALIANA OZONE-INDUCED PROTEIN 1)		ABA Down		0.75
At5g16290	eFP TAIR Neighbours	acetolactate synthase small subunit, putative	SAM G			0.75
At2g24560	eFP TAIR Neighbours	carboxylesterase		ABA Up		0.75
At5g11710	eFP TAIR Neighbours	(EPSIN1); binding				0.75
At1g02640	eFP TAIR Neighbours	BXL2 (BETA-XYLOSIDASE 2); hydrolase, hydrolyzing O-glycosyl compounds			GA Up	0.75
At4g04640	eFP TAIR Neighbours	ATPC1 (ATP synthase gamma chain 1)		ABA Down		0.75
At1g53580	eFP TAIR Neighbours	ETHE1/GLX2-3/GLY3 (GLYOXALASE 2-3); hydroxyacylglutathione hydrolase	SAM G			0.75
At2g17630	eFP TAIR Neighbours	phosphoserine aminotransferase, putative		ABA Down		0.75
At1g75350	eFP TAIR Neighbours	EMB2184 (EMBRYO DEFECTIVE 2184); structural constituent of ribosome		ABA Down		0.75
At2g39970	eFP TAIR Neighbours	peroxisomal membrane protein (PMP36)		ABA Down		0.75
At2g01290	eFP TAIR Neighbours	ribose-5-phosphate isomerase	SAM G			0.75
At1g49760	eFP TAIR Neighbours	PAB8 (POLY(A) BINDING PROTEIN 8); RNA binding / translation initiation factor				0.75
At3g11630	eFP TAIR Neighbours	2-cys peroxiredoxin, chloroplast (BAS1)				0.75
At3g29350	eFP TAIR Neighbours	AHP2 (HISTIDINE-CONTAINING PHOSPHOTRANSMITTER 2); histidine phosphotransfer kinase/ protein binding / signal transducer				0.75
At4g11120	eFP TAIR Neighbours	translation elongation factor Ts (EF-Ts), putative				0.75
At5g61130	eFP TAIR Neighbours	glycosyl hydrolase family protein 17				0.75
At5g16070	eFP TAIR Neighbours	chaperonin, putative		ABA Down		0.75
At1g16520	eFP TAIR Neighbours	similar to unknown protein [Arabidopsis thaliana] (TAIR:AT1G56080.1); similar to unknown [Populus trichocarpa] (GB:ABK96045.1); contains InterPro domain Paired amphipathic helix; (InterPro:IPR003822)				0.75
At2g47780	eFP TAIR Neighbours	rubber elongation factor (REF) protein-related				0.75
At2g26330	eFP TAIR Neighbours	ER (ERECTA)		ABA Up		0.75

At2g16510	eFP TAIR Neighbours	vacuolar ATP synthase 16 kDa proteolipid subunit 5 / V-ATPase 16 kDa proteolipid subunit 5 (AVAP5)	SAM G	ABA Down		0.75
At2g29570	eFP TAIR Neighbours	PCNA2 (PROLIFERATING CELL NUCLEAR ANTIGEN 2); DNA binding / DNA polymerase processivity factor		ABA Down		0.75
At1g49400	eFP TAIR Neighbours	EMB1129 (EMBRYO DEFECTIVE 1129); structural constituent of ribosome				0.75
At2g21160	eFP TAIR Neighbours	translocon-associated protein alpha (TRAP alpha) family protein		ABA Down		0.75
At4g29350	eFP TAIR Neighbours	PFN2/PRF2/PRO2 (PROFILIN 2); actin binding / protein binding				0.75
At2g28000	eFP TAIR Neighbours	CPN60A (chloroplast / 60 kDa chaperonin alpha subunit); ATP binding / protein binding / unfolded protein binding				0.75
At5g66510	eFP TAIR Neighbours	GAMMA CA3 (GAMMA CARBONIC ANHYDRASE 3); carbonate dehydratase		ABA Down		0.75
At4g17390	eFP TAIR Neighbours	60S ribosomal protein L15 (RPL15B)				0.75
At5g56030	eFP TAIR Neighbours	HSP81-2 (EARLY-RESPONSIVE TO DEHYDRATION 8); ATP binding				0.75
At3g12390	eFP TAIR Neighbours	nascent polypeptide associated complex alpha chain protein, putative / alpha-NAC, putative				0.75
At3g61070	eFP TAIR Neighbours	PEX11E	SAM G			0.75
At5g49540	eFP TAIR Neighbours	similar to unknown [Picea sitchensis] (GB:ABK26769.1); contains InterPro domain Protein of unknown function DUF786 (InterPro:IPR008504)				0.75
At3g04120	eFP TAIR Neighbours	GAPC (GLYCERALDEHYDE-3-PHOSPHATE DEHYDROGENASE C SUBUNIT); glyceraldehyde-3-phosphate dehydrogenase				0.75
At3g23390	eFP TAIR Neighbours	60S ribosomal protein L36a/L44 (RPL36aA)		ABA Down		0.75

Candidate gene *At1g14810* as identified on IL 1-1-3 putatively associated with organic acid and hexose sugars and correlated genes as generated by SeedNet available on http://vseednet.nottingham.ac.uk. The candidate gene codes for an aspartate semialdeyhde dehydrogenase. The copredicted genes are supplied with the Pearson coefficient values. Correlated genes of relevance to glycolysis are highlighted in grey. Localization of gene candidates was achieved by utilizing data as analyzed on dry IL seeds of harvest seasons I and II in Akko, Israel.
