# Supplementary material for: Metabolic Profiling of a Mapping Population Exposes New Insights in the Regulation of Seed Metabolism and Seed, Fruit, and Plant Relations
Source: PLoS Genet. 2012 Mar 29;8(3):e1002612. doi: 10.1371/journal.pgen.1002612 (PMC3315483; doi:10.1371/journal.pgen.1002612)
Supplement: Table S5 — Candidate gene At1g20575 and its correlated genes associated with organic acid and hexose sugars on IL 4-4. Candidate gene At1g20575 as identified on IL 4-4 putatively associated with organic acids and hexose sugars and co-predicted genes as generated by SCoPNET available on http://vseednet.nottingham.ac.uk. The candidate gene is involved in D-ribose catabolism. The co-predicted genes are supplied with the co-prediction PMI values. Co-predicted genes of relevance to organic acids or sugars are highlighted in grey. Localization of gene candidates was achieved by utilizing data as analyzed on dry IL seeds of harvest seasons I and II in Akko, Israel. (PDF) [file pgen.1002612.s014.pdf]

SCoPNET Co-Prediction Table S5. Candidate gene At1g20575 and its correlated genes associated with organic acid and hexose sugars on IL 4-4.

AGI	Links	Annotation	ABA Regulation	GA Regulation	Dormancy Node Strength	Germination Node Strength	PMI score
At5g15070	eFP TAIR Neighbours	acid phosphatase/ oxidoreductase/ transition metal ion binding			1	4	7.1
At4g23660	eFP TAIR Neighbours	ATPPT1 (ARABIDOPSIS THALIANA POLYPRENYLTRANSFERASE 1); 4-hydroxybenzoate nonaprenyltransferase			0	5	7.1
At3g27020	eFP TAIR Neighbours	YSL6 (YELLOW STRIPE LIKE 6); oligopeptide transporter	Col ABA UP		2	3	7.1
At1g30473	eFP TAIR Neighbours	metal ion binding			3	3	6.91
At5g66250	eFP TAIR Neighbours	kinectin-related			5	2	6.76
At5g49840	eFP TAIR Neighbours	ATP-dependent Clp protease ATP-binding subunit ClpX, putative			1	6	6.76
At2g32950	eFP TAIR Neighbours	COP1 (CONSTITUTIVE PHOTOMORPHOGENIC 1)	Col ABA UP		0	7	6.76
At1g24180	eFP TAIR Neighbours	IAR4 (IAA-conjugate-resistant 4); pyruvate dehydrogenase (acetyl-transferring)			0	7	6.76
At4g32680	eFP TAIR Neighbours	similar to unnamed protein product [Vitis vinifera] (GB:CAO44139.1)			1	7	6.63
At3g45700	eFP TAIR Neighbours	proton-dependent oligopeptide transport (POT) family protein			1	7	6.63
At1g03470	eFP TAIR Neighbours	kinase interacting family protein			3	5	6.63
At3g16530	eFP TAIR Neighbours	legume lectin family protein			2	7	6.51
At4g12250	eFP TAIR Neighbours	GAE5 (UDP-D-GLUCURONATE 4-EPIMERASE 5); catalytic			0	11	6.31
At2g16480	eFP TAIR Neighbours	SWIB complex BAF60b domain-containing protein / plus-3 domain-containing protein			2	9	6.31
At3g11320	eFP TAIR Neighbours	organic anion transmembrane transporter			0	12	6.22
AtCg00905_ AtCg01230	eFP TAIR Neighbours				9	4	6.14
At1g56090	eFP TAIR Neighbours	tetratricopeptide repeat (TPR)-containing protein		GA Down	10	3	6.14
At3g17611	eFP TAIR Neighbours	rhomboid family protein / zinc finger protein-related			1	16	5.87
At3g60730	eFP TAIR Neighbours	pectinesterase family protein			5	13	5.82
At2g07360	eFP TAIR Neighbours	SH3 domain-containing protein			11	12	5.57
At5g27150	eFP TAIR Neighbours	NHX1 (NA /H EXCHANGER); sodium ion transmembrane transporter/ sodium:hydrogen antiporter			0	26	5.45
At1g76340	eFP TAIR Neighbours	integral membrane family protein	Col ABA Down		0	30	5.3
At1g66650	eFP TAIR Neighbours	seven in absentia (SINA) protein, putative			3	37	5.02
At1g55730_ At1g55720	eFP TAIR Neighbours				0	40	5.02
At1g30860	eFP TAIR Neighbours	protein binding / zinc ion binding			4	44	4.83
At1g63840	eFP TAIR Neighbours	zinc finger (C3HC4-type RING finger) family protein			1	52	4.74
At1g64980	eFP TAIR Neighbours	similar to unnamed protein product [Vitis vinifera] (GB:CAO62125.1); contains domain SSF53448 (SSF53448)	Col ABA Down		0	62	4.58
At4g05440	eFP TAIR Neighbours	EDA35 (embryo sac development arrest 35)			0	370	2.79

Candidate gene *At1g20575* as identified on IL 4-4 putatively associated with organic acids and hexose sugars and copredicted genes as generated by SCoPNET available on http://vseednet.nottingham.ac.uk. The candidate gene is involved in D-ribose catabolism. The copredicted genes are supplied with the coprediction PMI values. Copredicted genes of relevance to organic acids or sugars are highlighted in grey. Localization of gene candidates was achieved by utilizing data as analyzed on dry IL seeds of harvest seasons I and II in Akko, Israel.
