# Supplementary material for: Metabolic Profiling of a Mapping Population Exposes New Insights in the Regulation of Seed Metabolism and Seed, Fruit, and Plant Relations
Source: PLoS Genet. 2012 Mar 29;8(3):e1002612. doi: 10.1371/journal.pgen.1002612 (PMC3315483; doi:10.1371/journal.pgen.1002612)
Supplement: Table S6 — Candidate gene At1g20575 involved in the D-ribose catabolism and its correlated genes associated with organic acid and hexose sugars on IL 4-4. Candidate gene At1g20575 as identified on IL 4-4 putatively associated with organic acid and hexose sugars and correlated genes as generated by SeedNet available on http://vseednet.nottingham.ac.uk. The candidate gene is involved in D-ribose catabolism. The co-predicted genes are supplied with Pearson's coefficient values. Correlated genes of relevance to organic acids or sugars are highlighted in grey. Localization of gene candidates was achieved by utilizing data as analyzed on dry IL seeds of harvest seasons I and II in Akko, Israel. (PDF) [file pgen.1002612.s015.pdf]

SeedNet Correlation Table S6. Candidate gene At1g20575 involved in the D-ribose catabolism and its correlated genes associated with organic acid and hexose sugars on IL 4-4.

AGI	Links	Annotation	SAM NG/G	ABA Regulation	GA Regulation	Correlation
At5g35680	eFP TAIR Neighbours	eukaryotic translation initiation factor 1A, putative / eIF-1A, putative / eIF-4C, putative		ABA Down		0.85
At5g50870	eFP TAIR Neighbours	UBC27 (UBIQUITIN-CONJUGATING ENZYME 27); ubiquitin-protein ligase				0.85
At3g59280	eFP TAIR Neighbours	TXR1 (THAXTOMIN A RESISTANT 1)				0.84
At1g28120	eFP TAIR Neighbours	Identical to Ubiquitin thioesterase otubain-like [Arabidopsis Thaliana] (GB:Q8LG98;GB:Q9C7E1); similar to otubain-like cysteine protease [Populus trichocarpa] (GB:ABU93349.1); contains InterPro domain Ovarian tumour, otubain (InterPro:IPR003323); contains InterPro domain Ubiquitin thioesterase Otubain (InterPro:IPR016615)				0.83
At5g20090	eFP TAIR Neighbours	similar to unknown protein [Arabidopsis thaliana] (TAIR:AT4G14695.1); similar to unknown protein [Arabidopsis thaliana] (TAIR:AT4G22310.1); similar to hypothetical protein [Vitis vinifera] (GB:CAN75662.1); contains InterPro domain Protein of unknown function UPF0041 (InterPro:IPR005336)				0.83
At3g46010	eFP TAIR Neighbours	ADF1 (ACTIN DEPOLYMERIZING FACTOR 1)				0.83
At1g22920	eFP TAIR Neighbours	AJH1/CSN5A/JAB1 (COP9 SIGNALOSOME 5A)				0.83
At2g24040	eFP TAIR Neighbours	hydrophobic protein, putative / low temperature and salt responsive protein, putative		ABA Down		0.82
At4g10040	eFP TAIR Neighbours	CYTC-2 (CYTOCHROME C-2); electron carrier		ABA Down		0.82
At5g49540	eFP TAIR Neighbours	similar to unknown [Picea sitchensis] (GB:ABK26769.1); contains InterPro domain Protein of unknown function DUF786 (InterPro:IPR008504)				0.81
At1g65220	eFP TAIR Neighbours	eIF4-gamma/eIF5/eIF2-epsilon domain-containing protein		ABA Down		0.81
At3g50360	eFP TAIR Neighbours	ATCEN2 (CENTRIN2); calcium ion binding		ABA Down		0.81
At3g57870	eFP TAIR Neighbours	AHUS5 (SUMO CONJUGATION ENZYME 1); ubiquitin-protein ligase		ABA Down		0.81
At1g53460	eFP TAIR Neighbours	structural molecule				0.8
At5g06190	eFP TAIR Neighbours	unknown protein		ABA Down		0.8
At1g49740	eFP TAIR Neighbours	phospholipase C				0.8
At3g07640	eFP TAIR Neighbours	similar to unnamed protein product [Vitis vinifera] (GB:CAO41126.1)				0.8
At1g43900	eFP TAIR Neighbours	protein phosphatase 2C, putative / PP2C, putative				0.8
At1g12780	eFP TAIR Neighbours	UGE1 (UDP-D-GLUCOSE/UDP-D-GALACTOSE 4-EPIMERASE 1); UDP-glucose 4-epimerase/ protein dimerization				0.8
At3g25230	eFP TAIR Neighbours	ROF1 (ROTAMASE FKBP 1); FK506 binding / calmodulin binding / peptidyl-prolyl cis-trans isomerase				0.8
At3g17940	eFP TAIR Neighbours	aldose 1-epimerase family protein				0.8
At5g19440	eFP TAIR Neighbours	cinnamyl-alcohol dehydrogenase, putative (CAD)		ABA Up		0.8
At4g39730	eFP TAIR Neighbours	lipid-associated family protein				0.79
At2g03870	eFP TAIR Neighbours	small nuclear ribonucleoprotein, putative / snRNP, putative / Sm protein, putative				0.79
At3g58180	eFP TAIR Neighbours	PBS lyase HEAT-like repeat-containing protein				0.79
At5g05370	eFP TAIR Neighbours	ubiquinol-cytochrome C reductase complex ubiquinone-binding protein, putative / ubiquinol-cytochrome C reductase complex 8.2 kDa protein, putative				0.79
At1g02330	eFP TAIR Neighbours	similar to unnamed protein product [Vitis vinifera] (GB:CAO40200.1); contains InterPro domain Hepatocellular carcinoma-associated antigen 59 (InterPro:IPR010756)				0.79
At1g67350	eFP TAIR Neighbours	unknown protein				0.79
At5g51880	eFP TAIR Neighbours	oxidoreductase, acting on paired donors, with incorporation or reduction of molecular oxygen, 2-oxoglutarate as one donor, and incorporation of one atom each of oxygen into both donors		ABA Down		0.79
At3g10860	eFP TAIR Neighbours	ubiquinol-cytochrome C reductase complex ubiquinone-binding protein, putative / ubiquinol-cytochrome C reductase complex 8.2 kDa protein, putative				0.79

At5g03455	eFP TAIR Neighbours	CDC25				0.78
At4g05390	eFP TAIR Neighbours	ATRFNR1 (ROOT FNR 1); oxidoreductase		ABA Down		0.78
At5g08290	eFP TAIR Neighbours	YLS8 (yellow-leaf-specific gene 8); catalytic				0.78
At5g03030	eFP TAIR Neighbours	DNAJ heat shock N-terminal domain-containing protein				0.78
At4g02620	eFP TAIR Neighbours	(VACUOLAR ATPASE SUBUNIT F); hydrogen ion transporting ATP synthase, rotational mechanism / hydrogen ion transporting ATPase, rotational mechanism				0.78
At3g18430	eFP TAIR Neighbours	calcium-binding EF hand family protein				0.78
At2g29420	eFP TAIR Neighbours	ATGSTU7 (GLUTATHIONE S-TRANSFERASE 25); glutathione transferase				0.78
At3g07910	eFP TAIR Neighbours	similar to unnamed protein product [Vitis vinifera] (GB:CAO16842.1)				0.78
At5g65110	eFP TAIR Neighbours	ACX2 (ACYL-COA OXIDASE 2); acyl-CoA oxidase				0.78
At3g46460	eFP TAIR Neighbours	UBC13 (ubiquitin-conjugating enzyme 13); ubiquitin-protein ligase				0.77
At4g12790	eFP TAIR Neighbours	ATP-binding family protein				0.77
At3g09970	eFP TAIR Neighbours	calcineurin-like phosphoesterase family protein				0.77
At3g07170	eFP TAIR Neighbours	sterile alpha motif (SAM) domain-containing protein				0.77
At4g00860	eFP TAIR Neighbours	ATOZI1 (ARABIDOPSIS THALIANA OZONE-INDUCED PROTEIN 1)		ABA Down		0.77
At2g42910	eFP TAIR Neighbours	ribose-phosphate pyrophosphokinase 4 / phosphoribosyl diphosphate synthetase 4 (PRS4)				0.77
At1g43710	eFP TAIR Neighbours	EMB1075 (EMBRYO DEFECTIVE 1075); carboxy-lyase				0.77
At1g25380	eFP TAIR Neighbours	mitochondrial substrate carrier family protein				0.77
At4g35260	eFP TAIR Neighbours	IDH1 (ISOCITRATE DEHYDROGENASE 1); isocitrate dehydrogenase (NAD)				0.77
At1g48030	eFP TAIR Neighbours	dihydrolipoamide dehydrogenase 1, mitochondrial / lipoamide dehydrogenase 1 (MTLPD1)				0.77
At5g40650	eFP TAIR Neighbours	SDH2-2 (succinate dehydrogenase 2-2)		ABA Down		0.76
At3g62450	eFP TAIR Neighbours	similar to hypothetical protein MtrDRAFT_AC140551g61v2 [Medicago truncatula] (GB:ABE91880.2)				0.76
At3g60880	eFP TAIR Neighbours	dihydrodipicolinate synthase 1 (DHDPS1) (DHDPS) (DHPS1)				0.76
At1g48140	eFP TAIR Neighbours	dolichol-phosphate mannosyltransferase-related		ABA Down		0.76
At1g64190	eFP TAIR Neighbours	6-phosphogluconate dehydrogenase family protein		ABA Down		0.76
At5g49480	eFP TAIR Neighbours	ATCP1 (CA2 -BINDING PROTEIN 1); calcium ion binding		ABA Down		0.76
At3g57450	eFP TAIR Neighbours	similar to unnamed protein product [Vitis vinifera] (GB:CAO40798.1)		ABA Down		0.76
At3g09085	eFP TAIR Neighbours	similar to unnamed protein product [Vitis vinifera] (GB:CAO15778.1)				0.76
At3g44190	eFP TAIR Neighbours	pyridine nucleotide-disulphide oxidoreductase family protein				0.76
At4g17650	eFP TAIR Neighbours	aromatic-rich family protein				0.76
At2g45730	eFP TAIR Neighbours	eukaryotic initiation factor 3 gamma subunit family protein				0.76
At3g52730	eFP TAIR Neighbours	ubiquinol-cytochrome C reductase UQCRX/QCR9-like family protein		ABA Down		0.76
At5g40770	eFP TAIR Neighbours	ATPHB3 (PROHIBITIN 3)				0.76
At4g23885	eFP TAIR Neighbours	similar to unknown protein [Arabidopsis thaliana] (TAIR:AT5G24165.1); similar to unnamed protein product [Vitis vinifera] (GB:CAO69543.1)				0.75
At3g03420	eFP TAIR Neighbours	Ku70-binding family protein				0.75
At5g22950	eFP TAIR Neighbours	VPS24.1				0.75
At5g53940	eFP TAIR Neighbours	yippee family protein				0.75
At2g30530	eFP TAIR Neighbours	similar to unknown protein [Arabidopsis thaliana] (TAIR:AT5G01970.1); similar to unnamed protein product [Vitis vinifera] (GB:CAO21287.1)		ABA Up		0.75
At5g14710	eFP TAIR Neighbours	similar to unnamed protein product [Vitis vinifera] (GB:CAO14760.1)		ABA Down		0.75

At5g17280	eFP TAIR Neighbours	similar to Os09g0530900 [Oryza sativa (japonica cultivar-group)] (GB:NP_001063752.1); similar to unknown [Populus trichocarpa] (GB:ABK92656.1)		ABA Down		0.75
At5g63000	eFP TAIR Neighbours	similar to hypothetical protein OsJ_001206 [Oryza sativa (japonica cultivar-group)] (GB:EAZ11381.1); similar to Os01g0271000 [Oryza sativa (japonica cultivar-group)] (GB:NP_001042705.1); similar to hypothetical protein OsI_001310 [Oryza sativa (indica cultivar-group)] (GB:EAY73463.1)				0.75
At2g31570	eFP TAIR Neighbours	ATGPX2 (GLUTATHIONE PEROXIDASE 2); glutathione peroxidase		ABA Down	GA Up	0.75
At5g53560	eFP TAIR Neighbours	ATB5-A (Cytochrome b5 A)				0.75
At1g05180	eFP TAIR Neighbours	AXR1 (AUXIN RESISTANT 1); small protein activating enzyme				0.75
At3g04780	eFP TAIR Neighbours	Encodes a protein with little sequence identity with any other protein of known structure or function. Part of this protein shows a 42 = sequence identity with the C-terminal domain of the 32-kD human thioredoxin-like protein.				0.75
At5g53540	eFP TAIR Neighbours	MSP1 protein, putative / intramitochondrial sorting protein, putative				0.75

Candidate gene *At1g20575* as identified on IL 4-4 putatively associated with organic acid and hexose sugars and correlated genes as generated by SeedNet available on http://vseednet.nottingham.ac.uk. The candidate gene is involved in D-ribose catabolism. The copredicted genes are supplied with the Pearson coefficient values. Correlated genes of relevance to organic acids or sugars are highlighted in grey. Localization of gene candidates was achieved by utilizing data as analyzed on dry IL seeds of harvest seasons I and II in Akko, Israel.
