# Supplementary material for: Metabolic Profiling of a Mapping Population Exposes New Insights in the Regulation of Seed Metabolism and Seed, Fruit, and Plant Relations
Source: PLoS Genet. 2012 Mar 29;8(3):e1002612. doi: 10.1371/journal.pgen.1002612 (PMC3315483; doi:10.1371/journal.pgen.1002612)
Supplement: Table S7 — Parameters as calculated on seed and fruit networks. Based on the networks presented in Figure 7, Figure 8, and Figures S5, S6, S7, S8, S9, the following typical network typical parameters at different r thresholds were computed: number of nodes, number of edges, degree of connectivity, clustering coefficients, network density, and network diameter. In addition, the percentage of the significant correlations at a given r threshold in accordance to the total number of correlations were calculated. (PDF) [file pgen.1002612.s016.pdf]

Table S7. Parameters as calculated on seed and fruit networks.

	Seed season I network r=0.3	Seed season I network r=0.5	Fruit season I network r=0.3	Fruitseason I network r=0.5	Seed-Fruit season I network r=0.3	Seed season II network r=0.3	seed season I ∪ II network r=0.3	seed season I ∩ II network r=0.3
**No. of Nodes**	63	55	83	53	150	50	63	44
**No. of Edges**	689	225	383	134	1289	366	899	146
**% of significant correlations of total no. of correlations**	37.01	13.62	12.5	3.17	12.96	30.5	28.8 [1]	4.68 [1]
**Degree of connectivity**	21.87	8.18	9.23	5.06	17.19	14.64	28.54	6.6
**Clustering coefficient**	0.61	0.4	0.38	0.45	0.46	0.61	0.65	0.4
**Network Density**	0.35	0.15	0.11	0.09	0.12	0.3	0.46	0.15
**Network Diameter**	6	8	7	4	6	4	4	5

[1] of total number of correlations of seasons I and II

Based on the networks presented in Figures 7 and 8 and Figures S5-S9, network typical parameters at different *r* thresholds were computed: number of nodes, number of edges, degree of connectivity, clustering coefficient, network density, and the network diameter. Also, the percentage of the significant correlations at a given *r* threshold in accordance to the total number of correlations were calculated.
