# Supplementary material for: Metabolic Profiling of a Mapping Population Exposes New Insights in the Regulation of Seed Metabolism and Seed, Fruit, and Plant Relations
Source: PLoS Genet. 2012 Mar 29;8(3):e1002612. doi: 10.1371/journal.pgen.1002612 (PMC3315483; doi:10.1371/journal.pgen.1002612)
Supplement: Table S8 — Fruit ILs ranked based on individual variances compared with overall average variance of seed IL population. Shown are the 25 fruit ILs chosen for the subset network parameters comparison with reference to the network parameters of the seed IL network. ILs were ranked based on their variance as compared with the average variances of the seed IL dataset. To show differences of variances F-statistics were applied to estimate p-values. None of the chosen ILs show significant differences between individual IL variance and overall average seed IL dataset variance. (PDF) [file pgen.1002612.s017.pdf]

Table S8. Fruit ILs ranked based on individual variances compared to overall average variance of seed IL population.

	σ^2	p of F-value statistics
**Seed complete**	**0.4687**	**-**
Fruit IL1-4	0.4776	0.4323
Fruit IL3-2	0.4791	0.4337
Fruit IL8-3	0.48	0.4345
Fruit IL1-4-18	0.4846	0.4385
Fruit IL5-3	0.427	0.3846
Fruit IL9-2-5	0.5158	0.4644
Fruit IL5-4	0.4183	0.3757
Fruit IL10-2	0.5222	0.4694
Fruit IL3-4	0.4133	0.3705
Fruit IL4-3	0.3966	0.3529
Fruit IL9-3	0.5447	0.4863
Fruit IL9-1-2	0.5489	0.4893
Fruit IL2-4	0.5503	0.4903
Fruit IL1-1-3	0.5518	0.4914
Fruit M82	0.3602	0.3123
Fruit IL7-5-5	0.3597	0.3117
Fruit IL2-1-1	0.358	0.3098
Fruit IL3-5	0.5909	0.5174
Fruit IL10-3	0.3457	0.2955
Fruit IL1-1-2	0.3395	0.2881
Fruit IL4-2	0.3158	0.2595
Fruit IL7-4-1	0.3096	0.2519
Fruit IL12-2	0.3047	0.2459
Fruit IL9-2-6	0.2912	0.2292
Fruit IL11-1	0.2909	0.2287

Shown are the 25 fruit ILs chosen to perform subset network parameters comparison in reference to the network parameters of the seed IL network. ILs were ranked based on their variance as compared to the average variances of the seed IL dataset. To show differences of variances F-statistics were applied to estimate p-values.

None of the chosen ILs show significant differences between individual IL variance and overall average seed IL dataset variance.
