# Supplementary material for: Metabolic Profiling of a Mapping Population Exposes New Insights in the Regulation of Seed Metabolism and Seed, Fruit, and Plant Relations
Source: PLoS Genet. 2012 Mar 29;8(3):e1002612. doi: 10.1371/journal.pgen.1002612 (PMC3315483; doi:10.1371/journal.pgen.1002612)
Supplement: Table S9 — Conserved correlations across seasons and tissues. Conserved correlations across seasons within the same tissue. Conserved correlations of combinatorial fruit-seed networks were also compared for different seasons. To compare networks, datasets must be synchronized for metabolites, which leads to loss of correlations. Roman Capitals refer to harvest seasons. (PDF) [file pgen.1002612.s018.pdf]

Conserved correlation Table S9. Conserved correlations across seasons and tissues.

**Tissue - Season**	**n of correlations**	**n of conserved correlations**	**% of conserved correlations**	**% of conserved correlations in accordance to network 1**	**% of conserved correlations in accordance to network 2**
Seed I	689	-	-	-	-
Seed II	366	-	-	-	-
Fruit I	383	-	-	-	-
Fruit III	434	-	-	-	-
Fruit IV	741	-	-	-	-
Seed I ∪ II	899	146	16.2	21.1	40.0
Fruit I ∪ III	787	92	11.7	21.2	24
Fruit I ∪ IV	1050	101	9.6	13.6	26.4
Fruit III ∪ IV	1555	231	14.9	53.3	31.1
(Fruit I ∪ Seed I) ∩ (Fruit I ∪ Seed II)	257	0	0.0	0.0	0.0
(Fruit III ∪ Seed I) ∩ (Fruit III ∪ Seed II)	474	1	0.0	0.0	0.0
(Fruit IV ∪ Seed I) ∩ (Fruit IV ∪ Seed II)	744	0	0.0	0.0	0.0

Conserved correlations across seasons within the same tissue. Also conserved correlations of combinatorial fruit-seed networks - different seasons compared. To compare networks, datasets need to be synchronized for metabolites, which leads to loss of correlations. Roman capitals refer to harvest seasons.
