# Supplementary material for: Metabolic Profiling of a Mapping Population Exposes New Insights in the Regulation of Seed Metabolism and Seed, Fruit, and Plant Relations
Source: PLoS Genet. 2012 Mar 29;8(3):e1002612. doi: 10.1371/journal.pgen.1002612 (PMC3315483; doi:10.1371/journal.pgen.1002612)
Supplement: Text S1 — Candidate gene identification via the A. thaliana co-response database. (PDF) [file pgen.1002612.s019.pdf]

Text S1. Candidate gene identification via the *A. thaliana* co-response database.

Since the SeedNet coefficient threshold is fixed to a minimum of 0.75 some of the initially identified candidate genes did not generate any hits the SeedNet nor the SCoPNET platform. Thus, to extent our search as described in the main text we also included the *A. thaliana* co-response database (http://csbdb.mpimp-golm.mpg.de/index.html) for all identified candidate genes. The results of these queries are summed up in subsequent tables section: Text S1_Table 1-4.

Amino acid metabolism: Co-localized QTL characterized by quantitative changes in Arg/ornithine content for season I, showed to be conserved for ornithine in season II, namely ILs 2-4 and 12-2. On IL 2-4, the gene *At2g34470* was identified, which is involved in N compound metabolic processes and correlates positively with i) *At1g75330* coding for a ornithine carbamoyltransferase [*A. thaliana* co-response database], which is involved in the Arg conversion to ornithine in plants, ii) *At1g80600* coding for an acetylornithine aminotransferase[*A. thaliana* co-response database], and iii) *At2g37500* coding for an Arg biosynthesis protein [*A. thaliana* Co-response database]. Furthermore, gene *At4g21120* was identified on IL 2-4 encoding for a member of the cationic amino acid transporter subfamily and involved in Arg import. On IL 12-2 one gene was detected, namely *At3g16290*, involved in embryo development and seed dormancy. This gene ortholog correlated to the genes related to Arg/Orn metabolism as *At2g34470* (mentioned above). For a full list of candidate genes and correlating genes see Text S1_Table 1. On ILs 4-3 and 8-1, associated with a conserved quantitative change of Asn, we identified one putative candidate gene on IL 4-3: *At3g16150*, involved in glycoprotein catabolic processes with an asparaginase activity [*A. thaliana* co-response database]. This ortholog correlated positively to genes: *At2g22250*, a glutamate/aspartate-prephenate aminotransferase [*A. thaliana* co-response database] and *At5g11520*, an aspartate amino transferase [*A. thaliana* Co-response database] For exact *r* values refer to Text S1_Table 2.

Glycolysis: Although significant changes for sugars for season I did not show to reoccur for season II, still ILs 4-1, 4-3, 4-3-2 displayed intriguing features. Characterized by highly overlapping segments, these three ILs showed to have increased concentrations of fructose and glucose opposed by decreased

quantities of sucrose, suggesting for a QTL embedding genes with significant impact on sucrose metabolism. For the introgressed segments associated with sucrose, namely 4-1 and 4-3, no gene directly involved in sucrose metabolism was found in the overlapping regions. However, on non-overlapping segments of the same introgression in chromosome 4 we identified ortholog *At4g25650* encoding an oxidation reductase and correlating fructose-6-phosphate 2-kinase involved in glycolysis [*A. thaliana* co-response database] (Text S1_Table 3).
Following the same approach we also identified candidate genes putatively associated with the pattern of change of co-localized fumarate/malate and glucose/fructose (Figure S2) in ILs 1-1-3, 4-4, and 5-4. In these instances we identified *Arabidopsis* orthologs exhibiting significant correlations by correspondence analysis to genes found in the introgressed segment and associated with the co-localized mQTLs. Although a fumarate QTL was detected in the introgression of IL 5-5, the chromosomal segment corresponding to IL 5-4 shares a large portion with IL 5-5 and fumarate content changed significantly also in IL 5-4 as compared to M82, when a less stringent p-value was applied (data not shown). Additionally, on IL 5-4 a QTL for fructose/glucose was identified in the fruit pericarp [2]. However, no putatively associated gene could be found on this segment. That said, on segment IL 1-1-3 we identified an aspartate semialdeyhde dehydrogenase, shown to be correlating to a fructose biphosphate aldolase involved in the catalysis of fructose-6-phosphate, and concomitantly correlating to malate dehydrogenase, as earlier mentioned a key enzyme in the TCA cycle. On IL 4-4, ortholog *At1g19600* involved in the D-ribose catabolism [78] was found correlating to pyruvate kinase, an important enzyme in glycolysis and concomitantly to malate dehydrogenase. For a full list of candidate and correlating genes see Text S1_Table 4.

As before we blasted again all corresponding Unigene identifiers against the the Max Planck Institute of molecular plant physiology in Golm *Solanum pennellii* in-house BLAST search system. Results may be viewed in supplementary Text S2.

Text S1 Tables:

Text S1_Table 1. Seed mQTL and putative genes and their correlated genes associated with Arg and ornithine metabolism.

**Candidate gene/ortholog: At2g34470**			
**Function: nitrogen compound metabolic process [TAIR]**			
**IL2-4**			
**Correlating to:**			
**Gene/ortholog**	**Function**	**r**	**Reference**
At1g75330	ornithine carbamoyltransferase	0.48	*A. thaliana* Co-response database
At1g80600	acetylornithine aminotransferase	0.35	*A. thaliana* Co-response database
At2g37500	arginine biosynthesis protein	0.35	*A. thaliana* Co-response database
**Candidate gene/ortholog: At4g21120**			
**Function: Encodes a member of the cationic amino acid transporter (CAT) subfamily of amino acid polyamine choline transporters. Mediates efficient uptake of Lys, Arg and Glu in a yeast system. L-arginine import, L-glutamate import, basic amino acid transport [TAIR]**			
**IL2-4**			
**Correlating to: no correlation detected in *A. thaliana* Co-response database**			
**Candidate gene/ortholog: At3g16290**			
**Involved in: embryo development, ending in seed dormancy [TAIR]**			
**IL12-2**			
**Correlating to:**			
**Gene/ortholog**	**Function**	**r**	**Reference**
At1g80600	acetylornithine aminotransferase	0.57	*A. thaliana* Co-response database
At1g75330	ornithine carbamoyltransferase	0.38	*A. thaliana* Co-response database
At2g37500	arginine biosynthesis protein	0.46	*A. thaliana* Co-response database

List of candidate genes associated with ILs 2-4 and 12-2 in association with Arg and ornithine mQTL: candidate genes putatively associated with metabolite concentrations of named metabolites in the seed. Each candidate gene, its putative function, its locus, and the correlated genes as identified on the *Arabidopsis thaliana* co-response database hosted at the Max Planck Institute of Molecular Plant Physiology: http://csbdb.mpimp-golm.mpg.de/csbdb/dbcor/ath/ath_tsgq.html, is listed here. The correlated genes are supplied with the correlation *r* value. Localization of gene candidates was achieved by utilizing data as analyzed on dry IL seeds of harvest seasons I and II in Akko, Israel.

Text S1_Table 2. Seed mQTL and putative genes and their correlated genes associated with Asn metabolism

Involved in: glycoprotein catabolic process, has asparaginase activity [TAIR]			
**IL4-3**			
**Correlating to:**			
**Gene/ortholog**	**Function**	**r**	**Reference**
At2g22250	aminotransferase class	0.4	*A. thaliana* Co-response database
At5g11520	aspartate amino transferase	0.35	*A. thaliana* Co-response database

List of candidate genes associated with IL 4-3 in association with Asn mQTL: candidate genes putatively associated with metabolite concentrations of the named metabolite in the seed. Each candidate gene, its putative function, its locus, and the correlated genes as identified on the *Arabidopsis thaliana* co-response database hosted at the Max Planck Institute of Molecular Plant Physiology: http://csbdb.mpimp-golm.mpg.de/csbdb/dbcor/ath/ath_tsgq.html, is listed here. The correlated genes are supplied with the correlation *r* value. Localization of gene candidates was achieved by utilizing data as analyzed on dry IL seeds of harvest seasons I and II in Akko, Israel.

Text S1_Table 3. Seed mQTL and putative genes and their correlated genes associated with sucrose metabolism.

Candidate gene/ortholog: At4g25650			
**Function: encoding an oxidation reductase Function [TAIR]**			
**IL4-1**			
**Correlating to:**			
**Gene/ortholog**	**Function**	**r**	**Reference**
At3g12780	phosphglycerate kinase	0.85	*A. thaliana* Co-response database
At1g07110	fructose-6-phosphate 2-kinase/ fructose-2,6-bisphosphatase (F2KP)	0.72	*A. thaliana* Co-response database
**Candidate gene/ortholog: At5g63840**			
**Function: involved in cellulose biosynthetic processes with a glucosidase and hydrolase activity [TAIR]**			
**IL4-3-2**			
**Correlating to:**			
**Gene/ortholog**	**Function**	**r**	**Reference**
At5g43710	glycoside hydrolase family protein	0.72	*A. thaliana* Co-response database
At1g76550	pyrophosphate-fructose-6-phosphate	0.61	*A. thaliana* Co-response database
**Candidate gene/ortholog: At5g10920**			
**Function: involved in the Arg biosynthetic process [TAIR]**			
**IL4-3**			
**Correlating to:**			
**Gene/ortholog**	**Function**	**r**	**Reference**
At4g32840	phosphofructokinase family protein	0.61	*A. thaliana* Co-response database

List of candidate genes associated with IL 4-3 in association with Asn mQTL: candidate genes putatively associated with metabolite concentrations of the named metabolite in the seed. Each candidate gene, its putative function, its locus, and the correlated genes as identified on the *Arabidopsis thaliana* co-response database hosted at the Max Planck Institute of Molecular Plant Physiology: http://csbdb.mpimp-golm.mpg.de/csbdb/dbcor/ath/ath_tsgq.html, is listed here. The correlated genes are supplied with the correlation *r* value. Localization of gene candidates was achieved by utilizing data as analyzed on dry IL seeds of harvest seasons I and II in Akko, Israel.

Text S1_Table 4. TCA cycle intermediates and sugar mQTL co-localization associated with putative candidate genes and their correlated genes.

**Candidate gene/ortholog: At1g14810**			
**Function: encoding an aspartate semialdehyde dehyrogenase [TAIR]**			
**IL1-1-3**			
**Correlating to (fruit - fructose/glucose):**			
**Gene/ortholog**	**Function**	**r**	**Reference**
At3g52930	fructose-bisphosphate aldolase	0.43	*A. thaliana* co-response database
At1g12000	pyrophosphate--fructose-6-phosphate 1-phosphotransferase beta subunit	0.32	*A. thaliana* co-response database
At5g08570	pyrophosphate--fructose-6-phosphate 1-phosphotransferase beta subunit	0.28	*A. thaliana* co-response database
**Correlating to (seed - fumarate):**			
**Gene/ortholog**	**Function**	**r**	**Reference**
At1g53240	malate dehydrogenase (NAD)	0.80	*A. thaliana* co-response database
At3g47520	malate dehydrogenase (NAD)	0.53	*A. thaliana* co-response database
At4g00570	malate oxidoreductase	0.45	*A. thaliana* co-response database
At1g04410	malate dehydrogenase	0.52	*A. thaliana* co-response database
At2g47510	fumarate hydratase	0.32	*A. thaliana* co-response database
At5g50850	pyruvate dehydrogenase E1 component beta subunit	0.57	*A. thaliana* co-response database
At5g43330	malate dehydrogenase	0.32	*A. thaliana* co-response database
**Candidate gene/ortholog: At2g30200**			
**Function: involved in the fatty acid biosynthetic process [TAIR]**			
**IL1-1-3**			
**Correlating to (fruit - fructose/glucose):**			
**Gene/ortholog**	**Function**	**r**	**Reference**
At1g07110	pyrophosphate--fructose-6-phosphate 1-phosphotransferase-related / pyrophosphate-dependent 6-phosphofructose-1-kinase-related	0.42	*A. thaliana* co-response database
**Correlating to (seed - fumarate):**			
**Gene/ortholog**	**Function**	**r**	**Reference**
At3g47520	malate dehydrogenase (NAD)	0.80	*A. thaliana* co-response database
At1g53240	malate dehydrogenase (NAD)	0.60	*A. thaliana* co-response database
At5g23250	succinyl-CoA ligase (GDP-forming) alpha-chain, mitochondrial, putative / succinyl-CoA synthetase, alpha chain, putative / SCS-alpha, putative	0.61	*A. thaliana* co-response database
At5g49460	ATP-citrate synthase, putative / ATP-citrate (pro-S-)-lyase, putative / citrate cleavage enzyme, putative	0.48	*A. thaliana* co-response database
At1g53240	malate dehydrogenase	0.45	*A. thaliana* co-response database

At3g47520	malate dehydrogenase	0.31	*A. thaliana* co-response database
At2g47510	fumarate hydratase	0.29	*A. thaliana* co-response database

Candidate gene/ortholog: At1g19600

Function: involved in D-ribose catabolic process [TAIR]

IL4-4

Correlating to (fruit - fructose/glucose):

Gene/ortholog	Function	r	Reference
At5g63680	pyruvate kinase	0.50	*A. thaliana* co-response database
At1g76550	pyrophosphate--fructose-6-phosphate 1-phosphotransferase alpha subunit	0.52	*A. thaliana* co-response database
At4g32840	phosphofructokinase family protein	0.46	*A. thaliana* co-response database
At2g36460	fructose-bisphosphate aldolase	0.49	*A. thaliana* co-response database
At5g56350	pyruvate kinase	0.37	*A. thaliana* co-response database
At3g52930	fructose-bisphosphate aldolase	0.42	*A. thaliana* co-response database

Correlating to (seed - fumarate):

Gene/ortholog	Function	r	Reference
At3g1930	dihydrolipoamide S-acetyltransferase	0.28	*A. thaliana* co-response database
At5g23250	succinyl-CoA ligase (GDP-forming) alpha-chain	0.39	*A. thaliana* co-response database
At3g47520	malate dehydrogenase (NAD)	0.30	*A. thaliana* co-response database
Atg04410	malate dehydrogenase (NAD)	0.30	*A. thaliana* co-response database
At5g08300	succinyl-CoA ligase (GDP-forming) alpha-chain	0.30	*A. thaliana* co-response database
At2g47510	fumarate hydratase	-0.08	*A. thaliana* co-response database
At1g24180	pyruvate dehydrogenase E1 component alpha subunit	0.67	*A. thaliana* co-response database
At5g43330	malate dehydrogenase	0.53	*A. thaliana* co-response database
At5g23250	succinyl-CoA ligase (GDP-forming) alpha-chain	0.29	*A. thaliana* co-response database
At2g20420	succinyl-CoA ligase (GDP-forming) beta-chain	0.37	*A. thaliana* co-response database

Candidate gene/ortholog: At1g20575

Function: involved in D-ribose catabolic process [TAIR]

IL4-4

Correlating to (fruit - fructose/glucose):

Gene/ortholog	Function	r	Reference
At5g63680	pyruvate kinase	0.70	*A. thaliana* co-response database
At1g76550	pyrophosphate--fructose-6-phosphate 1-phosphotransferase alpha subunit	0.60	*A. thaliana* co-response database

At5g56350	pyruvate kinase	0.44	*A. thaliana* co-response database
At4g32840	phosphofructokinase family protein	0.27	*A. thaliana* co-response database
**Correlating to (seed - fumarate):**			
**Gene/ortholog**	**Function**	**r**	**Reference**
At1g24180	pyruvate dehydrogenase E1 component alpha subunit	0.51	*A. thaliana* co-response database
At5g66760	succinate dehydrogenase (ubiquinone) flavoprotein subunit	0.63	*A. thaliana* co-response database
At3g15020	malate dehydrogenase (NAD)	0.47	*A. thaliana* co-response database
At5g40650	succinate dehydrogenase	0.41	*A. thaliana* co-response database
At5g43330	malate dehydrogenase	0.56	*A. thaliana* co-response database
At2g44350	citrate synthase	0.64	*A. thaliana* co-response database
At5g08300	succinyl-CoA ligase	0.52	*A. thaliana* co-response database
At1g24180	pyruvate dehydrogenase E1 component alpha subunit	0.67	*A. thaliana* co-response database
At2g20420	succinyl-CoA ligase (GDP-forming) beta-chain	0.48	*A. thaliana* co-response database
At5g23250	succinyl-CoA ligase (GDP-forming) alpha-chain	0.32	*A. thaliana* co-response database
**Candidate gene/ortholog: At4g12590**			
**Function: unknown**			
**IL 5-4**			
**Correlating to (fruit - fructose/glucose):**			
**Gene/ortholog**	**Function**	**r**	**Reference**
At1g12000	pyrophosphate--fructose-6-phosphate 1-phosphotransferase beta subunit	0.36	*A. thaliana* co-response database
At1g76550	pyrophosphate--fructose-6-phosphate 1-phosphotransferase alpha subunit	0.28	*A. thaliana* co-response database

**Correlating to (seed - fumarate):**			
**Gene/ortholog**	**Function**	**r**	**Reference**
At3g15020	malate dehydrogenase (NAD),	0.49	*A. thaliana* co-response database
At5g66760	succinate dehydrogenase (ubiquinone) flavoprotein subunit	0.55	*A. thaliana* co-response database
At5g40650	succinate dehydrogenase, iron-sulphur subunit	0.46	*A. thaliana* co-response database
At3g27380	succinate dehydrogenase, iron-sulphur subunit	0.53	*A. thaliana* co-response database
At5g43330	malate dehydrogenase	0.56	*A. thaliana* co-response database
At5g08300	succinyl-CoA ligase (GDP-forming) alpha-chain	0.52	*A. thaliana* co-response database
At5g66760	succinate dehydrogenase (ubiquinone) flavoprotein subunit	0.48	*A. thaliana* co-response database

At5g50850	pyruvate dehydrogenase E1 component beta subunit	0.47	*A. thaliana* co-response database
At5g40650	succinate dehydrogenase	0.48	*A. thaliana* co-response database
At5g03290	isocitrate dehydrogenase	0.55	*A. thaliana* co-response database

List of candidate genes associated with ILs 1-1-3, 4-4, and 5-4 in association with fumarate, malate, glucose, and fructose mQTL both in the seed and in the fruit: candidate genes putatively associated with metabolite concentrations of the named metabolites in the seed and the fruit. Each candidate gene, its putative function, its locus, and the correlated genes as identified on the *Arabidopsis thaliana* co-response database hosted at the Max Planck Institute of Molecular Plant Physiology: http://csbdb.mpimp-golm.mpg.de/csbdb/dbcor/ath/ath_tsgq.html, is listed here. The correlated genes are supplied with the correlation *r* value. Localization of gene candidates was achieved by utilizing data as analyzed on dry IL seeds and IL fruits of harvest seasons I in Akko, Israel.
