# Supplementary material for: Metabolic Profiling of a Mapping Population Exposes New Insights in the Regulation of Seed Metabolism and Seed, Fruit, and Plant Relations
Source: PLoS Genet. 2012 Mar 29;8(3):e1002612. doi: 10.1371/journal.pgen.1002612 (PMC3315483; doi:10.1371/journal.pgen.1002612)
Supplement: Text S2 — Candidate gene BLAST results against Solanum pennellii. (PDF) [file pgen.1002612.s020.pdf]

Text S2. Genome and promoter sequence of candidate genes as listed in the main text

<u>SGN-U261955: 4697 bp (S. lycopersicum) and 4691 bp (S pennellii)</u>

```
                      *        20         *        40         *        60         *        80         *       100
S.lycopers : CTGCACAATGTTCAATACTAGAACAATGCAGCAACTTTTACTTGTCTACTTTAGAAACGGACTCTGTACAAACGGACTAACATCATAGGCATATATCAAGACAAAT :  106
S.pennelli : CTGCACAATGTTCAATACTAGAACAATGCAGCAACTTTTACTTGTCTACTTTAGAAACAGACTCTGTACGAACGGACTAACATCATAGGCATATATCAAGACAAAT :  106
             CTGCACAATGTTCAATACTAGAACAATGCAGCAACTTTTACTTGTCTACTTTAGAAAC GACTCTGTAC AACGGACTAACATCATAGGCATATATCAAGACAAAT

                *       120         *       140         *       160         *       180         *       200         *
S.lycopers : AAAATAAGAGTACTAGCTAGGAACGTAACATTGTGCTATCATTTAGACACAACTAATTCAGTTACCAGGAGCAATAGGAACATCTTTGCTAACAACCACATTAGAA :  212
S.pennelli : AAAATTGGAGTA-TAGCTAAAAACGTAACATTGTGCTATCATTTAGACACAACTAATTCAGTTACCAGGAGCAATAGGAACATCTTTGCTAACAACCACATTAGAA :  211
             AAAAT  GAGTA TAGCTA  AACGTAACATTGTGCTATCATTTAGACACAACTAATTCAGTTACCAGGAGCAATAGGAACATCTTTGCTAACAACCACATTAGAA

                  220         *       240         *       260         *       280         *       300         *       3
S.lycopers : GTACCCTTTCCTTCTTCAATGTTGTTCTTCCACCCTTTGCTCTTTTCAAATTCCTTTGCTGTGTCATAAGAAGCATGCAGCCCGAAAAAGAAGTAATACGCCAAAA :  318
S.pennelli : GTACCATTTCCTTCTTCAATATTGTTCTTCCACCCTTTGCTCTTTTCGAATTCCTTTGCTGTGTCATAAGAAGCATGTAGCCCGAAAAAGAAGTAATACGCCAAAA :  317
             GTACC TTTCCTTCTTCAAT TTGTTCTTCCACCCTTTGCTCTTTTC AATTCCTTTGCTGTGTCATAAGAAGCATG AGCCCGAAAAAGAAGTAATACGCCAAAA

             20         *       340         *       360         *       380         *       400         *       420
S.lycopers : GAAAACCTGTCCAGCCAGCAAATCTGATGAACGAATCTTTATCTATCGAGCCTAGTAGGAAAATGTTGATAGCTATCGATAATGATGGCAACCAAGGCACAAGAGG :  424
S.pennelli : GAAAACCTGTCCAGCCAGCAAATCTGATGAACGAATCTTTATCTATCGAGCCTAGTAGGAAAATGTTGATAGCTATTGATAATGATGGCAACCAAGGCACAAGAGG :  423
             GAAAACCTGTCCAGCCAGCAAATCTGATGAACGAATCTTTATCTATCGAGCCTAGTAGGAAAATGTTGATAGCTAT GATAATGATGGCAACCAAGGCACAAGAGG

                  *       440         *       460         *       480         *       500         *       520         *
S.lycopers : CACACCCCAAAGCTTAGGATTACGAGCCTGAGGAATGAAAATACAAATCCCTGCAGTTGCTGAAACCCAAATAGGCACAGTGAAGCAGTATCCAATCCATCCTTTC :  530
S.pennelli : CACACCCCAAAGCTTAGGATTACGAGCCTGAGGAATGAAAATACAAATCCCTGCAGTTGCTGAAACCCAAATAGGCACAGTGAAGCAGTATCCAATCCATCCTTTC :  529
             CACACCCCAAAGCTTAGGATTACGAGCCTGAGGAATGAAAATACAAATCCCTGCAGTTGCTGAAACCCAAATAGGCACAGTGAAGCAGTATCCAATCCATCCTTTC

                    540         *       560         *       580         *       600         *       620         *
S.lycopers : TCACTAAGTCCCCAGTAAGCAGCTGTGGCGATTGAGGACCCGAGGATGAGCAATATGCATATAATTAGCTTGTTGCGGTTTGCGTTTGTGGTCACACCACTCACAT :  636
S.pennelli : TCACTAAGTCCCCAGTAAGCAGCTGTGGCGATTGAGGACCCGAGGATGAGCAATATGCATATAATTAGCTTGTTGCGGTTTGCGTTTGTGGTCACACCACTCACAT :  635
             TCACTAAGTCCCCAGTAAGCAGCTGTGGCGATTGAGGACCCGAGGATGAGCAATATGCATATAATTAGCTTGTTGCGGTTTGCGTTTGTGGTCACACCACTCACAT

              640         *       660         *       680         *       700         *       720         *       740
S.lycopers : AGTAACGGCGAACGAGGAGAGCGAGGGCCACGAGCATGAAGATGAAAAGGGTGGAGATCGACAAGAGATTGGAGAGAATTTCGAGCTTTGTGAAGAAGGCGATGAT :  742
S.pennelli : AGTAACGACGAACGAGGAGAGCGAGGGCCACGAGCATGAAGATGAAAAGGGTGGAGATTGACAAGAGATTGGAGAGAATTTCGAGCTTTGTGAAGAAGGCGATGAT :  741
             AGTAACG CGAACGAGGAGAGCGAGGGCCACGAGCATGAAGATGAAAAGGGTGGAGAT GACAAGAGATTGGAGAGAATTTCGAGCTTTGTGAAGAAGGCGATGAT

                    *       760         *       780         *       800         *       820         *       840
S.lycopers : TGCAGTAGCAGACATCATGACCGCTGTGGCATTGACGGGCGTCCCTGTTTTTTCATCAACATAGGAAAACCACGGAGGCATCATGTGAGTCCGCGCAATGTGTGTG :  848
S.pennelli : TGCAGTAGCAGACATCATGACCGCCGTGGCATTGACGGGCGTCCCTGTTTTTTCATCAACATAGGAAAACCACGGAGGCATCATGTGAGTCCGCGCAATGTGAGTG :  847
             TGCAGTAGCAGACATCATGACCGC GTGGCATTGACGGGCGTCCCTGTTTTTTCATCAACATAGGAAAACCACGGAGGCATCATGTGAGTCCGCGCAATGTG GTG

              *       860         *       880         *       900         *       920         *       940         *
S.lycopers : AGATAACGAGCCTGACCAACCGCGCCTACAAGCAACACTGAGGTCATTCCTTTCAACGCACCTGGAATTAGTGACAAAGTTAGAAATTTCGCTAAATGTGTTCAAA :  954
S.pennelli : AGATAACGAGCCTGACCAACCGCGCCTACAAGCAACACTGAGGTCATTCCTTTCAACGCACCTGCAATTAGTGACAAAGTCAGAAATTTCGCTAAATGTGTTCAAA :  953
             AGATAACGAGCCTGACCAACCGCGCCTACAAGCAACACTGAGGTCATTCCTTTCAACGCACCTG AATTAGTGACAAAGT AGAAATTTCGCTAAATGTGTTCAAA

                960         *       980         *      1000         *      1020         *      1040         *      1060
S.lycopers : ATTTTCACAAGTATGTAGCTCCGCCCCTACCTGCAGCTACAATATATTGCGCCCAACTCCATCCAACTGCTTTGAACGCCACAGAAAACGGAGCATTAGGATCAAT : 1060
S.pennelli : ATTTTCATGAGTATGTAGCTCCGCCCCTACCTGCAGCTACAATATATTGCGCCCAACTCCATCCAACAGCTTTGAACGCCACAGAAAACGGAGCATTAGGATCAAT : 1059
             ATTTTCA  AGTATGTAGCTCCGCCCCTACCTGCAGCTACAATATATTGCGCCCAACTCCATCCAAC GCTTTGAACGCCACAGAAAACGGAGCATTAGGATCAAT

                      *      1080         *      1100         *      1120         *      1140         *      1160
S.lycopers : GCTCTGATAAGGCTGCATAAGACAGAGTGTTATAGCCAATAAACAGTACACAGTAGTGGTGATCACCATAGATCCAACTAGGCCAATGGGGATATCTCGAGCTGGA : 1166
S.pennelli : GCTCTGATAAGGCTGCATAAGACAGAGTGTTATAGCCAATAAACAGTACACAGTAGTGGTGATCACCATAGATCCAACTAGGCCAATGGGGATATCTCGAGCTGGA : 1165
             GCTCTGATAAGGCTGCATAAGACAGAGTGTTATAGCCAATAAACAGTACACAGTAGTGGTGATCACCATAGATCCAACTAGGCCAATGGGGATATCTCGAGCTGGA

                *      1180         *      1200         *      1220         *      1240         *      1260         *
S.lycopers : TCCTTAGTTTCCTCAGCCATAGTCGAAACAGCATCAAAACCAACATAAGCAAAGAACAACACCGCAGAAGCTTTGAAAATGCCACGAACTCCAAATGGCGTAAAGG : 1272
S.pennelli : TCCTTAGTTTCCTCAGCCATAGTCGAAACAGCATCAAAACCAACATAAGCAAAGAACAACACCGCAGAAGCTTTGAAAATGCCACGAACTCCAAATGGCGTAAAGG : 1271
             TCCTTAGTTTCCTCAGCCATAGTCGAAACAGCATCAAAACCAACATAAGCAAAGAACAACACCGCAGAAGCTTTGAAAATGCCACGAACTCCAAATGGCGTAAAGG

                 1280         *      1300         *      1320         *      1340         *      1360         *      13
S.lycopers : GGGTGTAGTTCTTGGTATCTGCCTTGATTAGGCCACAGATGATGATGAAAACAATCACCACAATGTGAATGATCGAGGCAACATAGTTGAGACGAGAAGAGCCTTT : 1378
S.pennelli : GGGTGTAGTTCTTGGTATCTGCCTTGATTAGGCCACAGATGATGATGAAAACAATCACCAGAATGTGAATGATCGAGGCAACATAGTTGAGACGAGAAGAGCCTTT : 1377
             GGGTGTAGTTCTTGGTATCTGCCTTGATTAGGCCACAGATGATGATGAAAACAATCACCA AATGTGAATGATCGAGGCAACATAGTTGAGACGAGAAGAGCCTTT

             80         *      1400         *      1420         *      1440         *      1460         *      1480
S.lycopers : CGTACTACGAATGGCAATGATGCAGATGATGAAGCAGACCGCAACAGCGATTGGGTCAAGTTGGTTGTAGCCCTTAGCTAAACCATGAGCCGTGATGAGGAATTTT : 1484
S.pennelli : CGTACTACGAATGGCAATGATGCAGATGATGAAGCAGACCGCAACAGCGATTGGGTCAAGTTGGTTATAGCCCTTGGCTAAACCATGAGCCGTGATGAGGAATTTT : 1483
             CGTACTACGAATGGCAATGATGCAGATGATGAAGCAGACCGCAACAGCGATTGGGTCAAGTTGGTT TAGCCCTT GCTAAACCATGAGCCGTGATGAGGAATTTT

                  *      1500         *      1520         *      1540         *      1560         *      1580         *
S.lycopers : TCAGGCTTGAAGTTCAACAAAGTTGCAAAGTATGAGGTCCATGAACGAGCCACTGCAGCACCACCAATCACATATTCAAGAAGGATGTTTCCAGCAGCAATGAAGG : 1590
S.pennelli : TCAGGCTTGAAGTTCAACAAAGTTGCAAAATATGAGGTCCATGAACGAGCCACTGCAGCACCACCAATCACATATTCAAGAAGGATGTTTCCTGCAGCAATGAAGG : 1589
             TCAGGCTTGAAGTTCAACAAAGTTGCAAA TATGAGGTCCATGAACGAGCCACTGCAGCACCACCAATCACATATTCAAGAAGGATGTTTCC GCAGCAATGAAGG

                   1600         *      1620         *      1640         *      1660         *      1680         *
S.lycopers : CCACAAAGTCACCAAGCTCCACCCTCAAGTAGGCAAATGAACCACCTAAAATAATCATTTTAAAACTTAGTACATACTCCTTAATAAAAACTACATCAACATTGAT : 1696
S.pennelli : CCACAAAGTCACCAAGCTCCACCCTCAAGTAGGCAAATGAACCACCTAAAATAATCATTTTAAAACTTAGTACATACTCCTTAATAAAAACTACATCAACATTGTT : 1695
             CCACAAAGTCACCAAGCTCCACCCTCAAGTAGGCAAATGAACCACCTAAAATAATCATTTTAAAACTTAGTACATACTCCTTAATAAAAACTACATCAACATTG T

             1700         *      1720         *      1740         *      1760         *      1780         *      1800
S.lycopers : GAAATTATAATTAAGTAAATAAAAGTGTGATGTATACGTATTTTAAAAGTTTAAGCTTTTCCAAATACATAGACACATACACTTAAACAAACATTAATTTGCAGTT : 1802
S.pennelli : GAAATTATAATTAAGTAAATAAAAGTGTGATGTATATGTATTTTAAAAGTTTAAGCTTT-CCACATACATATACACATACACTTAAACAAACATTAATTTGCAGTT : 1800
             GAAATTATAATTAAGTAAATAAAAGTGTGATGTATA GTATTTTAAAAGTTTAAGCTTT CCA ATACATA ACACATACACTTAAACAAACATTAATTTGCAGTT

                    *      1820         *      1840         *      1860         *      1880         *      1900
S.lycopers : GCTGATTATAATTTCAACTTATCGAGCATATTCAACGAGTTGATCTGATCAATGCTTCATTTTAAAACCCTAACTGTTCATGTAGTTATAATAA------GCATGC : 1902
S.pennelli : GCTGATTATAATTTCAACTTATCGAGCATATTCAACGAGTTGATCTGATCAAGGCTTCATTTTAAACCCCTGACTGTTCATGTAGTAATAATAATAATTAGCATGC : 1906
             GCTGATTATAATTTCAACTTATCGAGCATATTCAACGAGTTGATCTGATCAA GCTTCATTTTAAA CCCT ACTGTTCATGTAGT ATAATAA      GCATGC

              *      1920         *      1940         *      1960         *      1980         *      2000         *
S.lycopers : ATGT--TAGCCAATGTTTTAATGAACAGAGTAGATGAGATTATACAATATTAATAAGACGTATTGAATGATTGCCAGTGATAAAACAATGAAATATAGTACAAAAT : 2006
S.pennelli : ATGTGTTAGCTAATGTTTTAATGAACAGAGTAGATGAGATTATACAATATTAATAAGACGTATTGAATGATTGCCAGTGATAAAACAATGAAATATAGTACAAAAT : 2012
             ATGT  TAGC AATGTTTTAATGAACAGAGTAGATGAGATTATACAATATTAATAAGACGTATTGAATGATTGCCAGTGATAAAACAATGAAATATAGTACAAAAT

               2020         *      2040         *      2060         *      2080         *      2100         *      2120
S.lycopers : GATTTATTTTGTCGATCATAATGTAGTATCTACATTTTACAATTAGTTCGAAATAATTCAGACAATAGTAAATTTACAAATTACAGATTAAATTATACCAAAAAAA : 2112
S.pennelli : GATTTATTTTGTCGATCATAATGTAGTATCTACATTTTACAATTAGTTCGAAATAATTCAGACAATAGTAAATTTACAAAATACAGATTAAATTATACCAAAAAAA : 2118
             GATTTATTTTGTCGATCATAATGTAGTATCTACATTTTACAATTAGTTCGAAATAATTCAGACAATAGTAAATTTACAAA TACAGATTAAATTATACCAAAAAAA
```

```
                      *      2140         *      2160         *      2180         *      2200         *      2220
S.lycopers : CAAAATTGATATTGAATGGGCGTGTGCACTGGGAATATAAATGTGCCAAAAGGGTAAAAAAATTCAAGCGGTCTAACAGTATAGGATTTTATACTAGAAGATATTA : 2218
S.pennelli : CAAAATTGACATTGAATGGGCGTGTGCACTGGGAATATAAATGTGCCAAAAGGGTAAAAAAATTCAAGAGGTCTAACAGTATAGGATTTTATACTGGAAGATAATA : 2224
             CAAAATTGA ATTGAATGGGCGTGTGCACTGGGAATATAAATGTGCCAAAAGGGTAAAAAAATTCAAG GGTCTAACAGTATAGGATTTTATACT GAAGATA TA

                *      2240         *      2260         *      2280         *      2300         *      2320         *
S.lycopers : ACTGATTCACATTTCTATCTTTTCTACTTAAATTAGAAAGATAATAACTGATCCAAATCTCTATCTTTTACTACTTAAATTAGAAGATAATAACTGATCCACATCT : 2324
S.pennelli : ACTAATTCACATCTCTATCTTTTCTACTTAAATTAGAA-GATTATAACTGATCCAAATCTCTATCTTTTTCTACTTAAATTAGAAGATAATAACTGATCCACATCT : 2329
             ACT ATTCACAT TCTATCTTTTCTACTTAAATTAGAA GAT ATAACTGATCCAAATCTCTATCTTTT CTACTTAAATTAGAAGATAATAACTGATCCACATCT

                 2340         *      2360         *      2380         *      2400         *      2420         *      24
S.lycopers : CTATCTTTTTCTACTTAAATATGAAGTTTTCGTATACGTTTTAATTTGTGACATGCACCTAATTTACTCAACATGATGTGTTTTACCACTAAAGCAAATTACATGA : 2430
S.pennelli : CTATCTTTTTCTACTTAAATATGAAGTTTTCGTATACGTTTTAATTTGTGACATGCACCTAATTTACTCAACATGATGTGTTTTACCACTAAAGCAAATTACATGA : 2435
             CTATCTTTTTCTACTTAAATATGAAGTTTTCGTATACGTTTTAATTTGTGACATGCACCTAATTTACTCAACATGATGTGTTTTACCACTAAAGCAAATTACATGA

             40         *      2460         *      2480         *      2500         *      2520         *      2540
S.lycopers : TGTGTTTTTACCAATAAAGCAAATGCTTCAAAATTTCAATCTCATGCTTACCAAAGTAAACAATATTCCACAGTTTTATCCTATGGTCGTCCCACAAAAAGGAGAA : 2536
S.pennelli : TGTGTTTTTACCAATAAAGCAAATGCTTCAAAATTTCAATCTCATGCTTCCCAAAGTAAACAATATTCCACAGTTTTATCCTATGGTCGTCCCACAAAAAGGAGAA : 2541
             TGTGTTTTTACCAATAAAGCAAATGCTTCAAAATTTCAATCTCATGCTT CCAAAGTAAACAATATTCCACAGTTTTATCCTATGGTCGTCCCACAAAAAGGAGAA

                  *      2560         *      2580         *      2600         *      2620         *      2640         *
S.lycopers : AAATTAACGAAAATCTATATGAACTCTGACCTAATTGTAAAATGACGTTATTGCATTGCAAAAATGTATCTTTTTCCTGAATTAATATATGAGAATAATTATAACT : 2642
S.pennelli : AAATCAACGAAAATCTATATGAACTCTGACCTAATTGTAAAATGACGTTATTGCATTGCAAGA-TGCATCTTTTTCCTGAATTAATATATGAGAATAATTATAACT : 2646
             AAAT AACGAAAATCTATATGAACTCTGACCTAATTGTAAAATGACGTTATTGCATTGCAA A TG ATCTTTTTCCTGAATTAATATATGAGAATAATTATAACT

                   2660         *      2680         *      2700         *      2720         *      2740         *
S.lycopers : TTATGGATATTTAGATTTTAAATTTGAAAATATCAAACTAATTTAATTCTAAAGAACAATTCT--------AAAATGTAACTAGAGATCCTAAAAGTCCAAAAATG : 2740
S.pennelli : TTATGGATATTTAGATTTTAAATTTGAAAATATCAAACTAATTTAATTCTAAAGAATAATTCTCATAGAGTAAAATGTAACTAGCGATCCTAAAAGTCCAAAAATG : 2752
             TTATGGATATTTAGATTTTAAATTTGAAAATATCAAACTAATTTAATTCTAAAGAA AATTCT        AAAATGTAACTAG GATCCTAAAAGTCCAAAAATG

             2760         *      2780         *      2800         *      2820         *      2840         *      2860
S.lycopers : CAGCCGAAAATATTACGTTAGCCTTGTTTTTCCAACCTGGAACGTTGAATCTTCAACCCTGGCCCCAGTACCCACTGCCATAACTGCTTAGTGGGGACTGCA-GAG : 2845
S.pennelli : CAGCCGAAAATATTACGTTAGCCTTGTGTTTCCAACCTGGAACGTTGAATCTTCAACCCTGGCCCCAGTACCCATTGCCATAACTGCTTAGTGGGGACTGCAAGAG : 2858
             CAGCCGAAAATATTACGTTAGCCTTGT TTTCCAACCTGGAACGTTGAATCTTCAACCCTGGCCCCAGTACCCA TGCCATAACTGCTTAGTGGGGACTGCA GAG

                    *      2880         *      2900         *      2920         *      2940         *      2960
S.lycopers : TGACTCAAATTTCTATTTTGCTGGCGCTGACTTTAGAAAAGGAAAATCACAATTTCACGACAATCCCACAACTTTT-GGTTCATTACATTTTCATATTGGGCTGTA : 2950
S.pennelli : AGACTCAAATTTCTATTTTGCTGGCGCTGACTTTAGAA-----ATTTCAGAACTTTTAGACAATTCCACAACTTTTTGGTTCATTACATTTTCATATTGGGCTGTA : 2959
              GACTCAAATTTCTATTTTGCTGGCGCTGACTTTAGAA     A  TCA AA TT   GACAAT CCACAACTTTT GGTTCATTACATTTTCATATTGGGCTGTA

              *      2980         *      3000         *      3020         *      3040         *      3060         *
S.lycopers : CACAACTCACACCGCTATAGTGATTGATTTGAACATAAGTTATAATAGGGTATTAAA--TATCAGCATCAATATTTCAAATGTTATAAAAAAAAGAACATATTATG : 3054
S.pennelli : CACAACTCACAACGTTACGGTGATTGATTTGAAGATAAGTTATGAT-GGGTATTAAAAATATCAACATCAATACTCCAAACGTAATAATCAAAAAAACGTATTATG : 3064
             CACAACTCACA CG TA  GTGATTGATTTGAA ATAAGTTAT AT GGGTATTAAA  TATCA CATCAATA T CAAA GT ATAA  AAAA AAC TATTATG

               3080         *      3100         *      3120         *      3140         *      3160         *      3180
S.lycopers : ATTTTT-GGGTACTAAAATTTTATCTTAGGATTATCTCCTTTAACCCATTACACCAAATGTGTCCACTTCATTTGGAAAAGGAAGAAATATCCAAACTTAATAGCT : 3159
S.pennelli : ATTTTTTGGGTACTAAAATTTTTTCTTAGGATTATCTCCTTTAACCCATTACACCAAATGTGTCCACTTCATTTGGAAAAGCAAGAAATATCCAAACTTAATAGCT : 3170
             ATTTTT GGGTACTAAAATTTT TCTTAGGATTATCTCCTTTAACCCATTACACCAAATGTGTCCACTTCATTTGGAAAAG AAGAAATATCCAAACTTAATAGCT

                      *      3200         *      3220         *      3240         *      3260         *      3280
S.lycopers : TCGTAACAACATACCCCTATTAAGTTAATGCCTTAGCTACAATTCACTAATTGTTTGGTTGGAAATTAATTATTTTTAGATTAATTAATTTAAAATTAGCTACTTT : 3265
S.pennelli : TCGTGACAACATACCCCTATTAAGTTAATGCCTTAGCTACCATTCACTAATTGTTTGGTTAGAAATTAATTA---------------ATTTAGAATTAGCTACTTT : 3261
             TCGT ACAACATACCCCTATTAAGTTAATGCCTTAGCTAC ATTCACTAATTGTTTGGTT GAAATTAATTA               ATTTA AATTAGCTACTTT

                *      3300         *      3320         *      3340         *      3360         *      3380         *
S.lycopers : GGGATAATTTATCTATCATGTGTATTAAATAGTTTATCACACTCCAATAATATAAATGATGGAATACGTTATCCCAATATAATAATCAAACAAGACACTATAATTT : 3371
S.pennelli : GAGATAATTTATTTACCATGTGTATTAAATAGTTTATCACACTGCAATAATATAAATGATGAGATATATTATTCGAATATAATAATCAAACAAAACACTATAATTT : 3367
             G GATAATTTAT TA CATGTGTATTAAATAGTTTATCACACT CAATAATATAAATGATG  ATA  TTAT C AATATAATAATCAAACAA ACACTATAATTT

                 3400         *      3420         *      3440         *      3460         *      3480         *      35
S.lycopers : TATCCTAGATAAAAAAAAATTCCATAACTATATACTCTTATCCCTCAAACCAATCCAACCGATCCCTAAGTATCAGCAACATGGTGGCCTGCAGAGAGCGACTGGG : 3477
S.pennelli : TATCTTAAATAAAAAAA---TCCATAACTATATACTTTTATCTCTCAAACCAACCGAACCGATCCCTAGTCATCAGGGACATGGTGACCTGCAGAGAGCGACTGGG : 3470
             TATC TA ATAAAAAAA   TCCATAACTATATACT TTATC CTCAAACCAA C AACCGATCCCTA   ATCAG  ACATGGTG CCTGCAGAGAGCGACTGGG

             00         *      3520         *      3540         *      3560         *      3580         *      3600
S.lycopers : CGTGGGATAATTTAATTAAGAGATGTAGACATTTTATTAATTATTTACTCCTACTTATTAGGATCCACTGTTTCAACTCCGTCAACTAAATAATAAACTTCAAACA : 3583
S.pennelli : CGTGGGATAATTTAATTAAGAGATGTAGACATTTTATTAATTATTTACTCCTACTTATTAGGATCCATTGTTTCAATTCGGTCAACTAAATAATAAACTTCAAACA : 3576
             CGTGGGATAATTTAATTAAGAGATGTAGACATTTTATTAATTATTTACTCCTACTTATTAGGATCCA TGTTTCAA TC GTCAACTAAATAATAAACTTCAAACA

                  *      3620         *      3640         *      3660         *      3680         *      3700         *
S.lycopers : TTTTTAAATATGAAAGGCAAATTATTGACAAATGCATGTGTACACTACCTAGCTGTTAATTTACTACAGTATTATTTTCATTTATACAACGACGAATCACTCTTAA : 3689
S.pennelli : TTTTTAAATATGAAAGGCAAATTATTGACAAATGCATGTGTACACTAGCTAGCTGTTAATTTACTACAGTATTATTTTCATTTATACAACGACGAAACACTCTTAA : 3682
             TTTTTAAATATGAAAGGCAAATTATTGACAAATGCATGTGTACACTA CTAGCTGTTAATTTACTACAGTATTATTTTCATTTATACAACGACGAA CACTCTTAA

                   3720         *      3740         *      3760         *      3780         *      3800         *
S.lycopers : TCCAAACTTTCTTTTATATTAATACCATC-CTGGCTAAGTAGAAGAAGAAACACACAAATACAGGCAAAAATAATAATAAAAAATTATGTTATAAACGTATAATGC : 3794
S.pennelli : TCCAAACTTTCTTTTATATTAATACCATCGCTGGCTAACTAGAAGAAGAAACACACAAATACAGGCAAAAATAAAAATAAAAAATTATGTTATAAACGTATAATGC : 3788
             TCCAAACTTTCTTTTATATTAATACCATC CTGGCTAA TAGAAGAAGAAACACACAAATACAGGCAAAAATAA AATAAAAAATTATGTTATAAACGTATAATGC

             3820         *      3840         *      3860         *      3880         *      3900         *      3920
S.lycopers : CCTTATTAATCAAACAGTTGTCACTATCTTTCAAGTCCCGTAAGTCCAACGGAGAACTTTGCATAATATTCACAAAATTTAACTTTTCGTCATAATAATAACTTTT : 3900
S.pennelli : CCTTAGTAATCAAACAGTTGTCACTATCTTTCAAGTCTCGTAAGTCCAACGGAGAACTTTGCATAATATTCACAAAATTTAACTTTTCGTCATAACAA-------- : 3886
             CCTTA TAATCAAACAGTTGTCACTATCTTTCAAGTC CGTAAGTCCAACGGAGAACTTTGCATAATATTCACAAAATTTAACTTTTCGTCATAA AA

                    *      3940         *      3960         *      3980         *      4000         *      4020
S.lycopers : CGTCGTAATAAATATCAACTTAAAATCAAAACTCCTT--GTACAGTTTCTTATAGAAAAATTAACTTTTATCTTGATAACAATTTACCTGCTTATGAAAAGTGATT : 4004
S.pennelli : -----------ATATCAACTTAAAATCAAAACTCCTTTTGTACAGTTTCTTATAGAAAAATTAACTTTTATCTTGACAACAATTTACCTGCTTATGAAAAGTGATT : 3981
                        ATATCAACTTAAAATCAAAACTCCTT  GTACAGTTTCTTATAGAAAAATTAACTTTTATCTTGA AACAATTTACCTGCTTATGAAAAGTGATT

              *      4040         *      4060         *      4080         *      4100         *      4120         *
S.lycopers : TTAAGAAAATAATATGTGTAAAATTTATTA----------CATAATCGTGCCTTATTCTCTAAGTTACTATCCCATAAGTGGAAAATTCTTATCCGAAGGTAATTT : 4100
S.pennelli : TTAAGAAAATAATATGTGTAAAATTTATTATATCATGTTACATAATCGTGCCTTATTCTCTAAGTTACTATTCCATAAGTGGAAAATTCTTATCTGAAGGTAACTT : 4087
             TTAAGAAAATAATATGTGTAAAATTTATTA          CATAATCGTGCCTTATTCTCTAAGTTACTAT CCATAAGTGGAAAATTCTTATC GAAGGTAA TT

               4140         *      4160         *      4180         *      4200         *      4220         *      4240
S.lycopers : GATCACGAAATCAACTTTCTC-------GAAGTTTCATGTTTTGAGGATAAAGCGAGAAAATATTTTTTAATCTTACATGACATGAATTTAAATTAATCAAAGGTT : 4199
S.pennelli : GATCACGAAATCAACTTTCTCTTAATTAGAAGTCTCGTGTTTTGAGAATAAAGCGTGAAAATATTTTTTAATCTTACATGACACGAATTTAAATGAATCAAAGGTC : 4193
             GATCACGAAATCAACTTTCTC       GAAGT TC TGTTTTGAG ATAAAGCG GAAAATATTTTTTAATCTTACATGACA GAATTTAAAT AATCAAAGGT

                      *      4260         *      4280         *      4300         *      4320         *      4340
S.lycopers : CAAAGCAACTGAATTTGAAAGAAAAGGGTTTAGACAAAGTCAAATTAATCGAGATTTCAAAACATACCTGCAACAGGGATCTCAACGGCAAACTCAGTGTAACAGA : 4305
S.pennelli : CAAAGCAACTGAATTTGAAAGAAAAGGGTTTAGACAAATTCAAATTAATCGAGATTTCAAAACATACCTGCAACAGGGATCTCAACGGCAAACTCAGTGTAACAGA : 4299
             CAAAGCAACTGAATTTGAAAGAAAAGGGTTTAGACAAA TCAAATTAATCGAGATTTCAAAACATACCTGCAACAGGGATCTCAACGGCAAACTCAGTGTAACAGA
```

```
                 *      4360          *      4380          *      4400          *      4420          *      4440          *
S.lycopers : AGACAGAGAGCAAGGCGGAGACGCCGGAAACTGCATATGACAGTACAACAGCAGGGCCAGCGTCCTGGCTCGCTTCAAGCCCCGTCAGTACGAAGATTCCTGCTCC : 4411
S.pennelli : AGACAGAGAGCAAGGCGGAGACGCCGGAAACTGCATATGACAGTACAACAGCAGGGCCAGCGTCCTGGCTCGCTTCAAGCCCAGTCAGTACGAAGATTCCTGCTCC : 4405
             AGACAGAGAGCAAGGCGGAGACGCCGGAAACTGCATATGACAGTACAACAGCAGGGCCAGCGTCCTGGCTCGCTTCAAGCCC GTCAGTACGAAGATTCCTGCTCC

                4460          *      4480          *      4500          *      4520          *      4540          *      45
S.lycopers : AATCACCGCTCCCATCCCAAACCATATTAGGTCCCACCATGTCAATGTCTTCTTCATTTCATGGTGACTCCGTGACTTCGCCTCCAGCTCTGCTTCATCATTCGAT : 4517
S.pennelli : AATCACCGCTCCCATCCCAAACCATATTAGGTCCCACCATGTCAATGTCTTCTTCATTTCATGGTGACTCCGTGACTTCGCCTCCAGCTCTGCTTCATCATTCGAT : 4511
             AATCACCGCTCCCATCCCAAACCATATTAGGTCCCACCATGTCAATGTCTTCTTCATTTCATGGTGACTCCGTGACTTCGCCTCCAGCTCTGCTTCATCATTCGAT

             60          *      4580          *      4600          *      4620          *      4640          *      4660
S.lycopers : CGCGTCAACGCCCGTTCGATTAACCTCGTCGGAGTTTGCCTCAGCGCATTCGTGTAATTTCCCCAGCTCTTGAACGATTCCTCCGGCAGAAAATCGTCTTTTGTGC : 4623
S.pennelli : CGCGTCAACGCCCGTTCGATTAACCTCGTTGGAGTTTGCCTCAGCGCATTCGCGTAATTTCCCCAGCTCTTGAACGATTCCTCCGGCAGAAAATCGTCTTTTGTGC : 4617
             CGCGTCAACGCCCGTTCGATTAACCTCGT GGAGTTTGCCTCAGCGCATTCG GTAATTTCCCCAGCTCTTGAACGATTCCTCCGGCAGAAAATCGTCTTTTGTGC

                  *      4680          *      4700          *      4720          *
S.lycopers : ACACACAATTTCTTCGCCTGATTCCTCCTCCTGATTCCGGTACTTCACCTTCTATCCCCATGTTCTTTCACTTT : 4697
S.pennelli : ACACACAATTTCTTCGCCTGATTCCTCCTCCTGATTCCGGTACTTCACTTTCTATCCCCATGCTCTTTCACTTT : 4691
             ACACACAATTTCTTCGCCTGATTCCTCCTCCTGATTCCGGTACTTCAC TTCTATCCCCATG TCTTTCACTTT
```

SGN-U242840: 4287 bp (S. lycopersicum) and 4335 bp (S pennellii)

```
                      *        20         *        40         *        60         *        80         *       100
S.lycopers : CATACAAGCTCAAGATGTATATCATTTGTGCTTTACTATAAGATTTATCGAA--TTCTCAAAATATAATGTGTAAATTCAGGTTGCCAACAAACTAGACGGAAGA :  103
S.pennelli : CATACAAGCTCAAGATGTAATTCATTTGTGCTTTACCATAAGATTTATCGAAAATTCCCAAAA-ATAATGTGTAAATTCAGGTTGCCAACAAACTAGACGGAAGA :  104
             CATACAAGCTCAAGATGTAWWTCATTTGTGCTTTACYATAAGATTTATCGAAAATTCYCAAAATATAATGTGTAAATTCAGGTTGCCAACAAACTAGACGGAAGA

                 *       120         *       140         *       160         *       180         *       200         *
S.lycopers : ACTTTTCGAGTTCCAATAATTTTTTACATTTTTTGTCAAACGCAAGTAGTCTTATTCAATTCACCACAAAAATATCAACTACTCGATTGCTCTTACAAATATTTT :  208
S.pennelli : ACTTTTTGAGTTCCAATAATATTTTACATTTTTTGTCAAACGCAAGTAGTCTTATTCAATTCACCACAAAAATATCAACTACTTGATTGCTGTTACAAATATTTT :  209
             ACTTTTYGAGTTCCAATAATWTTTTACATTTTTTGTCAAACGCAAGTAGTCTTATTCAATTCACCACAAAAATATCAACTACTYGATTGCTSTTACAAATATTTT

                     220         *       240         *       260         *       280         *       300         *
S.lycopers : GAGTGAAATCTCTTTTAAGAGATTATAAAACTACGTTAGGGGAGAAGTAGCTAGCAAAATA-ATCCCTCTTCCATTGAAGCCTTTTAAATTCTTACGAGCTGTAT :  312
S.pennelli : GAGTGAAATCTCTA--AAGAGATTATAAAACTACGTTAGGGGAGAAGTAGCTAGCAACATATATCCCTCTTCCATTGAAGCCTTTTAAATTCTTACGAGCTGTAT :  312
             GAGTGAAATCTCTWTTAAGAGATTATAAAACTACGTTAGGGGAGAAGTAGCTAGCAAMATATATCCCTCTTCCATTGAAGCCTTTTAAATTCTTACGAGCTGTAT

                320         *       340         *       360         *       380         *       400         *       420
S.lycopers : GACGAGCTGTAGAATTTTTTACTGTGTAGTGAATTACCTCTCTCTGCTGATACTAAGCTTATCAATCCAGTAATCAAGCTGACCAGCAACACATTCAGAACCTGT :  417
S.pennelli : GACGAGCTGTAGAATTTTTTACTGTGTAGTGAATTACCTCTCTCTGCTGATACTAAGCTTATCAATCCAGTAATCAAGCTGACCAGCAACACATTCGGAACCTGT :  417
             GACGAGCTGTAGAATTTTTTACTGTGTAGTGAATTACCTCTCTCTGCTGATACTAAGCTTATCAATCCAGTAATCAAGCTGACCAGCAACACATTCRGAACCTGT

                      *       440         *       460         *       480         *       500         *       520
S.lycopers : GGAGCCATATGACCTGAATTTTTTAATTGAATTTTCGACCCCTAGATATTCGTAGACATCCTTGTCAAATATCGGATGTAGACTGTTCAACTCGTCAAGGCTAAG :  522
S.pennelli : GGAGCCATATGACCTGAATTTTTTAATTGAATTTTCGACCCCTAGATATTCGTAGACATCCTTGTCAAATATCGGATGTAGACTGTTCAACTCATCAAGGCTAAG :  522
             GGAGCCATATGACCTGAATTTTTTAATTGAATTTTCGACCCCTAGATATTCGTAGACATCCTTGTCAAATATCGGATGTAGACTGTTCAACTCRTCAAGGCTAAG

                 *       540         *       560         *       580         *       600         *       620         *
S.lycopers : GTCTTGAAGCTCACAGTTTCTGGAAACACACAAAGCGACAGACCTCCCGACTATGTCATGAGAAGTTCTGAAAGGTATTCCCTGCAAGAAAATATCACAATTTCA :  627
S.pennelli : GTCTTGAAGCTCGCAGTTTTTGGAAACACACAAAGCGACAGACCTCCCAACTATGTCATGAGATGTTCTGAAAGGTATTCCCTGCAAGAAAATATCACAATTTCA :  627
             GTCTTGAAGCTCRCAGTTTYTGGAAACACACAAAGCGACAGACCTCCCRACTATGTCATGAGAWGTTCTGAAAGGTATTCCCTGCAAGAAAATATCACAATTTCA

                     640         *       660         *       680         *       700         *       720         *
S.lycopers : TCATTATTAAGACAT-TACCA--CATTTAAAAAGATTTAGTTCTTGAAGTAAGTGATCACTTGTGCAACAAACTTGGAATGAACCAAGAGGTTCTA----ACTAC :  725
S.pennelli : TCATTATTGAGACATGTAAGAGCTAATTAGAAGAATTTA--TTTTAAAGTAAGTGATCACTTGTGCAACAAACTTGATATGAACCAAGAGGTTTTACTGAGTTAC :  730
             TCATTATTRAGACATGTAMSAGCYAWTTARAARRATTTAGTTYTTRAAGTAAGTGATCACTTGTGCAACAAACTTGRWATGAACCAAGAGGTTYTACTGARYTAC

                740         *       760         *       780         *       800         *       820         *       840
S.lycopers : AAGTTTATTAGTCAACCCAGAGGTGTGTACTAATTTGTAAAGCCGTGGGGGGGGGGGGGTCAA----------------GTGACGTAGTTC------------- :  801
S.pennelli : AAGTTTCTTAGTCAACCCAGAGGTGTGTACTAATTTGTAAAGCCATGGGGTGGTTGATGAACAATTTGGGATTCCTAAAGGCAACTTGGTGCAAATAGTAACTTT :  835
             AAGTTTMTTAGTCAACCCAGAGGTGTGTACTAATTTGTAAAGCCRTGGGGKGGKKGRKGRWCAATTTGGGATTCCTAAAGGYRACKTRGTKCAAATAGTAACTTT

                      *       860         *       880         *       900         *       920         *       940
S.lycopers : -------TCACATCT-----------------------------GTTGACGTAA-------ATCCCA-------------------------------------- :  825
S.pennelli : TAGCATACCATATTTCAACAGATTAACTTTTATACTGCGTGTACATTGCCATAATGGTGGTGTTCCATTACAAAAATTTGGAAATCGTTCTCTAGCACAGAAAAC :  940
             TAGCATAYCAYATYTCAACAGATTAACTTTTATACTGCGTGTACRTTGMCRTAATGGTGGTRTYCCATTACAAAAATTTGGAAATCGTTCTCTAGCACAGAAAAC

                 *       960         *       980         *      1000         *      1020         *      1040         *
S.lycopers : ---------------------------------------------GTTTTCAAAGATAAAATCACTTGCCTTTTTCACAAGGTAATCAGCGAGTGTAGTGGCAT :  884
S.pennelli : CCATCTAATTATACGTCATTATAACTAGAGAAGGTACAAATCAAGGATTCTCGGAGTAGAAATCACTTGCCTTTTTCACGAGGTAATCAGCGAGTGTAGTAGCAT : 1045
             CCATCTAATTATACGTCATTATAACTAGAGAAGGTACAAATCAAGGRTTYTCRRAGWWRAAATCACTTGCCTTTTTCACRAGGTAATCAGCGAGTGTAGTRGCAT

                    1060         *      1080         *      1100         *      1120         *      1140         *
S.lycopers : CAAGATGACCAGCTGGTAGTGCTTTTTGTATCCTCTCTCGGTTAAAGGAAACATTCTGTGCAAACTCTGCTGAGACTTCAAGCATCCCAACTATTGCCTTCACAC :  989
S.pennelli : CAAGATGACCAGCTGGTAGAGCTTTTTCTATTCTCTCTCGGTTAAAGGAAACATTCTGTGCAAACTCTGCTGAGACCTCGAGCATCCCAACTATTGCCTTGACAC : 1150
             CAAGATGACCAGCTGGTAGWGCTTTTTSTATYCTCTCTCGGTTAAAGGAAACATTCTGTGCAAACTCTGCTGAGACYTCRAGCATCCCAACTATTGCCTTSACAC

              1160         *      1180         *      1200         *      1220         *      1240         *      1260
S.lycopers : TATCAAATACGGGCTCCTTATCTTCCTATTTAATATAAAAACGAGGTTCAATATATAAGACTTAAAATAATGCAGAGACCGAAAATGTTGATAAAGATAGTGAAG : 1094
S.pennelli : TGTCAAATACAGGCTCCTTATCTTCCTATTTAATATAAAAA-----------------GAGTTAAA------------CAGAAAATGTTGATAAAGATACTGAAG : 1226
             TRTCAAATACRGGCTCCTTATCTTCCTATTTAATATAAAAACGAGGTTCAATATATAAGASTTAAAATAATGCAGAGACMGAAAATGTTGATAAAGATASTGAAG

                      *      1280         *      1300         *      1320         *      1340         *      1360
S.lycopers : CAGAAAAA-ATATCGGAACTAATTGGGG-TCGCCTTGCTGTTAAGCAGTTAATGAAAATGTATATATGTGTG--CAGAGGGAAG----GGTGGTGAATTTCCTCG : 1191
S.pennelli : CAGGAAAACATAACAGAATTAATTGGGGGTCGCCTTGCTGTTAAGCAGTTAATGAAAATGTATATATGTGTGTGCACAGGGAAGTTGTAGTGGTGAATTTCCACA : 1331
             CAGRAAAACATAWCRGAAYTAATTGGGGGTCGCCTTGCTGTTAAGCAGTTAATGAAAATGTATATATGTGTGTGCASAGGGAAGTTGTRGTGGTGAATTTCCWCR

                 *      1380         *      1400         *      1420         *      1440         *      1460         *
S.lycopers : GAAGGAAGTGAATTAACTGATAAAGCATCTCTTTAGAGCATTTAGACTACTGTTGATTTATAGGCTTTCCAGGAAAATAACTATAACCTGCAAATCGCGATTGTA : 1296
S.pennelli : GAAGGAAGTAAATTAACTGATAAAGCATCTTTTCTGTGCATT-AGACTACTGTTGATTTATAGGCTTTCCAAGAAAGTACTTTTAACCTGCAAATCGCGATTGTA : 1435
             GAAGGAAGTRAATTAACTGATAAAGCATCTYTTYWGWGCATTTAGACTACTGTTGATTTATAGGCTTTCCARGAAARTAMYTWTAACCTGCAAATCGCGATTGTA

                    1480         *      1500         *      1520         *      1540         *      1560         *
S.lycopers : AGCATGAGGAAGTCCCTTGCACAACACAAGAAGTGAAACTAAGTCCCCTATGACTCTAGCAGATTTTCCACGAACAAGCTCCATTGGATCTGGATTCTTCTTTTG : 1401
S.pennelli : AGCATGAGGAAGTCCCTTGCACAACACAAGAAGTGAAACTAAGTCCCCTATGACTCTAGCAGATTTTCCACGAACAAGCTCCATTGGATCTGGATTCTTCTTTTG : 1540
             AGCATGAGGAAGTCCCTTGCACAACACAAGAAGTGAAACTAAGTCCCCTATGACTCTAGCAGATTTTCCACGAACAAGCTCCATTGGATCTGGATTCTTCTTTTG

              1580         *      1600         *      1620         *      1640         *      1660         *      1680
S.lycopers : GGGCATTATACTGCTTCCAGTGGAAACTTTATCACTAGGGGTTAAGAAACCAAATTCTTCGGATGCCCACAATACCCATTCTTCACCCAGGCGAGAGAGATGAAC : 1506
S.pennelli : GGGCATTATACTGCTTCCAGTGGAAACTTTATCACTAGGGGTTAAGAAGCCAAATTCTTCGGATGCCCACAATACCCATTCTTCACCCAGGCGAGAGAGATGAAC : 1645
             GGGCATTATACTGCTTCCAGTGGAAACTTTATCACTAGGGGTTAAGAARCCAAATTCTTCGGATGCCCACAATACCCATTCTTCACCCAGGCGAGAGAGATGAAC

                      *      1700         *      1720         *      1740         *      1760         *      1780
S.lycopers : AGCAGTGATAGAATTAGCAGAAAGTAACTCCAGCACAAAATCTCGATCTGAAACTGCATCAATGCTATTGCATATAAACATTGTCACCATGATTGAAAAGGCCAT : 1611
S.pennelli : AGCAGTGATGGAATTAGCAGAAAGTAACTCCAGCACAAAATCTCGATCTGAAACTGCATCAATGCTATTGCACATAAACATTGTCACCATGATTGAAAAGGCCAT : 1750
             AGCAGTGATRGAATTAGCAGAAAGTAACTCCAGCACAAAATCTCGATCTGAAACTGCATCAATGCTATTGCAYATAAACATTGTCACCATGATTGAAAAGGCCAT

                 *      1800         *      1820         *      1840         *      1860         *      1880         *
S.lycopers : ATGTAATTGCCAGAA-GAAATTAATGCAAATGCAGATTAATTACACATAAAGTGATACTACTATACATGATATCAGAGGGTAAAAGCAAA-TGATTACAACAGAA : 1714
S.pennelli : ATGTAATTGTCAGAAAGAAATTAATGCAAATGCAGATTAACCACACA--GAGTGCTAGTACTATACATGATATCAGAGGGTACACGCAAAATGATTACAACAGAA : 1853
             ATGTAATTGYCAGAAAGAAATTAATGCAAATGCAGATTAAYYACACATARAGTGMTASTACTATACATGATATCAGAGGGTAMAMGCAAAATGATTACAACAGAA

                    1900         *      1920         *      1940         *      1960         *      1980         *
S.lycopers : GCATGAGACAAGACTGTAACATTAAGGATTTCTTCTTCCAATTCACCCTTTTTATAAAATGAGACTAGTAGCAAAATAGTATTTTTCTTCCAAATAATATTCAGT : 1819
S.pennelli : GCATGAGACAAGACTGTAACATTAAGGATTTCTTCTTCCAATTAATCCTTTTTCAAAAACGAAACTAGTACCAAAATAGTATCTT-CTTCCAAATAATCTTCAGG : 1957
             GCATGAGACAAGACTGTAACATTAAGGATTTCTTCTTCCAATTMAYCCTTTTTMWAAAAYGARACTAGTASCAAAATAGTATYTTTCTTCCAAATAATMTTCAGK

              2000         *      2020         *      2040         *      2060         *      2080         *      2100
S.lycopers : GAACACACAATCATTAGTTTACAGACATCAATTAGCTTGAGAGTCGAGACTTAAGATCATCAGAGCCAAATTTGGAGGAAATATGTTTCTATGAGTTAGGCTATG : 1924
S.pennelli : GAACACACAATCATTAGTTTACTGACATCAATTAGCTTGAGAGTCGAGACTTAAGCTCATCAGGGCCAAATATGGAGGCAATAGGTTTCTATGAGTTAGGCTATG : 2062
             GAACACACAATCATTAGTTTACWGACATCAATTAGCTTGAGAGTCGAGACTTAAGMTCATCAGRGCCAAATWTGGAGGMAATAKGTTTCTATGAGTTAGGCTATG
```

```
                      *      2120         *      2140         *      2160         *      2180         *      2200
S.lycopers : CTATGATTTTGTAGTTCTCTTCACGGTTATTAATACAAAAGTTGGTTATTTACCCCAAAAAGGTTATATCTTGATTCACTAAAAACAAGAAGTAGCACAATACAC : 2029
S.pennelli : CTGTGATCTTGTAATTCTCTTCACAGTTATTAATACAAAAAATGGTTATTTACCCAAAAAAGGGT---------------------------------------- : 2127
             CTRTGATYTTGTARTTCTCTTCACRGTTATTAATACAAAARWTGGTTATTTACCCMAAAAAGGKTATATCTTGATTCACTAAAAACAAGAAGTAGCACAATACAC

                 *      2220         *      2240         *      2260         *      2280         *      2300         *
S.lycopers : CTTCTCCTCACAACCCTTTCTAAAGAAAACTAAAGAAAACAAAACCACAGTCTGTTGCAAGTTCGATTTGTATTAAGAATATAATTAGATGAAATTACTTGT-CA : 2133
S.pennelli : -----------------TTCTAAAGAAAAGTAAACGGAACGAATACACAGTCTGTTGCAAGTTCCATTGGTATTAAGCATATAATTAGATGAAATAAATTGTATA : 2215
             CTTCTCCTCACAACCCTTTCTAAAGAAAASTAAASRRAACRAAWMCACAGTCTGTTGCAAGTTCSATTKGTATTAAGMATATAATTAGATGAAATWAMTTGTAYA

                 2320         *      2340         *      2360         *      2380         *      2400         *
S.lycopers : TTAGTACCTGTTCCTCATGGGAGCAGTAAATCCCAATGCATCAGACGTCATAAACCTATCAATGGGAAGACCAGTGCCAGCTAATGCACATGCACCTAGAGGGCA : 2238
S.pennelli : TTAGTACCTGTTCCTCATGGGGGCAGTGAATCCCAATGCATCAGAAGTCATAAACCTATCAATGGGAAGACCAGTGCCAGCTAATGCACATGCACCTAGAGGGCA : 2320
             TTAGTACCTGTTCCTCATGGGRGCAGTRAATCCCAATGCATCAGAMGTCATAAACCTATCAATGGGAAGACCAGTGCCAGCTAATGCACATGCACCTAGAGGGCA

              2420         *      2440         *      2460         *      2480         *      2500         *      2520
S.lycopers : GAAATTCACCCTTGCTCTGCAATCTAATAAGCGACCAGCATCACGCTCAAGCTGCAAAGAAATAATTCTTTTTCCGTCAGCAACATATAGGTTTAATATTCTTAA : 2343
S.pennelli : GAAATTCACCCTTGCTCTACAATCTAATAAGCGACCAGCATCACGCTCAAGCTGCAATGAAATTGTTCTTCTGTCGTCAGCAATATGTAGGTTTAATATTCTTGA : 2425
             GAAATTCACCCTTGCTCTRCAATCTAATAAGCGACCAGCATCACGCTCAAGCTGCAAWGAAATWRTTCTTYTKYCGTCAGCAAYATRTAGGTTTAATATTCTTRA

                      *      2540         *      2560         *      2580         *      2600         *      2620
S.lycopers : GCTCTAGCACAGCCCAATGAAGCCACTAAACTAGCAGAAAGGGACAATTGGGATCCAAAGCCAAGAAGAA-CATGATTGCTAGATGATAAGATGACAAAAAGATA : 2447
S.pennelli : GCTCCAGTGCAGCTCAATGAAACCATTGAAATAGCAGAAAGGGACACATTGGGTCCAAAGCAAAGAAGAAATATGGTTGCTAAATGATAAGGTGAAAAACAGATA : 2530
             GCTCYAGYRCAGCYCAATGAARCCAYTRAAMTAGCAGAAAGGGACAMWTKGGRTCCAAAGCMAAGAAGAAAYATGRTTGCTARATGATAAGRTGAMAAAMAGATA

                 *      2640         *      2660         *      2680         *      2700         *      2720         *
S.lycopers : AAGTGAGTGCAGAAAGAAGTGAGAAAGCAAAGTTCTTTCTAAATATTCAAATAAACAAAAACAAACAAATGAATAAAAGGAAATATATAGAAGGAAGAACGGAAA : 2552
S.pennelli : ATGCGAGCGCAGAAAAAAAGTGA-AAAGCAAAGTTCTTTCTAAATATTCAAATAAATAAAAA--------------AAAGG-------TAGAAGAAAGAACAAAAA : 2613
             AWGYGAGYGCAGAAARAAGTGAGAAAGCAAAGTTCTTTCTAAATATTCAAATAAAYAAAAACAAACAAATGAATAAAAGGAAATATATAGAAGRAAGAACRRAAA

                 2740         *      2760         *      2780         *      2800         *      2820         *
S.lycopers : A-GGAACCAACAAA-AACTATGGTCGAAACAATATGCATAATGCACATCAAGAGAAAACCAGATACTCCTTTCACAGAAATTACATTGTGTAATTATATTGAGCA : 2655
S.pennelli : AAGGGAACAACAAATAACGATGGTTGAAACAATACGCA-------CATCAAGAGAAAACCAGATACTCCTATCACAGAAAT-ATAGCTTGCAATTATGTTGAGGA : 2710
             AAGGRAMCAACAAATAACKATGGTYGAAACAATAYGCATAATGCACATCAAGAGAAAACCAGATACTCCTWTCACAGAAATTAYAKYKTGYAATTATRTTGAGSA

              2840         *      2860         *      2880         *      2900         *      2920         *      2940
S.lycopers : TCTAAATGCAACAACCAAGTTACAGCTG-CACTTCCGAATAGAGCAAAGAGTCATTCAACAGACAAATATGAGAAAATGCTCTATCTGGAAAAGTCATTGCAAA- : 2758
S.pennelli : CCGAAATGCAACATGAAAGTTACAGGTGACACTTGCGAACAGAGCAAAGTGTCCTTAAACAGACAAATATGAGAAAATGCTCCATCTCGATA-GTCATTGCAAAA : 2814
             YCKAAATGCAACAWSMAAGTTACAGSTGACACTTSCGAAYAGAGCAAAGWGTCMTTMAACAGACAAATATGAGAAAATGCTCYATCTSGAWAAGTCATTGCAAAA

                      *      2960         *      2980         *      3000         *      3020         *      3040
S.lycopers : TGTTTTCTATTGAAAAATCTAATACCCCTTAAGTAAGATAATTAAAATGGACAAGTTTCTACTAACTAAACATACTAGAGGTCGGTGAGATAAGAAGACATTGAA : 2863
S.pennelli : TGTTTTCTCTTGAAAGATCTAATACCCCGTTAGTAAGATGATTCAAATGGACTAATT------AATTAAACATACTAGAAGTCAGTGAGGTAAGAAGACATTGAA : 2913
             TGTTTTCTMTTGAAARATCTAATACCCCKTWAGTAAGATRATTMAAATGGACWARTTTCTACTAAYTAAACATACTAGARGTCRGTGAGRTAAGAAGACATTGAA

                 *      3060         *      3080         *      3100         *      3120         *      3140         *
S.lycopers : TTTCAAGGAAAAGGTATGTAAAAGCTTGTGGTATTGTTTGAGGCTCCACTTGTCAC------------------------TCTTTTGATGCATAAACGAGTGTTT : 2944
S.pennelli : TTTCAAGGGAACGGGATGTAAAAGCCTGTGGTATTGTCTGAGGCTCCACTTGCCACAAGAAATCGCTTATTTTTTCCATCTCTTTTGATGCATAAACAACCATTT : 3018
             TTTCAAGGRAAMGGKATGTAAAAGCYTGTGGTATTGTYTGAGGCTCCACTTGYCACAAGAAATCGCTTATTTTTTCCATCTCTTTTGATGCATAAACRASYRTTT

                 3160         *      3180         *      3200         *      3220         *      3240         *
S.lycopers : CAAGTGATCTTCACTTCATCATGCTTCATGTGATAATAAAATGATGTAC-AAGTGTGGGAAGGAGGTAGCAGGAACCCGGTACAATAGTCCAGGTGTGCCCAAGC : 3048
S.pennelli : CAAGCGATCTTCAATTCCTCGGGCTTCATATGATAATAAGATGATGCATTAAGTGTTAAAAGGAAGTAGCAGCTACCCGGAAGAATTGTCCAGGCGTGTGC---- : 3119
             CAAGYGATCTTCAMTTCMTCRKGCTTCATRTGATAATAARATGATGYAYTAAGTGTKRRAAGGARGTAGCAGSWACCCGGWASAATWGTCCAGGYGTGYSCAAGC

              3260         *      3280         *      3300         *      3320         *      3340         *      3360
S.lycopers : TGACTAGACATCACCAGTAAAATTATATAAAAAAAATGATATGTTAAGTATAGAACATTAATCAATGAAGTCAGCCAAATTCTGCTAACCTGTTCAACATATGCT : 3153
S.pennelli : ------GCCATTAT-----AAAATAAATAAATTAAATGATGTGTTAAGAATAAGGCATTA-TCAATGAAGTCGGCCAAATTCTGCTAACCTGTTCAACATATGCT : 3212
             TGACTAGMCATYAYCAGTAAAAWTAWATAAAWWAAATGATRTGTTAAGWATARRRCATTAATCAATGAAGTCRGCCAAATTCTGCTAACCTGTTCAACATATGCT

                      *      3380         *      3400         *      3420         *      3440         *      3460
S.lycopers : AAGAGAAGGTGTTGCAACAGAACAGGTTGGGCCCTTTGCAGATGAGTGTATCCAGGAACAATAAGATCCTCATTTTGGAGAGCTAATTCAACCATTGCAATCTGC : 3258
S.pennelli : AAGAGAAGGTGTTGCAACAGAACAGGTTGGGCCCTTTGCAGATGAGTGTATCCAGGAACAATAAGATCCTCATTTTGGAGAGCTAATTCAACCATTGCAATCTGC : 3317
             AAGAGAAGGTGTTGCAACAGAACAGGTTGGGCCCTTTGCAGATGAGTGTATCCAGGAACAATAAGATCCTCATTTTGGAGAGCTAATTCAACCATTGCAATCTGC

                 *      3480         *      3500         *      3520         *      3540         *      3560         *
S.lycopers : AGGTTATGTTGCAATAAACGAATGAGTGCATAATAACCTTTAAGCAACACACAAATTGATCAACTTTGCTCCGACTTCACAATTTTATCCCATAATATGAAAAAC : 3363
S.pennelli : AGGTTATGTTGCAATAAACACATGAGTGCATAATAACCTTTAAGCAACATGTAAATTCATAAACTTTGCTTGGATTTCACAATTTTATCCCATAATATGAAAAAC : 3422
             AGGTTATGTTGCAATAAACRMATGAGTGCATAATAACCTTTAAGCAACAYRYAAATTSATMAACTTTGCTYSGAYTTCACAATTTTATCCCATAATATGAAAAAC

                 3580         *      3600         *      3620         *      3640         *      3660         *
S.lycopers : ACGATACACTATATTCATGTTAGCTGATCAAATACAGGAAAAAAGTGGATATTGCATTGAACTTCCAAACGATGATAACACAAAACAAAAAGCAAATGACCTGAA : 3468
S.pennelli : ACTATACACTAAATTCATGTTTGCTGATCAAATACAGGAAAAAAGTGGATATTGCATTGAACTTCCAAATGATGATAACACAAAACAAAAAGCAAATGACCTGAA : 3527
             ACKATACACTAWATTCATGTTWGCTGATCAAATACAGGAAAAAAGTGGATATTGCATTGAACTTCCAAAYGATGATAACACAAAACAAAAAGCAAATGACCTGAA

              3680         *      3700         *      3720         *      3740         *      3760         *      3780
S.lycopers : GATTCTTAATGCATGAAATGATTCCGTCAATAGCATCACGACACCACAAGCGAAAATCAGTTGCAACCTGATCATTTCTACTCCGGGCAGTGTGAAGCTTTTTGG : 3573
S.pennelli : GATTCCTAATGCGTGAAATGATTCCGTCAATAGCATCACGACACCACAAGCGAAAATCAGTTGCAACCTGATCATTTCTACTCCGGGCAGTGTGAAGCTTTTTGG : 3632
             GATTCYTAATGCRTGAAATGATTCCGTCAATAGCATCACGACACCACAAGCGAAAATCAGTTGCAACCTGATCATTTCTACTCCGGGCAGTGTGAAGCTTTTTGG

                      *      3800         *      3820         *      3840         *      3860         *      3880
S.lycopers : CAGGTTCACCAACCAAATCAGTAAGTGCTGCTTCAATATTCATATGCACATCCTCTCTATCTGTTCTCCATACAAATTCACCTGCCTCAATTTGCCTCTCTATCT : 3678
S.pennelli : CAGGTTCACCAACCAAATCAGTAAGTGCTGCTTCAATATTCATATGCACATCCTCTCTATCCGTTCTCCATACAAATTCACCTGCCTCAATTTGCCTCTCTATCT : 3737
             CAGGTTCACCAACCAAATCAGTAAGTGCTGCTTCAATATTCATATGCACATCCTCTCTATCYGTTCTCCATACAAATTCACCTGCCTCAATTTGCCTCTCTATCT

                 *      3900         *      3920         *      3940         *      3960         *      3980         *
S.lycopers : CTTCAAGACCTTTTATGATAGTATCTCTATCAATATCACTCATCAATCCCTGCAACATTATTGCAAATTCACATACATTAGAAACACCC-TTTATCCTCATTAGA : 3782
S.pennelli : CCTCAAGACCTTTTATGATAGTATCTCTATCACTATCACTCATCAATCCCTGCAA-----------ATTCACATGCATAAGAAACACCCATTTATCCTCATTAGA : 3831
             CYTCAAGACCTTTTATGATAGTATCTCTATCAMTATCACTCATCAATCCCTGCAACATTATTGCAAATTCACATRCATWAGAAACACCCATTTATCCTCATTAGA

                 4000         *      4020         *      4040         *      4060         *      4080         *
S.lycopers : ACAAAATGTCACAATTTAAACTGCTAATACTGATTGTTTGTAAATATAAGAAAACCCCTTAATGAAAAGAACCAATCTTGGCAATACCCAGAAACGAAAAACGTA : 3887
S.pennelli : A-AAAAAAACACAATTCAAACTGCTAATATTGATTGTTTGTAAATATAAGAAAACCCCTTAATGAAAAGAACCAATCTTGGCAATACCCAGAAACGAAAAACGTA : 3935
             ACAAAAWRWCACAATTYAAACTGCTAATAYTGATTGTTTGTAAATATAAGAAAACCCCTTAATGAAAAGAACCAATCTTGGCAATACCCAGAAACGAAAAACGTA

              4100         *      4120         *      4140         *      4160         *      4180         *      4200
S.lycopers : TAAATTAACCTGTTTAGCAAGCATTGAAGCATGAGCTTTACTACCCATGATATCCTGTTTATAGAGAGCTTTATCAAACGAAATGGACTCAGTGAACTTCTCCAC : 3992
S.pennelli : TAAATTAACCTGTTTAGCAAGCATTGAGGCATGAGCTTTACTACCCATGATATCCTGTTTATAGAGAGCTTTATCAAACGAAATGGACTCAGTGAACTTCTCCAC : 4040
             TAAATTAACCTGTTTAGCAAGCATTGARGCATGAGCTTTACTACCCATGATATCCTGTTTATAGAGAGCTTTATCAAACGAAATGGACTCAGTGAACTTCTCCAC
```

```
                    *      4220         *      4240         *      4260         *      4280         *      4300
S.lycopers : AGCATCGGTAACGCTCTCTTCGAAACGGCCACCCCATAGCTTCACTTCCTTTTCCGCCGTCGTCGCCGCCGTAGAGGGTGGTGAATTCATCTGCTGCGCTGCACA : 4097
S.pennelli : AGCATCGGTAACGCTCTCTTCGAAACGGCCACCCCATAGCTTCACCTCCTTTTCCGCCGTCTTCGCCGCCGTAGAGGGTGGTGAATTCATCTGCTGCGCTGCACA : 4145
             AGCATCGGTAACGCTCTCTTCGAAACGGCCACCCCATAGCTTCACYTCCTTTTCCGCCGTCKTCGCCGCCGTAGAGGGTGGTGAATTCATCTGCTGCGCTGCACA

                *      4320         *      4340         *      4360         *      4380         *      4400         *
S.lycopers : GGTGAACAATACTAAACGAGCATTGGAGTTGAAAGAGAGGCTTTGGGGTTTAGGAGTAAGGGCGTGTTTGGATGAAGAGAAAATGGGGGAAGTTGTCAAAGAAGA : 4202
S.pennelli : GGTGAACAATACTAAACGAGCATTGGAGTTGAAAGAGAGGCTCTGGGGTTTAGGAGTAAGGGCGTGTTTGGATGAAGAGAAAATGGGGGAAGTTGTCAAAGAAGA : 4250
             GGTGAACAATACTAAACGAGCATTGGAGTTGAAAGAGAGGCTYTGGGGTTTAGGAGTAAGGGCGTGTTTGGATGAAGAGAAAATGGGGGAAGTTGTCAAAGAAGA

                   4420         *      4440         *      4460         *      4480         *
S.lycopers : AGATGAAGAAATTAACAGTGACTCCATTGACGGCGAACAAATATCTGTGAAGAAGATGAAGACGAAGGGTAGAATTATATGTATT : 4287
S.pennelli : AGATGAAGAAATTAACAGTGACTCCATTGACGGCGAACAAATATCTGTGAAGAAGATGAAGACGAAGGGTAGAATTATATGTATT : 4335
             AGATGAAGAAATTAACAGTGACTCCATTGACGGCGAACAAATATCTGTGAAGAAGATGAAGACGAAGGGTAGAATTATATGTATT
```

SGN-U217186: 8442 bp (S. lycopersicum) and 8452 bp (S pennellii)

```
                      *        20         *        40         *        60         *        80         *       100
S.lycopers : TAAAACTTATAATTTAAAAGAAATCATATATATTTATATGAATATCAATGATAGAAAAGTATCATTCTGTTTTAGACTAACTAAAAGAGTGTCAAGTGTACTTAA :  105
S.pennelli : TAAAACTTATAATTTAAAATAAATCATATATATTTATATGAATATCAATGATAGAAAAGTATCATTCTGTTTTAGACTAACTAAAAGAGTGTCAAGTGTACTTAA :  105
             TAAAACTTATAATTTAAAA AAATCATATATATTTATATGAATATCAATGATAGAAAAGTATCATTCTGTTTTAGACTAACTAAAAGAGTGTCAAGTGTACTTAA

                 *       120         *       140         *       160         *       180         *       200         *
S.lycopers : AATTACCAAAATTAAGATCAGAAAACTTCCAAGTCTGAAGACTTAAACAAGGGATGAGCTTCAAACTATCTCTGTATAACAATCTTCATCAATGTTTAATTCTTC :  210
S.pennelli : AATTACCAAAATTAAGATCAGAAAACTTC-AAGTCTGAAGACTTAAACAAGGGATGAGCTTCAAACTATCTCTGTATAACAATCTTCATCAATGTTTAATTCTTC :  209
             AATTACCAAAATTAAGATCAGAAAACTTC AAGTCTGAAGACTTAAACAAGGGATGAGCTTCAAACTATCTCTGTATAACAATCTTCATCAATGTTTAATTCTTC

                   220         *       240         *       260         *       280         *       300         *
S.lycopers : TAGTAAAGATTTACGTATTAGCTGGTCTAACCCAATACATGATGAACACGGAAC-GGAAAATGTAGACTTCATCACAACTACCAACTTCTTACAAAATTTGGATC :  314
S.pennelli : TAGTAAAGATTTACGTATTAGCTGGTCTAACCCAATACATGATGAACACGGAAGAGGAAAATGTAGACTTCATCACAACTACCAACTTCTTACAAAATTTGGATC :  314
             TAGTAAAGATTTACGTATTAGCTGGTCTAACCCAATACATGATGAACACGGAA  GGAAAATGTAGACTTCATCACAACTACCAACTTCTTACAAAATTTGGATC

               320         *       340         *       360         *       380         *       400         *       420
S.lycopers : GACCAATCATCTGCAATACGCACATTAGGCTTGCGAATGGTTGGAACAGATCCTCGATTTCCTTGAATGAAGAGTGGCCCAAGCTCAATTTCCACTTTCTTATTT :  419
S.pennelli : GACCAATCATCTGCAATACGCACATTAGGCTTGCGAATGGTTGGAACAGATCCTCGATTTCCTTGAATGAAGAGTGGCCCAAGCTCAATTTCCACTTTCTTATTT :  419
             GACCAATCATCTGCAATACGCACATTAGGCTTGCGAATGGTTGGAACAGATCCTCGATTTCCTTGAATGAAGAGTGGCCCAAGCTCAATTTCCACTTTCTTATTT

                      *       440         *       460         *       480         *       500         *       520
S.lycopers : CCTGGTTCAATAATGGCTGCTTTAGCTCCAGGGGACAATCCTAACAAGATTATCCTCTCTACAGTGCACTCGGAAGAAAATGTGTCACTGCCAGCTGTAGACGGG :  524
S.pennelli : CCTGGTTCAATAATGGCTGTTTTAGCTCCAGGGGACAATCCTAACAAGATTATCCTCTCTACAGTGCACTCGGAAGAAAATGTGTCACTGCCAGCTGTAGACGGG :  524
             CCTGGTTCAATAATGGCTG TTTAGCTCCAGGGGACAATCCTAACAAGATTATCCTCTCTACAGTGCACTCGGAAGAAAATGTGTCACTGCCAGCTGTAGACGGG

                 *       540         *       560         *       580         *       600         *       620         *
S.lycopers : GCTGCATTTGAAGATATAAGCTTCCCATTTGAGAATGTGAAACGCCGATGAATGAAGGTACCTTGTTTGAACTCATAGCTCTTCCCATCATCTATATAAAGCTCA :  629
S.pennelli : GCTGCATTTGAAGATATAAGCTTCCCATTTGAGAATGTGAAACGCCGATGAATGAAGGTACCTTGTTTGAACTCATAGCTCTTCCCATCATCTATATAAAGCTCA :  629
             GCTGCATTTGAAGATATAAGCTTCCCATTTGAGAATGTGAAACGCCGATGAATGAAGGTACCTTGTTTGAACTCATAGCTCTTCCCATCATCTATATAAAGCTCA

                   640         *       660         *       680         *       700         *       720         *
S.lycopers : CCTTCAGCTGCCTTGGAACTATTAAGAGCTATAACCTGCAGTATAAAACAAGCTGGTAGTTAAAGATGCTCCAAACACTATCACAAAAAATAGCAAAAGTGTAAG :  734
S.pennelli : CCTTCAGCTGCCTTGGAACTATTAAGAGCTATAACCTGCAGTATAAAACAAGCTGGTTGTTAAAGATGCTCCAAACACTATCACAAAAAGTAGCAAAAGTGTAAG :  734
             CCTTCAGCTGCCTTGGAACTATTAAGAGCTATAACCTGCAGTATAAAACAAGCTGGT GTTAAAGATGCTCCAAACACTATCACAAAAA TAGCAAAAGTGTAAG

               740         *       760         *       780         *       800         *       820         *       840
S.lycopers : ACAGAACCATGAATTGATGATGTGGCATGACCATAAAAAAATGTTATTGGCAGGTCATTCCTAGTCAGTAGTCGAGAAAACAAGGTGCCGTTTAAGGAAGAATTTC :  839
S.pennelli : ACGGAACCATGAATTGATGATGTGGCATGACCATAAAAAAATGTTATTGGCAGGTCATTCCTAGTCAGTAGTCGAGAAAACAAGGTGCCGTTTAAGGAAGAATTTC :  839
             AC GAACCATGAATTGATGATGTGGCATGACCATAAAAAAATGTTATTGGCAGGTCATTCCTAGTCAGTAGTCGAGAAAACAAGGTGCCGTTTAAGGAAGAATTTC

                      *       860         *       880         *       900         *       920         *       940
S.lycopers : AGATTGAGAAGGAAAATGCATTGAATTGGATATGAAGGAAGAGAAGACATTGACGAGTTTTTCTTTATTGTCTAAGAATATTGTTTATGAGCTCAAATGTCAGAA :  944
S.pennelli : AGATTGAGAAGGAAAATGCATTGAATTGGATATGAAGGAAGAGAAGACATTGACGAGTTTTTCTTTATTGTCTAAGAATATTGTTTATGAGCTCAAATGTCAGAA :  944
             AGATTGAGAAGGAAAATGCATTGAATTGGATATGAAGGAAGAGAAGACATTGACGAGTTTTTCTTTATTGTCTAAGAATATTGTTTATGAGCTCAAATGTCAGAA

                 *       960         *       980         *      1000         *      1020         *      1040         *
S.lycopers : CTTTGGATTCACAAAGAAAGATATTAGTGAAATTAGCTATGTGCCCGAATGCCAGAACAAAGGAAAACACATCAGTTAAAGAACAGATATTCGAAAAAGCTACAA : 1049
S.pennelli : CTTTGGATTCACAAAGAAAGATATTAGTGAAATTAGCTATGTGCCCGAATGCCAGAACAAAGGAAAACATATCAGTTAAAGAACAGATATTCGAAAAAGCTACAA : 1049
             CTTTGGATTCACAAAGAAAGATATTAGTGAAATTAGCTATGTGCCCGAATGCCAGAACAAAGGAAAACA ATCAGTTAAAGAACAGATATTCGAAAAAGCTACAA

                  1060         *      1080         *      1100         *      1120         *      1140         *
S.lycopers : GATATAGATGTTACCAGAGTATAAGGATCGTTTTCCATCTGTGTTGAGCTACGACGTAAACGATCTTTCCTAGGTATGATAGTTCCAGCCCTTTGAAAAGAAGGA : 1154
S.pennelli : GATATAGATGTTACCAGAGTATAAGGATCATTTTCCATCTGTGTTGAGCTACGACGTAAACGATCTTTCCTTGGTATGATAGTTCCAGCCCTTTGAAAAGAAGGA : 1154
             GATATAGATGTTACCAGAGTATAAGGATC TTTTCCATCTGTGTTGAGCTACGACGTAAACGATCTTTCCT GGTATGATAGTTCCAGCCCTTTGAAAAGAAGGA

              1160         *      1180         *      1200         *      1220         *      1240         *      1260
S.lycopers : ATACTATCTTGCGAGACCTCATATTTGTGTGTATGACCTGCTTTGTATACAGATGCACTTCTTAAATCATACCAGGATTCCTCTCCTGGTAGATAGACAGAAACA : 1259
S.pennelli : ATACTATCTTGCGAGACCTCATATTTGTGTGTATGACCTGCTTTGTATACAGATGCACTTCTTAAATCATACCAGGATTCCTCTCCTGGTAGATAGACAGAAACA : 1259
             ATACTATCTTGCGAGACCTCATATTTGTGTGTATGACCTGCTTTGTATACAGATGCACTTCTTAAATCATACCAGGATTCCTCTCCTGGTAGATAGACAGAAACA

                      *      1280         *      1300         *      1320         *      1340         *      1360
S.lycopers : TATTTTGCTTTCTGCATGGAAAGATATGAACTTTCAGGTAATAAAGAAAAGAAAAGAGGCTGATTCAAGAAGGAACATTGCTGAGAATGAAAATGAATTGCTCCC : 1364
S.pennelli : TGTTTTGCTTTCTGCATGGAAAGATATGAACTTTCAGGTAATAAAGAAAAGAAAAGAGGCTGATTCAAGAAGGAACATTGCTGAGAATGAAAATGAATTGCTCCC : 1364
             T TTTTGCTTTCTGCATGGAAAGATATGAACTTTCAGGTAATAAAGAAAAGAAAAGAGGCTGATTCAAGAAGGAACATTGCTGAGAATGAAAATGAATTGCTCCC

                 *      1380         *      1400         *      1420         *      1440         *      1460         *
S.lycopers : TGCATTTATTTTATGTGAATGTTCCACTGGAACATAAGAATTTAAGATGTTAATTTGACTTTAATAATATATTGGTGATAATGAAAGAGTATTAAAGTTGTGGTT : 1469
S.pennelli : TGCATTTATTTTATGTGAATGTTCCACTGGAACATAAGAATTTAAGATGTTAATTTGACTTTAATAATATATTGGTGATAATGAAAGAGTATTAAAGTTGTGGTT : 1469
             TGCATTTATTTTATGTGAATGTTCCACTGGAACATAAGAATTTAAGATGTTAATTTGACTTTAATAATATATTGGTGATAATGAAAGAGTATTAAAGTTGTGGTT

                  1480         *      1500         *      1520         *      1540         *      1560         *
S.lycopers : GAAAAAAGAGCAGACATTACATCAAAGGTTAAAGAGTGACCAGTTTCAGTAATTTAAATTCTTGAAAGTTCTATCTAACATTTTGGGATGTCCCATAATAGAAAG : 1574
S.pennelli : GAAAAAAGAGCAGACAT-ACATCAAAAGTTAAAGAGTGACCAGTTTCAGTAATTTAAATTCTTGAAAGTTCTATCTAACATTTTGGGATGTCCCATAATAGAAAG : 1573
             GAAAAAAGAGCAGACAT ACATCAAA GTTAAAGAGTGACCAGTTTCAGTAATTTAAATTCTTGAAAGTTCTATCTAACATTTTGGGATGTCCCATAATAGAAAG

              1580         *      1600         *      1620         *      1640         *      1660         *      1680
S.lycopers : AGTGACATAAATTTGAACAGAAGGAATAGTTGTTTGGTTAGAACAGAACCTCTGTATAAACTCCTTGCACCAGAAGACCATTCCCAACCATGAAAGCCTCATCAT : 1679
S.pennelli : AGTGACATAAATTTGAACGGAAGGAATAGTTGTTTGGTTAGAACAGAACCTCTGTATAAACTCCTTGCACCAGAAGACCATTCCCAACCATGAAAGCCTCATCAT : 1678
             AGTGACATAAATTTGAAC GAAGGAATAGTTGTTTGGTTAGAACAGAACCTCTGTATAAACTCCTTGCACCAGAAGACCATTCCCAACCATGAAAGCCTCATCAT

                      *      1700         *      1720         *      1740         *      1760         *      1780
S.lycopers : TGCTAAAGGATTTTTCGTCTCCAGGGAACTCCATCCAAAGCGGACGAGCAACTGGAGTACCACTTGAATTTGCTTCTCTAAATAGAGTGTAGAAATAAGGAAGAT : 1784
S.pennelli : TGCTAAAGGATTTTTCATCTCCAGGGAACTCCATCCAAAGCGGACGAGCAACTGGAGTACCACTTGAATTTGCTTCTCTAAATAGAGTGTAGAAATAAGGAAGAT : 1783
             TGCTAAAGGATTTTTC TCTCCAGGGAACTCCATCCAAAGCGGACGAGCAACTGGAGTACCACTTGAATTTGCTTCTCTAAATAGAGTGTAGAAATAAGGAAGAT

                 *      1800         *      1820         *      1840         *      1860         *      1880         *
S.lycopers : ACATGTAACGAACATGTATCGCTTCCCTCATCAATTGTGTATTTCTTTCTCTGTAAAGGACATAATAACCTTGATAAACAATAACATCTTACATCACATGTAGG- : 1888
S.pennelli : ACATGTAACGAACATGTATCGCTTCCCTCATCAATTGTGTATTTCTTTCTCTGTAAAGGACATAATAACCTTGATAAACAATAACATCTTACATCACATGTAGGG : 1888
             ACATGTAACGAACATGTATCGCTTCCCTCATCAATTGTGTATTTCTTTCTCTGTAAAGGACATAATAACCTTGATAAACAATAACATCTTACATCACATGTAGG

                  1900         *      1920         *      1940         *      1960         *      1980         *
S.lycopers : TCCTAAAGCCCAAAAAGATTGTATCGTTAAATAACACTCTTATTTGGTAGCTAAATATAAGAGCAATTGGTAGGAATTTTATCCATAAAGTGCTATATGGAACCA : 1993
S.pennelli : TCCTAAAGCCCAAAAAGATTGTATCGTTAAATAACACTCTTATTTGGTAGCTACATATAAGAGCCATTGGTAGGAATTTCATCCATATAGTGCTACATGGAACCA : 1993
             TCCTAAAGCCCAAAAAGATTGTATCGTTAAATAACACTCTTATTTGGTAGCTA ATATAAGAGC ATTGGTAGGAATTT ATCCATA AGTGCTA ATGGAACCA

              2000         *      2020         *      2040         *      2060         *      2080         *      2100
S.lycopers : GAATTAAACCAGAAGCAAGAACACAAATTCTTCACAAGAAAGAAACGCATTTCTACTTAGAAAGCCGAGTTTTGCAAATTTCAAAAAGAGCTTCACGAGAGTTAT : 2098
S.pennelli : GAATTAAACCAGAAGCAAGCACACAAATTCTTCACAAGAAAGAAACGCATTTCTACTTAGAAAGCCAAGTTTTGCAAATTTCAAAAAGAGCTTCACGTGAGTTAT : 2098
             GAATTAAACCAGAAGCAAG ACACAAATTCTTCACAAGAAAGAAACGCATTTCTACTTAGAAAGCC AGTTTTGCAAATTTCAAAAAGAGCTTCACG GAGTTAT
```

```
                      *      2120         *      2140         *      2160         *      2180         *      2200
S.lycopers : ACTGCCTCCCTTAAGTCTCGTTCTTTGGTCTCTTTGGGTAGTATAATTTCCATTTAGAACAAGCCTAGGGGGGGGTTGAAGTGTGCAGTTGAAAAATGGGACAAGT : 2203
S.pennelli : ACTGCCTCCCTTAAATCTCGTTGTTTGGTCTCTTTGGGTAGTATAATTTCCATTTAGAACAAGCCTAGGGGGGGGTTGAAGTGTGCAGTTGAAAAATGGGACAAGT : 2203
             ACTGCCTCCCTTAA TCTCGTT TTTGGTCTCTTTGGGTAGTATAATTTCCATTTAGAACAAGCCTAGGGGGGGGTTGAAGTGTGCAGTTGAAAAATGGGACAAGT

                 *      2220         *      2240         *      2260         *      2280         *      2300         *
S.lycopers : TGGAGGGAATTTTATGTATCAAAACCAAATGAGAAATGTAATGCTGACTTGTGGTGTATAACAATTACAGAAGCATTGGAGAAATAA--GACAAATATAAGAATT : 2306
S.pennelli : TGGAGGGAATTTTATGTATCAAAACCAAATGAGAAATGTAATGCTGACTTGTGGTGTATAACAATTACAGAAGCATTGGAGAAATAAAAGACAAATATAAGAATT : 2308
             TGGAGGGAATTTTATGTATCAAAACCAAATGAGAAATGTAATGCTGACTTGTGGTGTATAACAATTACAGAAGCATTGGAGAAATAA  GACAAATATAAGAATT

                   2320         *      2340         *      2360         *      2380         *      2400         *
S.lycopers : TGGAGAAAATTGACAAAGAAAACATATATGTATTGTATTATAAACTTTTTTCATAACCACGACATCCCAGTCAGGTTGCGTACACAACCTCGACTAATTCCACGG : 2411
S.pennelli : TGGAGAAAATTGACAAAGAAAACATATATGTATTGTATTATAAACTTTTTTCATAACCACGACATCCCAGTCAGGTTGCGTACACAACCTCGACTAATTCCACGG : 2413
             TGGAGAAAATTGACAAAGAAAACATATATGTATTGTATTATAAACTTTTTTCATAACCACGACATCCCAGTCAGGTTGCGTACACAACCTCGACTAATTCCACGG

              2420         *      2440         *      2460         *      2480         *      2500         *      2520
S.lycopers : GATACCTGCTACCTCCTGCCAACACAGGTATCACAGAACCTTGTCCACCGGGGCTTGGGTAGAGGAAAGAAATCACCTAGTATTTTTACCTCCATTCGGATGTGA : 2516
S.pennelli : GATACCTGCTACCTCCTGCCAACACACGTATCACAGAACCTTGTCCACCGGGGCTTGGGTAGAGGAAAGAAATCACCTAGTATTTTTACCTCCATTTGGATGTGA : 2518
             GATACCTGCTACCTCCTGCCAACACA GTATCACAGAACCTTGTCCACCGGGGCTTGGGTAGAGGAAAGAAATCACCTAGTATTTTTACCTCCATT GGATGTGA

                      *      2540         *      2560         *      2580         *      2600         *      2620
S.lycopers : TTCAGAAACAACATGGTTCTCAACCCATTTCATTGATCACTAGGCCACATTCTATGGTGGTCTAATGTCTTGTACTCTTATATTATAAGCTTTTAACATTAAATT : 2621
S.pennelli : TTCAGAAACAACATGGTTCTCAACCCATTTCATTGATCACTAGGCCACATTCTATGGTGGTCTAATGTCTTGTACTCTTATATTATAAGCTTTTAACATTAAATT : 2623
             TTCAGAAACAACATGGTTCTCAACCCATTTCATTGATCACTAGGCCACATTCTATGGTGGTCTAATGTCTTGTACTCTTATATTATAAGCTTTTAACATTAAATT

                 *      2640         *      2660         *      2680         *      2700         *      2720         *
S.lycopers : GAAGAATCATACCCAAATAACCAAGGTTCCCGTCTTTTAGTGTCATGATGTGCATGGCCCCGGAAAAAGGGATAATATGCACCTAATTGATACCAGCGAACCAAC : 2726
S.pennelli : GAAGAATCATACCCAAATAACCAAGGTTCCCGTCTTTTAGTGTCATGATGTGCATGGCCCCGGAAAAAGGGATAATATGCACCTAATTGATACCAGCGAACCAAC : 2728
             GAAGAATCATACCCAAATAACCAAGGTTCCCGTCTTTTAGTGTCATGATGTGCATGGCCCCGGAAAAAGGGATAATATGCACCTAATTGATACCAGCGAACCAAC

                   2740         *      2760         *      2780         *      2800         *      2820         *
S.lycopers : AATTCAGCGTCAGGATTACCAAAAAATCCACCAACATCTGCACCTGTTAAAGAAAGATGTTGCACAACAGTCATGAAATCAGAAAGATATGGCTGCCTACTAATT : 2831
S.pennelli : AATTCAGTGTCAGGATTACCAAAAAATCCACCAACATCTGCACCTGTTAAAGAAAGATGTTGCACAACCGTCATGAAATCAGAAAGATATGGCTGCCTACTAATT : 2833
             AATTCAG GTCAGGATTACCAAAAAATCCACCAACATCTGCACCTGTTAAAGAAAGATGTTGCACAAC GTCATGAAATCAGAAAGATATGGCTGCCTACTAATT

              2840         *      2860         *      2880         *      2900         *      2920         *      2940
S.lycopers : GACCGAGAAAAGAGAATCATATTAACTAACCAGAAAAGACTATTCCAGAGATGCTAAGAGTTAACACCATGGGGACTGAAACCCTTAAGTGCTCCCATTCTGCTG : 2936
S.pennelli : GACCGAGAAAAGAGAATCATATTAACTAACCAGAAAATACTATTCCAGAGATGCTAAGAGTTAACACCATGGGGACTGAAACCCTCAAGTGCTCCCATTCTGCTG : 2938
             GACCGAGAAAAGAGAATCATATTAACTAACCAGAAAA ACTATTCCAGAGATGCTAAGAGTTAACACCATGGGGACTGAAACCCT AAGTGCTCCCATTCTGCTG

                      *      2960         *      2980         *      3000         *      3020         *      3040
S.lycopers : TATTATCTCCAGTCCAAATTGCTCCATATCTTTGACTTCCGGCAAAGAAAGCCCTTGCCAAAACAAAAGGCCTATCTTTTCCATCTCCACGCTTTAGAAGCCCGT : 3041
S.pennelli : TATTATCTCCAGTCCAAATTGCTCCATATCTTTGACTTCCGGCAAAGAAAGCCCTTGCCAAAACAAAAGGCCTATCTTTTCCATCTCCACGCTTTAGAAGCCCGT : 3043
             TATTATCTCCAGTCCAAATTGCTCCATATCTTTGACTTCCGGCAAAGAAAGCCCTTGCCAAAACAAAAGGCCTATCTTTTCCATCTCCACGCTTTAGAAGCCCGT

                 *      3060         *      3080         *      3100         *      3120         *      3140         *
S.lycopers : CGGATGTTGCCATATGGAAATAGTAACCATATGAATTGTGCAACTCCCTGTGCTCTACTCCTCCATGATGTAAAGCATCTCTTGGCATTGTTACCTAGCATTGAG : 3146
S.pennelli : CGGATGTTGCCATATGGAAATAGTAACCATATGAATTGTGCAACTCCCTGTGCTCTACTCCTCCATGATGTAAAGCATCTCTTGGCATTGTTACCTAGCATTGAG : 3148
             CGGATGTTGCCATATGGAAATAGTAACCATATGAATTGTGCAACTCCCTGTGCTCTACTCCTCCATGATGTAAAGCATCTCTTGGCATTGTTACCTAGCATTGAG

                   3160         *      3180         *      3200         *      3220         *      3240         *
S.lycopers : AAAGAGTGAAAATTTCAATGACATCAGTAAATCTACCTGGTTTTCCTAATCACAATCGAGTGAAATAGCAAACTTGCTAAGTATTTGAGAAACTCAGGCCCAGAA : 3251
S.pennelli : AAAGAGTGAAAATTTCAATGACATCAGTAAATCTACCTGGTTTTCCTAATCACAATCGAGTGAAATAGCAAACTTGCTAAGTATTTGAGAAACTCAGGCCCAAAA : 3253
             AAAGAGTGAAAATTTCAATGACATCAGTAAATCTACCTGGTTTTCCTAATCACAATCGAGTGAAATAGCAAACTTGCTAAGTATTTGAGAAACTCAGGCCCA AA

              3260         *      3280         *      3300         *      3320         *      3340         *      3360
S.lycopers : ACTAATTGCAGTTCTTTATCAAAGAAATATTGGGCAACGCCAAACCACTTAATCAAGAACTACCATGAATGACAGTTATCAGACTGAAGCACTTCTAGCCCTGGG : 3356
S.pennelli : ACTAATTGCAGTTCTTTATCAAAGAAATATTGGGCAACGCCAAACCACTTAATCAAGAACTACCATGAATGACAGTTATCAGACTGAAGCACTTCTAGCCCTGGG : 3358
             ACTAATTGCAGTTCTTTATCAAAGAAATATTGGGCAACGCCAAACCACTTAATCAAGAACTACCATGAATGACAGTTATCAGACTGAAGCACTTCTAGCCCTGGG

                      *      3380         *      3400         *      3420         *      3440         *      3460
S.lycopers : GCTAATTATTCTTTTCAGATAAAATATCTCCAATTTCTATCTCATACATTCAAGGTTACGCTCACTGAAAAATAACAGAACTTGTTTAGTAGCATTTATATAACC : 3461
S.pennelli : GCTAATTATTCTTTTCAGATAAAATATCTCCAATTTCTATCTCATACATTCAAGGTTACGCTCAGTGAAAAATAACAGAACTTGTTTAGTAGCATTTATATAACC : 3463
             GCTAATTATTCTTTTCAGATAAAATATCTCCAATTTCTATCTCATACATTCAAGGTTACGCTCA TGAAAAATAACAGAACTTGTTTAGTAGCATTTATATAACC

                 *      3480         *      3500         *      3520         *      3540         *      3560         *
S.lycopers : CAGGCACTTAAATAGCGAAAGCTGAACAATCAGTGGCAACACTACAATCAGAGACGGATCCAACTTCCAAGATTTGAAGGTTGAGGGTGCCACTGTTTTACTACT : 3566
S.pennelli : CAGGCATTTACATAGCGAAAGCTGAACAATCAGTGGCAACACTGCAATCAGAGACGGATC-------CAAGATTTGAAGGTTGAGGGTGCCACTGTTTTATTACA : 3561
             CAGGCA TTA ATAGCGAAAGCTGAACAATCAGTGGCAACACT CAATCAGAGACGGATC       CAAGATTTGAAGGTTGAGGGTGCCACTGTTTTA TAC

                   3580         *      3600         *      3620         *      3640         *      3660         *
S.lycopers : AACTCATCGCTAGTAATGAAAATCGAATTAGGGTGTACAGTTAGTCGCTTCAATCCGTTTTTGGCCAATTGAGTTCGATTTACAAGTTTTTTGTATCTAAGTAAA : 3671
S.pennelli : AACTCATTGCTAGTAATGAAAATCGAATTAGGGTGTACAGTTAGTCGCTTCAATCTGTTTTTGGCCAATTGAGTTCGATTTACAAGTTTTTTGTATCTAAGTAAA : 3666
             AACTCAT GCTAGTAATGAAAATCGAATTAGGGTGTACAGTTAGTCGCTTCAATC GTTTTTGGCCAATTGAGTTCGATTTACAAGTTTTTTGTATCTAAGTAAA

              3680         *      3700         *      3720         *      3740         *      3760         *      3780
S.lycopers : CACACGATCATCGAACCAAAATGAATTTTATACAATTTTAACTAGTTCTTGATCAAATTCTATTGAAAAAAAATGAGAAAAACATAAACTAGAAAACTAAATTTT : 3776
S.pennelli : TACACGATCATCGAACCAAAATGAATTTTATACGATTTTAACTAGTTCTTGATCAAATTCTATTGAAAAAAA-TGAGAAAAACATAAACTAGAAAACTAAATTTT : 3770
              ACACGATCATCGAACCAAAATGAATTTTATAC ATTTTAACTAGTTCTTGATCAAATTCTATTGAAAAAAA TGAGAAAAACATAAACTAGAAAACTAAATTTT

                      *      3800         *      3820         *      3840         *      3860         *      3880
S.lycopers : TGAACACACAATTAAACTCCTTCAAAACAACTTCTACAAGGATCCCATCAACTTACAAGGAAAGAGAAATATTTTCATTACAAAAATTCAAAAAATTATCTATAC : 3881
S.pennelli : TGAATACACAATTAAACTCCTTCAAAACAACTTCTACAAGGATCCCATCAACTTACAAGGAAAGAGAAATATTTTCATTACAAAAATTCAAAAAATTATCTATAC : 3875
             TGAA ACACAATTAAACTCCTTCAAAACAACTTCTACAAGGATCCCATCAACTTACAAGGAAAGAGAAATATTTTCATTACAAAAATTCAAAAAATTATCTATAC

                 *      3900         *      3920         *      3940         *      3960         *      3980         *
S.lycopers : AATAATATTTTTTAAGAAAAATTGAACAAAACAAAACACTTGATAAAGTGCAAAAAGAAGTCTATGAACTAGCACAAAATAGCCCTTACCACTGTTGCCTTTGTT : 3986
S.pennelli : AATAATATTTTTTAAGAAAAATTGAACAAAACAAAACACTTGATAAAGTGCAAAAAGAAGTCTATGAACTAGCACAAAATGGCCCTTACCACTGTTGCCTTTGTT : 3980
             AATAATATTTTTTAAGAAAAATTGAACAAAACAAAACACTTGATAAAGTGCAAAAAGAAGTCTATGAACTAGCACAAAAT GCCCTTACCACTGTTGCCTTTGTT

                   4000         *      4020         *      4040         *      4060         *      4080         *
S.lycopers : CAAAGGCATAATACATAAATGTGCTACTTGGTCCCAGCTAACATCTATGCCCTCCAACTTTGGGTGTACACAAGTAGGCACTTACACTTGTATATAGTTAAAGAA : 4091
S.pennelli : CAAAGGCATAATACATAAATGTGCTACTTGGTCCCAGCTAACATCTATGCCCTCCAGCTTTGGGTGTACACAAGTAGGCACTTACACTTGTATATAGTTAAAGAA : 4085
             CAAAGGCATAATACATAAATGTGCTACTTGGTCCCAGCTAACATCTATGCCCTCCA CTTTGGGTGTACACAAGTAGGCACTTACACTTGTATATAGTTAAAGAA

              4100         *      4120         *      4140         *      4160         *      4180         *      4200
S.lycopers : GTAGACACATATGTGACATTATACATTTATTTAGGAAACAAATTTAATAGATAAAATGTACCATAGATAAAGGGGGGAGGGGGGAGGGGGGAGATTAAAATCAG : 4196
S.pennelli : GTAGACACATATGTGACATTATACATTTATTTAGGAAACAAATTTAATAGATAAAATGTACCATAGATAAAGGGGGGAGG-------GGGGAGATTAAAATCAG : 4183
             GTAGACACATATGTGACATTATACATTTATTTAGGAAACAAATTTAATAGATAAAATGTACCATAGATAAAGGGGGGAGG       GGGGAGATTAAAATCAG

                      *      4220         *      4240         *      4260         *      4280         *      4300
S.lycopers : ATTTAATTTAATATTATAGATCTTGAAAAATTAAAAAGAAGCAAAGTGATAATTTAAATAAATAAATTAACATATGGGTTCCATTGGAGGAAGAGTAGGAATAGT : 4301
S.pennelli : ATTTAATTTAATATTATAGATCTTGAAAGATTAAAAAGAAGCAAAGTGATAATTTAAATAAATAAATTAACATATGGGTTCCATTGGAGGAAGAGTAGGAATAGT : 4288
             ATTTAATTTAATATTATAGATCTTGAAA ATTAAAAAGAAGCAAAGTGATAATTTAAATAAATAAATTAACATATGGGTTCCATTGGAGGAAGAGTAGGAATAGT
```

```
                *      4320         *      4340         *      4360         *      4380         *      4400         *
S.lycopers : AAATGAAAAATATAAATAAGTAAAGAAAACACAAAAAGGGAAACAAGAAAAGAAAACGGGCAGTAGTTTGCCAGACATACACCGCAGGGATTCAAACCCTCGCTC : 4406
S.pennelli : AAATGAAAAATATAAATAAGTAAAGAAAACACAAAAAGGGAAACAAGAAAAGAAAACGGGCAGTAGTTTGCCAGATATACACCGCAGGGATTCAAACCCTCGCTC : 4393
             AAATGAAAAATATAAATAAGTAAAGAAAACACAAAAAGGGAAACAAGAAAAGAAAACGGGCAGTAGTTTGCCAGA ATACACCGCAGGGATTCAAACCCTCGCTC

                 4420         *      4440         *      4460         *      4480         *      4500         *
S.lycopers : ATGCGTGACATTCGCACACTAACAAACGGACCAGAAACAAATTTGTTACTGAGGGTGCCAAACGTTATTTATATAAATTACACACTATAATATTTATGTATATAT : 4511
S.pennelli : ATGCGTGACATTCGCACACTAACAAACGGACCAGAAACAAATTTGTTACTGAGGGTGCCAAACGTTATTTATATAAATTGCACACTATAATATTTATGTATATAT : 4498
             ATGCGTGACATTCGCACACTAACAAACGGACCAGAAACAAATTTGTTACTGAGGGTGCCAAACGTTATTTATATAAATT CACACTATAATATTTATGTATATAT

              4520         *      4540         *      4560         *      4580         *      4600         *      4620
S.lycopers : GAAAATTTTAGCAAAGCTACAGGTGTCGTGGCACCCTCACTGTCTCACATAGATCCGCCCCCGTTACAATTAGCTCATGGTCTTTAGTAGCTTGAAGATCAAAAG : 4616
S.pennelli : GAAAATTTTAGCAAAGCTACAGGTGTCATGGCACCCT-ACTGTCTCACATAGATCCGCCCCCGTTACAATTAGCTCATGGTCTTTAATAGCTTGAAGATCAAAAG : 4602
             GAAAATTTTAGCAAAGCTACAGGTGTC TGGCACCCT ACTGTCTCACATAGATCCGCCCCCGTTACAATTAGCTCATGGTCTTTA TAGCTTGAAGATCAAAAG

                     *      4640         *      4660         *      4680         *      4700         *      4720
S.lycopers : GGAAATTGACAAAAAAATAATTGGAAAAAACAAAATGAATACCTTAGAATCATCATTGACAACATCCTATTCTGATTAGCTTTTGTTCCTTTTACTTGGTACCTTA : 4721
S.pennelli : GGAAATTGACAAAAAAATAATTGGAAGAAACAAAATGAATACCTTAGAATCATCATTGACAACATCCTATTCTGATTAGCTTTTGTTCCTTTTACTTGCTACCTTA : 4707
             GGAAATTGACAAAAAAATAATTGGAA AAACAAAATGAATACCTTAGAATCATCATTGACAACATCCTATTCTGATTAGCTTTTGTTCCTTTTACTTG TACCTTA

                *      4740         *      4760         *      4780         *      4800         *      4820         *
S.lycopers : ATTGGTTTCAAAAACTTCCGATAGATCATTCCTAGTTTATATGGTACTAT-TTGTATCTTCTCTGTTTTCTATGTTTTGCTTACATACATTGACAACAAGGATTC : 4825
S.pennelli : ATTGGTTTCAAAAACTTCCGATAGATCATTCCTAGTTTATATGGTACTATATTGTATCTTCTCTGTTTTCTATGTTTTGCTTACATACATTGACAACAAGGATTC : 4812
             ATTGGTTTCAAAAACTTCCGATAGATCATTCCTAGTTTATATGGTACTAT TTGTATCTTCTCTGTTTTCTATGTTTTGCTTACATACATTGACAACAAGGATTC

                 4840         *      4860         *      4880         *      4900         *      4920         *
S.lycopers : AGTTTCAAGTTTAGCACCTAAAAGTAATGAGGTCTAAATCTACAACAAAACTGTTAAAAGTTCAACTCCTTATTATTCATTTTTGTCAAAGGATCTTATTTTCTG : 4930
S.pennelli : AGTTTCAAGTTTAGCACCTAAAAGTTATGAGGTCTAAATCTACAACAAAACTGTTAAAAGTTCAACTCCTTATTATTCATTTTTGTCAAAGGATCTTATTTTCTG : 4917
             AGTTTCAAGTTTAGCACCTAAAAGT ATGAGGTCTAAATCTACAACAAAACTGTTAAAAGTTCAACTCCTTATTATTCATTTTTGTCAAAGGATCTTATTTTCTG

              4940         *      4960         *      4980         *      5000         *      5020         *      5040
S.lycopers : ATGCTTTCCATAGGTGCTATCCTTGGAGAAATTAAGATATTTCGTATTTCTCTTGCTAAACCTGCTTCAAATTAGTCTGGAAACAGGACAACTGTAGGGAAATAA : 5035
S.pennelli : ATGCTTTCCACAGGTGCTATCCTTGGAGAAATTAAGATATTTCGTATTTCTCTTGCTAAACCTGCTTCAAATTAGTCTGGAAACAGGACAACTGTAGGGAAATAA : 5022
             ATGCTTTCCA AGGTGCTATCCTTGGAGAAATTAAGATATTTCGTATTTCTCTTGCTAAACCTGCTTCAAATTAGTCTGGAAACAGGACAACTGTAGGGAAATAA

                     *      5060         *      5080         *      5100         *      5120         *      5140
S.lycopers : TTAAATAAAACGATGGGGGCCTTGAGGGCTCAACGAAAAGAGATATTTAAAAAATGAAGATGTAAATGGAGTCGCACAACTTTTGCAACAGTAGAAATATTAAAA : 5140
S.pennelli : TTAAATAAAACGATGGGGGCCTTGAGGGCTCAACGAAAAGAGATATTTAAAAAATGAAGATGTAAATGGAGTCGCACAACTTTTGCAACAGTAGAAATATTAAAA : 5127
             TTAAATAAAACGATGGGGGCCTTGAGGGCTCAACGAAAAGAGATATTTAAAAAATGAAGATGTAAATGGAGTCGCACAACTTTTGCAACAGTAGAAATATTAAAA

                *      5160         *      5180         *      5200         *      5220         *      5240         *
S.lycopers : ACCAATGTAGAAGGAATAATCTCAAATTAAGATTTGGACCAGCAATTCAAGTGACTCATCTGAACCATCTACGCACAGGACTAAGAAACCATGGAGTACCCTATG : 5245
S.pennelli : ACCAATGTAGAAGGAATAATCTCAAATTAAGATTTGGACCAGCAATTCAAGTGACTCATCTGAACCATCTACGCACAGGACTAAGAAACCATGGAGTACCCTATG : 5232
             ACCAATGTAGAAGGAATAATCTCAAATTAAGATTTGGACCAGCAATTCAAGTGACTCATCTGAACCATCTACGCACAGGACTAAGAAACCATGGAGTACCCTATG

                 5260         *      5280         *      5300         *      5320         *      5340         *
S.lycopers : AGTTCAAATTATTTTGATTGGCTTTGCCAAACTGAAGAAGAAATAGATAGCTGATGATTTGGTAAGGAACGGGTTGATTCGCTTTGTCAAACAAAATGCTCATTG : 5350
S.pennelli : AGTTCAAATTATTTTGATTGGCATTGCCAAACTGAAGAAGAAATAGATAGCTGATGATTTGGTAAGGAACGGGTTGATTCGCTTTGCCAAACAAAATGCTCATTG : 5337
             AGTTCAAATTATTTTGATTGGC TTGCCAAACTGAAGAAGAAATAGATAGCTGATGATTTGGTAAGGAACGGGTTGATTCGCTTTG CAAACAAAATGCTCATTG

              5360         *      5380         *      5400         *      5420         *      5440         *      5460
S.lycopers : ATTGTCTAGTGTTAAATTTGAGATATGAGGAAACAATGTTGCCCAACTATTACAAGCATAGTACTCAGGATCAAACATGAAACATCTTGCTTCAGGAAGCATGTT : 5455
S.pennelli : ATTGTCTAGTGTTAAATTTGAGATATGAGGAAACAATGTTGCCCAACTATTACAAGCATAGTACTCAGGATCAAACATGAAACATCTTGCTTCAGGAAGCATGTT : 5442
             ATTGTCTAGTGTTAAATTTGAGATATGAGGAAACAATGTTGCCCAACTATTACAAGCATAGTACTCAGGATCAAACATGAAACATCTTGCTTCAGGAAGCATGTT

                     *      5480         *      5500         *      5520         *      5540         *      5560
S.lycopers : CATTTATTAAATAAATTTCTGCCTAGTTAGTCTTGGTGTACATTTGAGCATGCAGGAGTTGATTATCATTTCCACTTTTGTACTCTCAACCGCTGCAACATATGT : 5560
S.pennelli : CATTTATTAAATAAATCTCTGCCTAGTTAGTCTTGGTGTACATTAGAGCATGCAGGAGTTGATTATCATTTCCACTTTTGTACTCTCAACCACTGCAACATATGT : 5547
             CATTTATTAAATAAAT TCTGCCTAGTTAGTCTTGGTGTACATT GAGCATGCAGGAGTTGATTATCATTTCCACTTTTGTACTCTCAACC CTGCAACATATGT

                *      5580         *      5600         *      5620         *      5640         *      5660         *
S.lycopers : TGGCATCAACTCCAAAAGAAAGGGTCTACCTGTGGCCGTGTCTCTCCTAATTGACTCCTAGATAACATATGCAATCATTATGCAGACAAGTCTTGTGTATATAAA : 5665
S.pennelli : TGGCATCAACTCCAAAAGAAAGGGTCTACCTGTGGCCGTGTCTCTCCTAATTGACTCCTAGATAACATATGCAATCATTATGCAGACAAGTCTTGTGTATATAAA : 5652
             TGGCATCAACTCCAAAAGAAAGGGTCTACCTGTGGCCGTGTCTCTCCTAATTGACTCCTAGATAACATATGCAATCATTATGCAGACAAGTCTTGTGTATATAAA

                 5680         *      5700         *      5720         *      5740         *      5760         *
S.lycopers : AGTCTTGCAAATCGAAACCGCATTAAATATATAAAATTCTTGAAGATTCAGCAAATAATATACTGTAAATGGGTGCTTCGAATGAAAATACTTATCATCGCAATA : 5770
S.pennelli : ATTCTTGCAAATCGAAACCGCATTAAATATATAAAATTATTGAAGATTCAGCAAATAATATACTGTAAATGGGTGCTTCGAATGAAAATACTTATCATCGCAATA : 5757
             A TCTTGCAAATCGAAACCGCATTAAATATATAAAATT TTGAAGATTCAGCAAATAATATACTGTAAATGGGTGCTTCGAATGAAAATACTTATCATCGCAATA

              5780         *      5800         *      5820         *      5840         *      5860         *      5880
S.lycopers : GTACTCCAGTATTCGACTATTAACAAAGGAAAGAGTGTGGAT---------------ATTAACAAGTGAAAACTGACATATTGACTGCATTCTTGAAGAACTGCA : 5860
S.pennelli : GTACTCCAGTATTCGACTATTAACAAAGGAAAGAGTGTGGATGGAAAGAGTGTGGATATTAACAAGTGAAAACTGACATATTGACTGCATTCTTGAAGAACTGCA : 5862
             GTACTCCAGTATTCGACTATTAACAAAGGAAAGAGTGTGGAT               ATTAACAAGTGAAAACTGACATATTGACTGCATTCTTGAAGAACTGCA

                     *      5900         *      5920         *      5940         *      5960         *      5980
S.lycopers : AGAAATTCTACCTAATAAAATTTGAAAGACAAGTATAAGGATAATATGCAAGGGACAGAATAGCTAATTTTTTTAATGCTCTCATTCAGTTCACAGGACTACAAA : 5965
S.pennelli : AGAAATTCTACCTAATAAAATTTGAAAGACAAGTATAAGGATAATATGCAAGGGACAGAATAGCTAATTTTTTTAGTGCTCTCATTCAGTTCACAGGACTACAAA : 5967
             AGAAATTCTACCTAATAAAATTTGAAAGACAAGTATAAGGATAATATGCAAGGGACAGAATAGCTAATTTTTTTA TGCTCTCATTCAGTTCACAGGACTACAAA

                *      6000         *      6020         *      6040         *      6060         *      6080         *
S.lycopers : CTGGTGAAGATACTCTCTGGTGACAAGGGAACAGTAAGGACCCTACAGGGTCAATGCAGCATATCATCTAATGAACCAGCCCAACCTCCCAGACTCATAATTGGC : 6070
S.pennelli : CTGGTGAAGATACTCTCTGGTGACAAGGGAACAGTAAGAACCCTACAGGGTCAATGCAGCATATCATCTAATGAACCAGCCCAACCTCC-AGACTCATAATTGGC : 6071
             CTGGTGAAGATACTCTCTGGTGACAAGGGAACAGTAAG ACCCTACAGGGTCAATGCAGCATATCATCTAATGAACCAGCCCAACCTCC AGACTCATAATTGGC

                 6100         *      6120         *      6140         *      6160         *      6180         *
S.lycopers : CATGCAAGCAAAATTGGAAAGCCAAAATCCCATATGAAGTTTCTTGTTTTGTGTGGTTACTAGCTAGAGAAGTTGTCCTCACACTGGACAATCTGATGAAGAGGG : 6175
S.pennelli : CATGCAAGCAAAATTGGAAAGCCAAAATCCCATATGAAGTTTCTTGTTTTGTGTGGTTACTAGCTAGAGAAGTTGTCCTCACACTGGACAATCTGATGAAGAGGG : 6176
             CATGCAAGCAAAATTGGAAAGCCAAAATCCCATATGAAGTTTCTTGTTTTGTGTGGTTACTAGCTAGAGAAGTTGTCCTCACACTGGACAATCTGATGAAGAGGG

              6200         *      6220         *      6240         *      6260         *      6280         *      6300
S.lycopers : GAATGCACTTGTGCTCAAGATGCTTCATGTGCAATGAGAATGCAAAGCCAGTAACCACCTATTCTTACACAGCAAGCTCACAGGCATGAGTTGGAGTGTCATTTT : 6280
S.pennelli : GAATGCACTTGTGCTCAAGATGCTTCATGTGCAATGAGAATGCAAAGCCAGTAACCACCTATTCTTACACAGCAAGCTCACAGGCATGAGTTGGAGTGTCATTTT : 6281
             GAATGCACTTGTGCTCAAGATGCTTCATGTGCAATGAGAATGCAAAGCCAGTAACCACCTATTCTTACACAGCAAGCTCACAGGCATGAGTTGGAGTGTCATTTT

                     *      6320         *      6340         *      6360         *      6380         *      6400
S.lycopers : GAACCTTAAAAGCATAGCATGGACCATGCCGTGAAAGGTCTCTCAAGCTCTAAGAAGTTGGGAAGAAGCAGGCTCAGTAGCAAAGTGCAGAAATAGATGGACACT : 6385
S.pennelli : GAACCTTAAAAGCATAGCATGGACCATGCCGTGAAAGGTCTCTCAAGCTCTAAGAAGTTGGGAAGAAGCAGGCTCAGTAGCAAAGTGCAGAAATAGATGGACACT : 6386
             GAACCTTAAAAGCATAGCATGGACCATGCCGTGAAAGGTCTCTCAAGCTCTAAGAAGTTGGGAAGAAGCAGGCTCAGTAGCAAAGTGCAGAAATAGATGGACACT

                *      6420         *      6440         *      6460         *      6480         *      6500         *
S.lycopers : TATCCCTATATGTATCTGGTGGACAGTATGGAAGGAGAAAATTCCAGGTGCTTTTAGCAAATAGAGAATGATGTGCCGAAGATCAAGCCAAATTTTATTTTACTG : 6490
S.pennelli : TATCCCTATATGTATCTGGTGGACAGTGTGGAAGGAGAAAATTCCAGGTGCTTTGAGCAAATAGAGAATGATGTGCCGAAGATCAAGCCAAATTTTATTTTACTG : 6491
             TATCCCTATATGTATCTGGTGGACAGT TGGAAGGAGAAAATTCCAGGTGCTTT AGCAAATAGAGAATGATGTGCCGAAGATCAAGCCAAATTTTATTTTACTG
```

```
                     6520         *       6540         *       6560         *       6580         *       6600         *
S.lycopers : TTTTGTTTTTGGTGTAATCATTTCTACTCTAATGACACTATTTCTATCACTGTTATCCTTGATCCATTATAGATAGAAATAGGAACACTATCGGGTTCCAATTGC : 6595
S.pennelli : TTTTGTTTTTGGTGTAATCATTTCTACTCTAATGACACTATTTCTATCACTGTTATCCTTGATCCATTATAGATAGAAATAGGAGCACTATCGGGTTCCAATAGC : 6596
             TTTTGTTTTTGGTGTAATCATTTCTACTCTAATGACACTATTTCTATCACTGTTATCCTTGATCCATTATAGATAGAAATAGGA CACTATCGGGTTCCAAT GC

              6620         *       6640         *       6660         *       6680         *       6700         *       6720
S.lycopers : ATATATGGTTTCAGTACTACCTAAGTACTGTTTTGTATAATATGCAACGGACAGAATAATAGTCAGAATGTTGGTCCAGCATAACTGGAGAAGTATCAAAACAGT : 6700
S.pennelli : ATATATGGTTTCAGTACTACCTAAGTACTGTTTTGGATAATATGCAAGGGACAGAATAATAGTCAGAATGTTGGTCCAGCATAACTGGAGAAGTATCAAAACAGT : 6701
             ATATATGGTTTCAGTACTACCTAAGTACTGTTTTG ATAATATGCAA GGACAGAATAATAGTCAGAATGTTGGTCCAGCATAACTGGAGAAGTATCAAAACAGT

                     *       6740         *       6760         *       6780         *       6800         *       6820
S.lycopers : GAATCAAAATAATCACAGGTAAACTATATACCTCTGGTCCATTGAAGACGGAAGGTTCATTCATGTCATTCCAGATGTATAAATACTTTGTCGAGCCAACATAGC : 6805
S.pennelli : GAATCAAAACAATCACAGGTAAACTATATACCTCTGGTCCATTGAAGACGGAAGGTTCATTCATGTCATTCCAGATGTATAAATACTTTGTCGAGCCAACATAGC : 6806
             GAATCAAAA AATCACAGGTAAACTATATACCTCTGGTCCATTGAAGACGGAAGGTTCATTCATGTCATTCCAGATGTATAAATACTTTGTCGAGCCAACATAGC

                *       6840         *       6860         *       6880         *       6900         *       6920         *
S.lycopers : TATCAAGTGAAAATTTGTCACTCCACCATGACCTAATCTCGGGATTCAGCAGGTCAGTATATGATGAGGAACCAGGCCAGCACCATCCATCATAATCCTTACCAG : 6910
S.pennelli : TATCAAGTGAAAATTTGTCACTCCACCATGACCTAATCTCGGGATTCAGCAGGTCAGTATATGATGAGGAACCAGGCCAGCACCATCCATCATAATCCTTACCAG : 6911
             TATCAAGTGAAAATTTGTCACTCCACCATGACCTAATCTCGGGATTCAGCAGGTCAGTATATGATGAGGAACCAGGCCAGCACCATCCATCATAATCCTTACCAG

                     6940         *       6960         *       6980         *       7000         *       7020         *
S.lycopers : TAGCATCCTTAACATAGTATCCCTTTGCTGAGGCCTCCTTGTGTATATGGTAAGACTCATCTCTCTTGATATGAGGATCCACAATGGTAACCATGTGTCTACCCT : 7015
S.pennelli : TAGCATCCTTAACATAGTATCCCTTTGCTGAGGCCTCCTTGTGTATATGGTAAGACTCATCCCTCTTGATATGAGGATCCACAATGGTAACCATGTGTCTACCCT : 7016
             TAGCATCCTTAACATAGTATCCCTTTGCTGAGGCCTCCTTGTGTATATGGTAAGACTCATC CTCTTGATATGAGGATCCACAATGGTAACCATGTGTCTACCCT

              7040         *       7060         *       7080         *       7100         *       7120         *       7140
S.lycopers : TTGCAGCTAACTTCTTCTGCATTTCCTCTGGGTTAGGAAACAACACTCTGTCCCAAGTAAAGTACTTCTTCCCATCTGTGTGCTCAATATCAAGCCACAAAACAT : 7120
S.pennelli : TTGCAGCTAACTTCTTCTGCATTTCCTCTGGGTTAGGAAACAACACCCTGTCCCAAGTAAAGTACTTCTTCCCATCTGTGTGCTCAATATCAAGCCACAAAACAT : 7121
             TTGCAGCTAACTTCTTCTGCATTTCCTCTGGGTTAGGAAACAACAC CTGTCCCAAGTAAAGTACTTCTTCCCATCTGTGTGCTCAATATCAAGCCACAAAACAT

                     *       7160         *       7180         *       7200         *       7220         *       7240
S.lycopers : CATAAGGGATATCATGCTCATCAAACTTTGAATCAACATTATAAACATCTTCCTCGTCTCTATAATTCCATCTACATTGATGGTATGCAGTTGCGAATAACTGTG : 7225
S.pennelli : CATAAGGGATATCATGCTCATCAAATTTTGAATCAACATTATAAACATCTTCCTCGTCTCTATAATTCCATCTACATTGATGGTATGCAGTTGCGAATAACTGTG : 7226
             CATAAGGGATATCATGCTCATCAAA TTTGAATCAACATTATAAACATCTTCCTCGTCTCTATAATTCCATCTACATTGATGGTATGCAGTTGCGAATAACTGTG

                *       7260         *       7280         *       7300         *       7320         *       7340         *
S.lycopers : GCATAGATGGCCTTCCCGTTACACTAGTATACTGTCTAACCACATCTTTTGGCCCAGGACCAATGAAAAAGAAAATATCCACCACACCAGACTCACTCATCCACA : 7330
S.pennelli : GCATAGATGGCCTTCCCGTTACACTAGTATACTGTCTAACCACATCTTTTGGCCCAGGACCAATGAAAAAGAAAGTATCCACTACACCAGACTCACTCATCCACA : 7331
             GCATAGATGGCCTTCCCGTTACACTAGTATACTGTCTAACCACATCTTTTGGCCCAGGACCAATGAAAAAGAAA TATCCAC ACACCAGACTCACTCATCCACA

                     7360         *       7380         *       7400         *       7420         *       7440         *
S.lycopers : AAGTATCAATCCTGTGCTTGTCCGAGGGAAACATTATCTTTGAAGACTCATTTGAATTCCAGCCGGATCCCAATACATCAATCTGCATTTCTGCAGCATTCAACC : 7435
S.pennelli : AAGTATCAATCCTGTGCTTGTCCGAGGGCAACATTATCTTTGAAGACTCATTTGAATTCCAGCCGGATCCCAATACATCAATCTGCATTTCTGCAGCATTCAACC : 7436
             AAGTATCAATCCTGTGCTTGTCCGAGGG AACATTATCTTTGAAGACTCATTTGAATTCCAGCCGGATCCCAATACATCAATCTGCATTTCTGCAGCATTCAACC

              7460         *       7480         *       7500         *       7520         *       7540         *       7560
S.lycopers : AGAAAAAACCCGAACTACCCCTGGCTTTACCATGTGAAATCATGAAAGGAATTGAACCATAAAGCCCAAAAGGCGACTCGTGAAGATACTCAAACACATCAAGAT : 7540
S.pennelli : AGAAAAAACCCGAACTACCCCTGGCTTTACCATGTGAAATCATGAAAGGAATTGAACCATAAAGCCCAAAAGGCGACTCGTGAAGATACTCAAACACATCAAGAT : 7541
             AGAAAAAACCCGAACTACCCCTGGCTTTACCATGTGAAATCATGAAAGGAATTGAACCATAAAGCCCAAAAGGCGACTCGTGAAGATACTCAAACACATCAAGAT

                     *       7580         *       7600         *       7620         *       7640         *       7660
S.lycopers : TAAATAACCTATAAGGCTCAGAATATTCCTCTACATTAGGCCCCTTAGTTGGTTTCAAAGCAAAACTAGTAGCACGTTCAGGAATGCCATAAACAAAATCTGCAC : 7645
S.pennelli : TAAATAACCTATAAGGCTCAGAATATTCCTCTACATTAGGCCCCTTAGTTGGTTTCAAAGCAAAACTAGTAGCATGTTCAGGAATGCCATAAACAAAATCTGCAC : 7646
             TAAATAACCTATAAGGCTCAGAATATTCCTCTACATTAGGCCCCTTAGTTGGTTTCAAAGCAAAACTAGTAGCA GTTCAGGAATGCCATAAACAAAATCTGCAC

                *       7680         *       7700         *       7720         *       7740         *       7760         *
S.lycopers : CATAAAAAGATACATCAAAACTAATTGATTGTGGACCATAAGGCCTTGTATCAGTATGACTCCTAAACTTCTCCTCCCAATCATCCCCTTCTTTCTTCTCCCTCA : 7750
S.pennelli : CATAAAAAGATACATCAAAACTAATTGATTGTGGACCATAAGGCCTTGTATCAGTATGACTCCTAAACTTCTCCTCCCAATCATCCCCTTCTTTCTTCTCCCTCA : 7751
             CATAAAAAGATACATCAAAACTAATTGATTGTGGACCATAAGGCCTTGTATCAGTATGACTCCTAAACTTCTCCTCCCAATCATCCCCTTCTTTCTTCTCCCTCA

                     7780         *       7800         *       7820         *       7840         *       7860         *
S.lycopers : ACTGTTCAAAAGCAAACAACCCATTTGAGTTTATGGACAACACTCTCTTCCCACTTCCACTCTCTCTGGCAAAAACCTCAAATGGGTCATGTCTCAACACCCCTT : 7855
S.pennelli : ACTGTTCAAAAGCAAACAACCCATTTGAGTTTATGGACAACACTCTCTTCCCACTTCCACTCTCTCTGGCAAAAACCTCAAATGGGTCATGTCTCAACACCCCTT : 7856
             ACTGTTCAAAAGCAAACAACCCATTTGAGTTTATGGACAACACTCTCTTCCCACTTCCACTCTCTCTGGCAAAAACCTCAAATGGGTCATGTCTCAACACCCCTT

              7880         *       7900         *       7920         *       7940         *       7960         *       7980
S.lycopers : CATACCCATCAGACAAGTAAAAAACAGACGAAGAACTCGAACCTCCGTCGATTTGCTCCTCCTTTACTCTGGTTAACCACAGCTTAGTGTTGAGGAAATCTTCCT : 7960
S.pennelli : CATACCCATCAGACAAGTAAAAAACAGACGAAGAACTCGAACCTCCGTCGATTTGCTCCTCCTTTACTCTGGTTAACCACAGCTTAGTGTTGAGGAAATCTTCCT : 7961
             CATACCCATCAGACAAGTAAAAAACAGACGAAGAACTCGAACCTCCGTCGATTTGCTCCTCCTTTACTCTGGTTAACCACAGCTTAGTGTTGAGGAAATCTTCCT

                     *       8000         *       8020         *       8040         *       8060         *       8080
S.lycopers : CAATCACCTCAGGAACTTCAAATCTTTTCTTGGGTGGATTCAAATTTTGATCTTCATCAATTTTCACCCTCATCACACCATCTTGGTAGACGGAAAGAGTGAGAA : 8065
S.pennelli : CAATCACCTCAGGAACTTCAAATCTTTTCTTGGGTGGATTCAAATTTTGATCTTCATCAATTTTCACCCTCATCACACCATCTTGGTAGACGGAAAGAGTGAGAA : 8066
             CAATCACCTCAGGAACTTCAAATCTTTTCTTGGGTGGATTCAAATTTTGATCTTCATCAATTTTCACCCTCATCACACCATCTTGGTAGACGGAAAGAGTGAGAA

                *       8100         *       8120         *       8140         *       8160         *       8180         *
S.lycopers : CTAAAGGCTTATTGGGTTGTTCACTTTCT---------TCTTTGGGGACAAGTTTGGCTATAAGATCTCCATCGGAGATGGACACATCGACAACCCGCAGATTGC : 8161
S.pennelli : CTAAAGGCTTATTGGGTTGTTCACTTTCTGGGTTTTCTTCTTTGGGGACAAGTTTGGCTATAAGATCTCCATCGGAGATGGACACATCGACAACCCGCAGATTGC : 8171
             CTAAAGGCTTATTGGGTTGTTCACTTTCT         TCTTTGGGGACAAGTTTGGCTATAAGATCTCCATCGGAGATGGACACATCGACAACCCGCAGATTGC

                     8200         *       8220         *       8240         *       8260         *       8280         *
S.lycopers : ACGATCCAGGTTTTCGGGAACGGGCTCTTTTGCAAAATGGGGTTTGGTCACAGTTTCGAAACTCCTCCTTCTTCCAGGAGTAGGCAGAGGTAGCTAATAGAAGGA : 8266
S.pennelli : ACGATCCAGGTTTTCGGGAACGGGCTCTTTTGCAAAATGGGGTTTGGTCACAGTTCCGAAACTCCTCCTTCTTCCAGGAGTAGGCAGAGGTAGCCAATAGAAGGA : 8276
             ACGATCCAGGTTTTCGGGAACGGGCTCTTTTGCAAAATGGGGTTTGGTCACAGTT CGAAACTCCTCCTTCTTCCAGGAGTAGGCAGAGGTAGC AATAGAAGGA

              8300         *       8320         *       8340         *       8360         *       8380         *       8400
S.lycopers : GGAGAAGGAGTGGATATAACAGTAGTGGAGCTCTCATGGTTATAATCGAAGATCAATGTGGAAAGATATCATAGAAGATCGAAATTCGGAGGGAACTCTGCAAGA : 8371
S.pennelli : GGAGAAGGAGTGGATATAACAGTAGTGGAGCTCTCATGGTTATAATCGAAGATCAATGTGGAAAGATATCATAGAAGATCGGAATTCGGAGGGAACTCTGCAAGA : 8381
             GGAGAAGGAGTGGATATAACAGTAGTGGAGCTCTCATGGTTATAATCGAAGATCAATGTGGAAAGATATCATAGAAGATCG AATTCGGAGGGAACTCTGCAAGA

                     *       8420         *       8440         *       8460         *
S.lycopers : CTGCAACCTAGAGGTGAATGCTCTCCTCTATTTTATGCACTCTATACAAACACTCAATTGGAAAGTCAATG : 8442
S.pennelli : CTACAACCTAGAGGTGAATGCTCTCCTCTATTTTATGCACTCTATACAAACACTCAATTGGAAAGTCAATG : 8452
             CT CAACCTAGAGGTGAATGCTCTCCTCTATTTTATGCACTCTATACAAACACTCAATTGGAAAGTCAATG
```

Promoter sequences

SGN-U261955-promoter (1000bp):

```
                         *        20         *        40         *        60         *        80         *       100
S.lycopers : GATTTAACCTAGGATTGCTATAAAAGGTAATAAATAAAAAGTATTGCTGAAATCAGTAATTATTTATTAAAATGTATCAATTTATGTTATTTTCCT-TTTTAAAAA :  105
S.pennelli : TATTTAACATAGGATTGCTATAAATGGTAATAAATAAAAAGTATTGTAAAAATTAGTAATTATTTATTAAAATTTATCAATTTATGTAATTTTTTCATTTTTAAAA :  106
              ATTTAAC TAGGATTGCTATAAA GGTAATAAATAAAAAGTATTG   AAAT AGTAATTATTTATTAAAAT TATCAATTTATGT ATTTT    TTTT AAAA

                  *       120         *       140         *       160         *       180         *       200         *
S.lycopers : TTGGGCTGGCCCGGCAAGCCTGTATCCCACATAATTGTTGGTTGAGCCTATCATTTTTCGACCCACACTAAAATTGGA-CTAGTCCGATCTGATCC---------- :  200
S.pennelli : GTGGTCTGGCCCGGCAAGCCTGTAGTC-ACCTACTTGTGGATTGAGCCGACCATTTTCCGACCCACAAAAAAAAAAAGCTAATCCAACCTGACCCATAAAATATC :  211
              TGG CTGGCCCGGCAAGCCTGTA  C AC TA TTGT G TTGAGCC A CATTTT CGACCCACA  AAAA    A CTA TCC A CTGA CC

                  220         *       240         *       260         *       280         *       300         *         3
S.lycopers : --------------TTAGCCCGAAAGAAATGAGCTAGCCCTTATTGACAGCTCTAATAAATAAATACTCAATATCCATGTTTTTTTACATGCCATTATAAACTTGA :  292
S.pennelli : AAAACCTGTATGAATTAGCTCGAATGGGGTGGGCTAGCCCATATTGACAGTCCTAATC--CCAGGACACCACATTAATAGATAAATACTCA-----ACAAACTTGA :  310
                            TTAGC CGAA G   TG GCTAGCCC TATTGACAG  CTAAT      A  AC C A AT  AT   T   TAC        A AAACTTGA

             20         *       340         *       360         *       380         *       400         *       420
S.lycopers : AAACAAACACTGATTTAAAAT------TTAAAATTTAAACA------------GCTAACACCTAGTAATATATTTCCATAAATATGCATTCTTAGTTTCTTACCAT :  380
S.pennelli : AAACAAACACTGATTTAAAATCAAAATTTAAAATTTGGATACCAAATTTTACGGCCAACACCTACTCATATATTTCCATAAATATGCATACTTAGTTTCTTACCAT :  416
             AAACAAACACTGATTTAAAAT      TTAAAATTT  A A            GC AACACCTA T ATATATTTCCATAAATATGCAT CTTAGTTTCTTACCAT

                  *       440         *       460         *       480         *       500         *       520         *
S.lycopers : TTACGGCAGAATAAAGAAGATCTTCTTCACACACACATAAATACCTCTTTATATATAGTCTACTGTCTACATACATTGCATTTTACCAGTGTGTCTCTTCAAACTA :  486
S.pennelli : TTACGGCAGAATAAAGAAGATCTTCTTCACACACACATAAATACCTCTTTATATATAG--TACTCTCTACATACATTGCATTTTACCAGTGTGTCTCTTCAAATTA :  520
             TTACGGCAGAATAAAGAAGATCTTCTTCACACACACATAAATACCTCTTTATATATAG  TACT TCTACATACATTGCATTTTACCAGTGTGTCTCTTCAAA TA

                     540         *       560         *       580         *       600         *       620         *
S.lycopers : AATCCATTGGTAATCAATCACCAGGTATACATTTCATCTTCCTTTTATTCTCTGTTTTTCAATTATTCAGTGAAACCAATTAGGTACTTTTGTTTTGTTTTGCATA :  592
S.pennelli : AATTCATTGCTAATTAATCACCAGGTATACATTTCATCTTCCTTTTATTCTCTGTTTTTCAATTATTCAGTGAAACCAATTAGGTACTTTTGTTTTGTTTTGCATA :  626
             AAT CATTG TAAT AATCACCAGGTATACATTTCATCTTCCTTTTATTCTCTGTTTTTCAATTATTCAGTGAAACCAATTAGGTACTTTTGTTTTGTTTTGCATA

              640         *       660         *       680         *       700         *       720         *       740
S.lycopers : TTTTGGGATCTGAGATATGATTTTCCCCCCCTTGTTTTATGAGTTATGAAGTAAGTTAAAATTATTTCTCATAGC-GAAGTCTTCCTCAATTTACTCAGATTTTCT :  697
S.pennelli : TTTTGGGATCTGAGATATGATTTTTTTCCC-TTGTTTTATGAGTTATGAAGTAAGTTAAAATTATTTCTCATAGTTAAAGTCTTTCTCCTTTCACACAGATTTTCT :  731
             TTTTGGGATCTGAGATATGATTTT   CCC TTGTTTTATGAGTTATGAAGTAAGTTAAAATTATTTCTCATAG   AAGTCTT CTC  TT AC CAGATTTTCT

                  *       760         *       780         *       800         *       820         *       840
S.lycopers : CGGGAAAACATCTTATTCTGTTTCTTTAATCACATATCTCGGATTTTTCGTCTCTGAATACAGAGAAGTCTTTGTTAGGGATCTAGGAATACCAAACACTATCCGT :  803
S.pennelli : CGGGAAAACATCTTATTCTGTTTCTTTAATCACATGTCTCGAATTTTTTGTCTCTGGATACAGAGTAGTCTTTGTTAGGGATCTAGGAGTACCAAACACTATCCAT :  837
             CGGGAAAACATCTTATTCTGTTTCTTTAATCACAT TCTCG ATTTTT GTCTCTG ATACAGAG AGTCTTTGTTAGGGATCTAGGA TACCAAACACTATCC T

               *       860         *       880         *       900         *       920         *       940         *
S.lycopers : GTATTTGGCAGAATTCTGTTAAAAATCTACCTAATACGTATAAATAATTTATCGAAAAAATTAATTGAACTGTTTTCTTTTTGTTAGAATCTAGAACTCATAAATT :  909
S.pennelli : GTGTTTGGCAGAATTCTGTTAAAAATCTACCTAATATGTATAAATAATTTAT---AAAAATTAATTGAACTGTTTTTTTTTTGTTAGAATCTAGAACTCATAAATT :  940
             GT TTTGGCAGAATTCTGTTAAAAATCTACCTAATA GTATAAATAATTTAT   AAAAATTAATTGAACTGTTTT TTTTTGTTAGAATCTAGAACTCATAAATT

                960         *       980         *      1000         *      1020         *      1040
S.lycopers : CAAAATTGTGGATTGTTACGGTTTGTAATGTAATCCGTTTTTTTTTATGTAGTACTGCTAATTAGTTTTTGTAA--TATTCAAAATCATGCAG : 1000
S.pennelli : CAAAATTGT---------CGGCTTGTAATGTAATCAGTT----TTTATGTAGTACTGCTAATTAGTTTTTGTAAATTATGCAGAAA-GTGAAA : 1019
             CAAAATTGT         CGG TTGTAATGTAATC GTT    TTTATGTAGTACTGCTAATTAGTTTTTGTAA  TAT CA AA   TG A
```

SGN-U242840-promoter (1000bp):

```
                      *        20         *        40         *        60         *        80         *       100
S.lycopers : AAGTTTATCGATAACTTTTCTTTAGAGACTTGAATGATAATATATTTTGATTGAGGGCCTGGATGTATGATAATGTACTTCTATAGACATTTTCACCTCAAAACTT :  106
S.pennelli : AAGTTTATCGATAACTTTTCTTTAGAGAATTGAATGATAATACATTTTGATTGAGGGCCTGAATGTATGATAATGTACTTCTATAGACATTTTCACCTCCAAACTT :  106
             AAGTTTATCGATAACTTTTCTTTAGAGA TTGAATGATAATA ATTTTGATTGAGGGCCTG ATGTATGATAATGTACTTCTATAGACATTTTCACCTC AAACTT

                *       120         *       140         *       160         *       180         *       200         *
S.lycopers : AGAAACACTCATTGGCAGTACTTTTTATGTTGCATCAGTAAT--GGCGGGTTAATTGTATGTGGTTTAATTAGTAATGGGTGGACAATGTATTGATTTATAAATTA :  210
S.pennelli : AGAAACACTCATTGGCAGTACTTTTTCTGTTGCATTAGTAATATGGCGGGTTAATTGGATGTGGTTTAATTAGTAATGGGTGGACAATGTATTGATTTATAAATTA :  212
             AGAAACACTCATTGGCAGTACTTTTT TGTTGCAT AGTAAT  GGCGGGTTAATTG ATGTGGTTTAATTAGTAATGGGTGGACAATGTATTGATTTATAAATTA

                  220         *       240         *       260         *       280         *       300         *       3
S.lycopers : TATTAATAAGTTATATTATAATAAATAATTGAGCGCCACATATTTTAAAAATATTTAAATAGTTTAAATTTAAAATCGTTAAACAAATGATCAATTTTGAGCCTAA :  316
S.pennelli : TATTAATAAGTTAAATTATAACAAATAATTGAGCGCCATATATTTTAAAAATATTTAAATAGTTTAAATTTAAAATCGTTAAACAAATGGTCAATTTTGAACCTAA :  318
             TATTAATAAGTTA ATTATAA AAATAATTGAGCGCCA ATATTTTAAAAATATTTAAATAGTTTAAATTTAAAATCGTTAAACAAATG TCAATTTTGA CCTAA

             20         *       340         *       360         *       380         *       400         *       420
S.lycopers : AGGTGGATGCCAATGGTATTTTGGAGCAAATACATAGATCAATGATATTTTTGTCCCATTTCTAATACCTTAAGGGTATTTTAAGCTCTTTTCCGTTTCAAAAAAA :  422
S.pennelli : AGGTGGATGTCAATGGTATTTTGGAGCCAATACATAGATGAATGATAGTTTTGTCCCAATTCTAATACTTTAAGGGTATTTTAGGCTCTTTTCCGTTTTAAAAAAA :  424
             AGGTGGATG CAATGGTATTTTGGAGC AATACATAGAT AATGATA TTTTGTCCCA TTCTAATAC TTAAGGGTATTTTA GCTCTTTTCCGTTT AAAAAAA

                  *       440         *       460         *       480         *       500         *       520         *
S.lycopers : TATGCACATTCAAATATTGAAAACAAATAATCGTATTGATTCAGTGTTCAAAATCTAAAATTTACATCTTAATATTCAAATGTGCATTTATAATTTGACATCTTAA :  528
S.pennelli : TATGCACATTCAAATATTGAAAACAAATAATCTTACTGATTCAGTGTTCAAAATCTAAAATTTACATCTTAATATTCAAATGTGCATTTATAATTTGACATCTTAA :  530
             TATGCACATTCAAATATTGAAAACAAATAATC TA TGATTCAGTGTTCAAAATCTAAAATTTACATCTTAATATTCAAATGTGCATTTATAATTTGACATCTTAA

                    540         *       560         *       580         *       600         *       620         *
S.lycopers : TCTTAATAAACACTTTAATA-------------------CATAAATTTTAATCTTAATGAAAACAATGAAACCTTAAGAGTCATACATGAGTATATACTATCTCG :  614
S.pennelli : TCTTAATACACAGTTTAATATTCAAATGTTATTCAAATTCAGAAATTTTAATCTTAATGAAAACAATGAAACATTA-GAGTCTTATCTGAGTATATACTATCTCC :  635
             TCTTAATA ACA TTTAATA                   CA AAATTTTAATCTTAATGAAAACAATGAAAC TTA GAGTC TA  TGAGTATATACTATCTC

              640         *       660         *       680         *       700         *       720         *       740
S.lycopers : GGTACACACCTTAGCCTCTTTCTACCTAACGACTTGGCCCTCAATTCACTTGTAACATCACCGTTCTTGCAAGTTAACATCTTAATTTAAGCATACTAGAGAAAAT :  720
S.pennelli : GCTACACACCTTAGCCTCTTTCTACTTAACGACTTAGCCCTCAATTCACTTGTAACATCACCGTTCTTGCAAGTTAACATCTTAATTTAAGCATACTAGAGAAAAT :  741
             G TACACACCTTAGCCTCTTTCTAC TAACGACTT GCCCTCAATTCACTTGTAACATCACCGTTCTTGCAAGTTAACATCTTAATTTAAGCATACTAGAGAAAAT

                    *       760         *       780         *       800         *       820         *       840
S.lycopers : CTCAAAATAAACACTAACACCATTACAATGCCAATTTTTTTTTTATAGTAATGCTAATATAAAATAGACCTTAAAATTCAAACAAAATCTAAGTAGGTATAATTTG :  826
S.pennelli : CTCAAAATTAACACTAACACCATTACAATGCCA---TTTATTTTATAGCAATGTTAATCTAAAATAAACCTTAAAATTCAAACCAAATCTAAGTAGGTATAATTTG :  844
             CTCAAAAT AACACTAACACCATTACAATGCCA   TTT TTTTATAG AATG TAAT TAAAATA ACCTTAAAATTCAAAC AAATCTAAGTAGGTATAATTTG

              *       860         *       880         *       900         *       920         *       940         *
S.lycopers : GTATTATAATTTCTAATGACATTGGTGTAAAAGGGCATTGACTTTCCAATTGAGTGTTTGTATAGAGTGCATAAAATAGAGGAGAGCATTCACCTCTAGGTTGCAG :  932
S.pennelli : GTATTATAATTTCTAATGACATTGGTGTAAAAGGGCATTGACTTTCCAATTGAGTGTTTGTATAGAGTGCATAAAATAGAGGAGAGCATTCACCTCTAGGTTGTAG :  950
             GTATTATAATTTCTAATGACATTGGTGTAAAAGGGCATTGACTTTCCAATTGAGTGTTTGTATAGAGTGCATAAAATAGAGGAGAGCATTCACCTCTAGGTTG AG

                960         *       980         *      1000         *      1020
S.lycopers : TCTTGCAGAGTTCCCTCCGAATTTCGATCTTCTATGATATCTTTCCACATTGATCTTCGATTATAACC : 1000
S.pennelli : TCTTGCAGAGTTCCCTCCGAATTCCGATCTTCTATGATATCTTTCCACATTGATCTTCGATTATAACC : 1018
             TCTTGCAGAGTTCCCTCCGAATT CGATCTTCTATGATATCTTTCCACATTGATCTTCGATTATAACC
```

SGN-U217186-promoter (1000bp):

```
                      *        20         *        40         *        60         *        80         *       100
S.lycopers : -------ATCAAACTATAATA---TTATATTATTTATAATAATTTTGTTTGTTATTCTCATATGATTTGCTATTGTAAGCTCCGATATAACATGAATATAACATTT :   96
S.pennelli : TCGTTTGATCAAACTATAATAATATTATATTATTTATAATAACTTTGTTTGTTATTTTCGTATGATTTGCTATTATAAGCTT-AGTATAACATGAATATAATATTT :  105
                    ATCAAACTATAATA   TTATATTATTTATAATAA TTTGTTTGTTATT TC TATGATTTGCTATT TAAGCT    TATAACATGAATATAA ATTT

                *       120         *       140         *       160         *       180         *       200         *
S.lycopers : AAAAAGATTGATTTAGGTAAATAAACTTATATTTTCACGTTGTATTTTAAAGATATAGTAAGTTATGTATCTTAACTTTTATAGGACTT-ATATCGACTAAGGCAA :  201
S.pennelli : AAAAAAAT--ATTTACGTAAATAAATTTATATTTTCACATTATATTTTAAAGATATAGTAAAGTATGTATCTTAACTTTTATAGAACTTTATATCGACTAAGGCAA :  209
             AAAAA AT  ATTTA GTAAATAAA TTATATTTTCAC TT TATTTTAAAGATATAGTAA  TATGTATCTTAACTTTTATAG ACTT ATATCGACTAAGGCAA

                  220         *       240         *       260         *       280         *       300         *       3
S.lycopers : AGAATTTAGTTTAGGTTTCATAAGTTTTTCCTTTTAGTTGAAATTTATTTAAAAAGATGGATATAGAACTTATAATCTATACGGCAAAAAGTTTGGGTTTATATCT :  307
S.pennelli : AGAATTTAGTTTAGGTTTCATAAGTTTCTT-TTTTAGTTGAAGTTTATTTAGAAACATGGATATAGAATTTATAATCTATAAGGCAAAAAGTTTGGGTTTATATTT :  314
             AGAATTTAGTTTAGGTTTCATAAGTTT T  TTTTAGTTGAA TTTATTTA AAA ATGGATATAGAA TTATAATCTATA GGCAAAAAGTTTGGGTTTATAT T

             20         *       340         *       360         *       380         *       400         *       420
S.lycopers : TACATGTTTCTCCTTTTATTTGAAGTTTATTAGACATGAATAAATCGAACTAAAAACATATTCACTGCGTCTATTTTTTGTCATATAGATTCCATTTTATAATATT :  413
S.pennelli : TACATGTTTCTCCTTTTATTTGAAATTTATTAGATACGAATGAATCGAACTAAAAACATATTCACTACGTCTACTTTTTGTCATATAGATTCCATTTTATAATATT :  420
             TACATGTTTCTCCTTTTATTTGAA TTTATTAGA A GAAT AATCGAACTAAAAACATATTCACT CGTCTA TTTTTGTCATATAGATTCCATTTTATAATATT

                  *       440         *       460         *       480         *       500         *       520         *
S.lycopers : ATCATTGGACTCCATTTATTAATTTTAATCTAGTTAAAAGTTATATTACAAAACTTCTATGTATATATACTTCTATTCTAGTATATACTATTACTCCGTCCGTTTT :  519
S.pennelli : ATCATTCGACTCCATTTATTAATTTTAATCTAGTTAAAAGTTATATTA-AAAACTTCTATGTATATATACTTCTATTATAGTATATATTACTACTCCGTCCATTTA :  525
             ATCATT GACTCCATTTATTAATTTTAATCTAGTTAAAAGTTATATTA AAAACTTCTATGTATATATACTTCTATT TAGTATATA TA TACTCCGTCC TTT

                    540         *       560         *       580         *       600         *       620         *
S.lycopers : AAAAAATATCTTTATTTCCTTTTAAGTCTATCGAAAATATGA------------TTTTTTTATAACATTTTAATTTTTATTTTCCACCTAACATGTTTAAAGTCAC :  613
S.pennelli : AAAGTTTGTCCCTATTTCCTTTTAAGTCTGTCAAAAGAATGAGCTTTTTTCTTCTTTTTTTATAATACTTTAATTTTTATTTTCCACATAGCATGTTTAAAATTAC :  631
             AAA   T TC  TATTTCCTTTTAAGTCT TC AAA  ATGA            TTTTTTTATAA A TTTAATTTTTATTTTCCAC TA CATGTTTAAA T AC

              640         *       660         *       680         *       700         *       720         *       740
S.lycopers : AAGATTAAACGACATTTTGGTATATCTGACATAACTTTAATTTAGAATCAGAAAATTAAAAAATCTTCTTTCTTTTCTTTTAACTCTATTCCAAGTCAAACTACGT :  719
S.pennelli : AAGATTTAAAGACATTTTGGTATATTTGACATAACTTTAATTTAGAATCACAAGATTAAAAAATCTTTTTTCTTTTCTTTTAATTTCGTTTCAAGTCAAACTAGGT :  737
             AAGATT AA GACATTTTGGTATAT TGACATAACTTTAATTTAGAATCA AA ATTAAAAAATCTT TTTCTTTTCTTTTAA T   TT CAAGTCAAACTA GT

                    *       760         *       780         *       800         *       820         *       840
S.lycopers : CATCATTTTTTAGACGAAGGGAGTATTTTGACTATTATTTGTTTTATATATATTTAAGTAAAACTTGAAAAACACTTAATCATAAGATATAAAAAGTTGTTTCAAT :  825
S.pennelli : CATCCTTTTTTAGACGAAGGGAGTATTTTTACTATTATTTGTTTTATATA--TTTAAGTTAAACTTGAAAAACACTTGATCATAAGATATAAAAAGTTATTTCAAT :  841
             CATC TTTTTTAGACGAAGGGAGTATTTT ACTATTATTTGTTTTATATA  TTTAAGT AAACTTGAAAAACACTT ATCATAAGATATAAAAAGTT TTTCAAT

              *       860         *       880         *       900         *       920         *       940         *
S.lycopers : TTATATTAATGATCGAATACAAAAATATAATTTCACTCCAAAATAAAATTTATCTTTTTATATAATCAAATGTCTTATTAATTTCATTTTTTTTTTTGAGAAAAAA :  931
S.pennelli : TTATATTAATGATCAAATACAAAAATATAATTTCACTCCAAAATAAAAATTATCTTTTTATATAATCAAATGTCTTATTAATTCATCTTTTTTTTTTGAGAAAAAA :  947
             TTATATTAATGATC AATACAAAAATATAATTTCACTCCAAAATAAAA TTATCTTTTTATATAATCAAATGTCTTATTAATT    TTTTTTTTTTGAGAAAAAA

                960         *       980         *      1000         *      1020
S.lycopers : AAA-GGAAAGAAAAAGAGATGCAAATAAAGGCAAAAGTATATACTTTAAGGGTGCAAAATGCAATTACTT : 1000
S.pennelli : AAAAGGAAAGAAAAAGAGATGCAAATAAAGGCAAAAGTATATACTTTAAGGGTGCAAAATGCAATTACTT : 1017
             AAA GGAAAGAAAAAGAGATGCAAATAAAGGCAAAAGTATATACTTTAAGGGTGCAAAATGCAATTACTT
```
