# Supplementary material for: Metabolic Profiling of a Mapping Population Exposes New Insights in the Regulation of Seed Metabolism and Seed, Fruit, and Plant Relations
Source: PLoS Genet. 2012 Mar 29;8(3):e1002612. doi: 10.1371/journal.pgen.1002612 (PMC3315483; doi:10.1371/journal.pgen.1002612)
Supplement: Text S3 — Confirmation of season I seed network via repetition of analysis on season II seed network. (PDF) [file pgen.1002612.s021.pdf]

Text S3. Confirming season I seed network via repetition of analysis on season II seed network.

To confirm the general observations for season I we repeated the analysis on season II. In total 50 different metabolites were consistently identified. Applying the same parameters as before we generated a network with 366 edges (Figure S5, Table S7). Though reducing the number of edges as compared to the season I seed network, the season II seed network still exceeded the degree of connectivity as compared to the fruit network – season I (14.64 as compared to 9.23, Table S7). The centrality of the amino acid module in the seed network was also confirmed in the second season (Figure S5).

The inter-seasonal network, generated can be viewed in Figure S7, were red edges represent conserved correlations throughout both seasons, green edges represent correlations solely occurring in season I and blue edges represent correlations solely occurring in season II. In total the network incorporates 63 nodes and 899 edges achieving a degree of connectivity of 28.8 and a network density of 0.46 (Table S7). To emphasize the conserved correlations we also constructed a network displaying the intersect of both seasons only (Figure S9). The network incorporates 44 of the 63 and 50 nodes, respectively, and 146 of the 689 and 322 edges, respectively, resulting in 21.1% conserved correlations for season I and 45.3% for season II. The topology of the network was overall maintained in both seasons. For instance the hub-like character of the amino acid module, central to the network in both years, is observed. The amino acid module, including all six aforementioned amino acids, accounts for 15 of the 44 nodes (34%), whereas 100 of the 146 edges (68.5%) are directly linked to one or multiple amino acids. Whilst the inter-seasonal analysis indicates highly conserved metabolic co-response across years, the overlap might be underestimated given the fact that the measurements during the season II were performed on seeds bulked from individual plants.
